# Performance of Large Language Models vs Conventional Machine Learning for Predicting Clinical Outcomes With Limited Data: Comparative Study

**DOI:** 10.2196/83853

**Published:** 2026-04-01

**Authors:** Erwan Bigan, Stéphane Dufour

**Affiliations:** 1 TinyPred Paris France

**Keywords:** large language model, LLM, machine learning, ML, small data

## Abstract

**Background:**

Machine learning (ML) can be used to predict clinical outcomes. Training predictive models typically requires data for hundreds or thousands of patients. Lowering this requirement to a few tens of patients would enable new applications in clinical trials (eg, optimizing the design of a phase III trial by training a predictive model on phase II data and applying it to synthetic phase III patients) or in clinical decision support systems (for rare diseases or narrow indications). Large language models (LLMs) have recently been shown to outperform conventional ML algorithms for predictions on tabular data when the train dataset is small.

**Objective:**

This study aims to investigate the advantage of LLMs compared with conventional ML for predicting clinical outcomes by applying state-of-the-art models to recently published clinical datasets.

**Methods:**

We compared 2 LLMs, 1 proprietary (from OpenAI) and 1 open source (from the Meta Llama family), with conventional ML classification algorithms to predict clinical outcomes using 3 recently published clinical datasets spanning distinct conditions (sepsis, gastric cancer, and acute leukemia). Datasets were chosen such that their publication date was after the LLM knowledge cutoff date to ensure that models were never exposed to these data during pretraining. Datasets were sampled to vary the training size.

**Results:**

On average, the 2 tested LLMs perform better than conventional ML for training sizes below 50 patients, using the receiver operating characteristic area under the curve, the *F*_1_-score, the average precision, and the balanced accuracy as metrics. Contextual information was found to be key to this advantage.

**Conclusions:**

These preliminary results may be further optimized. They already show the potential of LLM-based ML to enable new clinical use cases when data are available for only a few tens of patients.

## Introduction

Predictive modeling of patient outcome is attracting increased attention, with applications in patient triaging [1], clinical decision-making [2], and enriching clinical trial design with best responders to treatment [3] or with patients with the fastest disease progression [4]. Robust predictions typically require training data for hundreds or thousands of patients. However, there exist situations where such training data are not readily available and where robust predictive modeling with smaller training data would be desirable (eg, rare diseases, narrow indications, and decision-support systems trained on data from a single hospital). Robust predictive modeling with smaller training data could also open entirely new applications, such as the simulation of a phase III clinical trial outcome based on a predictive model trained on phase II data (with typical sample size below 50 patients [5]), which could be used to make predictions on synthetic patients and thus optimize trial design to maximize chances of success.

**Figure 1:**
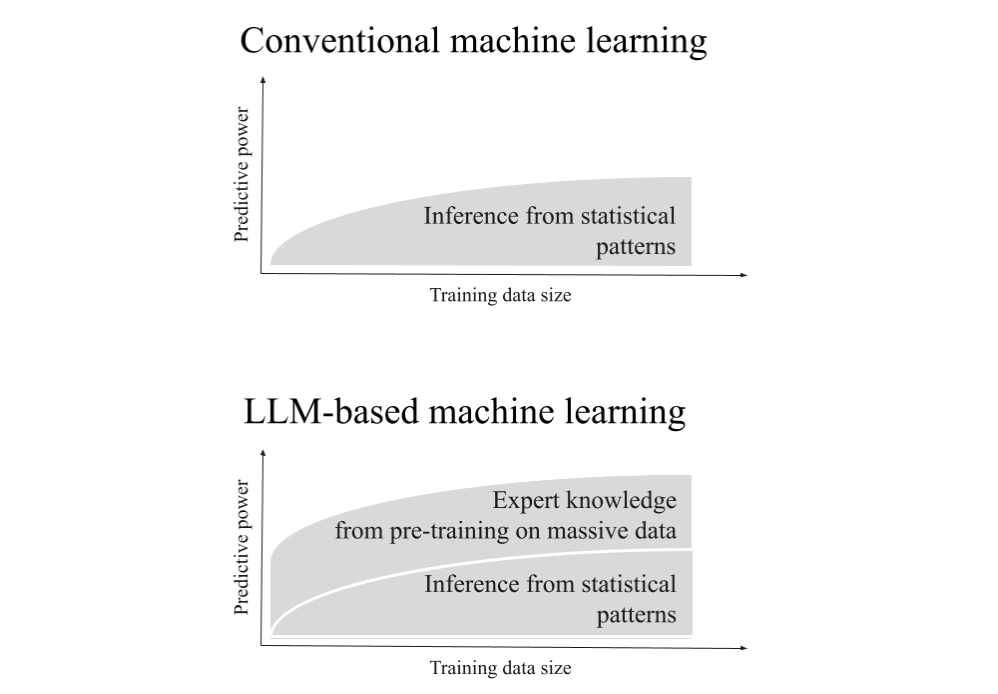
Schematic illustration of the predictive power as a function of train dataset size for conventional machine learning (top) and large language model (LLM)–based machine learning (bottom).

Hegselman et al [6] proposed using large language models (LLMs) to make predictions on tabular data, with enhanced performance compared with conventional machine learning (ML) when the training data are small. This is because LLMs are not only able to infer statistical patterns in tabular data (as conventional ML algorithms do), but they also benefit from domain knowledge acquired through their pretraining on massive data (typically the full internet). The relative advantage brought by this domain knowledge is even more important when the purely statistical signal is weak, which is typically the case when the training data are small (Figure 1).

This study follows the path laid out in this original proposal and assesses the performance of 2 state-of-the-art LLMs, 1 proprietary and 1 open-source, for classification tasks using published clinical datasets. A total of 3 clinical datasets [7-9] were selected such that their publication date was after the LLM knowledge cutoff date to ensure that they were never exposed to the LLMs during their pretraining and that the performance advantage does not result from data leakage (ie, prior knowledge of test data).

The predictive performance is assessed for various train dataset sizes (obtained by stratified sampling [10] of the original full datasets) and compared with that of conventional ML algorithms using the receiver operating characteristic area under the curve (ROC-AUC), which assesses the model discrimination capability (ie, the ability to rank test samples from lowest to highest probability regardless of model calibration); the *F*_1_-score, which is a composite score reflecting the tradeoff between precision and recall; the average precision (AP); and the balanced accuracy. Performance stability with respect to variations in the training set is also assessed.

Overall, LLMs tend to perform better than conventional ML. For the ROC-AUC, a statistically significant advantage is observed for training sizes of 10 or 20 samples for the sepsis and gastric cancer datasets and for a training size of 10 samples for the acute leukemia dataset. ROC-AUC point values in the 0.78 to 0.94 range are obtained with as few as 10 training samples. For other metrics, there are a few dataset, training size, and LLM-ML pair combinations where the advantage is not statistically significant, but there is no ML algorithm that consistently comes close to LLM performance.

## Methods

### Data

A total of 3 recently published clinical datasets were selected based on the following criteria: availability of patient-level data and publication date after the knowledge cutoff date of the used LLMs (as described in the “LLM Models” subsection).

Datasets were screened for near duplicates: 2 patients were considered near duplicates if their maximum absolute feature-wise difference did not exceed 5% of the corresponding feature SD. Near-duplicate pairs were detected using a radius-based nearest neighbor search, and such pairs were then checked for label concordance.

The first dataset [7] gives the mortality status within 30 days in sepsis patients admitted to the emergency department, along with sociodemographic and clinical characteristics collected at the time of admission. The dataset contains data for 1205 patients (of whom 191 died). There are no near duplicates or patients with missing data. Each patient is characterized by 20 features, 1 categorical (gender) and 19 numerical. To reflect the granularity of the raw data while avoiding creating unnecessarily long numerical strings for the LLM prompting, numerical features were either handled as integers (creatinine, N-terminal pro–B-type natriuretic peptide [NT-proBNP], platelets, fraction of inspired oxygen [FiO_2_], systolic blood pressure, diastolic blood pressure, age, and Glasgow Coma Scale score), rounded to the first decimal (activated partial thromboplastin time, blood sodium, C-reactive protein, hematocrit, partial pressure of oxygen [PaO_2_], total bilirubin, prothrombin time, lactic acid, and albumin), or rounded to the second decimal (procalcitonin and white blood cells), along with the applicable units.

The second dataset [8] gives the lymph node metastasis (LNM) status for patients who underwent gastric cancer surgery, along with sociodemographic and clinicopathological characteristics (collectively designated below as features). The original dataset contains data for 500 patients, including duplicates (2 patients), near duplicates (3 pairs, each with identical features and discordant label), and patients with missing data (30 patients), which were removed to keep 462 patients (of whom 258 had LNM), each characterized by 8 features. Four of these features are categorical: gender, tumor location (upper, lower, or middle), histology (intestinal, diffuse, or mixed/other), and signet-ring cell carcinoma (SRCC) status. Two are ordinal: tumor stage (T1a, T1b, T2, T3, T4a, or T4b) and tumor grade (G1, G2, or G3). Two are numerical: age and tumor size (both handled as integers, along with the applicable unit for tumor size).

The third dataset [9] gives the acute leukemia subtype for patients aged 18 years or older who were diagnosed with acute myeloid leukemia (AML), acute promyelocytic leukemia (APL), or acute lymphocytic leukemia. The original dataset contains data for 1410 patients, including duplicates (5 patients), near duplicates (2 pairs, each with concordant label and differing only in the precision of lactate dehydrogenase; for each such pair, only the patient with finer precision was retained), patients with missing data (187 patients), and patients with any zero-valued biomarker (356 patients), which were removed. Of the remaining 860 patients, 276 were diagnosed with acute lymphocytic leukemia and were also removed to focus on a binary classification task, leaving 584 patients (of whom 72 were diagnosed with APL), each characterized by 10 features, all numerical. These were either handled as integers (age), rounded to the first decimal (mean corpuscular volume, mean corpuscular hemoglobin concentration, lactate dehydrogenase, and platelets), or rounded to the second decimal (white blood cells, absolute neutrophil count, lymphocytes, monocytes, and fibrinogen), along with the applicable units. The original dataset also contained percentage values for some hematological parameters (neutrophils, lymphocytes, monocytes, and platelets), which were removed to retain only the absolute values.

Table 1 provides a summary of the properties of these datasets.

**Table 1:** Characteristics of the datasets used in this study.

Dataset	Patients, n		Features, n		
	Total	With outcome	Categorical	Ordinal	Numerical
Mortality by sepsis	1205	191	1	0	19
LNM^a^ status of gastric cancer	462	258	4	2	2
Acute leukemia subtype	584	72	0	0	10

^a^LNM: lymph node metastasis.

### Data Sampling

To reliably assess the performance of predictive models trained on very small samples of the above-described datasets (as small as a few tens of patients), a method compatible with the use of larger test samples was adopted. First, the training size (*TR*) and test size (*TS*) are chosen so that the latter is a multiple of the former: *TS* = *k* × *TR*. Second, a stratified sample *S* (stratified on the outcome to be predicted [10]) with size *TR* + *TS* = (*k* + 1) × *TR* is randomly selected from the full dataset. Third, this sample is randomly stratified into *k* + 1 folds, of which *n_splits_* (*n_splits_* ≤ *k* + 1) are randomly selected as training folds. For each such fold, the concatenation of the remaining *k* folds is used as a test set. The above-described process results in an inverted cross-validation and helps denoise the predictive model performance evaluation.

In standard cross-validation, training sets are mostly identical, whereas test sets are disjoint, and the variance across folds mostly reflects the variance across test sets. Pooling of test sets is therefore convenient to estimate CIs (using the DeLong method for ROC-AUC [11] or bootstrapping for any other metric). With the present inverted cross-validation, training sets are disjoint, whereas test sets are mostly identical, and the variance across folds mostly reflects the variance across training sets. Pooling such strongly overlapping test sets is therefore impractical.

For this reason, a large and fixed test size was chosen (large for CI estimation without pooling and fixed for fair comparison across train sizes or datasets). Starting from selected train sizes (*TR*=10, 20, or 50), the largest common multiple of these training sizes compatible with the size of the smallest dataset (gastric cancer) was chosen as the fixed test size, *TS*=400. A fixed number of folds was also chosen, defined as the maximum number of folds for the largest training size (*TR_max_*=50), which gives *n_splits_* = (*TR_max_* + *TS*) / *TR_max_* = 9.

Standard *scikit-learn* and *NumPy* libraries, initialized with the same random seed, were used for all random processes (*sklearn.model_selection.train_test_split* for initial stratified sampling of the full dataset, *sklearn.model_selection.StratifiedKFold* for splitting of this sample into *k* + 1 folds, and *numpy.random.choice* for choosing *n_splits_* out of these). This random seed was arbitrarily set to 1.

### Conventional ML Baseline

Logistic regression (LR), random forest (RF), and light gradient boosting machine (LGBM) were selected as the first set of algorithms for determining the conventional ML baseline. For LR and RF, their *scikit-learn* implementation [12] was used (*scikit-learn* version 1.7.2). For LGBM, the native implementation available as a Python package was used (*lightgbm* version 4.6.0). Nested cross-validation grid-search hyperparameter tuning was applied within each training set using an inner leave-one-out-cross-validation (LOOCV) for the smallest training sizes (*TR*≤20) or using 3-fold stratified inner cross-validation for larger training sizes (*TR*≥50), with standard scaling applied within the inner training to avoid any information leakage (Table S1 in Multimedia Appendix 1).

The best hyperparameters were selected based on the mean inner cross-validation ROC-AUC, and the corresponding models were retrained on the full training set before evaluation on the test set. All other hyperparameters beyond those spanned in the grid were left at their default values, except the *class_weight* parameter for LR and RF, which was set to *balanced*, and the *is_unbalance* parameter for LGBM, which was set to *True*, to automatically adjust weights inversely proportional to class frequencies in the input data. For the inner LOOCV folds having only the negative class present in the training set (case of the strongly imbalanced datasets, sepsis or acute leukemia, with the smallest training size, *TR*=10, resulting in a training set having only one positive, which is to be left out for one of the folds), the test probability was arbitrarily set to .5.

In addition to this initial set of algorithms, the recently proposed TabPFN [13] and TabICL [14] models, available as Python packages (*tabpfn* version 6.0.6 and *tabicl* version 0.1.4), were also included in the conventional ML baseline. These are transformer-based deep learning models that have been pretrained on synthetic datasets and are considered to outperform traditional ML algorithms.

One-hot encoding was used for categorical features, and ordinal encoding was used for ordinal features. As a preprocessing step before ML model fitting, features were normalized using standard scaling (ie, applying a linear transformation such that, over the training data, the mean is 0 and the SD is 1, with the same transformation applied to the test data).

### LLM Models

The LLMs used in this study were (1) the proprietary GPT-4o from OpenAI [15] (model snapshot *gpt-4o-2024-08-06*), accessed via the OpenAI application programming interface (API; Chat Completions endpoint, */v1/chat/completions*) in January 2026 using the Python software development kit (SDK; *openai* version 1.9.0), with temperature=0; and (2) the open-source Llama-3.3-70B-Instruct model from Meta [16] (Hugging Face checkpoint *meta-llama/Llama-3.3-70B-Instruct*), served using vLLM [17,18] (*vllm* version 0.13.0) on dual NVIDIA A100 graphics processing units (GPUs; tensor parallelism=2) with default *dtype* settings and decoded using beam search (beam width=5), retaining only the top-1 completion for analysis.

Their knowledge cutoff date is October 2023 for GPT-4o and December 2023 for Llama 3 70B. Therefore, during pretraining, these models were never exposed to the datasets used in this study, which were published in January 2024 (sepsis dataset), May 2024 (acute leukemia dataset), and March 2025 (gastric cancer dataset).

### LLM Prompting

To avoid having to fine-tune LLM weights and optimize associated hyperparameters (which would be challenging given the very small training samples considered in this study), in-context learning was chosen. This consists of using the LLM as is and including all training data in the prompt for every new prediction [19]. One caveat of in-context learning is that it consumes more tokens (a token is the basic unit into which words are split, with 0.75 word per token for most tokenizers used in current LLMs), which increases prediction costs for proprietary LLMs (that have pricing per token) and GPU memory requirements for open source LLMs. Another intrinsic limit is the LLM context window size, which limits the number of tokens per prompt. However, even for the largest tested training size (*TR*=50), the resulting prompt lengths remained below 16,000 tokens, which is well below the context window size (128,000 tokens for both GPT-4o and Llama 3 70B models).

### Prompting With Full Context

The LLMs were prompted as shown in the example in Textbox 1 for the sepsis dataset. This prompt follows the standard chat structure for instruction-following LLMs (instruct LLMs), which distinguishes between system, user, and assistant messages. Instruct LLMs are models that, after an initial pretraining phase that makes them strong next-token predictors, have been further trained to follow specific instructions using the chat format structure [19,20]. All messages shown in Textboxes 1 and 2 are submitted to the LLM in a single prompt. The system message gives the general context and is followed by the training examples, each consisting of a user message giving the patient characteristics followed by an assistant message giving the outcome. The last message is a single user message giving the characteristics of the patient whose outcome is to be predicted by the LLM.

**Example of a prompt submitted to the LLM for the sepsis dataset, for training size=10. It consists of a series of messages: first, the system message giving the general context; then a series of alternating user and assistant messages corresponding to the training patients; and finally, a user message corresponding to the test patient, for which the LLM is invited to make a prediction.**

**System**

You will be given characteristics of a sepsis patient admitted to the emergency department.

Classify the patient outcome into one of the following categories: survives, or dies.

Return only the name of the category, and nothing else.

MAKE SURE your output is one of the two categories stated.

**User**

Age: 38, Glasgow Coma Scale score: 14, Activated partial thromboplastin time: 43.5 seconds, Creatinine: 352 µmol/L, Blood sodium: 143.3 mEq/L, NT-proBNP: 10000 pg/mL, Procalcitonin: 100.0 ng/mL, C-reactive protein: 200.0 mg/L, White blood cells: 13.69 k/µL, Hematocrit: 14.9%, Platelets: 26 k/µL, Partial pressure of oxygen (PaO2): 109.0 mmHg, Fraction of inspired oxygen (FiO2): 21%, Lactic acid: 0.9 mmol/L, Systolic blood pressure: 124 mmHg, Diastolic blood pressure: 84 mmHg, Albumin: 24.2 g/L, Total bilirubin: 68.2 µmol/L, Prothrombin time: 14.8 seconds, Gender: female. Sepsis patient ->

**Assistant**

dies

**User**

Age: 84, Glasgow Coma Scale score: 15, Activated partial thromboplastin time: 52.0 seconds, Creatinine: 220 µmol/L, Blood sodium: 145.2 mEq/L, NT-proBNP: 8264 pg/mL, Procalcitonin: 100.0 ng/mL, C-reactive protein: 155.7 mg/L, White blood cells: 16.11 k/µL, Hematocrit: 37.4%, Platelets: 42 k/µL, Partial pressure of oxygen (PaO2): 82.8 mmHg, Fraction of inspired oxygen (FiO2): 21%, Lactic acid: 4.0 mmol/L, Systolic blood pressure: 101 mmHg, Diastolic blood pressure: 59 mmHg, Albumin: 29.9 g/L, Total bilirubin: 5.3 µmol/L, Prothrombin time: 16.4 seconds, Gender: male. Sepsis patient ->

**Assistant**

survives

**User**

Age: 78, Glasgow Coma Scale score: 11, Activated partial thromboplastin time: 40.0 seconds, Creatinine: 126 µmol/L, Blood sodium: 143.4 mEq/L, NT-proBNP: 2193 pg/mL, Procalcitonin: 0.81 ng/mL, C-reactive protein: 95.4 mg/L, White blood cells: 2.06 k/µL, Hematocrit: 22.7%, Platelets: 89 k/µL, Partial pressure of oxygen (PaO2): 80.9 mmHg, Fraction of inspired oxygen (FiO2): 33%, Lactic acid: 2.7 mmol/L, Systolic blood pressure: 116 mmHg, Diastolic blood pressure: 66 mmHg, Albumin: 27.0 g/L, Total bilirubin: 13.8 µmol/L, Prothrombin time: 14.8 seconds, Gender: male. Sepsis patient ->

**Assistant**

survives

**User**

Age: 53, Glasgow Coma Scale score: 15, Activated partial thromboplastin time: 44.1 seconds, Creatinine: 88 µmol/L, Blood sodium: 141.3 mEq/L, NT-proBNP: 1935 pg/mL, Procalcitonin: 4.05 ng/mL, C-reactive protein: 132.6 mg/L, White blood cells: 11.23 k/µL, Hematocrit: 35.2%, Platelets: 140 k/µL, Partial pressure of oxygen (PaO2): 86.0 mmHg, Fraction of inspired oxygen (FiO2): 29%, Lactic acid: 2.5 mmol/L, Systolic blood pressure: 95 mmHg, Diastolic blood pressure: 55 mmHg, Albumin: 29.6 g/L, Total bilirubin: 167.9 µmol/L, Prothrombin time: 16.7 seconds, Gender: female. Sepsis patient ->

**Assistant**

survives

**User**

Age: 56, Glasgow Coma Scale score: 14, Activated partial thromboplastin time: 47.1 seconds, Creatinine: 53 µmol/L, Blood sodium: 131.1 mEq/L, NT-proBNP: 959 pg/mL, Procalcitonin: 11.63 ng/mL, C-reactive protein: 354.1 mg/L, White blood cells: 0.55 k/µL, Hematocrit: 24.4%, Platelets: 46 k/µL, Partial pressure of oxygen (PaO2): 143.0 mmHg, Fraction of inspired oxygen (FiO2): 33%, Lactic acid: 1.3 mmol/L, Systolic blood pressure: 92 mmHg, Diastolic blood pressure: 57 mmHg, Albumin: 28.3 g/L, Total bilirubin: 5.2 µmol/L, Prothrombin time: 14.2 seconds, Gender: female. Sepsis patient ->

**Assistant**

survives

**User**

Age: 88, Glasgow Coma Scale score: 15, Activated partial thromboplastin time: 41.5 seconds, Creatinine: 138 µmol/L, Blood sodium: 143.2 mEq/L, NT-proBNP: 2603 pg/mL, Procalcitonin: 0.68 ng/mL, C-reactive protein: 227.5 mg/L, White blood cells: 12.94 k/µL, Hematocrit: 30.7%, Platelets: 175 k/µL, Partial pressure of oxygen (PaO2): 103.0 mmHg, Fraction of inspired oxygen (FiO2): 40%, Lactic acid: 3.4 mmol/L, Systolic blood pressure: 124 mmHg, Diastolic blood pressure: 56 mmHg, Albumin: 25.7 g/L, Total bilirubin: 115.9 µmol/L, Prothrombin time: 16.4 seconds, Gender: male. Sepsis patient ->

**Assistant**

survives

**User**

Age: 70, Glasgow Coma Scale score: 15, Activated partial thromboplastin time: 49.1 seconds, Creatinine: 269 µmol/L, Blood sodium: 145.3 mEq/L, NT-proBNP: 5719 pg/mL, Procalcitonin: 27.29 ng/mL, C-reactive protein: 240.2 mg/L, White blood cells: 21.83 k/µL, Hematocrit: 39.3%, Platelets: 104 k/µL, Partial pressure of oxygen (PaO2): 128.0 mmHg, Fraction of inspired oxygen (FiO2): 33%, Lactic acid: 3.4 mmol/L, Systolic blood pressure: 110 mmHg, Diastolic blood pressure: 67 mmHg, Albumin: 24.8 g/L, Total bilirubin: 24.1 µmol/L, Prothrombin time: 16.8 seconds, Gender: male. Sepsis patient ->

**Assistant**

survives

**User**

Age: 22, Glasgow Coma Scale score: 11, Activated partial thromboplastin time: 41.9 seconds, Creatinine: 57 µmol/L, Blood sodium: 139.8 mEq/L, NT-proBNP: 4968 pg/mL, Procalcitonin: 0.78 ng/mL, C-reactive protein: 138.3 mg/L, White blood cells: 8.86 k/µL, Hematocrit: 19.9%, Platelets: 199 k/µL, Partial pressure of oxygen (PaO2): 74.4 mmHg, Fraction of inspired oxygen (FiO2): 21%, Lactic acid: 1.8 mmol/L, Systolic blood pressure: 108 mmHg, Diastolic blood pressure: 79 mmHg, Albumin: 27.1 g/L, Total bilirubin: 39.1 µmol/L, Prothrombin time: 14.1 seconds, Gender: female. Sepsis patient ->

**Assistant**

survives

**User**

Age: 50, Glasgow Coma Scale score: 14, Activated partial thromboplastin time: 39.4 seconds, Creatinine: 100 µmol/L, Blood sodium: 142.8 mEq/L, NT-proBNP: 73 pg/mL, Procalcitonin: 2.06 ng/mL, C-reactive protein: 116.6 mg/L, White blood cells: 14.2 k/µL, Hematocrit: 38.9%, Platelets: 266 k/µL, Partial pressure of oxygen (PaO2): 71.8 mmHg, Fraction of inspired oxygen (FiO2): 21%, Lactic acid: 1.5 mmol/L, Systolic blood pressure: 95 mmHg, Diastolic blood pressure: 51 mmHg, Albumin: 37.5 g/L, Total bilirubin: 11.3 µmol/L, Prothrombin time: 16.0 seconds, Gender: male. Sepsis patient ->

**Assistant**

survives

**User**

Age: 63, Glasgow Coma Scale score: 15, Activated partial thromboplastin time: 46.6 seconds, Creatinine: 69 µmol/L, Blood sodium: 150.7 mEq/L, NT-proBNP: 781 pg/mL, Procalcitonin: 100.0 ng/mL, C-reactive protein: 228.4 mg/L, White blood cells: 9.37 k/µL, Hematocrit: 34.2%, Platelets: 76 k/µL, Partial pressure of oxygen (PaO2): 100.0 mmHg, Fraction of inspired oxygen (FiO2): 21%, Lactic acid: 1.0 mmol/L, Systolic blood pressure: 138 mmHg, Diastolic blood pressure: 77 mmHg, Albumin: 26.8 g/L, Total bilirubin: 15.6 µmol/L, Prothrombin time: 16.5 seconds, Gender: female. Sepsis patient ->

**Assistant**

survives

**User**

Age: 79, Glasgow Coma Scale score: 15, Activated partial thromboplastin time: 34.2 seconds, Creatinine: 166 µmol/L, Blood sodium: 139.8 mEq/L, NT-proBNP: 3012 pg/mL, Procalcitonin: 2.49 ng/mL, C-reactive protein: 200.0 mg/L, White blood cells: 19.45 k/µL, Hematocrit: 37.6%, Platelets: 181 k/µL, Partial pressure of oxygen (PaO2): 60.7 mmHg, Fraction of inspired oxygen (FiO2): 21%, Lactic acid: 1.4 mmol/L, Systolic blood pressure: 140 mmHg, Diastolic blood pressure: 60 mmHg, Albumin: 25.4 g/L, Total bilirubin: 14.7 µmol/L, Prothrombin time: 14.9 seconds, Gender: male. Sepsis patient ->

**Prompt used when stripping away all context information for the same example shown in Textbox 1. C0 corresponds to activated partial thromboplastin time; C1, creatinine; C2, blood sodium; C3, N-terminal pro–B-type natriuretic peptide (NT-proBNP); C4, procalcitonin; C5, C-reactive protein; C6, white blood cells; C7, hematocrit; C8, platelets; C9, partial pressure of oxygen (PaO2); C10, fraction of inspired oxygen (FiO2); C11, lactic acid; C12, systolic blood pressure; C13, diastolic blood pressure; C14, age; C15, albumin; C16, total bilirubin; C17, prothrombin time; C18, Glasgow Coma Scale score; and C19, gender.**

**System**

You will be given characteristics of a sample.

Classify the sample outcome into one of the following categories: 0, or 1.

Return only the name of the category, and nothing else.

MAKE SURE your output is one of the two categories stated.

**User**

C0 is –0.26, C1 is 2.14, C2 is 0.15, C3 is 1.96, C4 is 1.5, C5 is 0.15, C6 is 0.43, C7 is –1.8, C8 is –1.22, C9 is 0.5, C10 is –0.93, C11 is –1.29, C12 is 0.95, C13 is 1.74, C14 is –1.13, C15 is –1.08, C16 is 0.41, C17 is –0.86, C18 is 0.07, C19 is 1.0. Outcome is ->

**Assistant**

1

**User**

C0 is 1.92, C1 is 0.76, C2 is 0.55, C3 is 1.41, C4 is 1.5, C5 is –0.45, C6 is 0.84, C7 is 0.93, C8 is –1.01, C9 is –0.68, C10 is –0.93, C11 is 1.67, C12 is –0.65, C13 is –0.56, C14 is 1.21, C15 is 0.5, C16 is –0.79, C17 is 0.72, C18 is 0.73, C19 is –1.0. Outcome is ->

**Assistant**

0

**User**

C0 is –1.16, C1 is –0.22, C2 is 0.17, C3 is –0.49, C4 is –0.78, C5 is –1.27, C6 is –1.5, C7 is –0.86, C8 is –0.37, C9 is –0.76, C10 is 0.84, C11 is 0.43, C12 is 0.4, C13 is 0.08, C14 is 0.9, C15 is –0.3, C16 is –0.63, C17 is –0.86, C18 is –1.92, C19 is –1.0. Outcome is ->

**Assistant**

0

**User**

C0 is –0.11, C1 is –0.62, C2 is –0.28, C3 is –0.57, C4 is –0.71, C5 is –0.77, C6 is 0.02, C7 is 0.66, C8 is 0.32, C9 is –0.53, C10 is 0.25, C11 is 0.24, C12 is –1.06, C13 is –0.93, C14 is –0.37, C15 is 0.42, C16 is 2.32, C17 is 1.01, C18 is 0.73, C19 is 1.0. Outcome is ->

**Assistant**

0

**User**

C0 is 0.66, C1 is –0.98, C2 is –2.44, C3 is –0.87, C4 is –0.53, C5 is 2.25, C6 is –1.75, C7 is –0.65, C8 is –0.95, C9 is 2.02, C10 is 0.84, C11 is –0.9, C12 is –1.27, C13 is –0.74, C14 is –0.21, C15 is 0.06, C16 is –0.79, C17 is –1.45, C18 is 0.07, C19 is 1.0. Outcome is ->

**Assistant**

0

**User**

C0 is –0.78, C1 is –0.1, C2 is 0.12, C3 is –0.36, C4 is –0.78, C5 is 0.53, C6 is 0.31, C7 is 0.11, C8 is 0.8, C9 is 0.23, C10 is 1.87, C11 is 1.1, C12 is 0.95, C13 is –0.84, C14 is 1.41, C15 is –0.66, C16 is 1.33, C17 is 0.72, C18 is 0.73, C19 is –1.0. Outcome is ->

**Assistant**

0

**User**

C0 is 1.18, C1 is 1.27, C2 is 0.57, C3 is 0.62, C4 is –0.17, C5 is 0.7, C6 is 1.79, C7 is 1.16, C8 is –0.17, C9 is 1.35, C10 is 0.84, C11 is 1.1, C12 is –0.02, C13 is 0.17, C14 is 0.5, C15 is –0.91, C16 is –0.43, C17 is 1.11, C18 is 0.73, C19 is –1.0. Outcome is ->

**Assistant**

0

**User**

C0 is –0.67, C1 is –0.94, C2 is –0.6, C3 is 0.38, C4 is –0.78, C5 is –0.69, C6 is –0.37, C7 is –1.2, C8 is 1.12, C9 is –1.05, C10 is –0.93, C11 is –0.43, C12 is –0.16, C13 is 1.28, C14 is –1.94, C15 is –0.27, C16 is –0.14, C17 is –1.54, C18 is –1.92, C19 is 1.0. Outcome is ->

**Assistant**

0

**User**

C0 is –1.31, C1 is –0.49, C2 is 0.04, C3 is –1.15, C4 is –0.75, C5 is –0.98, C6 is 0.52, C7 is 1.11, C8 is 2.03, C9 is –1.17, C10 is –0.93, C11 is –0.71, C12 is –1.06, C13 is –1.3, C14 is –0.52, C15 is 2.61, C16 is –0.68, C17 is 0.32, C18 is 0.07, C19 is –1.0. Outcome is ->

**Assistant**

0

**User**

C0 is 0.53, C1 is –0.82, C2 is 1.71, C3 is –0.93, C4 is 1.5, C5 is 0.54, C6 is –0.29, C7 is 0.54, C8 is –0.55, C9 is 0.09, C10 is –0.93, C11 is –1.19, C12 is 1.93, C13 is 1.09, C14 is 0.14, C15 is –0.36, C16 is –0.59, C17 is 0.82, C18 is 0.73, C19 is 1.0. Outcome is ->

**Assistant**

0

**User**

C0 is –2.65, C1 is 0.2, C2 is –0.6, C3 is –0.23, C4 is –0.74, C5 is 0.15, C6 is 1.39, C7 is 0.95, C8 is 0.88, C9 is –1.67, C10 is –0.93, C11 is –0.81, C12 is 2.06, C13 is –0.47, C14 is 0.95, C15 is –0.75, C16 is –0.61, C17 is –0.76, C18 is 0.73, C19 is –1.0. Outcome is ->

### Prompting Without Context

To differentiate predictive strength arising from prior expert knowledge from predictive strength arising solely from inference of statistical patterns, prompts were also run while keeping the same statistical patterns in the training data but removing all contextual information. This was achieved by (1) using a system message that sets the problem as a pure statistical inference task; (2) replacing feature names (or column names combining feature and value in the case of one-hot encoding of categorical features) with generic column codes; (3) standard scaling train values (ie, applying a linear transformation such that the mean is 0 and the SD is 1) followed by rounding to the second decimal, and applying this same transformation to the test data; (4) removing any feature unit; and (5) replacing the clinical outcomes with “0” and “1.” The resulting prompt is shown in Textbox 2 for the same examples shown with full context in Textbox 1.

### Prompting Variations

To identify which element of context influences predictive strength, 3 prompt variations between full context and without context were also run. Starting from prompts with full context, the first variation consists of removing units for all nondimensionless numerical features. Starting from this first variation, the second variation consists of permutating feature names, replacing every name with the next one (and replacing the last one by the first one). Starting from this second variation (or equivalently from the first one), the third variation consists of replacing feature names with generic column codes. For all 3 variations, the system message and the outcome parts of the user and assistant messages were left unchanged.

Prompting variations were run only for the 2 smallest training sizes (*TR*=10 and 20), for which the predictive strength difference with vs without context is highest. The resulting prompt variants for the same examples shown in Textboxes 1 and 2 are given in Tables S3-S5 in Multimedia Appendix 1. Prompt examples for the full range of variations from full context to without context are given in Tables S7-S11 in Multimedia Appendix 1 for the gastric cancer dataset and in Tables S12-S16 in Multimedia Appendix 1 for the acute leukemia dataset.

### Processing of the LLM Response

The response object returned by the API (OpenAI API for GPT-4o and vLLM API for Llama 3 70B) is then parsed to extract (1) the prediction (with context: “survives” or “dies” for the sepsis dataset, “negative” or “positive” for the gastric cancer dataset, and “AML” or “APL” for the acute leukemia dataset; without context: “0” or “1”) and (2) the corresponding log probability (logarithm of its probability), which is the sum of the log probabilities of the individual tokens making up the full returned message. This log probability is then exponentiated into a probability *P* = exp(logprob), and the probability of the complement outcome is assigned as 1 – *P*. For all tested LLMs and all training/test folds, the returned message always exactly matched 1 of the 2 possible outcomes. This is consistent with the use of instruct LLMs (ie, LLMs that have been trained to follow instructions) [19,20].

### Metrics

The ROC-AUC, *F*_1_-score, AP, and balanced accuracy were used as predictive performance metrics for both the LLM-based approach and conventional ML. The AP corresponds to the noninterpolated area under the precision–recall curve and avoids the optimistic bias associated with trapezoidal interpolation, particularly in imbalanced settings.

### Statistical Analysis

A total of 2 statistical analyses were designed to account for both variations in the training set and variations in the test set.

#### CIs for Mean Performance Across Folds

This first analysis focuses on the mean metric (or metric difference in the case of pairwise model comparison) across the 9 inverted cross-validation folds, using a paired (patient-level) bootstrap for the test-population component and a fold-resampling (cluster) bootstrap for the training-set choice component. To preserve correlation across folds resulting from the overlap between test sets for different folds, bootstrap samples were generated by resampling patients with replacement (stratified by outcome) from the full selected sample *S* (union of train and test sets, which is common to all folds) with subsequent masking to retain only the test set component. Fold resampling was applied to account for variability related to training-set choice, and the mean of fold-level estimates (after fold resampling) was stored as bootstrapped value. The 95% CI was extracted from the distribution of bootstrapped values (n=5000).

#### Prediction Intervals for a Single Realized Training–Test Split

To quantify the variability expected for a single realized experiment (ie, a randomly selected training fold and corresponding test set), we estimated prediction intervals using a hierarchical bootstrap procedure. For each replicate, a fold was randomly sampled to represent the training-set choice, and the corresponding test set was resampled with replacement (stratified by outcome). The metric (or metric difference in the case of pairwise model comparison) was computed on this resampled test set, and its distribution across replicates (n=20,000) was used to determine the prediction interval. In contrast to CIs for the mean across folds, these prediction intervals characterize the dispersion of performance that may be observed in an individual training–test realization.

### Calibration and Decision Curve Analysis

Various post hoc calibration methods were applied to raw LLM probabilities and compared using the Brier score loss: isotonic calibration [21], Platt calibration [22], and beta calibration [23]. Calibration was fitted on the training set using an inner LOOCV. The test probability of LOOCV folds having only the negative class present in the training set was arbitrarily set to .5 (strongly imbalanced datasets, sepsis or acute leukemia, with the smallest training size, TR=10, may result in a training set having only 1 positive, which is to be left out for one of the folds).

The net clinical benefit was evaluated using decision curve analysis (DCA) [24]. The mean net benefit was computed over the following clinically relevant probability intervals: .05 to .30 for sepsis mortality, .30 to .60 for gastric cancer LNM status, and .02 to .15 for acute leukemia APL vs AML diagnosis. These intervals are in line with previous studies for such conditions [25,26]. They are centered around the prevalence, and the lower intervals for acute leukemia APL diagnosis and sepsis mortality reflect the higher clinical stakes and urgency of action.

## Results

### LLMs Tend to Perform Better Than Conventional ML

**Figure 2:**
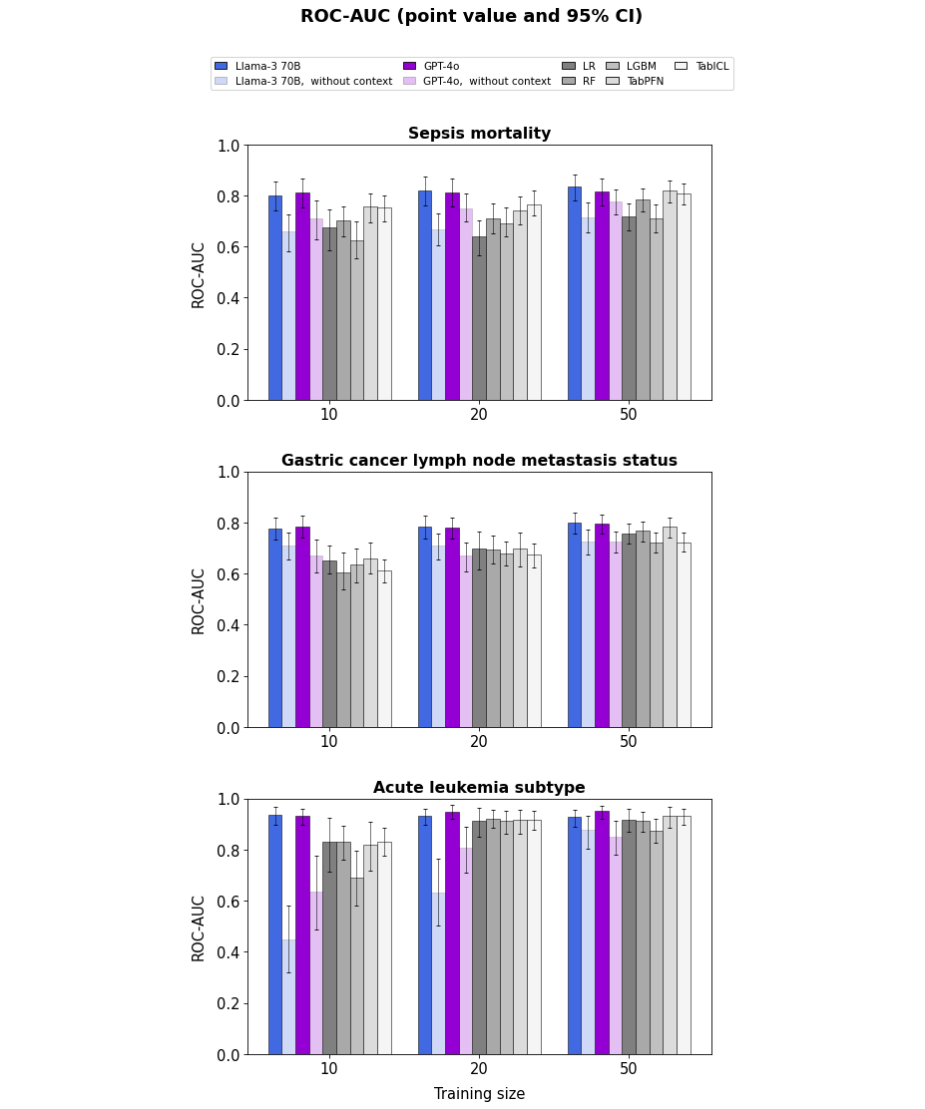
Point value and 95% CI for the mean receiver operating characteristic area under the curve (ROC-AUC) across folds, with or without context, as well as using conventional ML, for the sepsis (top), gastric cancer (middle), and leukemia (bottom) datasets. LGBM: light gradient boosting machine; LR: logistic regression; RF: random forest.

Figure 2 shows the point value and 95% CI for the mean ROC-AUC across folds as a function of training size for the GPT-4o and Llama 3 70B LLMs, with or without context, as well as for conventional ML algorithms. With context, and for the smallest training sizes (*TR*=10 and 20), point values are higher for LLMs than for conventional ML for all datasets. The same point value estimates are achieved with LLMs for a training size of *TR*=10 as with ML for a training size of *TR*=50. GPT-4o and Llama 3 70B both yield comparable ROC-AUCs.

The 95% CI in Figure 2 reflects the mixed effect of variations in the test population on the one hand and the choice of the training set (ie, choice of inverted cross-validation fold) on the other hand. The latter effect was found to be much lower for LLMs than for ML (approximately 2- to 15-fold lower SD across folds, depending on the dataset, training size, and considered LLM-ML pair; full set of ROC curves are available in Figures S1-S7 in Multimedia Appendix 1). This does not translate into any observable difference in CI width between LLMs and ML, presumably because the estimated value is the mean across the 9 folds, and this averaging reduces the impact on CI width.

This is not the case for the prediction interval, for which the estimated value corresponds to a single realization for a randomly selected fold (“Methods” section). Indeed, prediction intervals are relatively wider for conventional ML than for LLMs (Figure S32 vs Figure S8 in Multimedia Appendix 1 provide the ROC-AUC and Figures S34, S36, and S38 vs Figures S10, S12, and S14 in Multimedia Appendix 1 provide other metrics).

**Figure 3:**
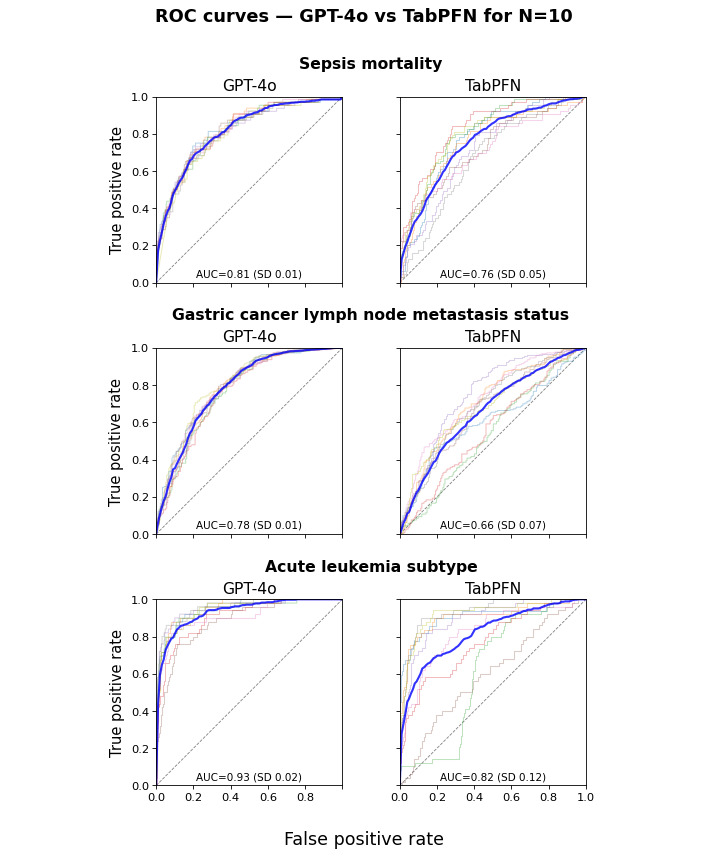
Receiver operating characteristic (ROC) curves for GPT-4o (left) and TabPFN (right) for the sepsis (top), gastric cancer (middle), and leukemia (bottom) datasets. The light-colored curves correspond to individual folds, and the blue curve represents the mean curve across folds.

This is illustrated in Figure 3 with the example comparison of receiver operating characteristic (ROC) curves for GPT-4o vs TabPFN for the smallest training size (*TR*=10). While there may exist specific training sets for which the performance of conventional ML approaches or even exceeds that of LLM-based ML, this is not the case for most training sets. In contrast, the LLM-based ML performance appears far more robust with respect to variations in the training set. Comparable patterns are observed for any LLM vs any conventional ML, with the smaller training sizes associated with stronger differences (Figures S1-S7 in Multimedia Appendix 1 provide the full set of ROC curves).

**Figure 4:**
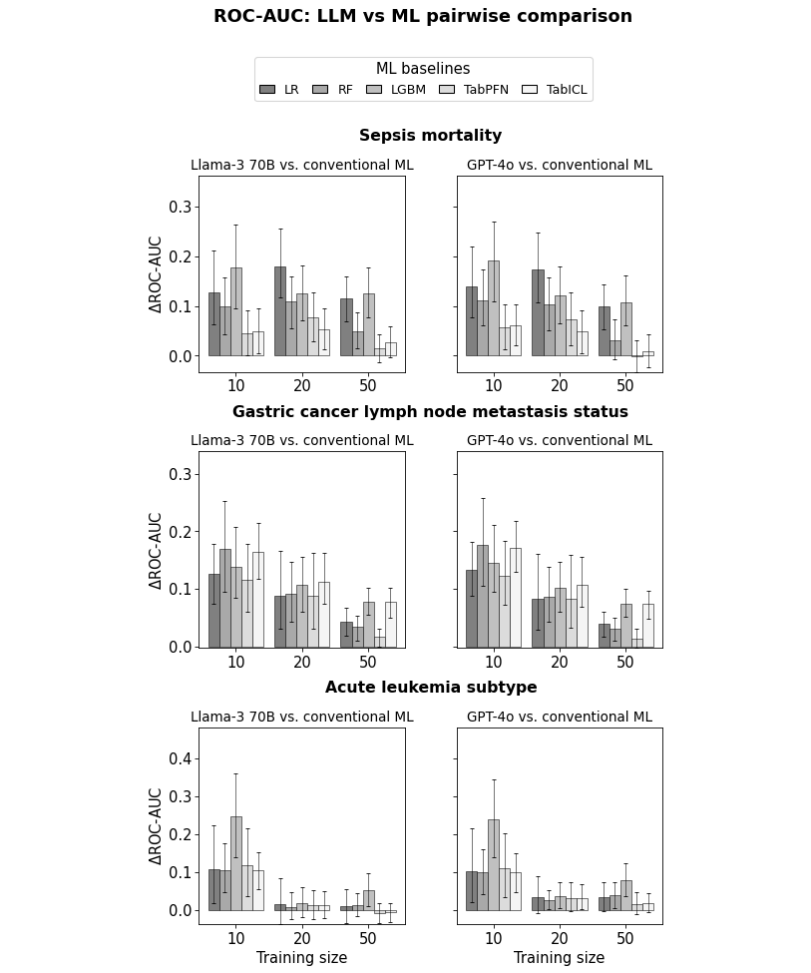
Difference in receiver operating characteristic area under the curve (ROC-AUC) between large language models (LLMs) and machine learning (ML) algorithms (point value and 95% CI) for the sepsis (top), gastric cancer (middle), and leukemia (bottom) datasets. LGBM: light gradient boosting machine; LR: logistic regression; RF: random forest.

Figure 4 shows the pairwise comparison between LLMs and conventional ML for the mean ROC-AUC across folds. For the smallest training sizes (*TR*=10 or 20 for sepsis mortality and gastric cancer LNM status, and *TR*=10 for acute leukemia subtype), the difference in mean ROC-AUC is significantly positive for any LLM-ML pair.

Point values and 95% CI, as well as pairwise comparisons, are given for the other metrics in Figures S10-S15 in Multimedia Appendix 1. There exist a few dataset, training size, and LLM–ML pair for which the LLM advantage is not statistically significant, but no ML algorithm consistently approaches the performance of LLMs.

### Context is Key to the LLM Performance Advantage

Figure 2 also shows that context is key to the LLM performance advantage, as removing it lowers the performance of LLMs to the level of conventional ML. This is consistent with the schematic illustration shown in Figure 1.

**Figure 5:**
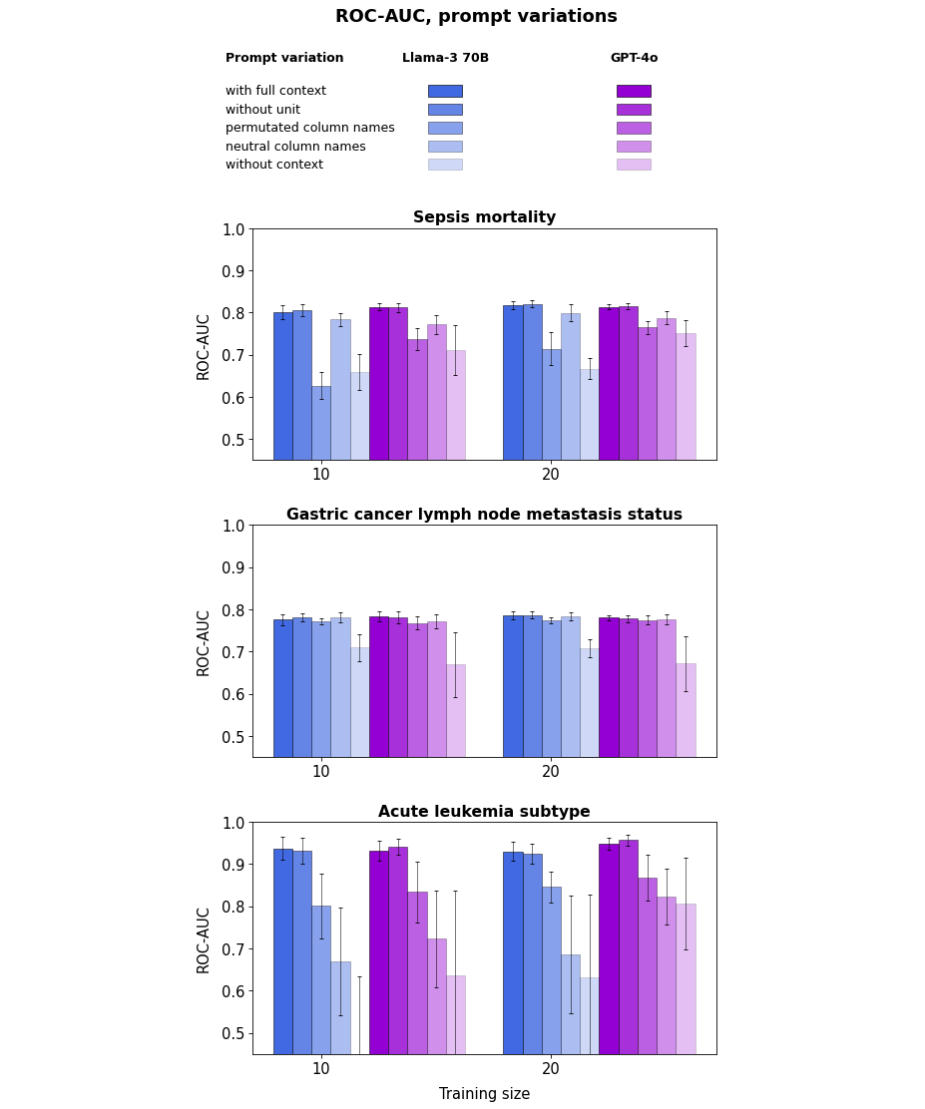
Large language model (LLM) receiver operating characteristic area under the curve (ROC-AUC) for various promptings (“Methods” section) for the sepsis (top), gastric cancer (middle), and leukemia (bottom) datasets. The point value represents the mean across folds, and the error bars represent the SD.

To further investigate which element of context contributes to this performance advantage, Figure 5 shows the ROC-AUC for both LLMs with or without context, as well as with the prompting variations described in the “Methods” section. Removing units for numerical features has no impact on ROC-AUC. This may reflect that units are inferred by the LLM from the range of values in the training set, or equivalently, that only values matter given the prevalence of standard units in the medical literature.

After removing units, either permutating feature names or replacing feature names with neutral column names reduces the ROC-AUC to a level between that observed with full context and that without context for the sepsis and leukemia datasets, but only marginally reduces it for the gastric cancer dataset. The reason why ROC-AUC is only marginally impacted for the gastric cancer dataset is that most features are categorical or ordinal, and feature textual values carry their own meaning (eg, T1a/b, T2, or T3 may be inferred as staging information, given that the system message conveys that the case concerns a patient with cancer).

For sepsis, permutating feature names degrades performance more than replacing them with neutral column names, whereas the opposite pattern is observed for leukemia. The reason for this pattern is unclear. One possible explanation is that, after permutation, the concordance between statistical associations of features with the target outcome and the LLM’s prior knowledge may be impacted differently depending on the specific permutation.

### LLMs Require Calibration

**Figure 6:**
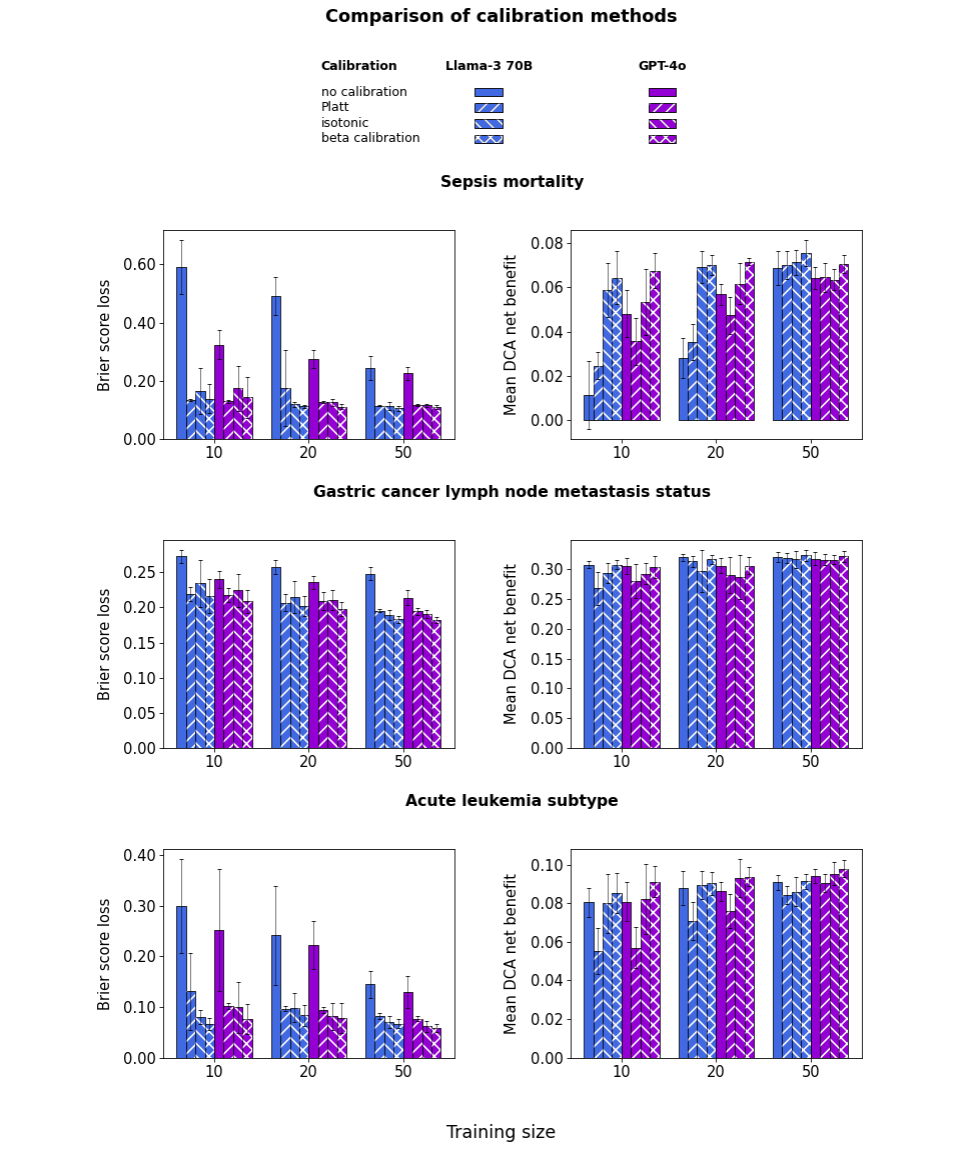
Large language model (LLM) Brier score loss (left) and decision curve analysis (DCA) mean net benefit (right) for the sepsis (top), gastric cancer (middle), and leukemia (bottom) datasets and various calibration methods. The point value represents the mean across folds, and the error bars represent the SD.

Figure 6 shows the Brier score loss and the DCA mean net benefit across the clinically relevant threshold interval (“Methods” section) for LLMs without calibration and after calibration with various methods. Without calibration, the Brier score loss is very high (ie, calibration is very poor). This reflects the fact that raw probabilities are sharply distributed around the extreme values of 0 or 1, with rare instances in between. After calibration, the Brier score loss becomes comparable to that of the best uncalibrated conventional ML algorithms. However, the mean DCA net benefit is clearly higher for isotonic or beta calibration than for Platt calibration. Platt calibration even lowers the DCA net benefit below that observed without calibration for the gastric cancer and acute leukemia datasets. This occurs because Platt calibration pushes all probabilities into a narrow interval, centered around .5 for the gastric cancer dataset and below .5 for the strongly imbalanced sepsis and acute leukemia datasets. Given that isotonic calibration introduces ties that slightly reduce ROC-AUC, the best overall results are achieved with beta calibration, which is therefore used in the results presented below.

**Figure 7:**
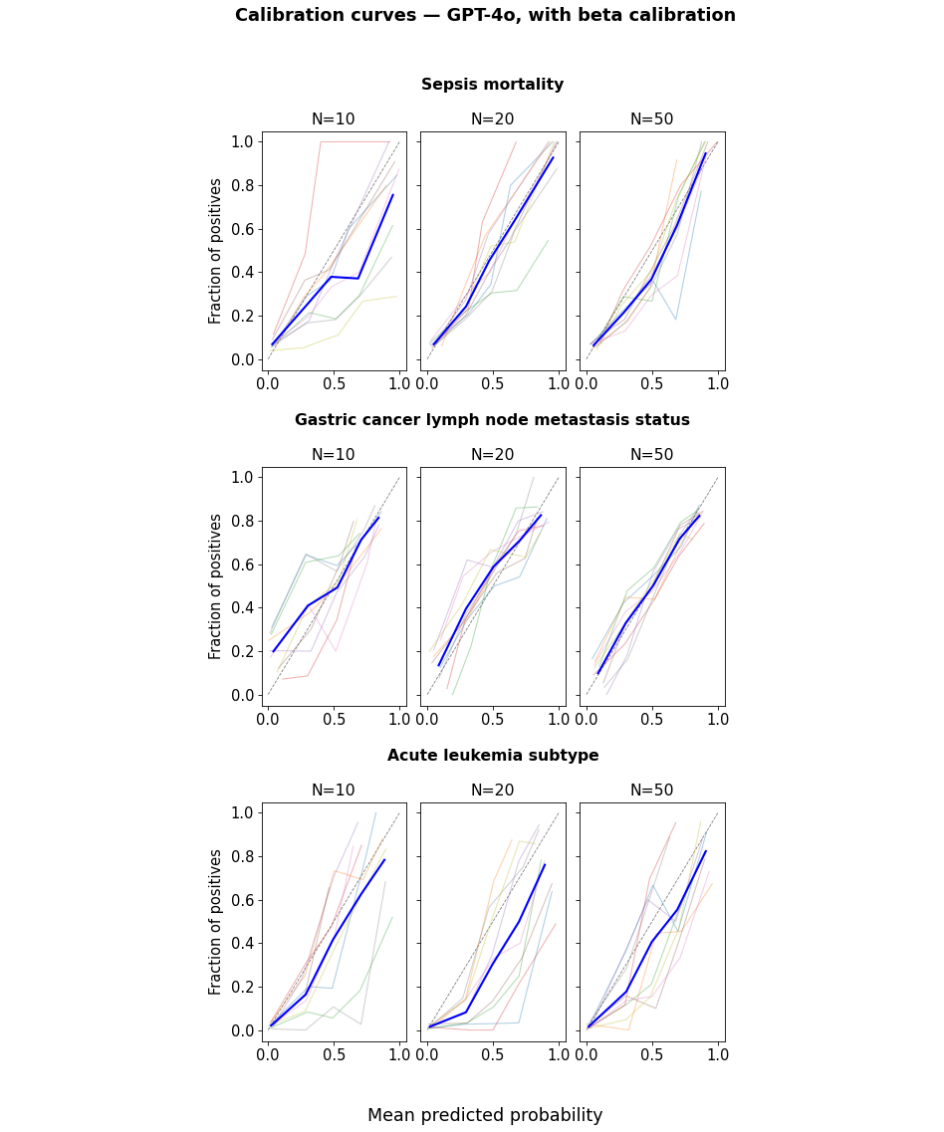
Calibration curves using 5 equal-width bins for GPT-4o with beta calibration for the sepsis (top), gastric cancer (middle), and leukemia (bottom) datasets. The light-colored curves correspond to individual folds, and the blue curve represents the mean curve across folds.

**Figure 8:**
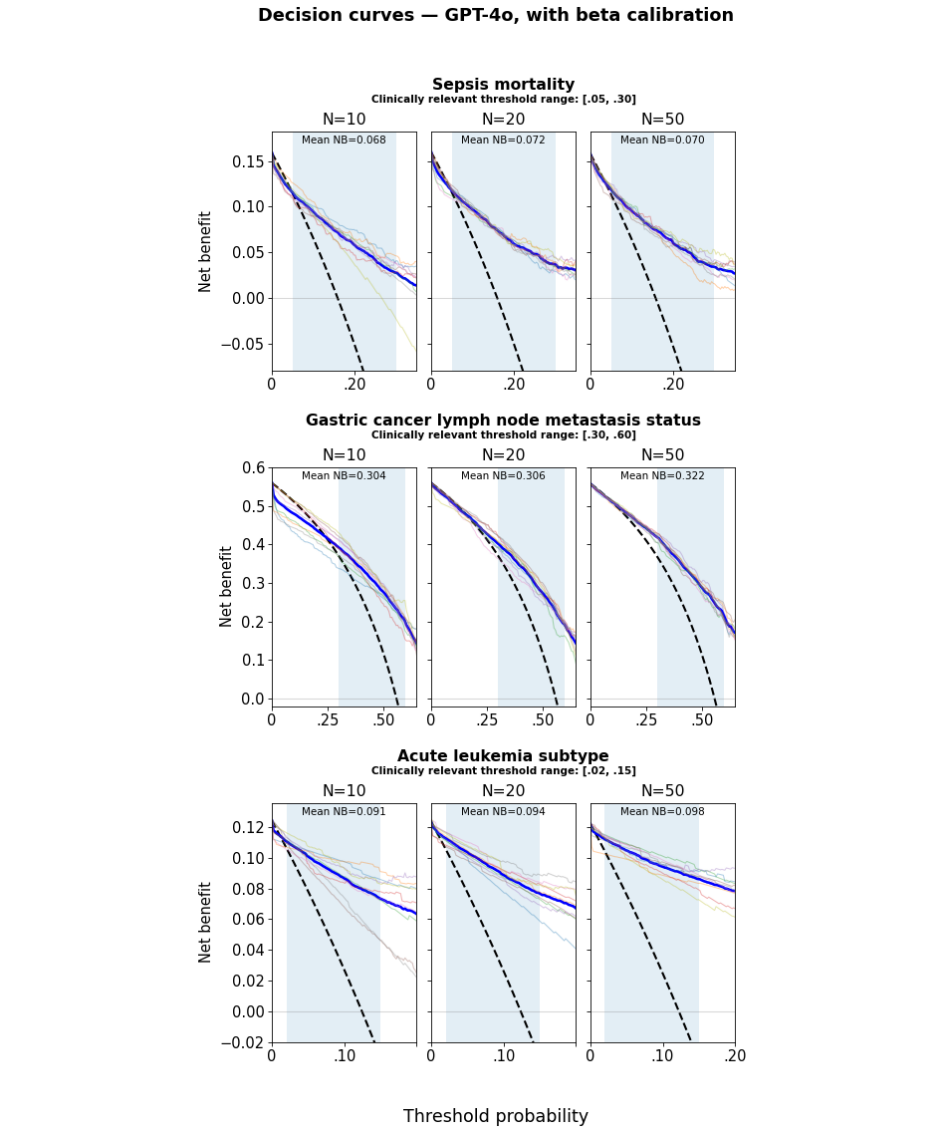
Decision curve analysis (DCA) net benefit curves for GPT-4o with beta calibration for the sepsis (top), gastric cancer (middle), and leukemia (bottom) datasets. The light-colored curves correspond to individual folds, and the blue curve represents the mean curve across folds. The black dashed line corresponds to the “treat all” strategy, and the thin horizontal line corresponds to the “treat none” strategy. The shaded area spans the clinically relevant threshold interval over which the mean net benefit is computed.

Calibration curves and DCA net benefit curves are provided in Figures S56-S68 and Figures S69-S81 in Multimedia Appendix 1 for all models and calibration methods and in Figures 7 and 8 for GPT-4o with beta calibration as an illustrative example. Figure 7 shows that the mean calibration curve across folds is reasonably well calibrated, although there is substantial dispersion across folds. Figure 8 shows the resulting net benefit advantage compared with the “treat all” strategy. The mean net benefit over the clinically relevant threshold interval increases only slightly with training size, but the dispersion of curves across folds (each corresponding to a different training set) decreases substantially. These curves illustrate the potential of LLMs for decision support system applications, even when training data are available only for small patient cohorts. The 95% CI for the mean net benefit, as well as pairwise comparisons between LLMs and conventional ML (Figures S30 and S31 in Multimedia Appendix 1), also confirms the LLM advantage over raw uncalibrated ML. However, this does not prove an absolute LLM advantage in this respect, as the conventional ML mean net benefit might itself be improved using post hoc calibration, which was not investigated in this study.

## Discussion

### Significance of Presented Results

Results obtained in this study confirm the potential of LLMs to reduce the number of patients needed to train predictive models. The performance in terms of both ROC-AUC and DCA mean net benefit illustrate this potential across a variety of use cases.

- The ROC-AUC reflects the ability of the model to properly rank patients from highest to lowest probability of outcome, without guaranteeing that probabilities are properly calibrated, as any monotonic transformation of the probability distribution leaves this metric unchanged. It is therefore a relevant metric for use cases requiring patient prioritization (eg, triaging patients from highest to lowest risk), but not for use cases in which the predictive model output would be used to guide treatment decisions for an individual patient.
- For use cases requiring the identification of a small fraction of the highest-risk patients within a total population, the lift can be an even more relevant metric [27]. As a first approximation, lift scales with the slope of the ROC curve near the origin. All else being equal, a steeper ROC curve translates into a higher ROC-AUC, although the relationship between these 2 metrics can be strongly influenced by the shape of the ROC curve.
- The DCA mean net benefit is a more relevant metric for use cases requiring not only discrimination of patients based on risk, but also reliable predictions for individual patients, with the minimization of false positives and false negatives, and the importance of proper calibration. Examples of such use cases include clinical decision support systems.

### Limitations

#### Testing With Limited Data

One of the challenges of predictive modeling on very small datasets is not only the limited data available to train a model but also the difficulty of reliably assessing predictive model performance. In this study, this challenge was circumvented by using larger test samples drawn from the full datasets. While this approach provides a sound estimation of predictive model performance, it could not be implemented if only truly limited data were available.

For example, if data for only 20 patients were available, it would be nearly impossible to reserve data for testing. LOOCV could be used to assess predictive strength, and leave-group-out cross-validation with Monte Carlo bootstrapping [28] (eg, leaving out a group of 2 patients—1 positive and 1 negative—with random sampling of all possible such 2-patient group choices, or even exhaustive testing in the case of very small datasets) could further be envisioned to assess model stability with respect to variations in the training–test split. However, these approaches would remain very noisy and may not reflect performance on larger test sets.

For clinical decision support systems, one approach to handle this challenge could be to share a predictive model trained on a small number of patients so that other health care providers can test it on their own data (akin to federated testing), thereby progressively building trust in the model’s predictive strength. For the phase III clinical trial simulation use case (training a predictive model on phase II data and applying it to synthetic phase III patients), one approach could consist of back-testing the overall use case on previous clinical trials for which phase II and phase III data are already available before applying it to a new phase III clinical trial design.

#### Influence of Treatment

Realization of the above-mentioned phase III clinical trial simulation use case would require more than what has been demonstrated in this study. In particular, it would require a single predictive model that includes treatment as one of its features and that properly accounts for treatment effects so that false positives and false negatives are minimized not only for the full trial population (treated or not) but also within each clinical trial arm.

In the worst-case scenario, having all false positives in the treatment arm and all false negatives in the control arm would lead to an overestimation of the odds ratio and of the expected statistical significance. In future work, the approach presented in this study could be further tested on datasets including a treatment variable.

#### Model Explainability

Several frameworks exist for ML explainability [29-31], the most widely used being Shapley additive explanations, which quantifies how much each feature contributes to the final prediction [30]. Although such frameworks are in principle equally applicable to conventional ML or LLM-based ML, they are computationally expensive because they require a large number of inferences.

Because inference is much more computationally expensive for the LLM-based ML approach than for conventional ML, we did not contemplate applying such frameworks in the context of this study.

As the field of LLMs is evolving rapidly, multiple trends toward faster LLM inference should eventually make it possible to apply Shapley additive explanations to the proposed LLM-based approach. Such trends include model size reduction through distillation [32] (training a smaller model using a larger model with only marginal performance loss) or quantization [33] (encoding model weights using fewer bits), innovations in transformer architecture such as speculative decoding [34], low-level optimization techniques such as kernel operator fusion [35], and advances in hardware evolution.

### Cost, Latency, Reproducibility, and Governance

At the time the experiments were conducted, the pricing of GPT-4o was US $2.5 per 1 million input tokens and US $10 per 1 million output tokens [15]. Considering input tokens first, and combining the system message and the user message for the test patient into an average token count per feature and per training patient, for all tested datasets and training sizes the token usage remained below 14 tokens per feature per training patient. For example, for sepsis mortality with 20 features and a training size of *TR*=20, the input token count remained below 5600, resulting in a per-prediction cost below US $0.014. The contribution of output tokens to the total cost is negligible because only a few output tokens are required per prediction. The observed latency was below 1 second per prediction.

For Llama 3 70B, a dual NVIDIA A100 GPU configuration, including disk storage, costs less than US $3 per hour on Runpod [36], which was used as the cloud computing platform for this study. The observed latency was below 2 seconds per prediction for all tested datasets and training sizes (with only marginal dependence on the dataset or training size), which translates to a marginal per-prediction cost below US $0.0017. For a fair comparison with OpenAI API costs, the one-off model deployment time and cost should also be considered in the case of this open-source model. This typically took less than 30 minutes, corresponding to a one-off setup cost of approximately US $1.50.

With respect to privacy and regulatory compliance, even if proprietary LLM providers such as OpenAI may offer deployment of their models on private clouds for certain cloud providers, open-source models are more flexible because they can be deployed on any private cloud or even on premises without any patient data leaving the health care provider’s environment. Similarly, reproducibility, reliability, and permanence of LLM access depend entirely on the LLM provider in the case of proprietary LLMs, whereas aspects can be fully controlled with open-source models for which weights and architecture have been publicly released.

### Comparison With Alternative Pretrained Approaches

There are alternative approaches that also leverage pretraining of deep learning models on large datasets to make predictions on smaller tabular datasets and that handle numerical values as numbers (rather than as text, as is the case for LLMs). Some approaches are generic (ie, applicable to any domain, similar to the LLM-based approach used in this study), whereas others are specialized for particular types of data.

Generic examples include TabPFN [13] or TabICL [14], which were included in the conventional ML baseline in this study. These foundation deep learning models for numerical tabular data have been pretrained on a wide variety of synthetic tabular datasets and have been shown to outperform conventional ML on datasets containing thousands of samples. While they have demonstrated a strong ability to infer statistical patterns on larger datasets, they are intrinsically unaware of the contextual information, which becomes crucial when only very limited data are available (eg, tens or hundreds of samples).

A specialized example has been reported by Ma et al [37]. In that study, a neural network model predicting drug response was pretrained on expansive datasets from cell-line screening experiments and then fine-tuned on smaller datasets from a different tissue or from patient-derived tumor cells to achieve superior predictive performance compared with state-of-the-art models. This approach requires developing specialized models for each new task, whereas the LLM-based ML approach offers the advantage of versatility (ie, a single model applicable to multiple tasks) and can benefit from innovations emerging from the broader LLM research community.

## Appendix

Multimedia Appendix 1 Additional tables and figures.

## Abbreviations

AML: acute myeloid leukemia
AP: average precision
API: application programming interface
APL: acute promyelocytic leukemia
DCA: decision curve analysis
FiO2: fraction of inspired oxygen
GPU: graphics processing unit
instruct LLM: instruction-following LLM
LGBM: light gradient boosting machine
LLM: large language model
LNM: lymph node metastasis
LOOCV: leave-one-out-cross-validation
LR: logistic regression
ML: machine learning
NT-proBNP: N-terminal pro–B-type natriuretic peptide
PaO2: partial pressure of oxygen
RF: random forest
ROC: receiver operating characteristic
ROC-AUC: receiver operating characteristic area under the curve
SDK: software development kit
SRCC: signet-ring cell carcinoma
TR: training size
TS: test size
