# Supplementary material for: Performance of Large Language Models vs Conventional Machine Learning for Predicting Clinical Outcomes With Limited Data: Comparative Study
Source: JMIR AI. 2026 Apr 1;5:e83853. doi: 10.2196/83853 (PMC13085988; doi:10.2196/83853)
Supplement: Multimedia Appendix 1 [file ai_v5i1e83853_app1.docx]

Supplementary Material

Conventional ML baseline hyperparameter grids

| Algorithm | Hyperparameter | Description | Values |
| --- | --- | --- | --- |
| Logistic Regression | C | Inverse of regularization strength | 0.1, *1.0*, 10.0 |
|  | Solver | Algorithm used in the optimization problem | *lbfgs*, liblinear |
| Random Forest | n_estimators | Number of trees in the forest | 50, *100* |
|  | max_depth | Maximum depth of the tree | 10, *None* |
|  | min_samples_leaf | Minimum number of samples required to be at a leaf node | *1*, 5 |
| LGBM | num_leaves | Maximum number of leaves in one tree | *31*, 50 |
|  | min_child_samples | Minimum number of samples in one leaf | 1, 5, *20* |
|  | max_depth | Maximum depth of the tree | *-1*, 10 |

Table S1: Hyperparameter grids used for tuning conventional ML algorithms. Default values from the source software libraries are italicized. For LGBM, the *min_child_samples* default value of 20 could only be used with sufficiently large train sizes (TR ≥ 50) to ensure possible splitting of the trees.

Example prompts

Sepsis dataset

| Role | Message |
| --- | --- |
| System | You will be given characteristics of a sepsis patient admitted to the emergency department.  Classify the patient outcome into one of the following categories: survives, or dies.  Return only the name of the category, and nothing else.  MAKE SURE your output is one of the two categories stated. |
| User | Age: 38, Glasgow Coma Scale score: 14, Activated partial thromboplastin time: 43.5 seconds, Creatinine: 352 µmol/L, Blood sodium: 143.3 mEq/L, NT-proBNP: 10000 pg/mL, Procalcitonin: 100.0 ng/mL, C-reactive protein: 200.0 mg/L, White blood cells: 13.69 k/µL, Hematocrit: 14.9%, Platelets: 26 k/µL, Partial pressure of oxygen (PaO2): 109.0 mmHg, Fraction of inspired oxygen (FiO2): 21%, Lactic acid: 0.9 mmol/L, Systolic blood pressure: 124 mmHg, Diastolic blood pressure: 84 mmHg, Albumin: 24.2 g/L, Total bilirubin: 68.2 µmol/L, Prothrombin time: 14.8 seconds, Gender: female. Sepsis patient -> |
| Assistant | dies |
| User | Age: 84, Glasgow Coma Scale score: 15, Activated partial thromboplastin time: 52.0 seconds, Creatinine: 220 µmol/L, Blood sodium: 145.2 mEq/L, NT-proBNP: 8264 pg/mL, Procalcitonin: 100.0 ng/mL, C-reactive protein: 155.7 mg/L, White blood cells: 16.11 k/µL, Hematocrit: 37.4%, Platelets: 42 k/µL, Partial pressure of oxygen (PaO2): 82.8 mmHg, Fraction of inspired oxygen (FiO2): 21%, Lactic acid: 4.0 mmol/L, Systolic blood pressure: 101 mmHg, Diastolic blood pressure: 59 mmHg, Albumin: 29.9 g/L, Total bilirubin: 5.3 µmol/L, Prothrombin time: 16.4 seconds, Gender: male. Sepsis patient -> |
| Assistant | survives |
| User | Age: 78, Glasgow Coma Scale score: 11, Activated partial thromboplastin time: 40.0 seconds, Creatinine: 126 µmol/L, Blood sodium: 143.4 mEq/L, NT-proBNP: 2193 pg/mL, Procalcitonin: 0.81 ng/mL, C-reactive protein: 95.4 mg/L, White blood cells: 2.06 k/µL, Hematocrit: 22.7%, Platelets: 89 k/µL, Partial pressure of oxygen (PaO2): 80.9 mmHg, Fraction of inspired oxygen (FiO2): 33%, Lactic acid: 2.7 mmol/L, Systolic blood pressure: 116 mmHg, Diastolic blood pressure: 66 mmHg, Albumin: 27.0 g/L, Total bilirubin: 13.8 µmol/L, Prothrombin time: 14.8 seconds, Gender: male. Sepsis patient -> |
| Assistant | survives |
| User | Age: 53, Glasgow Coma Scale score: 15, Activated partial thromboplastin time: 44.1 seconds, Creatinine: 88 µmol/L, Blood sodium: 141.3 mEq/L, NT-proBNP: 1935 pg/mL, Procalcitonin: 4.05 ng/mL, C-reactive protein: 132.6 mg/L, White blood cells: 11.23 k/µL, Hematocrit: 35.2%, Platelets: 140 k/µL, Partial pressure of oxygen (PaO2): 86.0 mmHg, Fraction of inspired oxygen (FiO2): 29%, Lactic acid: 2.5 mmol/L, Systolic blood pressure: 95 mmHg, Diastolic blood pressure: 55 mmHg, Albumin: 29.6 g/L, Total bilirubin: 167.9 µmol/L, Prothrombin time: 16.7 seconds, Gender: female. Sepsis patient -> |
| Assistant | survives |
| User | Age: 56, Glasgow Coma Scale score: 14, Activated partial thromboplastin time: 47.1 seconds, Creatinine: 53 µmol/L, Blood sodium: 131.1 mEq/L, NT-proBNP: 959 pg/mL, Procalcitonin: 11.63 ng/mL, C-reactive protein: 354.1 mg/L, White blood cells: 0.55 k/µL, Hematocrit: 24.4%, Platelets: 46 k/µL, Partial pressure of oxygen (PaO2): 143.0 mmHg, Fraction of inspired oxygen (FiO2): 33%, Lactic acid: 1.3 mmol/L, Systolic blood pressure: 92 mmHg, Diastolic blood pressure: 57 mmHg, Albumin: 28.3 g/L, Total bilirubin: 5.2 µmol/L, Prothrombin time: 14.2 seconds, Gender: female. Sepsis patient -> |
| Assistant | survives |
| User | Age: 88, Glasgow Coma Scale score: 15, Activated partial thromboplastin time: 41.5 seconds, Creatinine: 138 µmol/L, Blood sodium: 143.2 mEq/L, NT-proBNP: 2603 pg/mL, Procalcitonin: 0.68 ng/mL, C-reactive protein: 227.5 mg/L, White blood cells: 12.94 k/µL, Hematocrit: 30.7%, Platelets: 175 k/µL, Partial pressure of oxygen (PaO2): 103.0 mmHg, Fraction of inspired oxygen (FiO2): 40%, Lactic acid: 3.4 mmol/L, Systolic blood pressure: 124 mmHg, Diastolic blood pressure: 56 mmHg, Albumin: 25.7 g/L, Total bilirubin: 115.9 µmol/L, Prothrombin time: 16.4 seconds, Gender: male. Sepsis patient -> |
| Assistant | survives |
| User | Age: 70, Glasgow Coma Scale score: 15, Activated partial thromboplastin time: 49.1 seconds, Creatinine: 269 µmol/L, Blood sodium: 145.3 mEq/L, NT-proBNP: 5719 pg/mL, Procalcitonin: 27.29 ng/mL, C-reactive protein: 240.2 mg/L, White blood cells: 21.83 k/µL, Hematocrit: 39.3%, Platelets: 104 k/µL, Partial pressure of oxygen (PaO2): 128.0 mmHg, Fraction of inspired oxygen (FiO2): 33%, Lactic acid: 3.4 mmol/L, Systolic blood pressure: 110 mmHg, Diastolic blood pressure: 67 mmHg, Albumin: 24.8 g/L, Total bilirubin: 24.1 µmol/L, Prothrombin time: 16.8 seconds, Gender: male. Sepsis patient -> |
| Assistant | survives |
| User | Age: 22, Glasgow Coma Scale score: 11, Activated partial thromboplastin time: 41.9 seconds, Creatinine: 57 µmol/L, Blood sodium: 139.8 mEq/L, NT-proBNP: 4968 pg/mL, Procalcitonin: 0.78 ng/mL, C-reactive protein: 138.3 mg/L, White blood cells: 8.86 k/µL, Hematocrit: 19.9%, Platelets: 199 k/µL, Partial pressure of oxygen (PaO2): 74.4 mmHg, Fraction of inspired oxygen (FiO2): 21%, Lactic acid: 1.8 mmol/L, Systolic blood pressure: 108 mmHg, Diastolic blood pressure: 79 mmHg, Albumin: 27.1 g/L, Total bilirubin: 39.1 µmol/L, Prothrombin time: 14.1 seconds, Gender: female. Sepsis patient -> |
| Assistant | survives |
| User | Age: 50, Glasgow Coma Scale score: 14, Activated partial thromboplastin time: 39.4 seconds, Creatinine: 100 µmol/L, Blood sodium: 142.8 mEq/L, NT-proBNP: 73 pg/mL, Procalcitonin: 2.06 ng/mL, C-reactive protein: 116.6 mg/L, White blood cells: 14.2 k/µL, Hematocrit: 38.9%, Platelets: 266 k/µL, Partial pressure of oxygen (PaO2): 71.8 mmHg, Fraction of inspired oxygen (FiO2): 21%, Lactic acid: 1.5 mmol/L, Systolic blood pressure: 95 mmHg, Diastolic blood pressure: 51 mmHg, Albumin: 37.5 g/L, Total bilirubin: 11.3 µmol/L, Prothrombin time: 16.0 seconds, Gender: male. Sepsis patient -> |
| Assistant | survives |
| User | Age: 63, Glasgow Coma Scale score: 15, Activated partial thromboplastin time: 46.6 seconds, Creatinine: 69 µmol/L, Blood sodium: 150.7 mEq/L, NT-proBNP: 781 pg/mL, Procalcitonin: 100.0 ng/mL, C-reactive protein: 228.4 mg/L, White blood cells: 9.37 k/µL, Hematocrit: 34.2%, Platelets: 76 k/µL, Partial pressure of oxygen (PaO2): 100.0 mmHg, Fraction of inspired oxygen (FiO2): 21%, Lactic acid: 1.0 mmol/L, Systolic blood pressure: 138 mmHg, Diastolic blood pressure: 77 mmHg, Albumin: 26.8 g/L, Total bilirubin: 15.6 µmol/L, Prothrombin time: 16.5 seconds, Gender: female. Sepsis patient -> |
| Assistant | survives |
| User | Age: 79, Glasgow Coma Scale score: 15, Activated partial thromboplastin time: 34.2 seconds, Creatinine: 166 µmol/L, Blood sodium: 139.8 mEq/L, NT-proBNP: 3012 pg/mL, Procalcitonin: 2.49 ng/mL, C-reactive protein: 200.0 mg/L, White blood cells: 19.45 k/µL, Hematocrit: 37.6%, Platelets: 181 k/µL, Partial pressure of oxygen (PaO2): 60.7 mmHg, Fraction of inspired oxygen (FiO2): 21%, Lactic acid: 1.4 mmol/L, Systolic blood pressure: 140 mmHg, Diastolic blood pressure: 60 mmHg, Albumin: 25.4 g/L, Total bilirubin: 14.7 µmol/L, Prothrombin time: 14.9 seconds, Gender: male. Sepsis patient -> |

Table S2: Example of a prompt submitted to the LLM, with full context, for the sepsis dataset, for *TR* = 10. It consists in a series of messages: first, the system message giving the general context; then, a series of alternating user and assistant messages corresponding to the train patients; and finally, a user message corresponding to the test patient, for which the LLM is invited to make a prediction.

| Role | Message |
| --- | --- |
| System | You will be given characteristics of a sepsis patient admitted to the emergency department.  Classify the patient outcome into one of the following categories: survives, or dies.  Return only the name of the category, and nothing else.  MAKE SURE your output is one of the two categories stated. |
| User | Activated partial thromboplastin time: 43.5, Creatinine: 352, Blood sodium: 143.3, NT-proBNP: 10000, Procalcitonin: 100.0, C-reactive protein: 200.0, White blood cells: 13.69, Hematocrit: 14.9, Platelets: 26, Partial pressure of oxygen (PaO2): 109.0, Fraction of inspired oxygen (FiO2): 21, Lactic acid: 0.9, Systolic blood pressure: 124, Diastolic blood pressure: 84, Age: 38, Albumin: 24.2, Total bilirubin: 68.2, Prothrombin time: 14.8, Glasgow Coma Scale score: 14, Gender: female. Sepsis patient -> |
| Assistant | dies |
| User | Activated partial thromboplastin time: 52.0, Creatinine: 220, Blood sodium: 145.2, NT-proBNP: 8264, Procalcitonin: 100.0, C-reactive protein: 155.7, White blood cells: 16.11, Hematocrit: 37.4, Platelets: 42, Partial pressure of oxygen (PaO2): 82.8, Fraction of inspired oxygen (FiO2): 21, Lactic acid: 4.0, Systolic blood pressure: 101, Diastolic blood pressure: 59, Age: 84, Albumin: 29.9, Total bilirubin: 5.3, Prothrombin time: 16.4, Glasgow Coma Scale score: 15, Gender: male. Sepsis patient -> |
| Assistant | survives |
| User | Activated partial thromboplastin time: 40.0, Creatinine: 126, Blood sodium: 143.4, NT-proBNP: 2193, Procalcitonin: 0.81, C-reactive protein: 95.4, White blood cells: 2.06, Hematocrit: 22.7, Platelets: 89, Partial pressure of oxygen (PaO2): 80.9, Fraction of inspired oxygen (FiO2): 33, Lactic acid: 2.7, Systolic blood pressure: 116, Diastolic blood pressure: 66, Age: 78, Albumin: 27.0, Total bilirubin: 13.8, Prothrombin time: 14.8, Glasgow Coma Scale score: 11, Gender: male. Sepsis patient -> |
| Assistant | survives |
| User | Activated partial thromboplastin time: 44.1, Creatinine: 88, Blood sodium: 141.3, NT-proBNP: 1935, Procalcitonin: 4.05, C-reactive protein: 132.6, White blood cells: 11.23, Hematocrit: 35.2, Platelets: 140, Partial pressure of oxygen (PaO2): 86.0, Fraction of inspired oxygen (FiO2): 29, Lactic acid: 2.5, Systolic blood pressure: 95, Diastolic blood pressure: 55, Age: 53, Albumin: 29.6, Total bilirubin: 167.9, Prothrombin time: 16.7, Glasgow Coma Scale score: 15, Gender: female. Sepsis patient -> |
| Assistant | survives |
| User | Activated partial thromboplastin time: 47.1, Creatinine: 53, Blood sodium: 131.1, NT-proBNP: 959, Procalcitonin: 11.63, C-reactive protein: 354.1, White blood cells: 0.55, Hematocrit: 24.4, Platelets: 46, Partial pressure of oxygen (PaO2): 143.0, Fraction of inspired oxygen (FiO2): 33, Lactic acid: 1.3, Systolic blood pressure: 92, Diastolic blood pressure: 57, Age: 56, Albumin: 28.3, Total bilirubin: 5.2, Prothrombin time: 14.2, Glasgow Coma Scale score: 14, Gender: female. Sepsis patient -> |
| Assistant | survives |
| User | Activated partial thromboplastin time: 41.5, Creatinine: 138, Blood sodium: 143.2, NT-proBNP: 2603, Procalcitonin: 0.68, C-reactive protein: 227.5, White blood cells: 12.94, Hematocrit: 30.7, Platelets: 175, Partial pressure of oxygen (PaO2): 103.0, Fraction of inspired oxygen (FiO2): 40, Lactic acid: 3.4, Systolic blood pressure: 124, Diastolic blood pressure: 56, Age: 88, Albumin: 25.7, Total bilirubin: 115.9, Prothrombin time: 16.4, Glasgow Coma Scale score: 15, Gender: male. Sepsis patient -> |
| Assistant | survives |
| User | Activated partial thromboplastin time: 49.1, Creatinine: 269, Blood sodium: 145.3, NT-proBNP: 5719, Procalcitonin: 27.29, C-reactive protein: 240.2, White blood cells: 21.83, Hematocrit: 39.3, Platelets: 104, Partial pressure of oxygen (PaO2): 128.0, Fraction of inspired oxygen (FiO2): 33, Lactic acid: 3.4, Systolic blood pressure: 110, Diastolic blood pressure: 67, Age: 70, Albumin: 24.8, Total bilirubin: 24.1, Prothrombin time: 16.8, Glasgow Coma Scale score: 15, Gender: male. Sepsis patient -> |
| Assistant | dies |
| User | Activated partial thromboplastin time: 41.9, Creatinine: 57, Blood sodium: 139.8, NT-proBNP: 4968, Procalcitonin: 0.78, C-reactive protein: 138.3, White blood cells: 8.86, Hematocrit: 19.9, Platelets: 199, Partial pressure of oxygen (PaO2): 74.4, Fraction of inspired oxygen (FiO2): 21, Lactic acid: 1.8, Systolic blood pressure: 108, Diastolic blood pressure: 79, Age: 22, Albumin: 27.1, Total bilirubin: 39.1, Prothrombin time: 14.1, Glasgow Coma Scale score: 11, Gender: female. Sepsis patient -> |
| Assistant | survives |
| User | Activated partial thromboplastin time: 39.4, Creatinine: 100, Blood sodium: 142.8, NT-proBNP: 73, Procalcitonin: 2.06, C-reactive protein: 116.6, White blood cells: 14.2, Hematocrit: 38.9, Platelets: 266, Partial pressure of oxygen (PaO2): 71.8, Fraction of inspired oxygen (FiO2): 21, Lactic acid: 1.5, Systolic blood pressure: 95, Diastolic blood pressure: 51, Age: 50, Albumin: 37.5, Total bilirubin: 11.3, Prothrombin time: 16.0, Glasgow Coma Scale score: 14, Gender: male. Sepsis patient -> |
| Assistant | survives |
| User | Activated partial thromboplastin time: 46.6, Creatinine: 69, Blood sodium: 150.7, NT-proBNP: 781, Procalcitonin: 100.0, C-reactive protein: 228.4, White blood cells: 9.37, Hematocrit: 34.2, Platelets: 76, Partial pressure of oxygen (PaO2): 100.0, Fraction of inspired oxygen (FiO2): 21, Lactic acid: 1.0, Systolic blood pressure: 138, Diastolic blood pressure: 77, Age: 63, Albumin: 26.8, Total bilirubin: 15.6, Prothrombin time: 16.5, Glasgow Coma Scale score: 15, Gender: female. Sepsis patient -> |
| Assistant | survives |
| User | Activated partial thromboplastin time: 34.2, Creatinine: 166, Blood sodium: 139.8, NT-proBNP: 3012, Procalcitonin: 2.49, C-reactive protein: 200.0, White blood cells: 19.45, Hematocrit: 37.6, Platelets: 181, Partial pressure of oxygen (PaO2): 60.7, Fraction of inspired oxygen (FiO2): 21, Lactic acid: 1.4, Systolic blood pressure: 140, Diastolic blood pressure: 60, Age: 79, Albumin: 25.4, Total bilirubin: 14.7, Prothrombin time: 14.9, Glasgow Coma Scale score: 15, Gender: male. Sepsis patient -> |

Table S3: Example of a prompt submitted to the LLM, without units, for the same example as shown in Table S2.

| Role | Message |
| --- | --- |
| System | You will be given characteristics of a sepsis patient admitted to the emergency department.  Classify the patient outcome into one of the following categories: survives, or dies.  Return only the name of the category, and nothing else.  MAKE SURE your output is one of the two categories stated. |
| User | Creatinine: 43.5, Blood sodium: 352, NT-proBNP: 143.3, Procalcitonin: 10000, C-reactive protein: 100.0, White blood cells: 200.0, Hematocrit: 13.69, Platelets: 14.9, Partial pressure of oxygen (PaO2): 26, Fraction of inspired oxygen (FiO2): 109.0, Lactic acid: 21, Systolic blood pressure: 0.9, Diastolic blood pressure: 124, Age: 84, Albumin: 38, Total bilirubin: 24.2, Prothrombin time: 68.2, Glasgow Coma Scale score: 14.8, Gender: 14, Activated partial thromboplastin time: female. Sepsis patient -> |
| Assistant | dies |
| User | Creatinine: 52.0, Blood sodium: 220, NT-proBNP: 145.2, Procalcitonin: 8264, C-reactive protein: 100.0, White blood cells: 155.7, Hematocrit: 16.11, Platelets: 37.4, Partial pressure of oxygen (PaO2): 42, Fraction of inspired oxygen (FiO2): 82.8, Lactic acid: 21, Systolic blood pressure: 4.0, Diastolic blood pressure: 101, Age: 59, Albumin: 84, Total bilirubin: 29.9, Prothrombin time: 5.3, Glasgow Coma Scale score: 16.4, Gender: 15, Activated partial thromboplastin time: male. Sepsis patient -> |
| Assistant | survives |
| User | Creatinine: 40.0, Blood sodium: 126, NT-proBNP: 143.4, Procalcitonin: 2193, C-reactive protein: 0.81, White blood cells: 95.4, Hematocrit: 2.06, Platelets: 22.7, Partial pressure of oxygen (PaO2): 89, Fraction of inspired oxygen (FiO2): 80.9, Lactic acid: 33, Systolic blood pressure: 2.7, Diastolic blood pressure: 116, Age: 66, Albumin: 78, Total bilirubin: 27.0, Prothrombin time: 13.8, Glasgow Coma Scale score: 14.8, Gender: 11, Activated partial thromboplastin time: male. Sepsis patient -> |
| Assistant | survives |
| User | Creatinine: 44.1, Blood sodium: 88, NT-proBNP: 141.3, Procalcitonin: 1935, C-reactive protein: 4.05, White blood cells: 132.6, Hematocrit: 11.23, Platelets: 35.2, Partial pressure of oxygen (PaO2): 140, Fraction of inspired oxygen (FiO2): 86.0, Lactic acid: 29, Systolic blood pressure: 2.5, Diastolic blood pressure: 95, Age: 55, Albumin: 53, Total bilirubin: 29.6, Prothrombin time: 167.9, Glasgow Coma Scale score: 16.7, Gender: 15, Activated partial thromboplastin time: female. Sepsis patient -> |
| Assistant | survives |
| User | Creatinine: 47.1, Blood sodium: 53, NT-proBNP: 131.1, Procalcitonin: 959, C-reactive protein: 11.63, White blood cells: 354.1, Hematocrit: 0.55, Platelets: 24.4, Partial pressure of oxygen (PaO2): 46, Fraction of inspired oxygen (FiO2): 143.0, Lactic acid: 33, Systolic blood pressure: 1.3, Diastolic blood pressure: 92, Age: 57, Albumin: 56, Total bilirubin: 28.3, Prothrombin time: 5.2, Glasgow Coma Scale score: 14.2, Gender: 14, Activated partial thromboplastin time: female. Sepsis patient -> |
| Assistant | survives |
| User | Creatinine: 41.5, Blood sodium: 138, NT-proBNP: 143.2, Procalcitonin: 2603, C-reactive protein: 0.68, White blood cells: 227.5, Hematocrit: 12.94, Platelets: 30.7, Partial pressure of oxygen (PaO2): 175, Fraction of inspired oxygen (FiO2): 103.0, Lactic acid: 40, Systolic blood pressure: 3.4, Diastolic blood pressure: 124, Age: 56, Albumin: 88, Total bilirubin: 25.7, Prothrombin time: 115.9, Glasgow Coma Scale score: 16.4, Gender: 15, Activated partial thromboplastin time: male. Sepsis patient -> |
| Assistant | survives |
| User | Creatinine: 49.1, Blood sodium: 269, NT-proBNP: 145.3, Procalcitonin: 5719, C-reactive protein: 27.29, White blood cells: 240.2, Hematocrit: 21.83, Platelets: 39.3, Partial pressure of oxygen (PaO2): 104, Fraction of inspired oxygen (FiO2): 128.0, Lactic acid: 33, Systolic blood pressure: 3.4, Diastolic blood pressure: 110, Age: 67, Albumin: 70, Total bilirubin: 24.8, Prothrombin time: 24.1, Glasgow Coma Scale score: 16.8, Gender: 15, Activated partial thromboplastin time: male. Sepsis patient -> |
| Assistant | dies |
| User | Creatinine: 41.9, Blood sodium: 57, NT-proBNP: 139.8, Procalcitonin: 4968, C-reactive protein: 0.78, White blood cells: 138.3, Hematocrit: 8.86, Platelets: 19.9, Partial pressure of oxygen (PaO2): 199, Fraction of inspired oxygen (FiO2): 74.4, Lactic acid: 21, Systolic blood pressure: 1.8, Diastolic blood pressure: 108, Age: 79, Albumin: 22, Total bilirubin: 27.1, Prothrombin time: 39.1, Glasgow Coma Scale score: 14.1, Gender: 11, Activated partial thromboplastin time: female. Sepsis patient -> |
| Assistant | survives |
| User | Creatinine: 39.4, Blood sodium: 100, NT-proBNP: 142.8, Procalcitonin: 73, C-reactive protein: 2.06, White blood cells: 116.6, Hematocrit: 14.2, Platelets: 38.9, Partial pressure of oxygen (PaO2): 266, Fraction of inspired oxygen (FiO2): 71.8, Lactic acid: 21, Systolic blood pressure: 1.5, Diastolic blood pressure: 95, Age: 51, Albumin: 50, Total bilirubin: 37.5, Prothrombin time: 11.3, Glasgow Coma Scale score: 16.0, Gender: 14, Activated partial thromboplastin time: male. Sepsis patient -> |
| Assistant | survives |
| User | Creatinine: 46.6, Blood sodium: 69, NT-proBNP: 150.7, Procalcitonin: 781, C-reactive protein: 100.0, White blood cells: 228.4, Hematocrit: 9.37, Platelets: 34.2, Partial pressure of oxygen (PaO2): 76, Fraction of inspired oxygen (FiO2): 100.0, Lactic acid: 21, Systolic blood pressure: 1.0, Diastolic blood pressure: 138, Age: 77, Albumin: 63, Total bilirubin: 26.8, Prothrombin time: 15.6, Glasgow Coma Scale score: 16.5, Gender: 15, Activated partial thromboplastin time: female. Sepsis patient -> |
| Assistant | survives |
| User | Creatinine: 34.2, Blood sodium: 166, NT-proBNP: 139.8, Procalcitonin: 3012, C-reactive protein: 2.49, White blood cells: 200.0, Hematocrit: 19.45, Platelets: 37.6, Partial pressure of oxygen (PaO2): 181, Fraction of inspired oxygen (FiO2): 60.7, Lactic acid: 21, Systolic blood pressure: 1.4, Diastolic blood pressure: 140, Age: 60, Albumin: 79, Total bilirubin: 25.4, Prothrombin time: 14.7, Glasgow Coma Scale score: 14.9, Gender: 15, Activated partial thromboplastin time: male. Sepsis patient -> |

Table S4: Example of a prompt submitted to the LLM, with permutated column names, for the same example as shown in Tables S2-S3.

| Role | Message |
| --- | --- |
| System | You will be given characteristics of a sepsis patient admitted to the emergency department.  Classify the patient outcome into one of the following categories: survives, or dies.  Return only the name of the category, and nothing else.  MAKE SURE your output is one of the two categories stated. |
| User | C0: 43.5, C1: 352, C2: 143.3, C3: 10000, C4: 100.0, C5: 200.0, C6: 13.69, C7: 14.9, C8: 26, C9: 109.0, C10: 21, C11: 0.9, C12: 124, C13: 84, C14: 38, C15: 24.2, C16: 68.2, C17: 14.8, C18: 14, C19: female. Sepsis patient -> |
| Assistant | dies |
| User | C0: 52.0, C1: 220, C2: 145.2, C3: 8264, C4: 100.0, C5: 155.7, C6: 16.11, C7: 37.4, C8: 42, C9: 82.8, C10: 21, C11: 4.0, C12: 101, C13: 59, C14: 84, C15: 29.9, C16: 5.3, C17: 16.4, C18: 15, C19: male. Sepsis patient -> |
| Assistant | survives |
| User | C0: 40.0, C1: 126, C2: 143.4, C3: 2193, C4: 0.81, C5: 95.4, C6: 2.06, C7: 22.7, C8: 89, C9: 80.9, C10: 33, C11: 2.7, C12: 116, C13: 66, C14: 78, C15: 27.0, C16: 13.8, C17: 14.8, C18: 11, C19: male. Sepsis patient -> |
| Assistant | survives |
| User | C0: 44.1, C1: 88, C2: 141.3, C3: 1935, C4: 4.05, C5: 132.6, C6: 11.23, C7: 35.2, C8: 140, C9: 86.0, C10: 29, C11: 2.5, C12: 95, C13: 55, C14: 53, C15: 29.6, C16: 167.9, C17: 16.7, C18: 15, C19: female. Sepsis patient -> |
| Assistant | survives |
| User | C0: 47.1, C1: 53, C2: 131.1, C3: 959, C4: 11.63, C5: 354.1, C6: 0.55, C7: 24.4, C8: 46, C9: 143.0, C10: 33, C11: 1.3, C12: 92, C13: 57, C14: 56, C15: 28.3, C16: 5.2, C17: 14.2, C18: 14, C19: female. Sepsis patient -> |
| Assistant | survives |
| User | C0: 41.5, C1: 138, C2: 143.2, C3: 2603, C4: 0.68, C5: 227.5, C6: 12.94, C7: 30.7, C8: 175, C9: 103.0, C10: 40, C11: 3.4, C12: 124, C13: 56, C14: 88, C15: 25.7, C16: 115.9, C17: 16.4, C18: 15, C19: male. Sepsis patient -> |
| Assistant | survives |
| User | C0: 49.1, C1: 269, C2: 145.3, C3: 5719, C4: 27.29, C5: 240.2, C6: 21.83, C7: 39.3, C8: 104, C9: 128.0, C10: 33, C11: 3.4, C12: 110, C13: 67, C14: 70, C15: 24.8, C16: 24.1, C17: 16.8, C18: 15, C19: male. Sepsis patient -> |
| Assistant | dies |
| User | C0: 41.9, C1: 57, C2: 139.8, C3: 4968, C4: 0.78, C5: 138.3, C6: 8.86, C7: 19.9, C8: 199, C9: 74.4, C10: 21, C11: 1.8, C12: 108, C13: 79, C14: 22, C15: 27.1, C16: 39.1, C17: 14.1, C18: 11, C19: female. Sepsis patient -> |
| Assistant | survives |
| User | C0: 39.4, C1: 100, C2: 142.8, C3: 73, C4: 2.06, C5: 116.6, C6: 14.2, C7: 38.9, C8: 266, C9: 71.8, C10: 21, C11: 1.5, C12: 95, C13: 51, C14: 50, C15: 37.5, C16: 11.3, C17: 16.0, C18: 14, C19: male. Sepsis patient -> |
| Assistant | survives |
| User | C0: 46.6, C1: 69, C2: 150.7, C3: 781, C4: 100.0, C5: 228.4, C6: 9.37, C7: 34.2, C8: 76, C9: 100.0, C10: 21, C11: 1.0, C12: 138, C13: 77, C14: 63, C15: 26.8, C16: 15.6, C17: 16.5, C18: 15, C19: female. Sepsis patient -> |
| Assistant | survives |
| User | C0: 34.2, C1: 166, C2: 139.8, C3: 3012, C4: 2.49, C5: 200.0, C6: 19.45, C7: 37.6, C8: 181, C9: 60.7, C10: 21, C11: 1.4, C12: 140, C13: 60, C14: 79, C15: 25.4, C16: 14.7, C17: 14.9, C18: 15, C19: male. Sepsis patient -> |

Table S5: Example of a prompt submitted to the LLM, with neutral column names, for the same example as shown in Tables S2-S4.

| Role | Message |
| --- | --- |
| System | You will be given characteristics of a sample.  Classify the sample outcome into one of the following categories: 0, or 1.  Return only the name of the category, and nothing else.  MAKE SURE your output is one of the two categories stated. |
| User | C0 is -0.26, C1 is 2.14, C2 is 0.15, C3 is 1.96, C4 is 1.5, C5 is 0.15, C6 is 0.43, C7 is -1.8, C8 is -1.22, C9 is 0.5, C10 is -0.93, C11 is -1.29, C12 is 0.95, C13 is 1.74, C14 is -1.13, C15 is -1.08, C16 is 0.41, C17 is -0.86, C18 is 0.07, C19 is 1.0. Outcome is -> |
| Assistant | 1 |
| User | C0 is 1.92, C1 is 0.76, C2 is 0.55, C3 is 1.41, C4 is 1.5, C5 is -0.45, C6 is 0.84, C7 is 0.93, C8 is -1.01, C9 is -0.68, C10 is -0.93, C11 is 1.67, C12 is -0.65, C13 is -0.56, C14 is 1.21, C15 is 0.5, C16 is -0.79, C17 is 0.72, C18 is 0.73, C19 is -1.0. Outcome is -> |
| Assistant | 0 |
| User | C0 is -1.16, C1 is -0.22, C2 is 0.17, C3 is -0.49, C4 is -0.78, C5 is -1.27, C6 is -1.5, C7 is -0.86, C8 is -0.37, C9 is -0.76, C10 is 0.84, C11 is 0.43, C12 is 0.4, C13 is 0.08, C14 is 0.9, C15 is -0.3, C16 is -0.63, C17 is -0.86, C18 is -1.92, C19 is -1.0. Outcome is -> |
| Assistant | 0 |
| User | C0 is -0.11, C1 is -0.62, C2 is -0.28, C3 is -0.57, C4 is -0.71, C5 is -0.77, C6 is 0.02, C7 is 0.66, C8 is 0.32, C9 is -0.53, C10 is 0.25, C11 is 0.24, C12 is -1.06, C13 is -0.93, C14 is -0.37, C15 is 0.42, C16 is 2.32, C17 is 1.01, C18 is 0.73, C19 is 1.0. Outcome is -> |
| Assistant | 0 |
| User | C0 is 0.66, C1 is -0.98, C2 is -2.44, C3 is -0.87, C4 is -0.53, C5 is 2.25, C6 is -1.75, C7 is -0.65, C8 is -0.95, C9 is 2.02, C10 is 0.84, C11 is -0.9, C12 is -1.27, C13 is -0.74, C14 is -0.21, C15 is 0.06, C16 is -0.79, C17 is -1.45, C18 is 0.07, C19 is 1.0. Outcome is -> |
| Assistant | 0 |
| User | C0 is -0.78, C1 is -0.1, C2 is 0.12, C3 is -0.36, C4 is -0.78, C5 is 0.53, C6 is 0.31, C7 is 0.11, C8 is 0.8, C9 is 0.23, C10 is 1.87, C11 is 1.1, C12 is 0.95, C13 is -0.84, C14 is 1.41, C15 is -0.66, C16 is 1.33, C17 is 0.72, C18 is 0.73, C19 is -1.0. Outcome is -> |
| Assistant | 0 |
| User | C0 is 1.18, C1 is 1.27, C2 is 0.57, C3 is 0.62, C4 is -0.17, C5 is 0.7, C6 is 1.79, C7 is 1.16, C8 is -0.17, C9 is 1.35, C10 is 0.84, C11 is 1.1, C12 is -0.02, C13 is 0.17, C14 is 0.5, C15 is -0.91, C16 is -0.43, C17 is 1.11, C18 is 0.73, C19 is -1.0. Outcome is -> |
| Assistant | 0 |
| User | C0 is -0.67, C1 is -0.94, C2 is -0.6, C3 is 0.38, C4 is -0.78, C5 is -0.69, C6 is -0.37, C7 is -1.2, C8 is 1.12, C9 is -1.05, C10 is -0.93, C11 is -0.43, C12 is -0.16, C13 is 1.28, C14 is -1.94, C15 is -0.27, C16 is -0.14, C17 is -1.54, C18 is -1.92, C19 is 1.0. Outcome is -> |
| Assistant | 0 |
| User | C0 is -1.31, C1 is -0.49, C2 is 0.04, C3 is -1.15, C4 is -0.75, C5 is -0.98, C6 is 0.52, C7 is 1.11, C8 is 2.03, C9 is -1.17, C10 is -0.93, C11 is -0.71, C12 is -1.06, C13 is -1.3, C14 is -0.52, C15 is 2.61, C16 is -0.68, C17 is 0.32, C18 is 0.07, C19 is -1.0. Outcome is -> |
| Assistant | 0 |
| User | C0 is 0.53, C1 is -0.82, C2 is 1.71, C3 is -0.93, C4 is 1.5, C5 is 0.54, C6 is -0.29, C7 is 0.54, C8 is -0.55, C9 is 0.09, C10 is -0.93, C11 is -1.19, C12 is 1.93, C13 is 1.09, C14 is 0.14, C15 is -0.36, C16 is -0.59, C17 is 0.82, C18 is 0.73, C19 is 1.0. Outcome is -> |
| Assistant | 0 |
| User | C0 is -2.65, C1 is 0.2, C2 is -0.6, C3 is -0.23, C4 is -0.74, C5 is 0.15, C6 is 1.39, C7 is 0.95, C8 is 0.88, C9 is -1.67, C10 is -0.93, C11 is -0.81, C12 is 2.06, C13 is -0.47, C14 is 0.95, C15 is -0.75, C16 is -0.61, C17 is -0.76, C18 is 0.73, C19 is -1.0. Outcome is -> |

Table S6: Prompt used when stripping away all context information, for the same example as shown in Table S2-S5. C0 corresponds to Activated partial thromboplastin time, C1 to Creatinine, C2 to Blood sodium, C3 to NT-proBNP, C4 to Procalcitonin, C5 to C-reactive protein, C6 to White blood cells, C7 to Hematocrit, C8 to Platelets, C9 to Partial pressure of oxygen (PaO2), C10 to Fraction of inspired oxygen (FiO2), C11 to Lactic acid, C12 to Systolic blood pressure, C13 to Diastolic blood pressure, C14 to Age, C15 to Albumin, C16 to Total bilirubin, C17 to Prothrombin time, C18 to Glasgow Coma Scale score, and C19 to Gender.

Example prompts

Gastric cancer dataset

| Role | Message |
| --- | --- |
| System | You will be given characteristics of a gastric cancer patient.  Classify the Lymph Node Metastasis (LNM) status into one of the following categories: negative, or positive.  Return only the name of the category, and nothing else.  MAKE SURE your output is one of the two categories stated. |
| User | Age: 57, Tumor location: lower, Tumor stage: T3, Histology: diffuse, Tumor grade: G3, Tumor size: 7 mm, Gender: female, Tumor: not SRCC. LNM status is -> |
| Assistant | negative |
| User | Age: 59, Tumor location: upper, Tumor stage: T3, Histology: mixed/other, Tumor grade: G3, Tumor size: 60 mm, Gender: male, Tumor: not SRCC. LNM status is -> |
| Assistant | positive |
| User | Age: 56, Tumor location: upper, Tumor stage: T2, Histology: mixed/other, Tumor grade: G3, Tumor size: 65 mm, Gender: male, Tumor: not SRCC. LNM status is -> |
| Assistant | positive |
| User | Age: 80, Tumor location: lower, Tumor stage: T3, Histology: intestinal, Tumor grade: G3, Tumor size: 55 mm, Gender: female, Tumor: not SRCC. LNM status is -> |
| Assistant | positive |
| User | Age: 79, Tumor location: upper, Tumor stage: T3, Histology: intestinal, Tumor grade: G2, Tumor size: 10 mm, Gender: male, Tumor: not SRCC. LNM status is -> |
| Assistant | negative |
| User | Age: 74, Tumor location: upper, Tumor stage: T3, Histology: mixed/other, Tumor grade: G3, Tumor size: 70 mm, Gender: male, Tumor: not SRCC. LNM status is -> |
| Assistant | positive |
| User | Age: 67, Tumor location: lower, Tumor stage: T3, Histology: mixed/other, Tumor grade: G3, Tumor size: 45 mm, Gender: male, Tumor: not SRCC. LNM status is -> |
| Assistant | positive |
| User | Age: 73, Tumor location: upper, Tumor stage: T2, Histology: diffuse, Tumor grade: G3, Tumor size: 65 mm, Gender: male, Tumor: not SRCC. LNM status is -> |
| Assistant | negative |
| User | Age: 56, Tumor location: upper, Tumor stage: T3, Histology: intestinal, Tumor grade: G3, Tumor size: 50 mm, Gender: male, Tumor: not SRCC. LNM status is -> |
| Assistant | negative |
| User | Age: 69, Tumor location: lower, Tumor stage: T3, Histology: intestinal, Tumor grade: G3, Tumor size: 28 mm, Gender: male, Tumor: not SRCC. LNM status is -> |
| Assistant | positive |
| User | Age: 67, Tumor location: upper, Tumor stage: T1b, Histology: diffuse, Tumor grade: G3, Tumor size: 35 mm, Gender: female, Tumor: not SRCC. LNM status is -> |

Table S7: Example of a prompt submitted to the LLM for the gastric cancer dataset, for *TR* = 10. It consists in a series of messages: first, the system message giving the general context; then, a series of alternating user and assistant messages corresponding to the train patients; and finally, a user message corresponding to the test patient, for which the LLM is invited to make a prediction.

| Role | Message |
| --- | --- |
| System | You will be given characteristics of a gastric cancer patient.  Classify the Lymph Node Metastasis (LNM) status into one of the following categories: negative, or positive.  Return only the name of the category, and nothing else.  MAKE SURE your output is one of the two categories stated. |
| User | Age: 57, Tumor location: lower, Tumor size: 7, Tumor stage: T3, Histology: diffuse, Tumor grade: G3, Gender: female, Tumor: not SRCC. LNM status is -> |
| Assistant | negative |
| User | Age: 59, Tumor location: upper, Tumor size: 60, Tumor stage: T3, Histology: mixed/other, Tumor grade: G3, Gender: male, Tumor: not SRCC. LNM status is -> |
| Assistant | positive |
| User | Age: 56, Tumor location: upper, Tumor size: 65, Tumor stage: T2, Histology: mixed/other, Tumor grade: G3, Gender: male, Tumor: not SRCC. LNM status is -> |
| Assistant | positive |
| User | Age: 80, Tumor location: lower, Tumor size: 55, Tumor stage: T3, Histology: intestinal, Tumor grade: G3, Gender: female, Tumor: not SRCC. LNM status is -> |
| Assistant | positive |
| User | Age: 79, Tumor location: upper, Tumor size: 10, Tumor stage: T3, Histology: intestinal, Tumor grade: G2, Gender: male, Tumor: not SRCC. LNM status is -> |
| Assistant | negative |
| User | Age: 74, Tumor location: upper, Tumor size: 70, Tumor stage: T3, Histology: mixed/other, Tumor grade: G3, Gender: male, Tumor: not SRCC. LNM status is -> |
| Assistant | positive |
| User | Age: 67, Tumor location: lower, Tumor size: 45, Tumor stage: T3, Histology: mixed/other, Tumor grade: G3, Gender: male, Tumor: not SRCC. LNM status is -> |
| Assistant | positive |
| User | Age: 73, Tumor location: upper, Tumor size: 65, Tumor stage: T2, Histology: diffuse, Tumor grade: G3, Gender: male, Tumor: not SRCC. LNM status is -> |
| Assistant | negative |
| User | Age: 56, Tumor location: upper, Tumor size: 50, Tumor stage: T3, Histology: intestinal, Tumor grade: G3, Gender: male, Tumor: not SRCC. LNM status is -> |
| Assistant | negative |
| User | Age: 69, Tumor location: lower, Tumor size: 28, Tumor stage: T3, Histology: intestinal, Tumor grade: G3, Gender: male, Tumor: not SRCC. LNM status is -> |
| Assistant | positive |
| User | Age: 67, Tumor location: upper, Tumor size: 35, Tumor stage: T1b, Histology: diffuse, Tumor grade: G3, Gender: female, Tumor: not SRCC. LNM status is -> |

Table S8: Example of a prompt submitted to the LLM, without units, for the same example as shown in Table S7.

| Role | Message |
| --- | --- |
| System | You will be given characteristics of a gastric cancer patient.  Classify the Lymph Node Metastasis (LNM) status into one of the following categories: negative, or positive.  Return only the name of the category, and nothing else.  MAKE SURE your output is one of the two categories stated. |
| User | Gender: 57, Tumor size: lower, Tumor stage: 7, Histology: T3, Tumor grade: diffuse, Tumor: G3, Tumor location: female, Age: not SRCC. LNM status is ->'}, {'role': 'assistant', 'content': 'negative'}, {'role': 'user', 'content': 'Gender: 59, Tumor size: upper, Tumor stage: 60, Histology: T3, Tumor grade: mixed/other, Tumor: G3, Tumor location: male, Age: not SRCC. LNM status is -> |
| Assistant | negative |
| User | Gender: 59, Tumor size: upper, Tumor stage: 60, Histology: T3, Tumor grade: mixed/other, Tumor: G3, Tumor location: male, Age: not SRCC. LNM status is -> |
| Assistant | positive |
| User | Gender: 56, Tumor size: upper, Tumor stage: 65, Histology: T2, Tumor grade: mixed/other, Tumor: G3, Tumor location: male, Age: not SRCC. LNM status is -> |
| Assistant | positive |
| User | Gender: 80, Tumor size: lower, Tumor stage: 55, Histology: T3, Tumor grade: intestinal, Tumor: G3, Tumor location: female, Age: not SRCC. LNM status is -> |
| Assistant | positive |
| User | Gender: 79, Tumor size: upper, Tumor stage: 10, Histology: T3, Tumor grade: intestinal, Tumor: G2, Tumor location: male, Age: not SRCC. LNM status is -> |
| Assistant | negative |
| User | Gender: 74, Tumor size: upper, Tumor stage: 70, Histology: T3, Tumor grade: mixed/other, Tumor: G3, Tumor location: male, Age: not SRCC. LNM status is -> |
| Assistant | positive |
| User | Gender: 67, Tumor size: lower, Tumor stage: 45, Histology: T3, Tumor grade: mixed/other, Tumor: G3, Tumor location: male, Age: not SRCC. LNM status is -> |
| Assistant | positive |
| User | Gender: 73, Tumor size: upper, Tumor stage: 65, Histology: T2, Tumor grade: diffuse, Tumor: G3, Tumor location: male, Age: not SRCC. LNM status is -> |
| Assistant | negative |
| User | Gender: 56, Tumor size: upper, Tumor stage: 50, Histology: T3, Tumor grade: intestinal, Tumor: G3, Tumor location: male, Age: not SRCC. LNM status is -> |
| Assistant | negative |
| User | Gender: 69, Tumor size: lower, Tumor stage: 28, Histology: T3, Tumor grade: intestinal, Tumor: G3, Tumor location: male, Age: not SRCC. LNM status is -> |
| Assistant | positive |
| User | Gender: 67, Tumor size: upper, Tumor stage: 35, Histology: T1b, Tumor grade: diffuse, Tumor: G3, Tumor location: female, Age: not SRCC. LNM status is -> |

Table S9: Example of a prompt submitted to the LLM, with permutated column names, for the same example as shown in Tables S7-S8.

| Role | Message |
| --- | --- |
| System | You will be given characteristics of a gastric cancer patient.  Classify the Lymph Node Metastasis (LNM) status into one of the following categories: negative, or positive.  Return only the name of the category, and nothing else.  MAKE SURE your output is one of the two categories stated. |
| User | C0: 57, C1: lower, C2: 7, C3: T3, C4: diffuse, C5: G3, C6: female, C7: not SRCC. LNM status is -> |
| Assistant | negative |
| User | C0: 59, C1: upper, C2: 60, C3: T3, C4: mixed/other, C5: G3, C6: male, C7: not SRCC. LNM status is -> |
| Assistant | positive |
| User | C0: 56, C1: upper, C2: 65, C3: T2, C4: mixed/other, C5: G3, C6: male, C7: not SRCC. LNM status is -> |
| Assistant | positive |
| User | C0: 80, C1: lower, C2: 55, C3: T3, C4: intestinal, C5: G3, C6: female, C7: not SRCC. LNM status is -> |
| Assistant | positive |
| User | C0: 79, C1: upper, C2: 10, C3: T3, C4: intestinal, C5: G2, C6: male, C7: not SRCC. LNM status is -> |
| Assistant | negative |
| User | C0: 74, C1: upper, C2: 70, C3: T3, C4: mixed/other, C5: G3, C6: male, C7: not SRCC. LNM status is -> |
| Assistant | positive |
| User | C0: 67, C1: lower, C2: 45, C3: T3, C4: mixed/other, C5: G3, C6: male, C7: not SRCC. LNM status is -> |
| Assistant | positive |
| User | C0: 73, C1: upper, C2: 65, C3: T2, C4: diffuse, C5: G3, C6: male, C7: not SRCC. LNM status is -> |
| Assistant | negative |
| User | C0: 56, C1: upper, C2: 50, C3: T3, C4: intestinal, C5: G3, C6: male, C7: not SRCC. LNM status is -> |
| Assistant | negative |
| User | C0: 69, C1: lower, C2: 28, C3: T3, C4: intestinal, C5: G3, C6: male, C7: not SRCC. LNM status is -> |
| Assistant | positive |
| User | C0: 67, C1: upper, C2: 35, C3: T1b, C4: diffuse, C5: G3, C6: female, C7: not SRCC. LNM status is -> |

Table S10: Example of a prompt submitted to the LLM, with neutral column names, for the same example as shown in Tables S7-S9.

| Role | Message |
| --- | --- |
| System | You will be given characteristics of a sample.  Classify the sample outcome into one of the following categories: 0, or 1.  Return only the name of the category, and nothing else.  MAKE SURE your output is one of the two categories stated. |
| User | C0 is -1.11, C1 is -2.0, C2 is -1.77, C3 is 0.5, C4 is 0.0, C5 is 0.33, C6 is 2.0, C7 is -0.82, C8 is -0.82, C9 is 1.22, C10 is 0.0, C11 is -1.22. Outcome is -> |
| Assistant | 0 |
| User | C0 is -0.89, C1 is 0.5, C2 is 0.67, C3 is 0.5, C4 is 0.0, C5 is 0.33, C6 is -0.5, C7 is -0.82, C8 is 1.22, C9 is -0.82, C10 is 0.0, C11 is 0.82. Outcome is -> |
| Assistant | 1 |
| User | C0 is -1.22, C1 is 0.5, C2 is 0.9, C3 is -2.0, C4 is 0.0, C5 is 0.33, C6 is -0.5, C7 is -0.82, C8 is 1.22, C9 is -0.82, C10 is 0.0, C11 is 0.82. Outcome is -> |
| Assistant | 1 |
| User | C0 is 1.45, C1 is -2.0, C2 is 0.44, C3 is 0.5, C4 is 0.0, C5 is 0.33, C6 is -0.5, C7 is 1.22, C8 is -0.82, C9 is 1.22, C10 is 0.0, C11 is -1.22. Outcome is -> |
| Assistant | 1 |
| User | C0 is 1.33, C1 is 0.5, C2 is -1.63, C3 is 0.5, C4 is 0.0, C5 is -3.0, C6 is -0.5, C7 is 1.22, C8 is -0.82, C9 is -0.82, C10 is 0.0, C11 is 0.82. Outcome is -> |
| Assistant | 0 |
| User | C0 is 0.78, C1 is 0.5, C2 is 1.13, C3 is 0.5, C4 is 0.0, C5 is 0.33, C6 is -0.5, C7 is -0.82, C8 is 1.22, C9 is -0.82, C10 is 0.0, C11 is 0.82. Outcome is -> |
| Assistant | 1 |
| User | C0 is 0.0, C1 is 0.5, C2 is -0.02, C3 is 0.5, C4 is 0.0, C5 is 0.33, C6 is -0.5, C7 is -0.82, C8 is 1.22, C9 is 1.22, C10 is 0.0, C11 is -1.22. Outcome is -> |
| Assistant | 1 |
| User | C0 is 0.67, C1 is 0.5, C2 is 0.9, C3 is -2.0, C4 is 0.0, C5 is 0.33, C6 is 2.0, C7 is -0.82, C8 is -0.82, C9 is -0.82, C10 is 0.0, C11 is 0.82. Outcome is -> |
| Assistant | 0 |
| User | C0 is -1.22, C1 is 0.5, C2 is 0.21, C3 is 0.5, C4 is 0.0, C5 is 0.33, C6 is -0.5, C7 is 1.22, C8 is -0.82, C9 is -0.82, C10 is 0.0, C11 is 0.82. Outcome is -> |
| Assistant | 0 |
| User | C0 is 0.22, C1 is 0.5, C2 is -0.8, C3 is 0.5, C4 is 0.0, C5 is 0.33, C6 is -0.5, C7 is 1.22, C8 is -0.82, C9 is 1.22, C10 is 0.0, C11 is -1.22. Outcome is -> |
| Assistant | 1 |
| User | C0 is 0.0, C1 is -2.0, C2 is -0.48, C3 is -4.5, C4 is 0.0, C5 is 0.33, C6 is 2.0, C7 is -0.82, C8 is -0.82, C9 is -0.82, C10 is 0.0, C11 is 0.82. Outcome is -> |

Table S11: Prompt used when stripping away all context information, for the same example as shown in Tables S7-10. C0 corresponds to age, C1 to gender, C2 to tumor size, C3 to tumor stage, C4 to SRCC status, C5 to tumor grade, C6 to histology – diffuse type, C7 to histology – intestinal type, C8 to histology – Mixed/Other, C9 to tumor location – lower, C10 to tumor location – middle, C11 to tumor location – upper.

Example prompts

Acute leukemia dataset

| Role | Message |
| --- | --- |
| System | You will be given characteristics of an acute leukemia patient.  Classify the Leukemia into one of the following categories: AML, or APL.  Return only the name of the category, and nothing else.\n MAKE SURE your output is one of the two categories stated. |
| User | Age: 79, white blood cells: 9.55 k/µL, mean corpuscular volume: 85.5 fL, mean corpuscular hemoglobin concentration: 34.7 g/dL, absolute neutrophil count: 3.92 k/µL, lymphocytes: 3.44 k/µL, monocytes: 0.76 k/µL, platelets: 29.0 k/µL, fibrinogen: 1.4 g/L, lactate dehydrogenase: 12165.0 U/L. Leukemia is -> |
| Assistant | AML |
| User | Age: 52, white blood cells: 1.1 k/µL, mean corpuscular volume: 92.2 fL, mean corpuscular hemoglobin concentration: 35.6 g/dL, absolute neutrophil count: 0.1 k/µL, lymphocytes: 0.8 k/µL, monocytes: 0.2 k/µL, platelets: 142.0 k/µL, fibrinogen: 2.9 g/L, lactate dehydrogenase: 167.0 U/L. Leukemia is -> |
| Assistant | AML |
| User | Age: 78, white blood cells: 102.5 k/µL, mean corpuscular volume: 100.0 fL, mean corpuscular hemoglobin concentration: 32.4 g/dL, absolute neutrophil count: 5.2 k/µL, lymphocytes: 3.3 k/µL, monocytes: 2.4 k/µL, platelets: 6.0 k/µL, fibrinogen: 5.9 g/L, lactate dehydrogenase: 638.0 U/L. Leukemia is -> |
| Assistant | AML |
| User | Age: 71, white blood cells: 2.44 k/µL, mean corpuscular volume: 94.1 fL, mean corpuscular hemoglobin concentration: 32.0 g/dL, absolute neutrophil count: 0.66 k/µL, lymphocytes: 1.49 k/µL, monocytes: 0.12 k/µL, platelets: 29.0 k/µL, fibrinogen: 3.7 g/L, lactate dehydrogenase: 333.0 U/L. Leukemia is -> |
| Assistant | AML |
| User | Age: 64, white blood cells: 321.6 k/µL, mean corpuscular volume: 97.0 fL, mean corpuscular hemoglobin concentration: 33.8 g/dL, absolute neutrophil count: 40.2 k/µL, lymphocytes: 8.0 k/µL, monocytes: 6.4 k/µL, platelets: 59.0 k/µL, fibrinogen: 4.3 g/L, lactate dehydrogenase: 763.0 U/L. Leukemia is -> |
| Assistant | AML |
| User | Age: 9, white blood cells: 15.6 k/µL, mean corpuscular volume: 82.5 fL, mean corpuscular hemoglobin concentration: 33.7 g/dL, absolute neutrophil count: 1.1 k/µL, lymphocytes: 4.6 k/µL, monocytes: 0.2 k/µL, platelets: 124.0 k/µL, fibrinogen: 2.9 g/L, lactate dehydrogenase: 801.0 U/L. Leukemia is -> |
| Assistant | AML |
| User | Age: 53, white blood cells: 21.0 k/µL, mean corpuscular volume: 97.9 fL, mean corpuscular hemoglobin concentration: 31.9 g/dL, absolute neutrophil count: 6.41 k/µL, lymphocytes: 3.57 k/µL, monocytes: 10.08 k/µL, platelets: 162.0 k/µL, fibrinogen: 4.1 g/L, lactate dehydrogenase: 176.3 U/L. Leukemia is -> |
| Assistant | AML |
| User | Age: 53, white blood cells: 2.5 k/µL, mean corpuscular volume: 107.1 fL, mean corpuscular hemoglobin concentration: 35.6 g/dL, absolute neutrophil count: 1.29 k/µL, lymphocytes: 1.1 k/µL, monocytes: 0.07 k/µL, platelets: 83.0 k/µL, fibrinogen: 3.0 g/L, lactate dehydrogenase: 181.0 U/L. Leukemia is -> |
| Assistant | AML |
| User | Age: 60, white blood cells: 10.87 k/µL, mean corpuscular volume: 80.2 fL, mean corpuscular hemoglobin concentration: 36.2 g/dL, absolute neutrophil count: 1.96 k/µL, lymphocytes: 2.28 k/µL, monocytes: 0.22 k/µL, platelets: 13.0 k/µL, fibrinogen: 1.9 g/L, lactate dehydrogenase: 511.0 U/L. Leukemia is -> |
| Assistant | APL |
| User | Age: 12, white blood cells: 3.2 k/µL, mean corpuscular volume: 83.7 fL, mean corpuscular hemoglobin concentration: 34.5 g/dL, absolute neutrophil count: 0.9 k/µL, lymphocytes: 1.4 k/µL, monocytes: 0.1 k/µL, platelets: 12.0 k/µL, fibrinogen: 0.7 g/L, lactate dehydrogenase: 260.0 U/L. Leukemia is -> |
| Assistant | APL |
| User | Age: 51, white blood cells: 1.37 k/µL, mean corpuscular volume: 93.2 fL, mean corpuscular hemoglobin concentration: 34.7 g/dL, absolute neutrophil count: 0.26 k/µL, lymphocytes: 0.85 k/µL, monocytes: 0.01 k/µL, platelets: 113.0 k/µL, fibrinogen: 1.2 g/L, lactate dehydrogenase: 207.9 U/L. Leukemia is -> |

Table S12: Example of a prompt submitted to the LLM for the acute leukemia dataset, for *TR* = 10. It consists in a series of messages: first, the system message giving the general context; then, a series of alternating user and assistant messages corresponding to the train patients; and finally, a user message corresponding to the test patient, for which the LLM is invited to make a prediction.

| Role | Message |
| --- | --- |
| System | You will be given characteristics of an acute leukemia patient.  Classify the Leukemia into one of the following categories: AML, or APL.  Return only the name of the category, and nothing else.\n MAKE SURE your output is one of the two categories stated. |
| User | Age: 79, white blood cells: 9.55, mean corpuscular volume: 85.5, mean corpuscular hemoglobin concentration: 34.7, absolute neutrophil count: 3.92, lymphocytes: 3.44, monocytes: 0.76, platelets: 29.0, fibrinogen: 1.4, lactate dehydrogenase: 12165.0. Leukemia is -> |
| Assistant | AML |
| User | Age: 52, white blood cells: 1.1, mean corpuscular volume: 92.2, mean corpuscular hemoglobin concentration: 35.6, absolute neutrophil count: 0.1, lymphocytes: 0.8, monocytes: 0.2, platelets: 142.0, fibrinogen: 2.9, lactate dehydrogenase: 167.0. Leukemia is -> |
| Assistant | AML |
| User | Age: 78, white blood cells: 102.5, mean corpuscular volume: 100.0, mean corpuscular hemoglobin concentration: 32.4, absolute neutrophil count: 5.2, lymphocytes: 3.3, monocytes: 2.4, platelets: 6.0, fibrinogen: 5.9, lactate dehydrogenase: 638.0. Leukemia is -> |
| Assistant | AML |
| User | Age: 71, white blood cells: 2.44, mean corpuscular volume: 94.1, mean corpuscular hemoglobin concentration: 32.0, absolute neutrophil count: 0.66, lymphocytes: 1.49, monocytes: 0.12, platelets: 29.0, fibrinogen: 3.7, lactate dehydrogenase: 333.0. Leukemia is -> |
| Assistant | AML |
| User | Age: 64, white blood cells: 321.6, mean corpuscular volume: 97.0, mean corpuscular hemoglobin concentration: 33.8, absolute neutrophil count: 40.2, lymphocytes: 8.0, monocytes: 6.4, platelets: 59.0, fibrinogen: 4.3, lactate dehydrogenase: 763.0. Leukemia is -> |
| Assistant | AML |
| User | Age: 9, white blood cells: 15.6, mean corpuscular volume: 82.5, mean corpuscular hemoglobin concentration: 33.7, absolute neutrophil count: 1.1, lymphocytes: 4.6, monocytes: 0.2, platelets: 124.0, fibrinogen: 2.9, lactate dehydrogenase: 801.0. Leukemia is -> |
| Assistant | AML |
| User | Age: 53, white blood cells: 21.0, mean corpuscular volume: 97.9, mean corpuscular hemoglobin concentration: 31.9, absolute neutrophil count: 6.41, lymphocytes: 3.57, monocytes: 10.08, platelets: 162.0, fibrinogen: 4.1, lactate dehydrogenase: 176.3. Leukemia is -> |
| Assistant | AML |
| User | Age: 53, white blood cells: 2.5, mean corpuscular volume: 107.1, mean corpuscular hemoglobin concentration: 35.6, absolute neutrophil count: 1.29, lymphocytes: 1.1, monocytes: 0.07, platelets: 83.0, fibrinogen: 3.0, lactate dehydrogenase: 181.0. Leukemia is -> |
| Assistant | AML |
| User | Age: 60, white blood cells: 10.87, mean corpuscular volume: 80.2, mean corpuscular hemoglobin concentration: 36.2, absolute neutrophil count: 1.96, lymphocytes: 2.28, monocytes: 0.22, platelets: 13.0, fibrinogen: 1.9, lactate dehydrogenase: 511.0. Leukemia is -> |
| Assistant | APL |
| User | Age: 12, white blood cells: 3.2, mean corpuscular volume: 83.7, mean corpuscular hemoglobin concentration: 34.5, absolute neutrophil count: 0.9, lymphocytes: 1.4, monocytes: 0.1, platelets: 12.0, fibrinogen: 0.7, lactate dehydrogenase: 260.0. Leukemia is -> |
| Assistant | APL |
| User | Age: 51, white blood cells: 1.37, mean corpuscular volume: 93.2, mean corpuscular hemoglobin concentration: 34.7, absolute neutrophil count: 0.26, lymphocytes: 0.85, monocytes: 0.01, platelets: 113.0, fibrinogen: 1.2, lactate dehydrogenase: 207.9. Leukemia is -> |

Table S13: Example of a prompt submitted to the LLM, without units, for the same example as shown in Table S12.

| Role | Message |
| --- | --- |
| System | You will be given characteristics of an acute leukemia patient.  Classify the Leukemia into one of the following categories: AML, or APL.  Return only the name of the category, and nothing else.\n MAKE SURE your output is one of the two categories stated. |
| User | White blood cells: 79, mean corpuscular volume: 9.55, mean corpuscular hemoglobin concentration: 85.5, absolute neutrophil count: 34.7, lymphocytes: 3.92, monocytes: 3.44, platelets: 0.76, fibrinogen: 29.0, lactate dehydrogenase: 1.4, age: 12165.0. Leukemia is -> |
| Assistant | AML |
| User | White blood cells: 52, mean corpuscular volume: 1.1, mean corpuscular hemoglobin concentration: 92.2, absolute neutrophil count: 35.6, lymphocytes: 0.1, monocytes: 0.8, platelets: 0.2, fibrinogen: 142.0, lactate dehydrogenase: 2.9, age: 167.0. Leukemia is -> |
| Assistant | AML |
| User | White blood cells: 78, mean corpuscular volume: 102.5, mean corpuscular hemoglobin concentration: 100.0, absolute neutrophil count: 32.4, lymphocytes: 5.2, monocytes: 3.3, platelets: 2.4, fibrinogen: 6.0, lactate dehydrogenase: 5.9, age: 638.0. Leukemia is -> |
| Assistant | AML |
| User | White blood cells: 71, mean corpuscular volume: 2.44, mean corpuscular hemoglobin concentration: 94.1, absolute neutrophil count: 32.0, lymphocytes: 0.66, monocytes: 1.49, platelets: 0.12, fibrinogen: 29.0, lactate dehydrogenase: 3.7, age: 333.0. Leukemia is -> |
| Assistant | AML |
| User | White blood cells: 64, mean corpuscular volume: 321.6, mean corpuscular hemoglobin concentration: 97.0, absolute neutrophil count: 33.8, lymphocytes: 40.2, monocytes: 8.0, platelets: 6.4, fibrinogen: 59.0, lactate dehydrogenase: 4.3, age: 763.0. Leukemia is -> |
| Assistant | AML |
| User | White blood cells: 9, mean corpuscular volume: 15.6, mean corpuscular hemoglobin concentration: 82.5, absolute neutrophil count: 33.7, lymphocytes: 1.1, monocytes: 4.6, platelets: 0.2, fibrinogen: 124.0, lactate dehydrogenase: 2.9, age: 801.0. Leukemia is -> |
| Assistant | AML |
| User | White blood cells: 53, mean corpuscular volume: 21.0, mean corpuscular hemoglobin concentration: 97.9, absolute neutrophil count: 31.9, lymphocytes: 6.41, monocytes: 3.57, platelets: 10.08, fibrinogen: 162.0, lactate dehydrogenase: 4.1, age: 176.3. Leukemia is -> |
| Assistant | AML |
| User | White blood cells: 53, mean corpuscular volume: 2.5, mean corpuscular hemoglobin concentration: 107.1, absolute neutrophil count: 35.6, lymphocytes: 1.29, monocytes: 1.1, platelets: 0.07, fibrinogen: 83.0, lactate dehydrogenase: 3.0, age: 181.0. Leukemia is -> |
| Assistant | AML |
| User | White blood cells: 60, mean corpuscular volume: 10.87, mean corpuscular hemoglobin concentration: 80.2, absolute neutrophil count: 36.2, lymphocytes: 1.96, monocytes: 2.28, platelets: 0.22, fibrinogen: 13.0, lactate dehydrogenase: 1.9, age: 511.0. Leukemia is -> |
| Assistant | APL |
| User | White blood cells: 12, mean corpuscular volume: 3.2, mean corpuscular hemoglobin concentration: 83.7, absolute neutrophil count: 34.5, lymphocytes: 0.9, monocytes: 1.4, platelets: 0.1, fibrinogen: 12.0, lactate dehydrogenase: 0.7, age: 260.0. Leukemia is -> |
| Assistant | APL |
| User | White blood cells: 51, mean corpuscular volume: 1.37, mean corpuscular hemoglobin concentration: 93.2, absolute neutrophil count: 34.7, lymphocytes: 0.26, monocytes: 0.85, platelets: 0.01, fibrinogen: 113.0, lactate dehydrogenase: 1.2, age: 207.9. Leukemia is -> |

Table S14: Example of a prompt submitted to the LLM, with permutated column names, for the same example as shown in Tables S12-S13.

| Role | Message |
| --- | --- |
| System | You will be given characteristics of an acute leukemia patient.  Classify the Leukemia into one of the following categories: AML, or APL.  Return only the name of the category, and nothing else.\n MAKE SURE your output is one of the two categories stated. |
| User | C0: 79, C1: 9.55, C2: 85.5, C3: 34.7, C4: 3.92, C5: 3.44, C6: 0.76, C7: 29.0, C8: 1.4, C9: 12165.0. Leukemia is -> |
| Assistant | AML |
| User | C0: 52, C1: 1.1, C2: 92.2, C3: 35.6, C4: 0.1, C5: 0.8, C6: 0.2, C7: 142.0, C8: 2.9, C9: 167.0. Leukemia is -> |
| Assistant | AML |
| User | C0: 78, C1: 102.5, C2: 100.0, C3: 32.4, C4: 5.2, C5: 3.3, C6: 2.4, C7: 6.0, C8: 5.9, C9: 638.0. Leukemia is -> |
| Assistant | AML |
| User | C0: 71, C1: 2.44, C2: 94.1, C3: 32.0, C4: 0.66, C5: 1.49, C6: 0.12, C7: 29.0, C8: 3.7, C9: 333.0. Leukemia is -> |
| Assistant | AML |
| User | C0: 64, C1: 321.6, C2: 97.0, C3: 33.8, C4: 40.2, C5: 8.0, C6: 6.4, C7: 59.0, C8: 4.3, C9: 763.0. Leukemia is -> |
| Assistant | AML |
| User | C0: 9, C1: 15.6, C2: 82.5, C3: 33.7, C4: 1.1, C5: 4.6, C6: 0.2, C7: 124.0, C8: 2.9, C9: 801.0. Leukemia is -> |
| Assistant | AML |
| User | C0: 53, C1: 21.0, C2: 97.9, C3: 31.9, C4: 6.41, C5: 3.57, C6: 10.08, C7: 162.0, C8: 4.1, C9: 176.3. Leukemia is -> |
| Assistant | AML |
| User | C0: 53, C1: 2.5, C2: 107.1, C3: 35.6, C4: 1.29, C5: 1.1, C6: 0.07, C7: 83.0, C8: 3.0, C9: 181.0. Leukemia is -> |
| Assistant | AML |
| User | C0: 60, C1: 10.87, C2: 80.2, C3: 36.2, C4: 1.96, C5: 2.28, C6: 0.22, C7: 13.0, C8: 1.9, C9: 511.0. Leukemia is -> |
| Assistant | APL |
| User | C0: 12, C1: 3.2, C2: 83.7, C3: 34.5, C4: 0.9, C5: 1.4, C6: 0.1, C7: 12.0, C8: 0.7, C9: 260.0. Leukemia is -> |
| Assistant | APL |
| User | C0: 51, C1: 1.37, C2: 93.2, C3: 34.7, C4: 0.26, C5: 0.85, C6: 0.01, C7: 113.0, C8: 1.2, C9: 207.9. Leukemia is -> |

Table S15: Example of a prompt submitted to the LLM, with neutral column names, for the same example as shown in Tables S12-S14.

| Role | Message |
| --- | --- |
| System | You will be given characteristics of a sample.  Classify the sample outcome into one of the following categories: 0, or 1.  Return only the name of the category, and nothing else.  MAKE SURE your output is one of the two categories stated. |
| User | C0 is 1.11, C1 is -0.41, C2 is -0.78, C3 is 0.45, C4 is -0.2, C5 is 0.22, C6 is -0.4, C7 is -0.67, C8 is -1.16, C9 is 2.99. Outcome is -> |
| Assistant | 0 |
| User | C0 is -0.05, C1 is -0.5, C2 is 0.02, C3 is 1.06, C4 is -0.53, C5 is -1.07, C6 is -0.57, C7 is 1.37, C8 is -0.12, C9 is -0.41. Outcome is -> |
| Assistant | 0 |
| User | C0 is 1.07, C1 is 0.56, C2 is 0.96, C3 is -1.11, C4 is -0.08, C5 is 0.15, C6 is 0.11, C7 is -1.08, C8 is 1.95, C9 is -0.27. Outcome is -> |
| Assistant | 0 |
| User | C0 is 0.77, C1 is -0.49, C2 is 0.25, C3 is -1.38, C4 is -0.48, C5 is -0.74, C6 is -0.59, C7 is -0.67, C8 is 0.43, C9 is -0.36. Outcome is -> |
| Assistant | 0 |
| User | C0 is 0.47, C1 is 2.86, C2 is 0.6, C3 is -0.16, C4 is 2.95, C5 is 2.44, C6 is 1.33, C7 is -0.12, C8 is 0.85, C9 is -0.24. Outcome is -> |
| Assistant | 0 |
| User | C0 is -1.9, C1 is -0.35, C2 is -1.14, C3 is -0.23, C4 is -0.44, C5 is 0.78, C6 is -0.57, C7 is 1.05, C8 is -0.12, C9 is -0.23. Outcome is -> |
| Assistant | 0 |
| User | C0 is -0.0, C1 is -0.29, C2 is 0.7, C3 is -1.45, C4 is 0.02, C5 is 0.28, C6 is 2.46, C7 is 1.73, C8 is 0.71, C9 is -0.4. Outcome is -> |
| Assistant | 0 |
| User | C0 is -0.0, C1 is -0.49, C2 is 1.81, C3 is 1.06, C4 is -0.42, C5 is -0.93, C6 is -0.61, C7 is 0.31, C8 is -0.06, C9 is -0.4. Outcome is -> |
| Assistant | 0 |
| User | C0 is 0.3, C1 is -0.4, C2 is -1.42, C3 is 1.46, C4 is -0.37, C5 is -0.35, C6 is -0.56, C7 is -0.95, C8 is -0.82, C9 is -0.31. Outcome is -> |
| Assistant | 1 |
| User | C0 is -1.77, C1 is -0.48, C2 is -1.0, C3 is 0.31, C4 is -0.46, C5 is -0.78, C6 is -0.6, C7 is -0.97, C8 is -1.65, C9 is -0.38. Outcome is -> |
| Assistant | 1 |
| User | C0 is -0.09, C1 is -0.5, C2 is 0.14, C3 is 0.45, C4 is -0.51, C5 is -1.05, C6 is -0.63, C7 is 0.85, C8 is -1.3, C9 is -0.39. Outcome is -> |

Table S16: Prompt used when stripping away all context information, for the same example as shown in Tables S11-14. C0 corresponds to age, C1 to white blood cells, C2 to mean corpuscular volume, C3 to mean corpuscular hemoglobin concentration, C4 to absolute neutrophil count, C5 to lymphocytes, C6 to monocytes, C7 to platelets, C8 to fibrinogen – Mixed/Other, and C9 to lactate dehydrogenase.

ROC curves


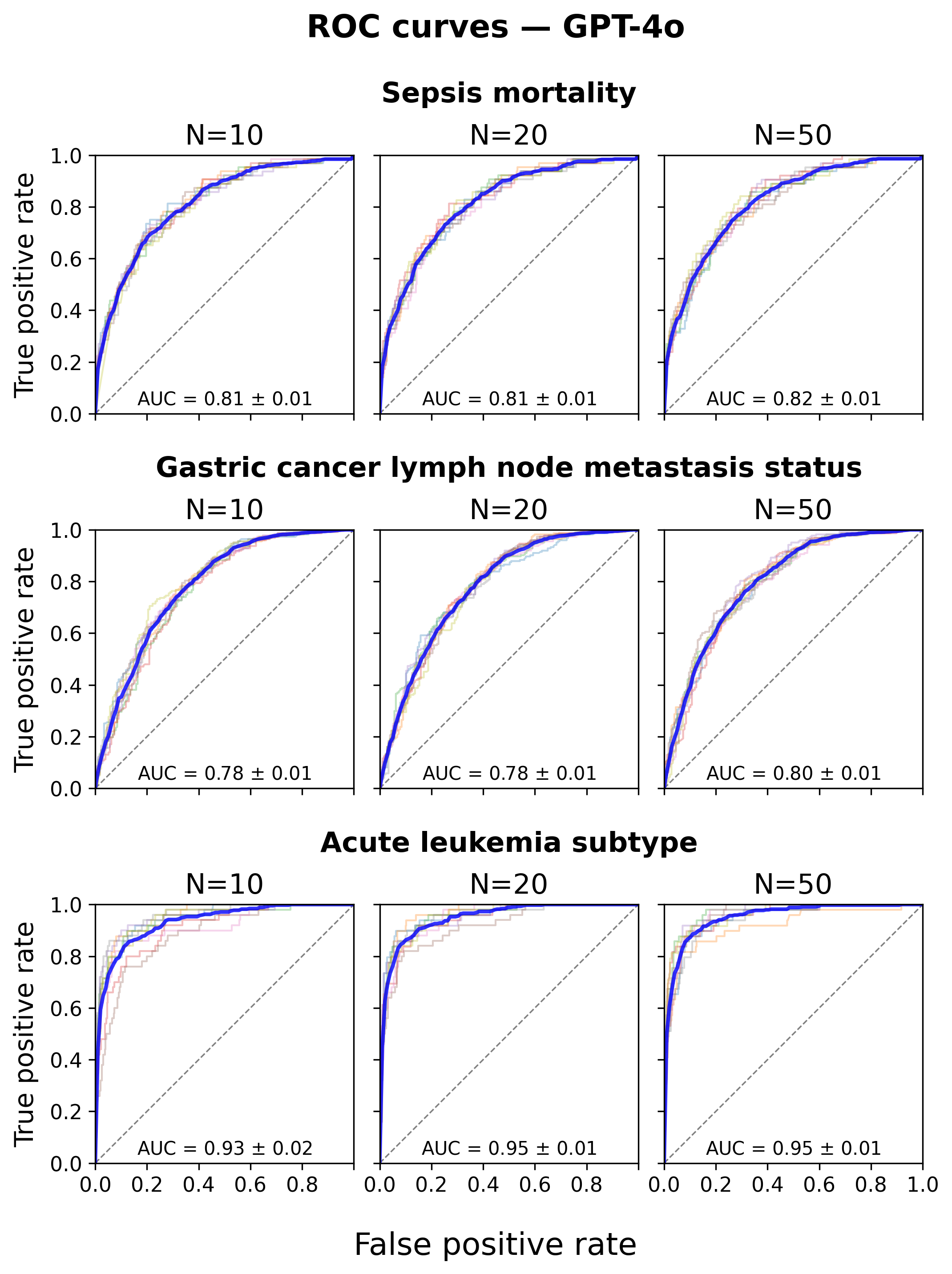


Figure S1: ROC curves for GPT-4o for the sepsis (top), gastric cancer (middle), and leukemia (bottom) datasets, and for various train sizes, TR = 10 (left), TR = 20 (center), and TR = 50 (right). The light-colored curves correspond to individual folds, and the blue curve is the mean curve across folds.


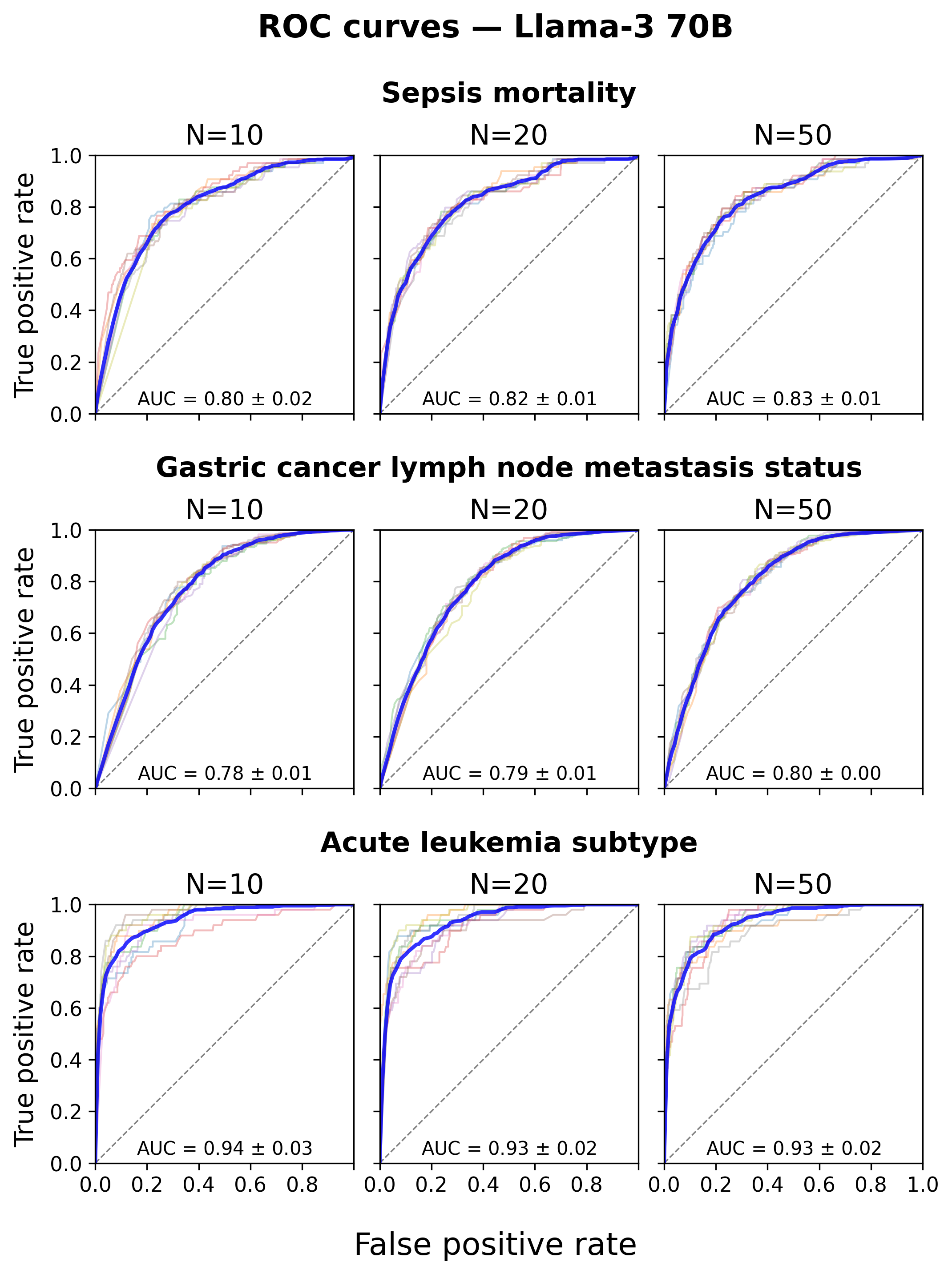


Figure S2: ROC curves for Llama 3 70B for the sepsis (top), gastric cancer (middle), and leukemia (bottom) datasets, and for various train sizes, TR = 10 (left), TR = 20 (center), and TR = 50 (right). The light-colored curves correspond to individual folds, and the blue curve is the mean curve across folds.


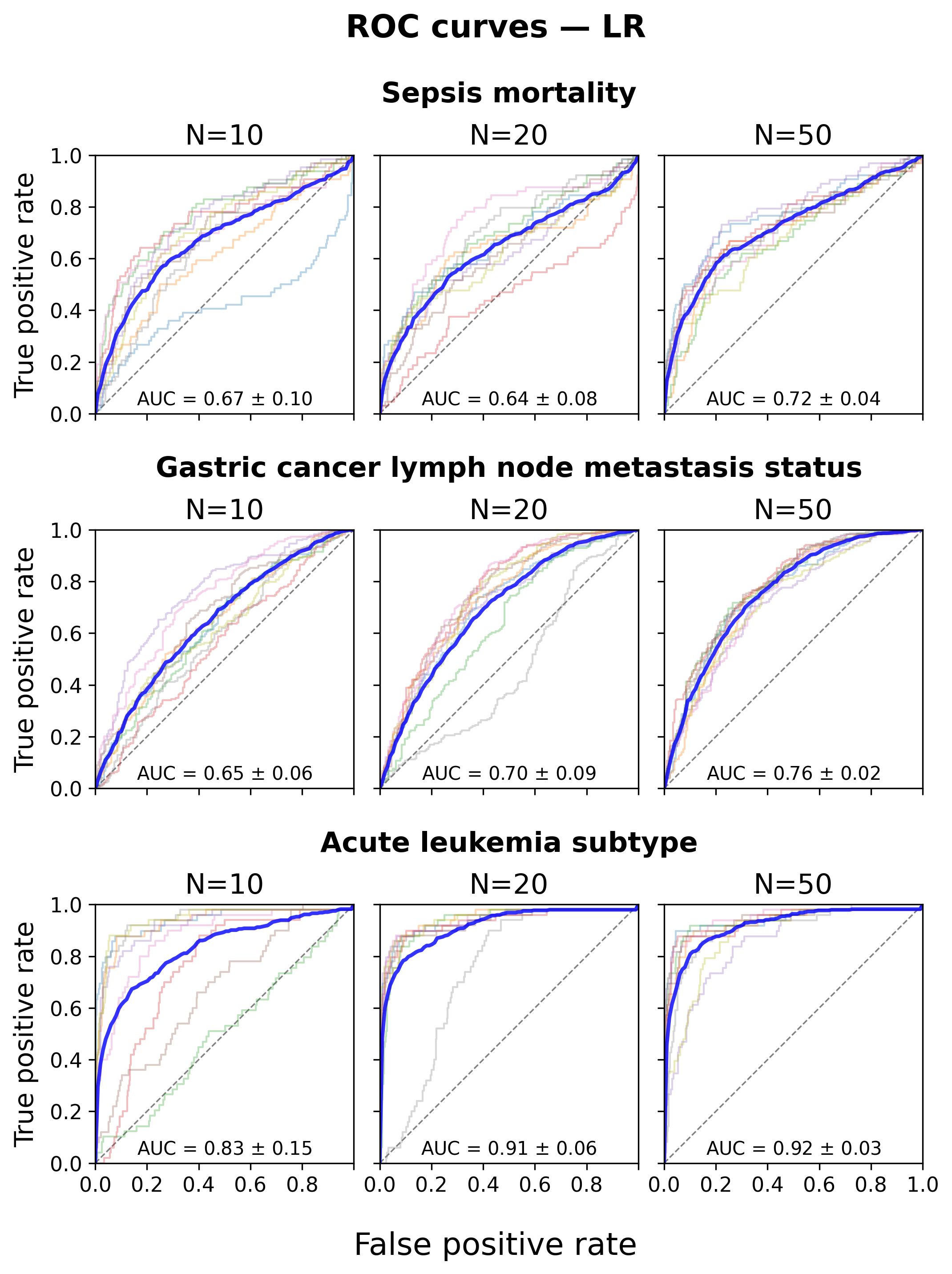


Figure S3: ROC curves for Logistic Regression (LR) for the sepsis (top), gastric cancer (middle), and leukemia (bottom) datasets, and for various train sizes, TR = 10 (left), TR = 20 (center), and TR = 50 (right). The light-colored curves correspond to individual folds, and the blue curve is the mean curve across folds.


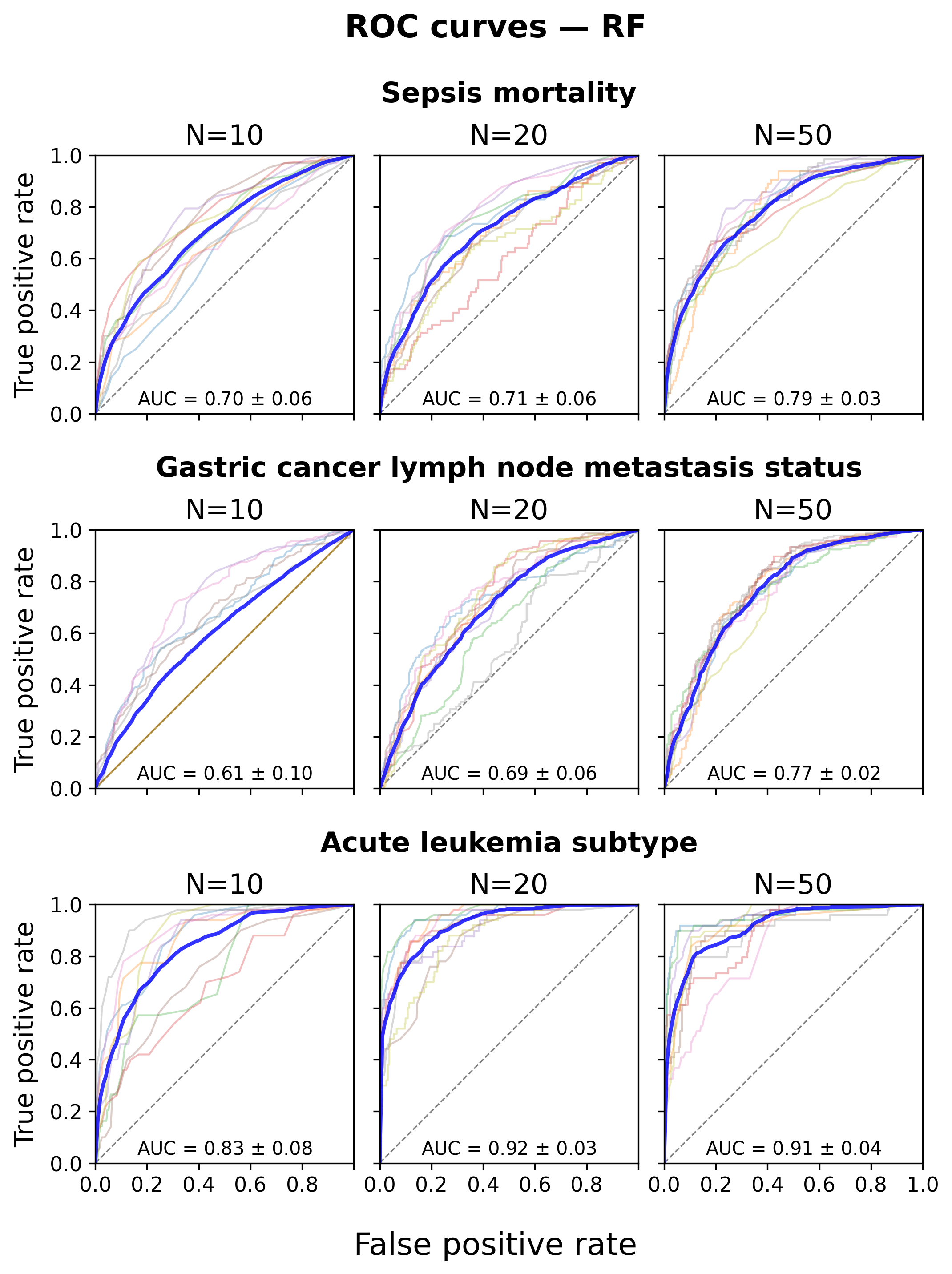


Figure S4: ROC curves for Random Forest (RF) for the sepsis (top), gastric cancer (middle), and leukemia (bottom) datasets, and for various train sizes, TR = 10 (left), TR = 20 (center), and TR = 50 (right). The light-colored curves correspond to individual folds, and the blue curve is the mean curve across folds.


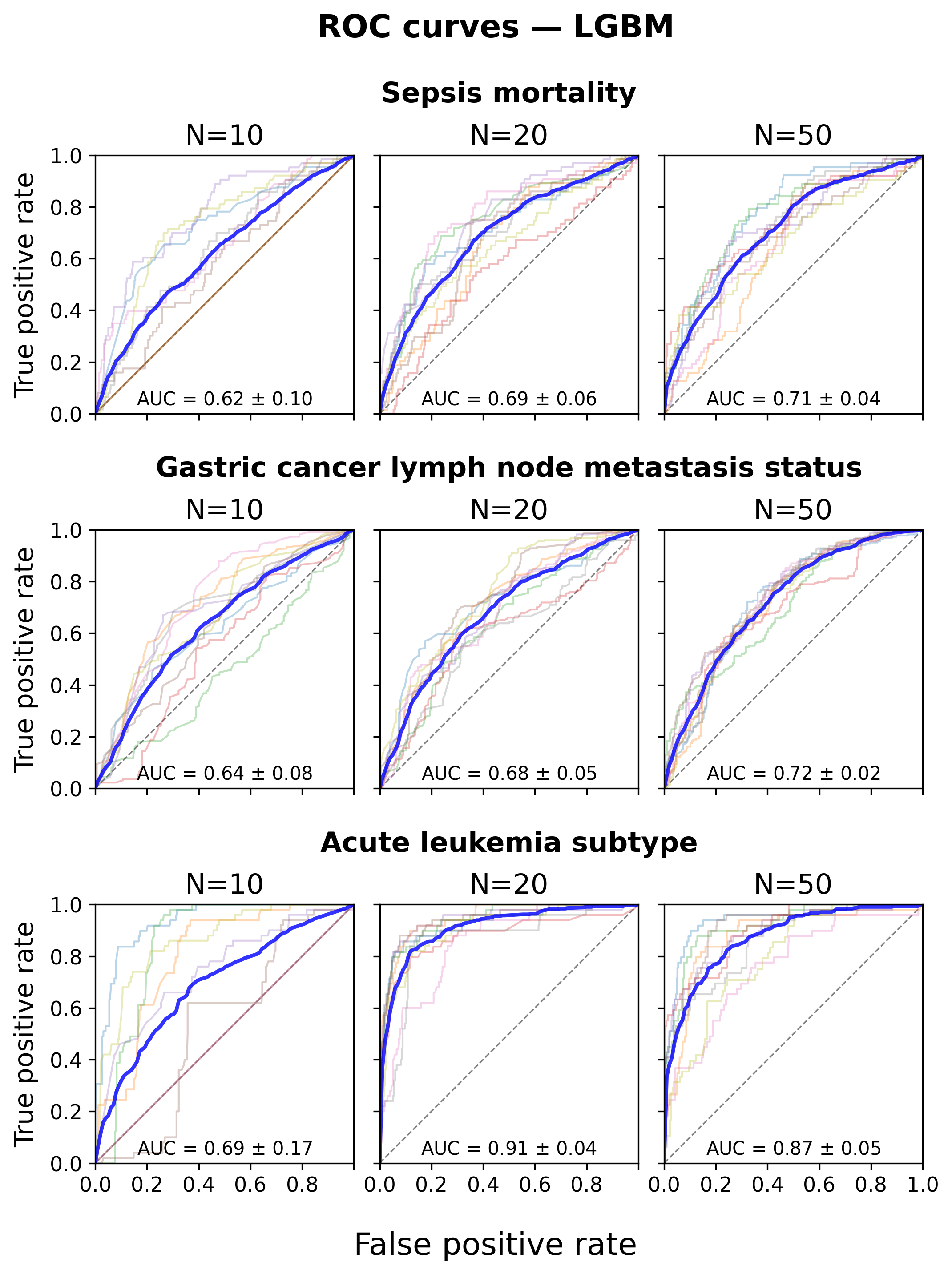


Figure S5: ROC curves for LGBM for the sepsis (top), gastric cancer (middle), and leukemia (bottom) datasets, and for various train sizes, TR = 10 (left), TR = 20 (center), and TR = 50 (right). The light-colored curves correspond to individual folds, and the blue curve is the mean curve across folds.


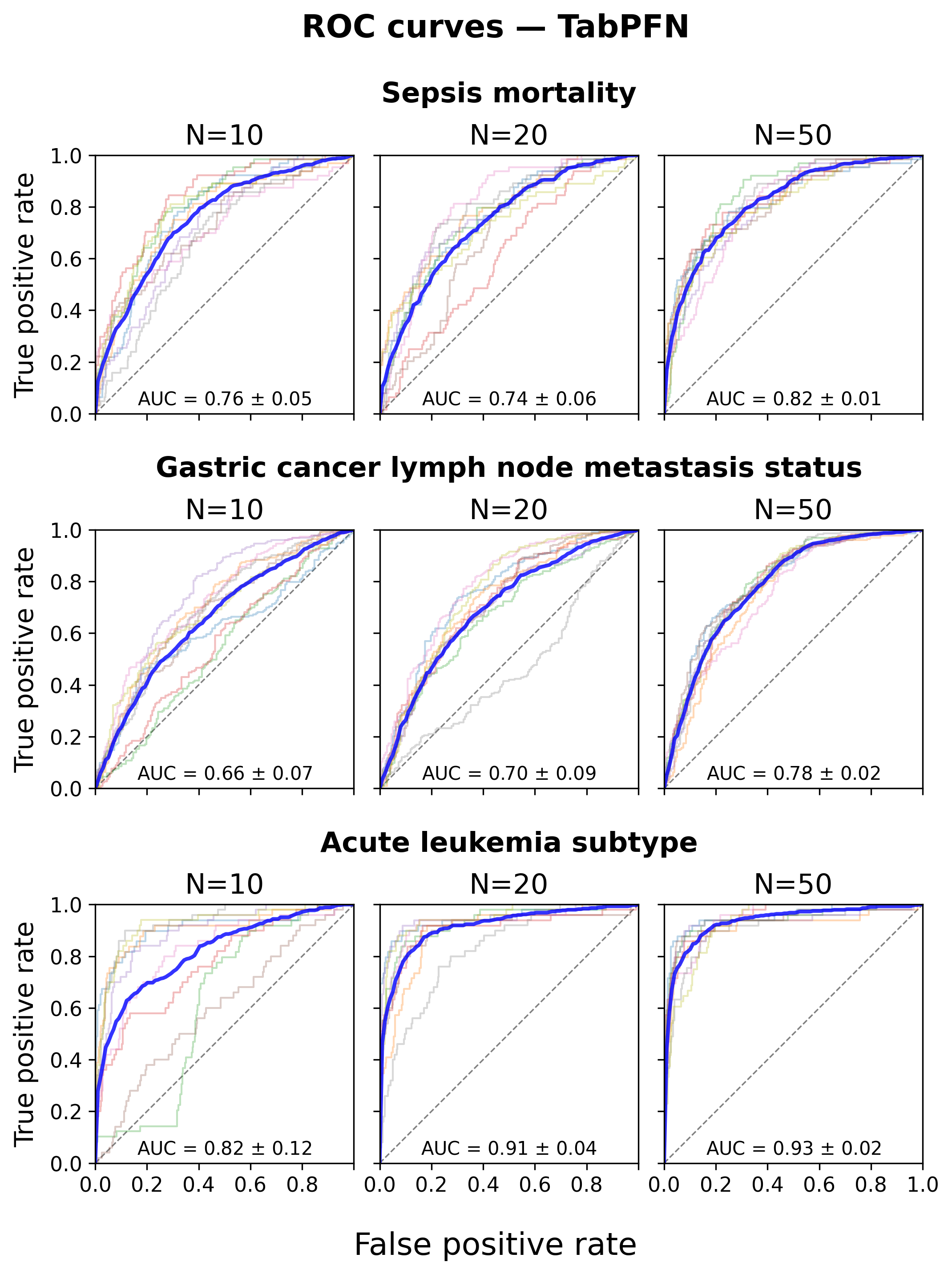


Figure S6: ROC curves for TabPFN for the sepsis (top), gastric cancer (middle), and leukemia (bottom) datasets, and for various train sizes, TR = 10 (left), TR = 20 (center), and TR = 50 (right). The light-colored curves correspond to individual folds, and the blue curve is the mean curve across folds.


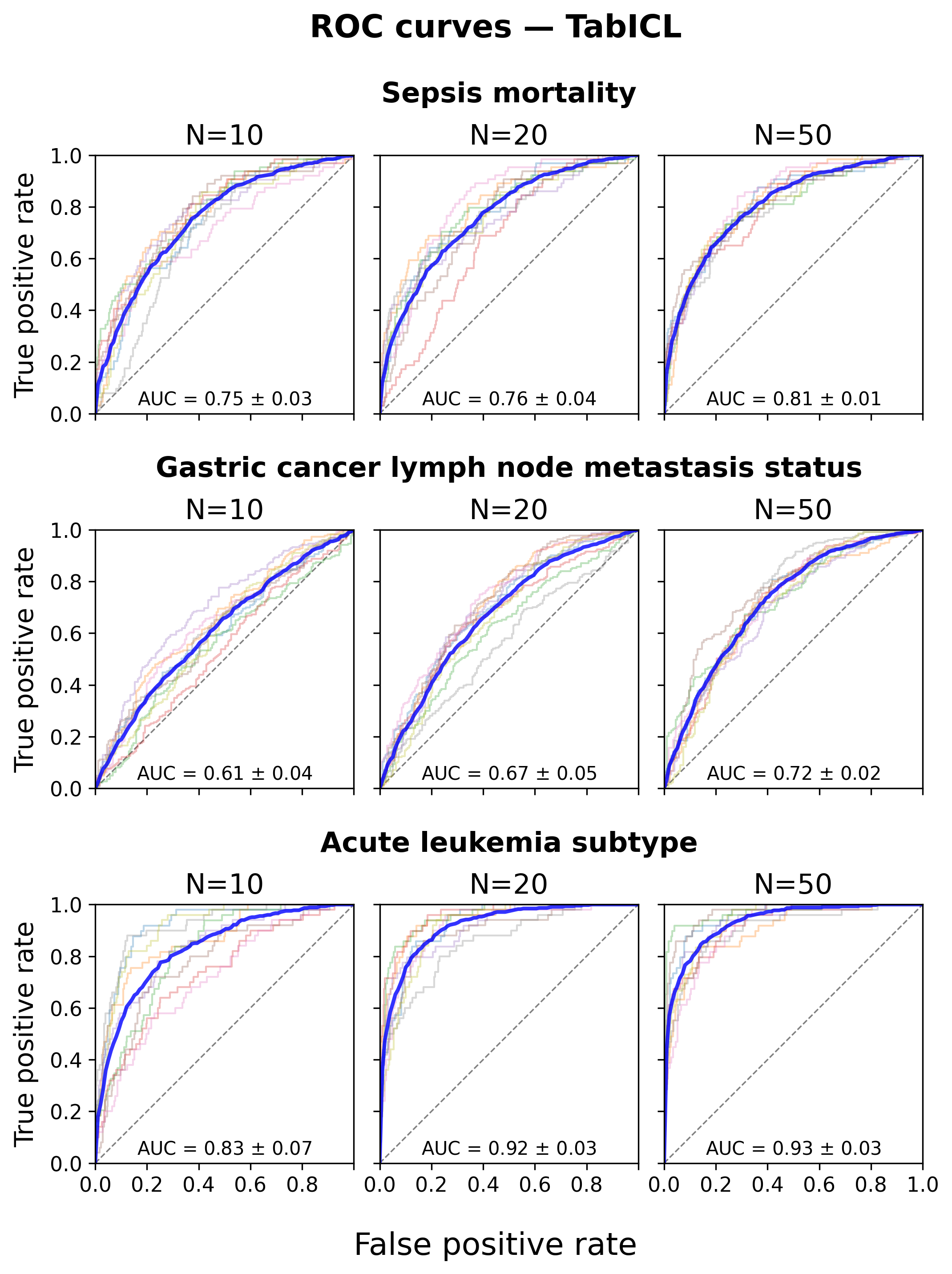


Figure S7: ROC curves for TabICL for the sepsis (top), gastric cancer (middle), and leukemia (bottom) datasets, and for various train sizes, TR = 10 (left), TR = 20 (center), and TR = 50 (right). The light-colored curves correspond to individual folds, and the blue curve is the mean curve across folds.

Confidence intervals

Without LLM calibration


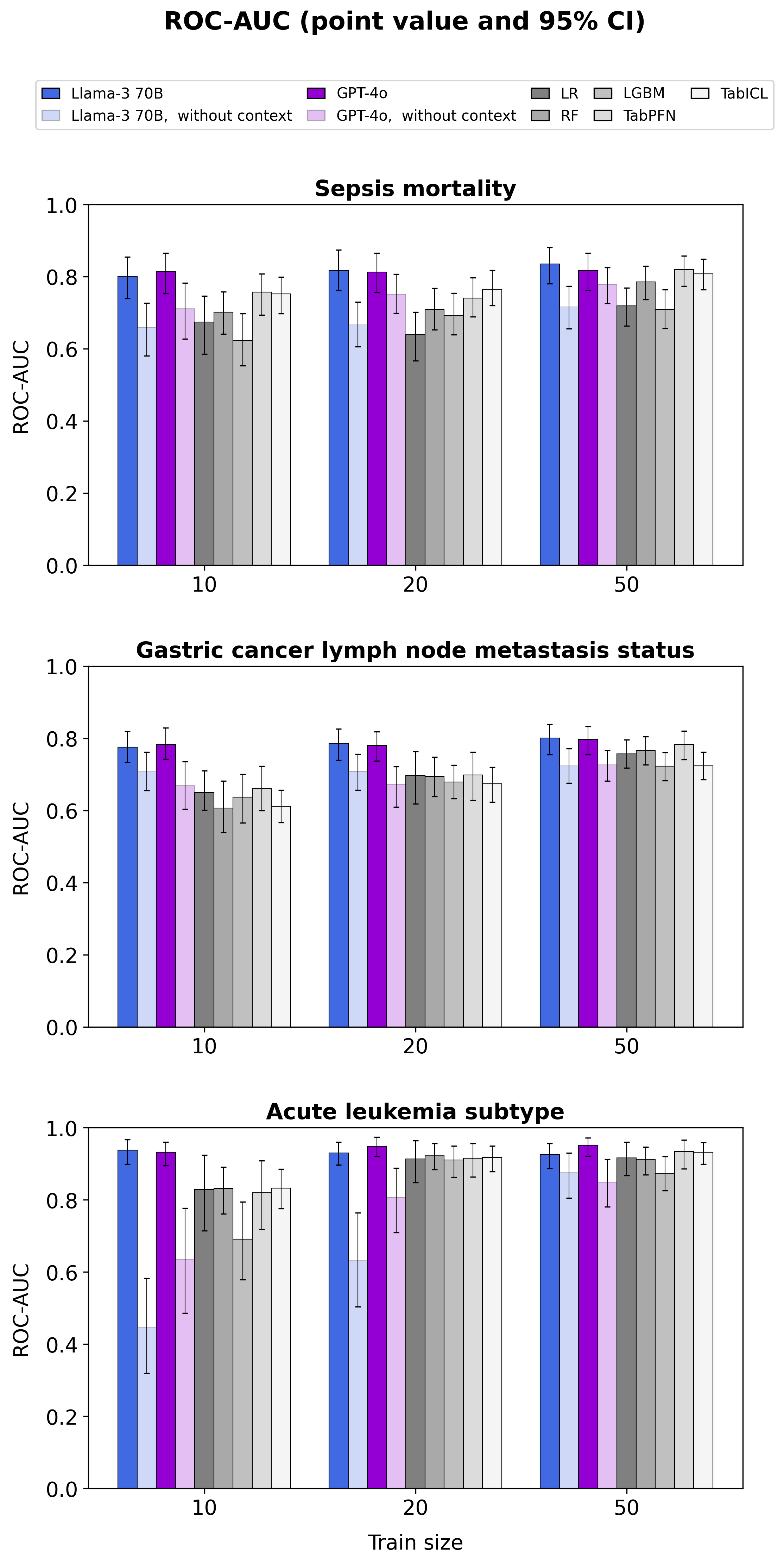


Figure S8: ROC-AUC point value and 95% CI (for the mean across folds), using LLMs with or without context, as well as using conventional ML, for the sepsis (top), gastric cancer (middle), and leukemia (bottom) datasets.


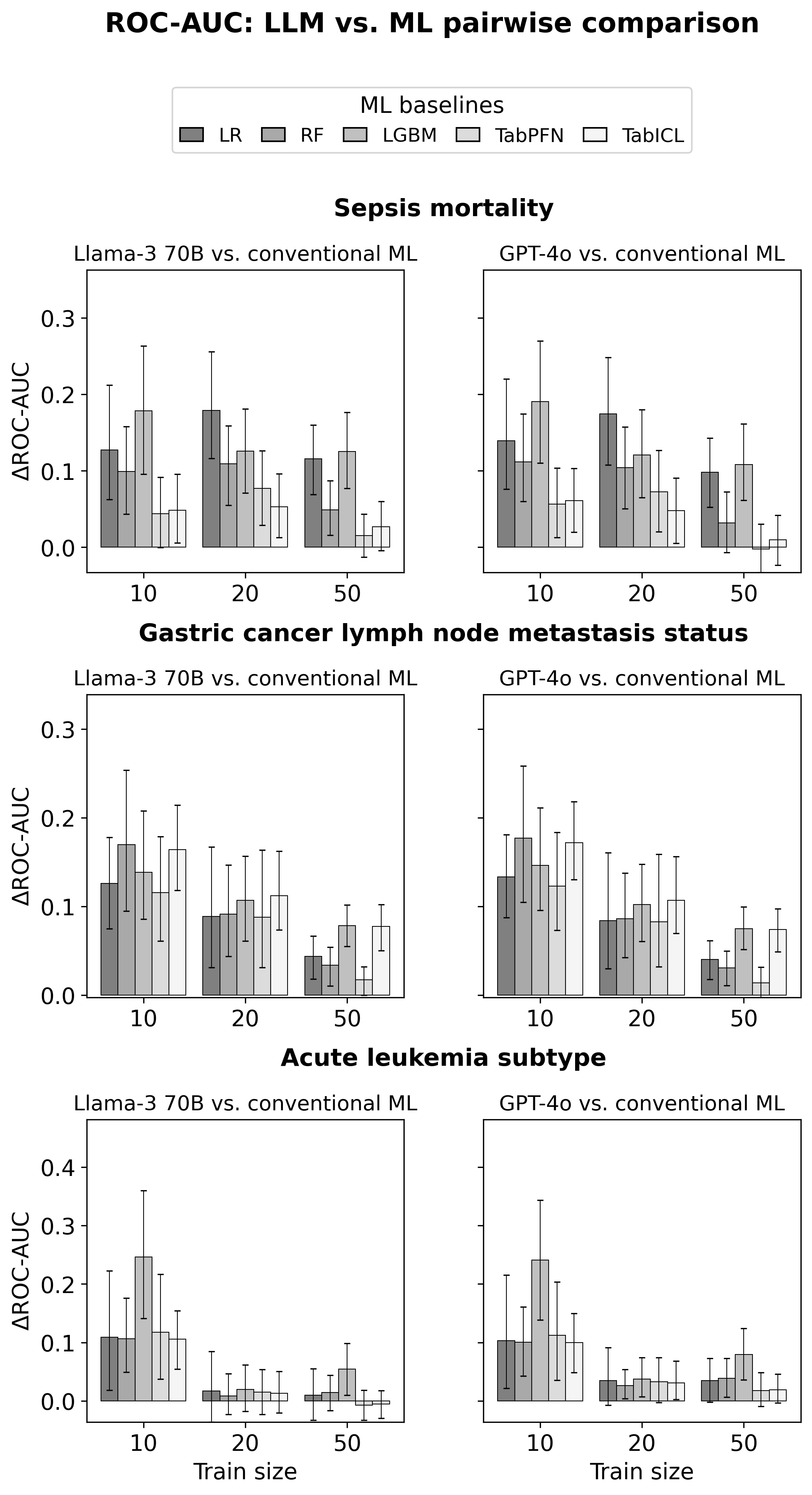


Figure S9: Difference in ROC-AUC between LLM and ML (point value and 95% CI for the mean across folds), for the sepsis (top), gastric cancer (middle), and leukemia (bottom) datasets.


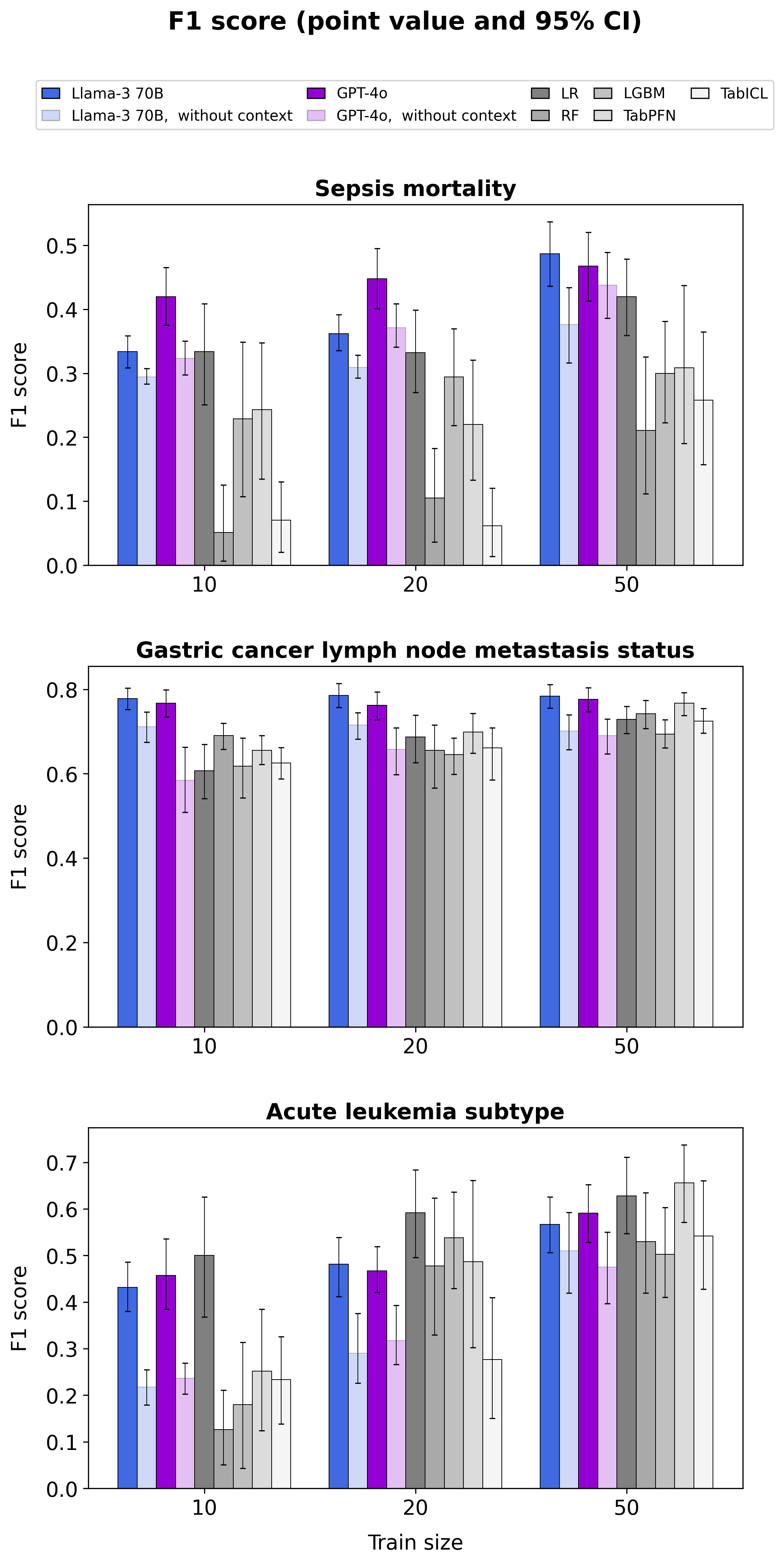


Figure S10: F1 score point value and 95% CI for the mean across folds, using LLMs with or without context, as well as using conventional ML, for the sepsis (top), gastric cancer (middle), and leukemia (bottom) datasets.


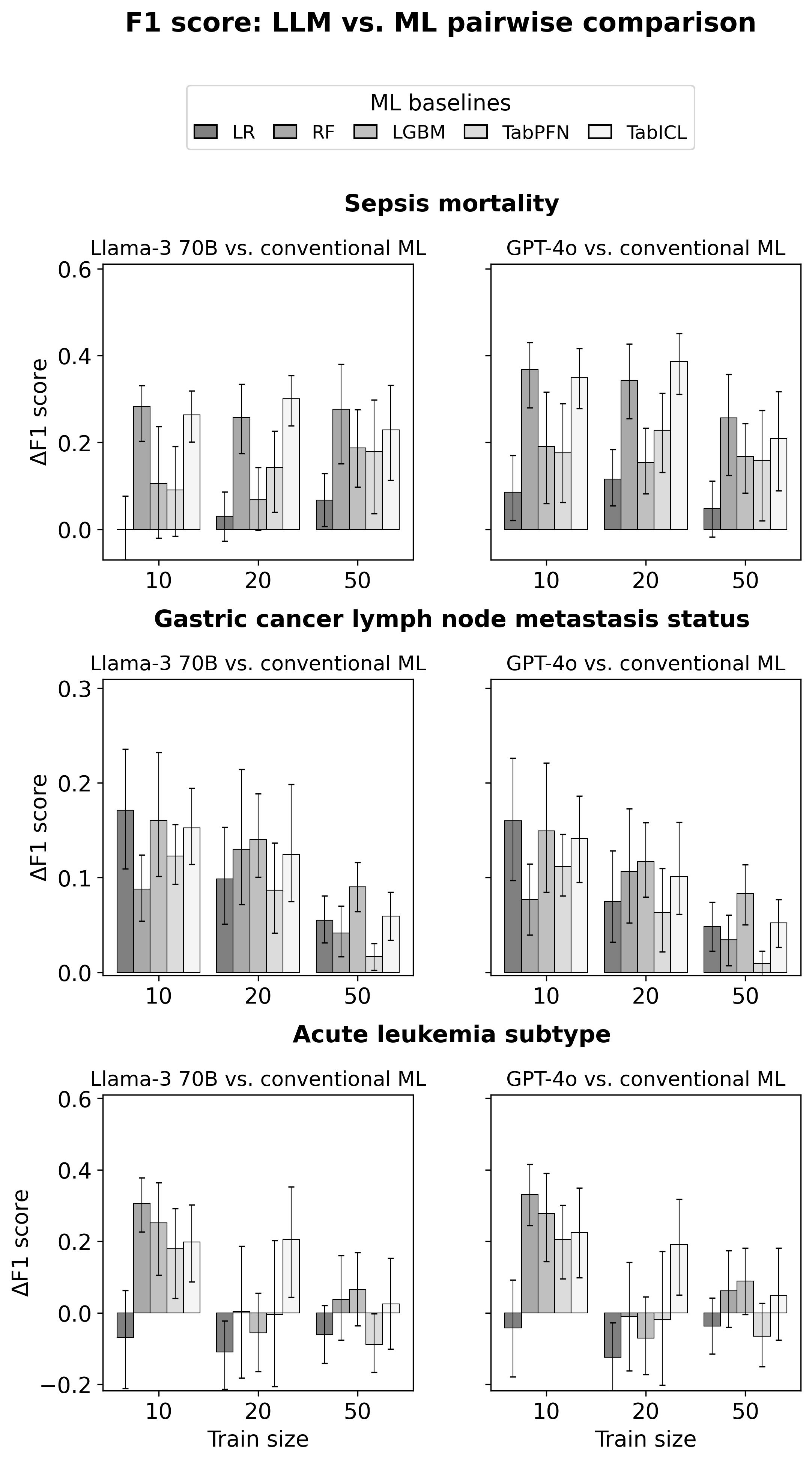


Figure S11: Difference in F1 score between LLM and ML (point value and 95% CI for the mean across folds), for the sepsis (top), gastric cancer (middle), and leukemia (bottom) datasets.


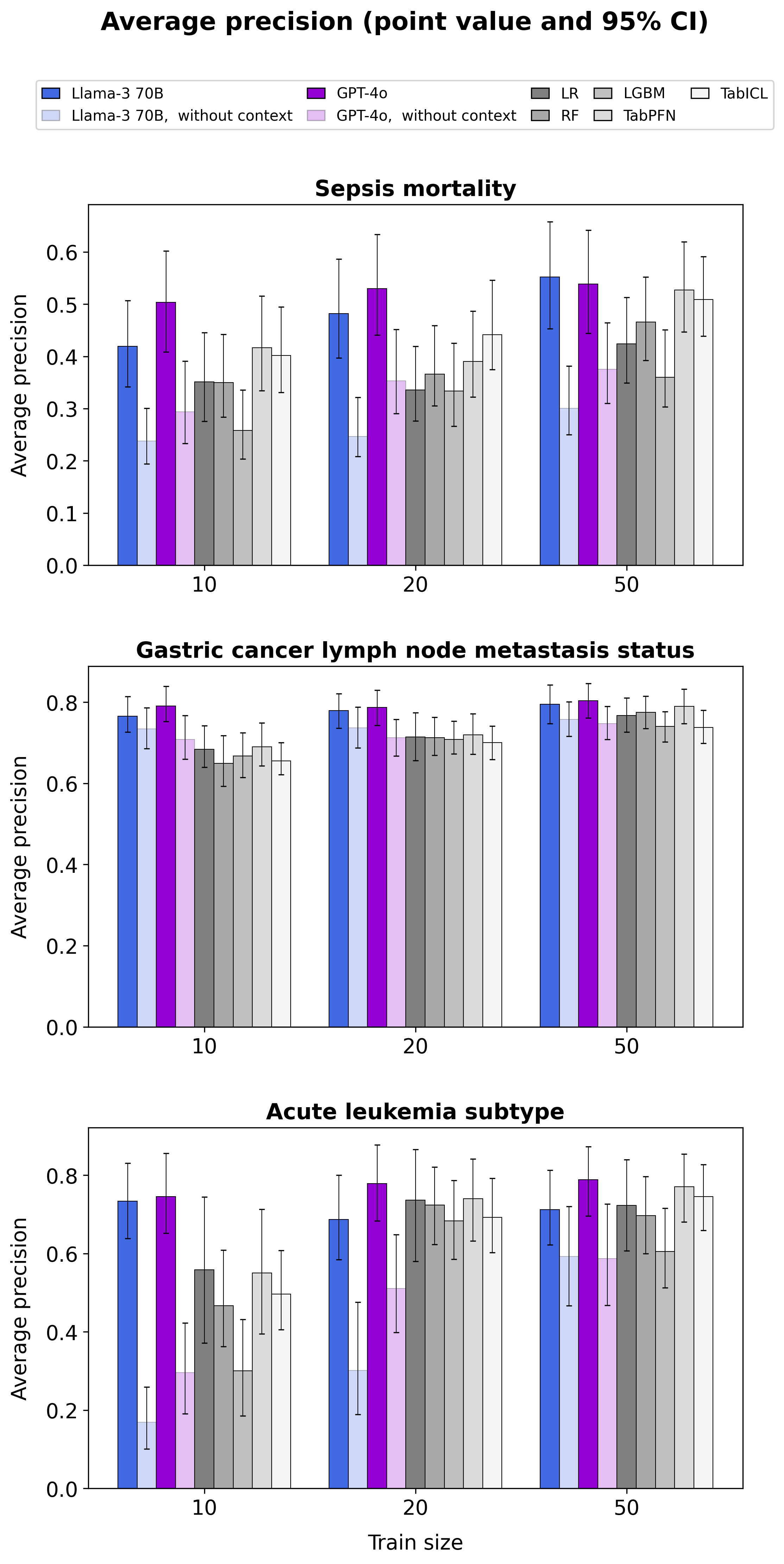


Figure S12: Average Precision (AP) point value and 95% CI for the mean across folds, using LLMs with or without context, as well as using conventional ML, for the sepsis (top), gastric cancer (middle), and leukemia (bottom) datasets.


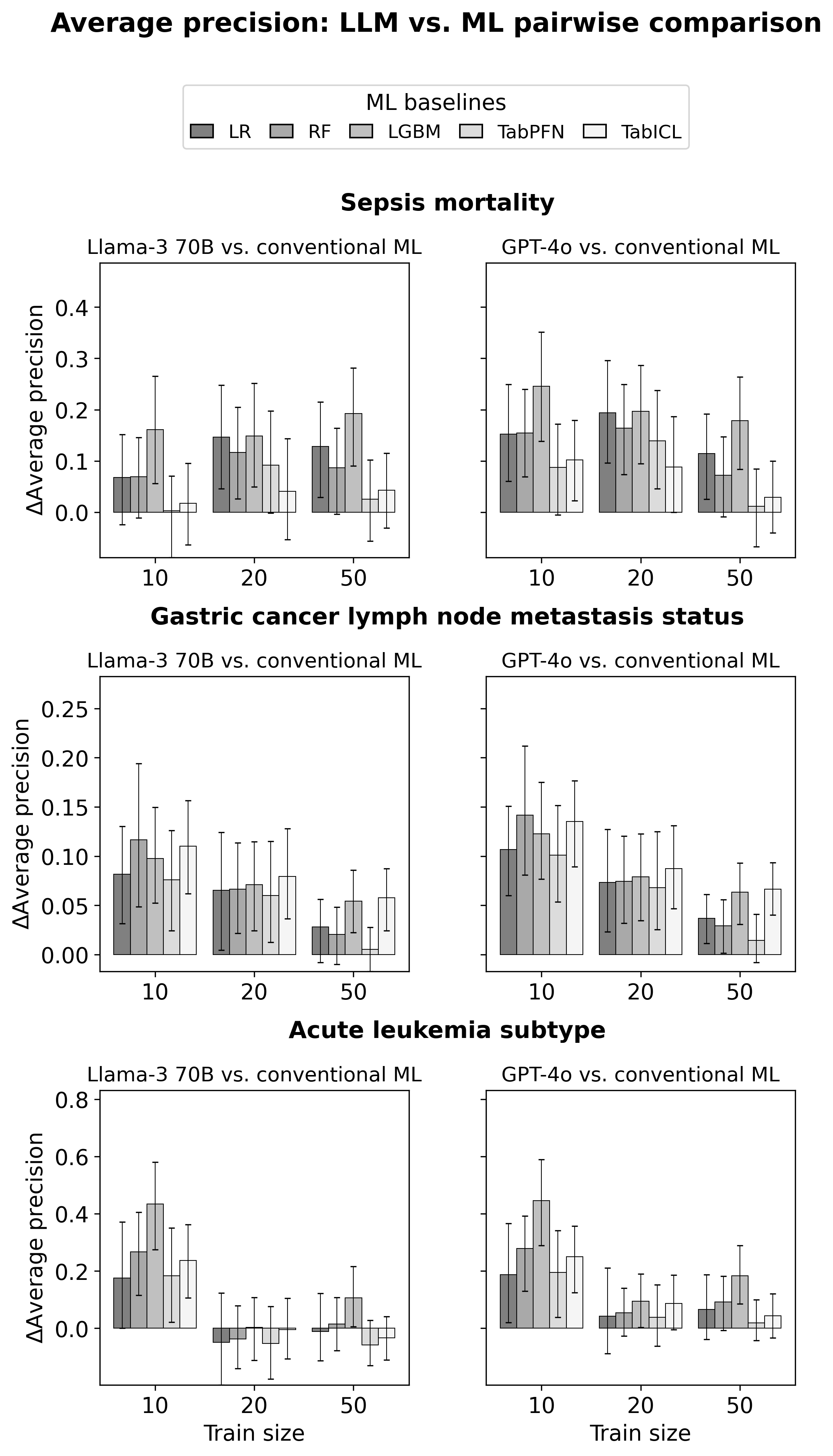


Figure S13: Difference in Average Precision (AP) between LLM and ML (point value and 95% CI for the mean across folds), for the sepsis (top), gastric cancer (middle), and leukemia (bottom) datasets.


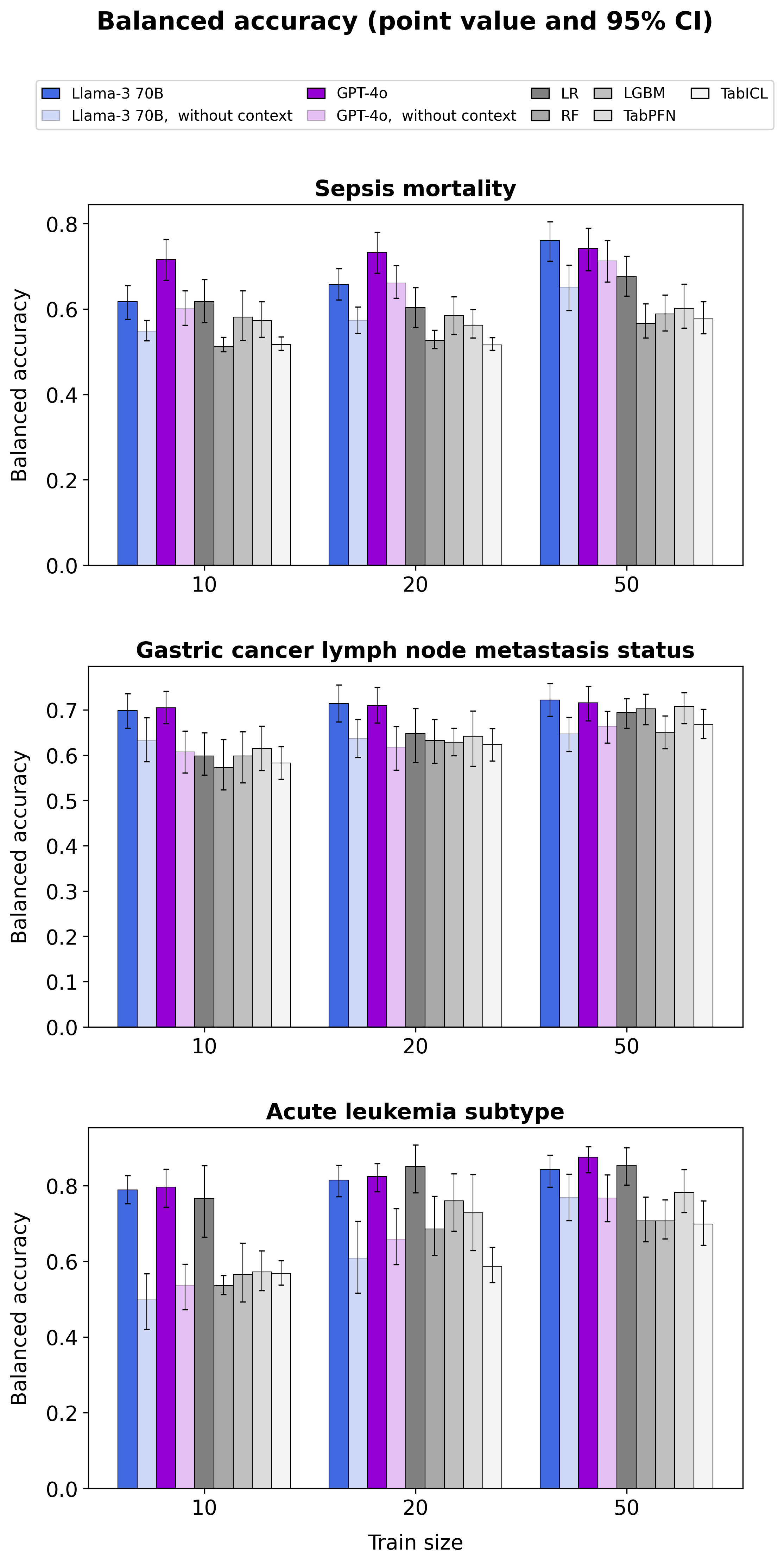


Figure S14: Point value and 95% CI for the mean balanced accuracy across folds, using LLMs with or without context, as well as using conventional ML, for the sepsis (top), gastric cancer (middle), and leukemia (bottom) datasets.


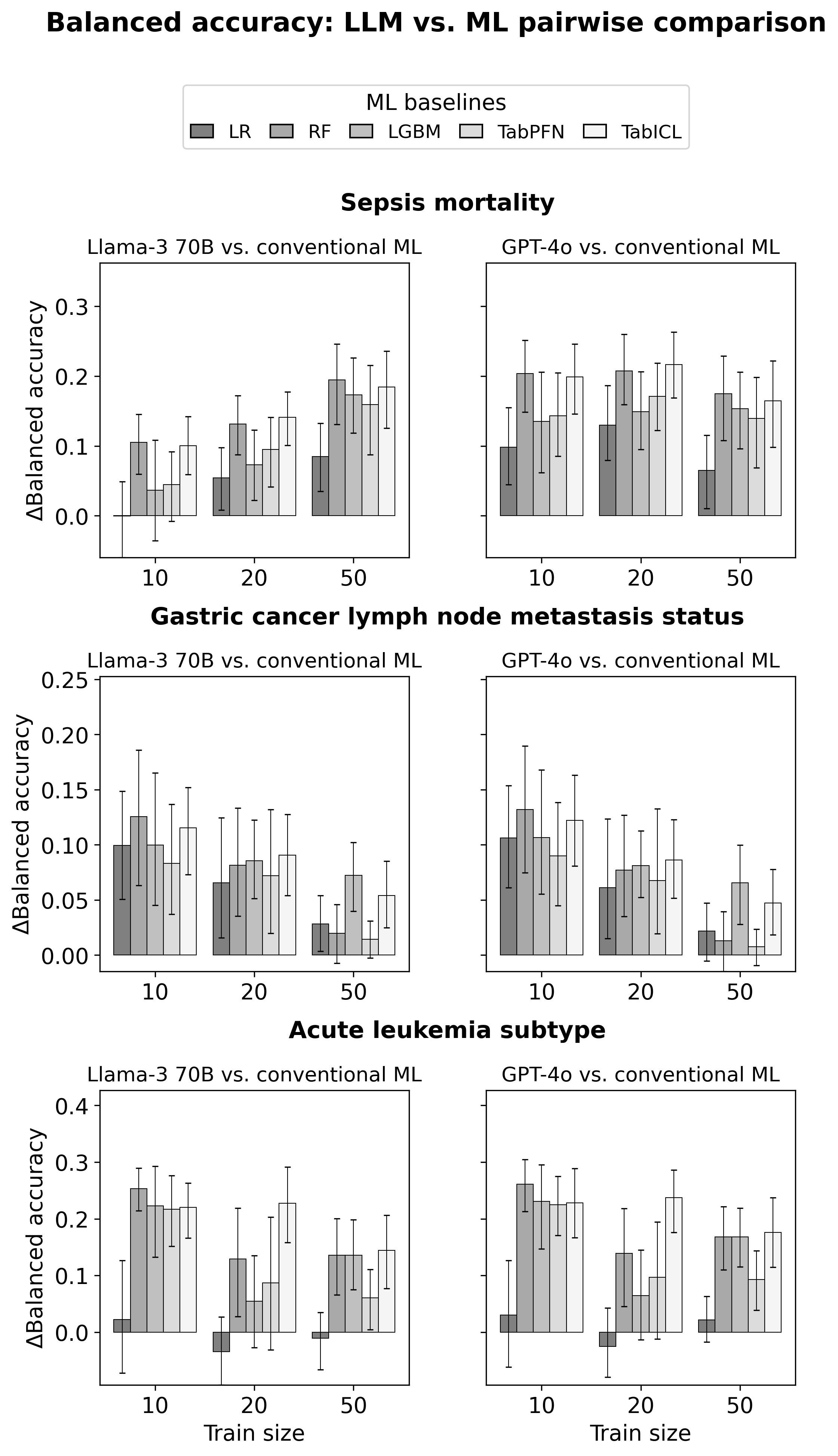


Figure S15: Difference in balanced accuracy between LLM and ML (point value and 95% CI for the mean across folds), for the sepsis (top), gastric cancer (middle), and leukemia (bottom) datasets.


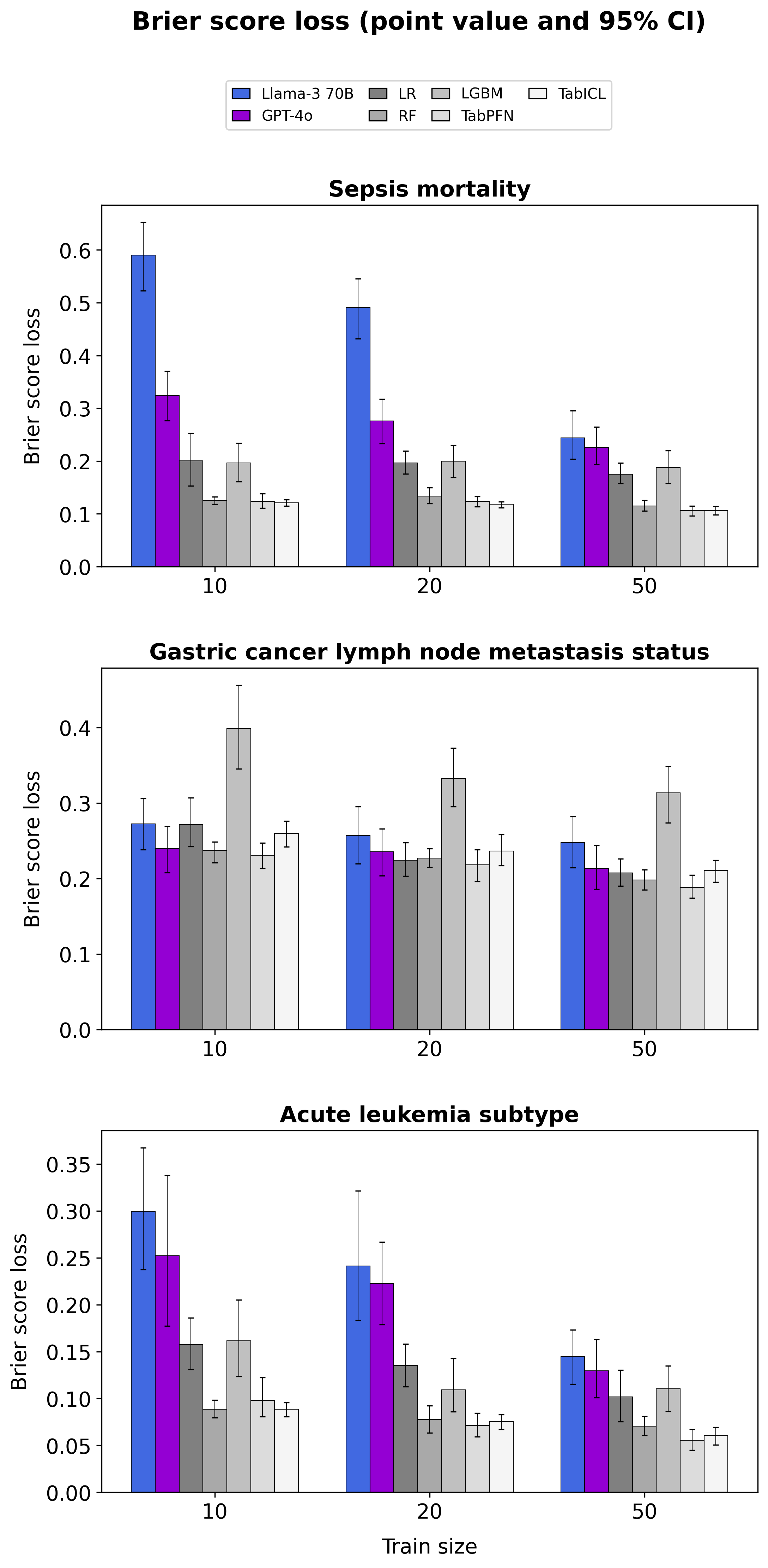


Figure S16: Brier score loss point value and 95% CI for the mean across folds, using LLMs (with context), as well as using conventional ML, for the sepsis (top), gastric cancer (middle), and leukemia (bottom) datasets.


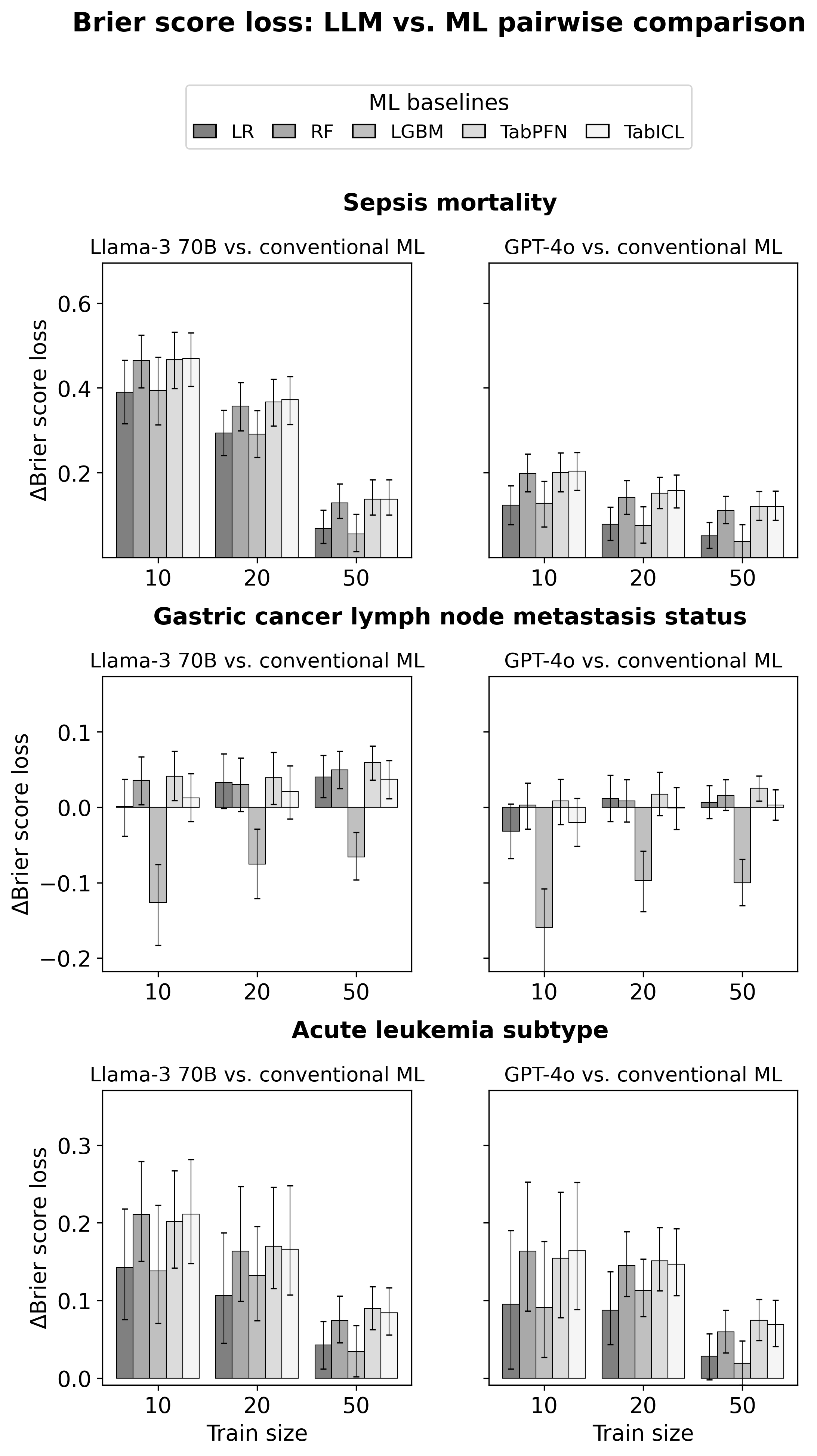


Figure S17: Difference in Brier score loss between LLM and ML (point value and 95% CI for the mean across folds), for the sepsis (top), gastric cancer (middle), and leukemia (bottom) datasets.


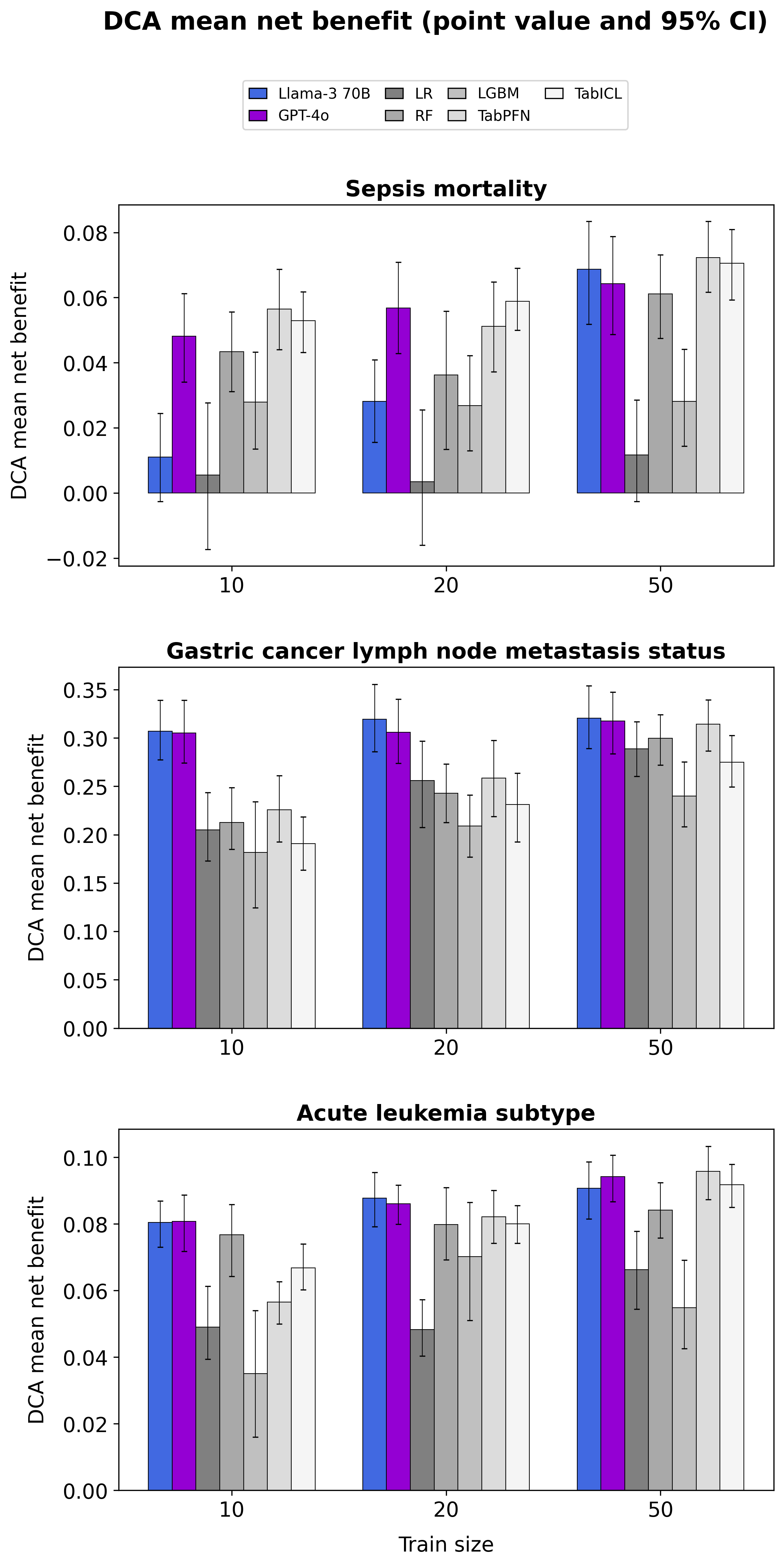


Figure S18: Decision Curve Analysis (DCA) mean net benefit (across the clinically relevant thresholds) point value and 95% CI for the mean (across folds), using LLMs (with context), as well as using conventional ML, for the sepsis (top), gastric cancer (middle), and leukemia (bottom) datasets.


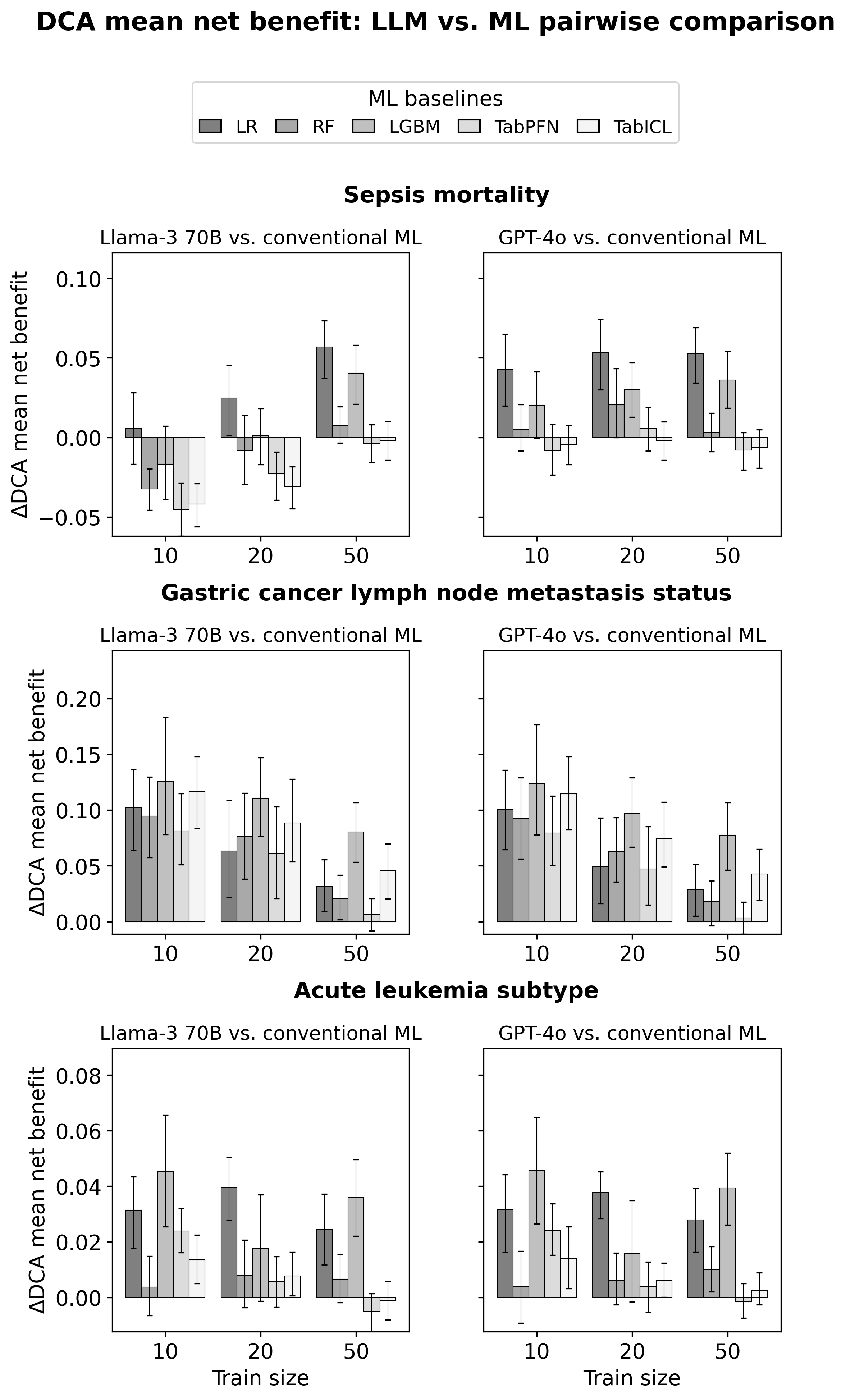


Figure S19: Difference in Decision Curve Analysis (DCA) mean net benefit (across the clinically relevant thresholds) between LLM and ML (point value and 95% CI for the mean across folds), for the sepsis (top), gastric cancer (middle), and leukemia (bottom) datasets.

Confidence intervals

With LLM beta calibration


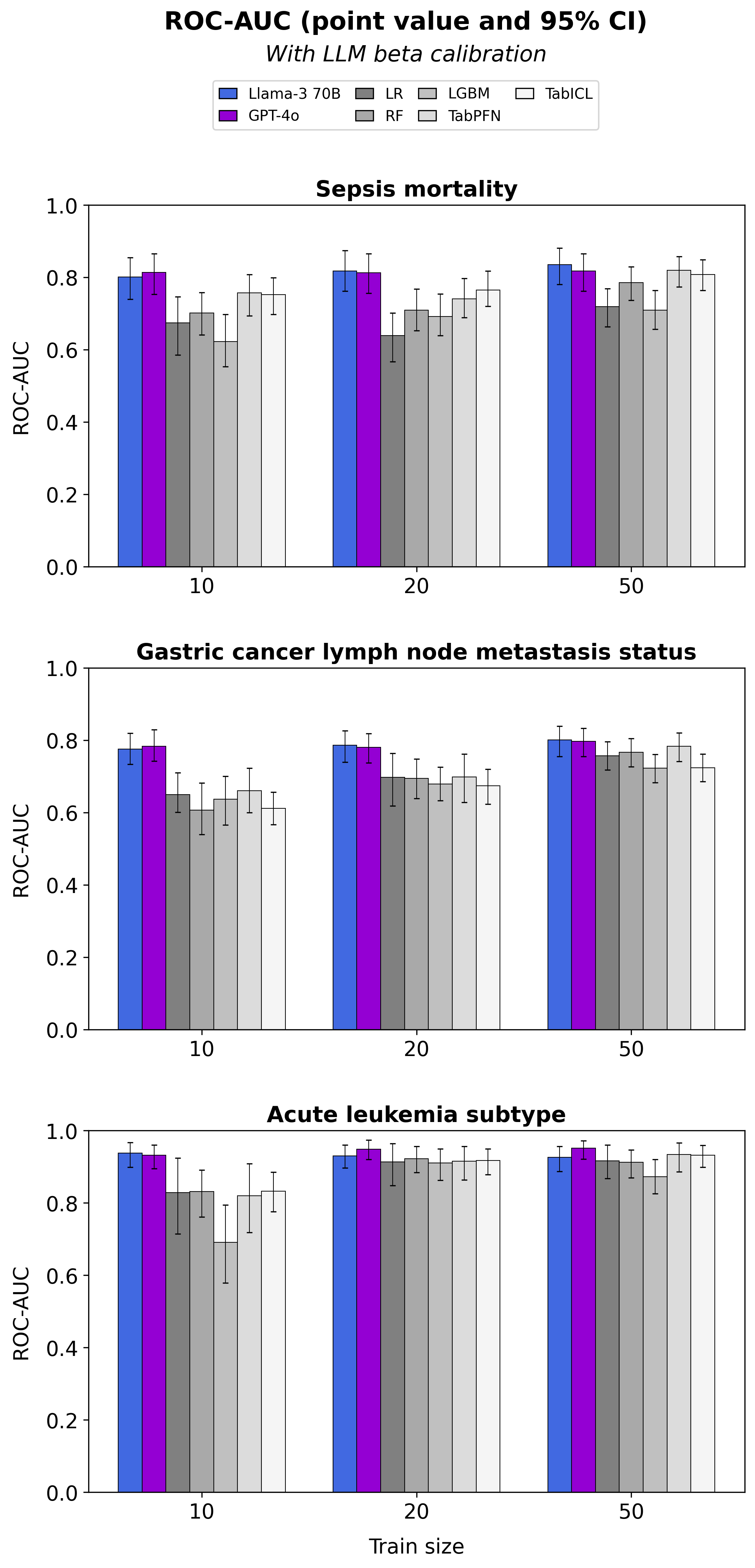


Figure S20: Point value and 95% CI for the mean ROC-AUC across folds, using LLMs (with context), as well as using conventional ML, for the sepsis (top), gastric cancer (middle), and leukemia (bottom) datasets.


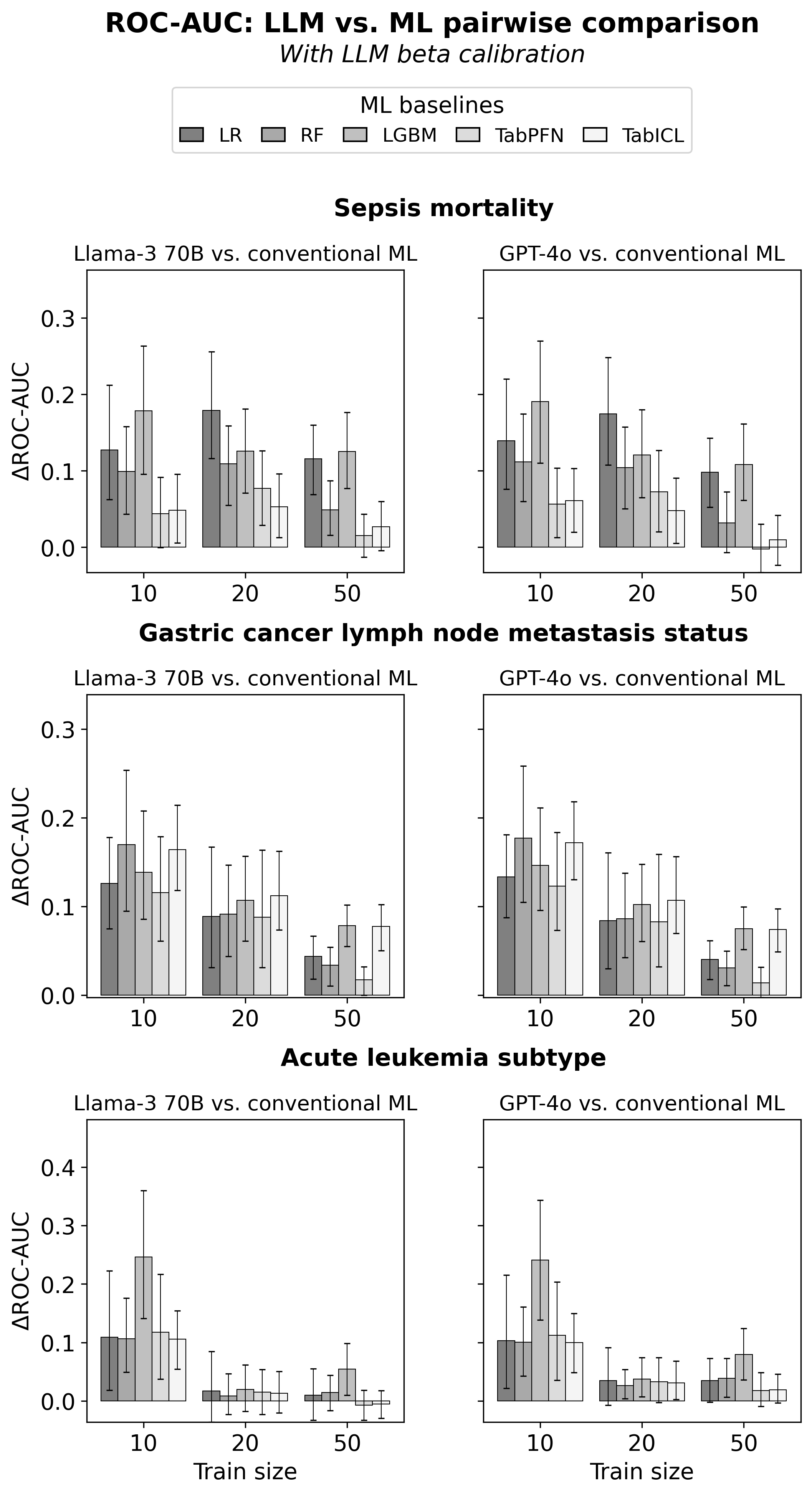


Figure S21: Difference in ROC-AUC between LLM and ML (point value and 95% CI for the mean across folds), for the sepsis (top), gastric cancer (middle), and leukemia (bottom) datasets.


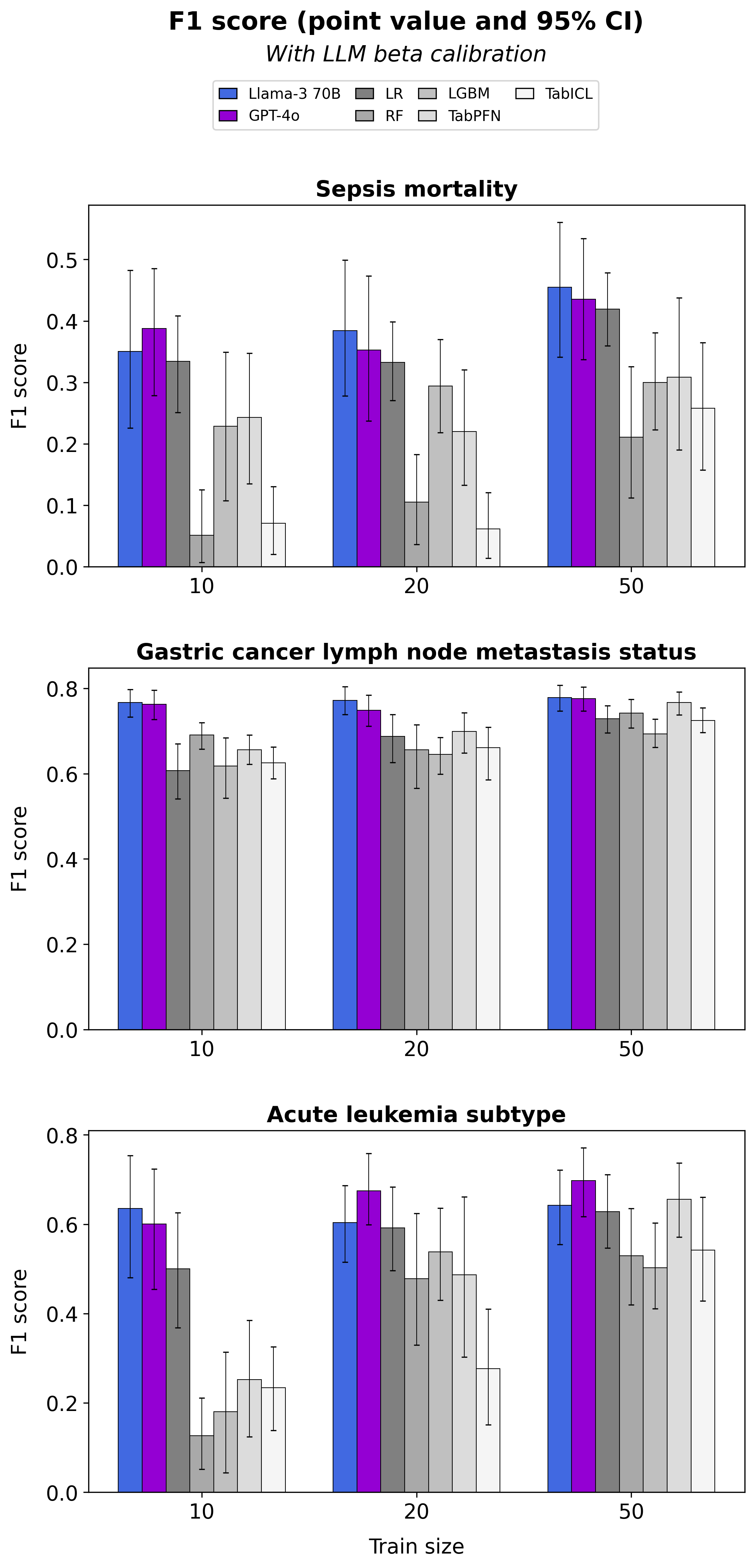


Figure S22: Point value and 95% CI for the mean F1 score across folds, using LLMs (with context), as well as using conventional ML, for the sepsis (top), gastric cancer (middle), and leukemia (bottom) datasets.


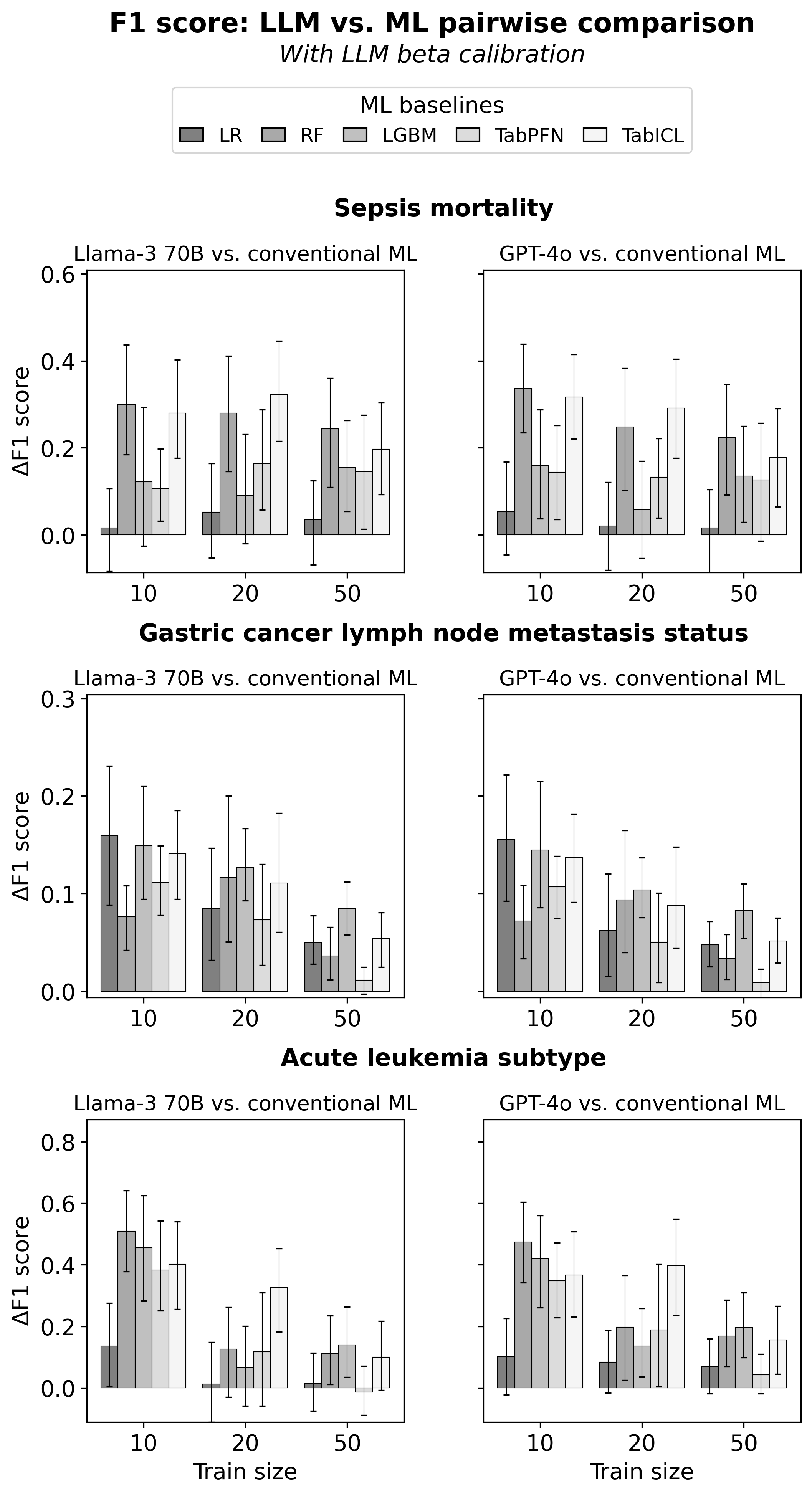


Figure S23: Difference in F1 score between LLM and ML (point value and 95% CI for the mean across folds), for the sepsis (top), gastric cancer (middle), and leukemia (bottom) datasets.


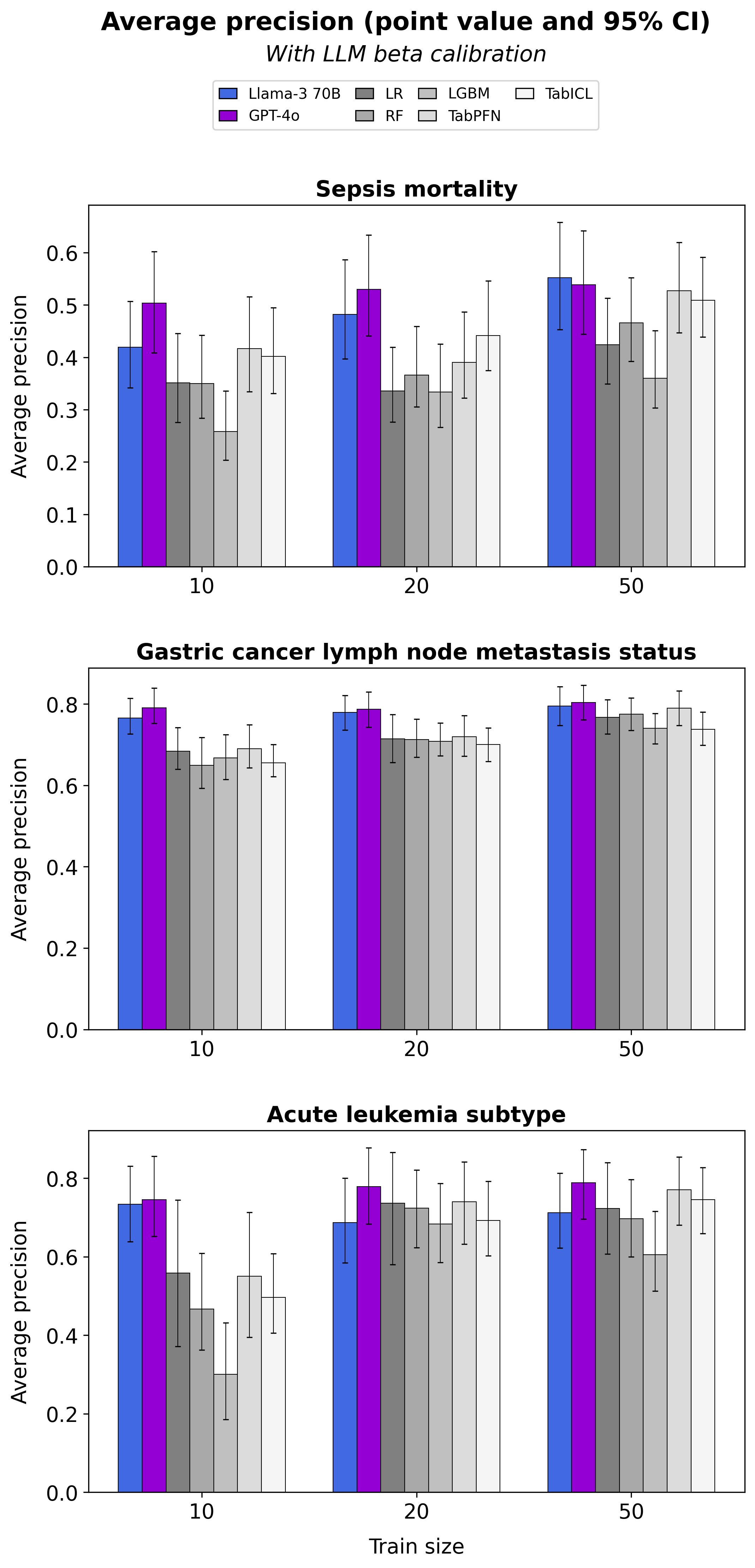


Figure S24: Point value and 95% CI for the mean Average Precision (AP) across folds, using LLMs (with context), as well as using conventional ML, for the sepsis (top), gastric cancer (middle), and leukemia (bottom) datasets.


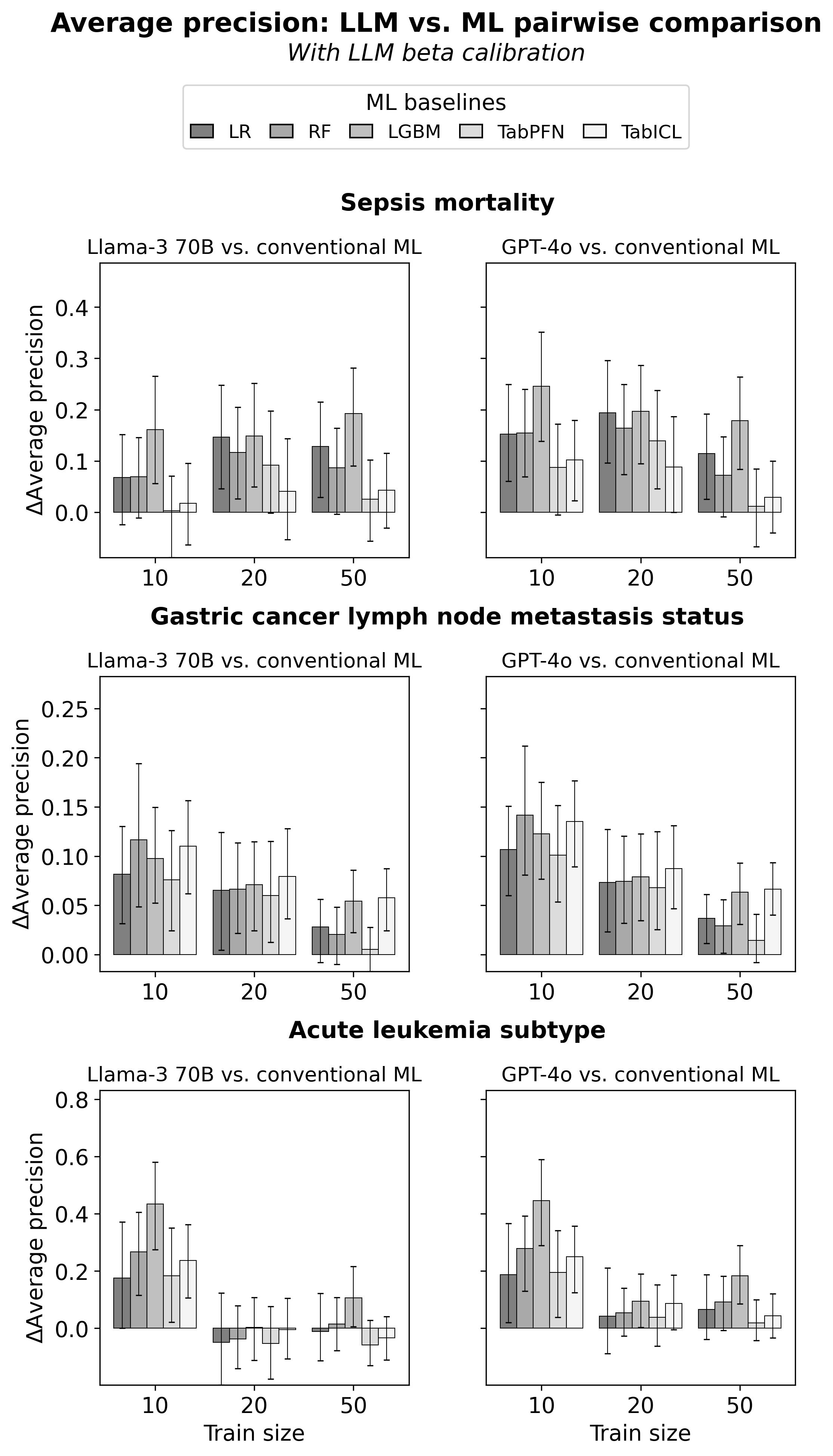


Figure S25: Difference in Average Precision (AP) between LLM and ML (point value and 95% CI for the mean across folds), for the sepsis (top), gastric cancer (middle), and leukemia (bottom) datasets.


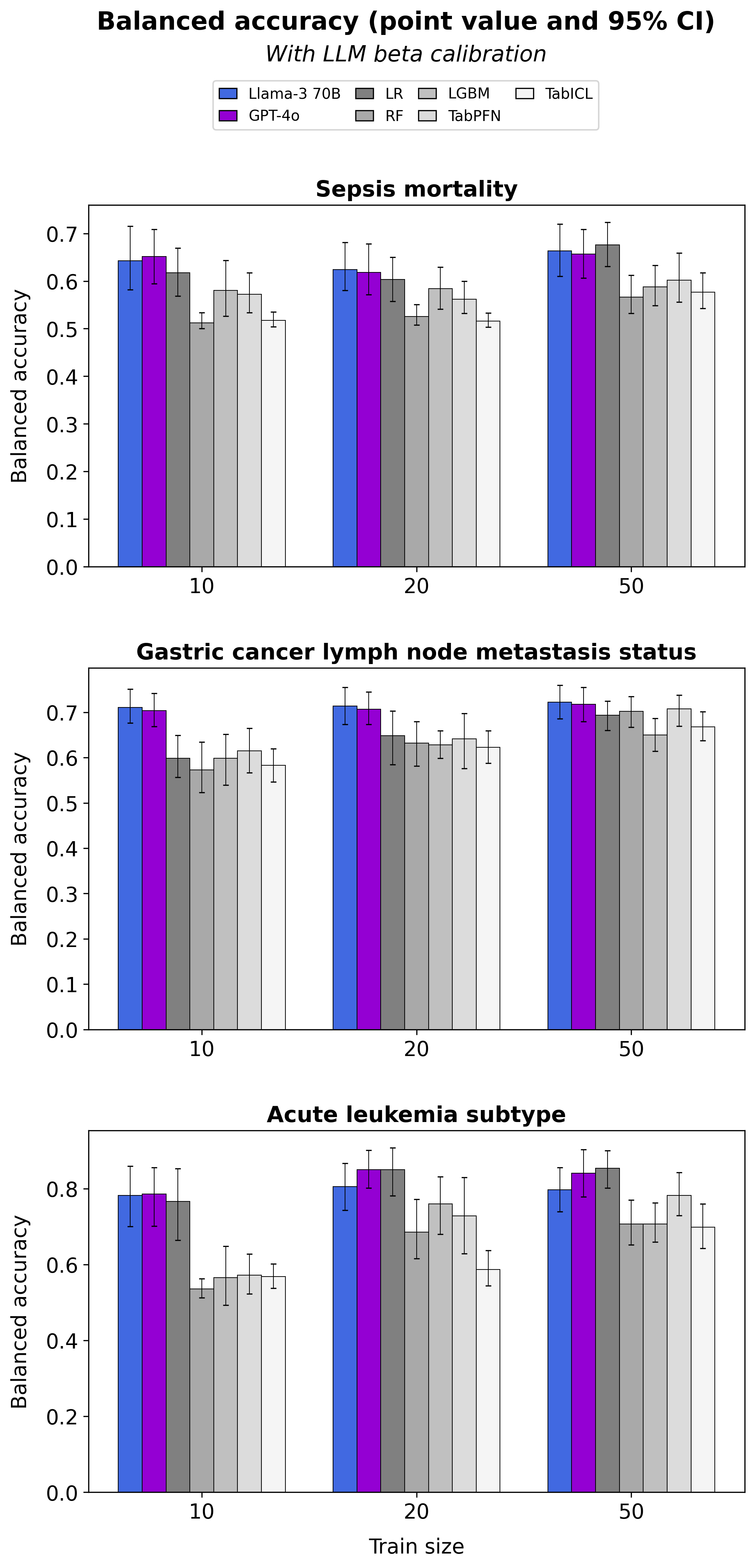


Figure S26: Point value and 95% CI for the mean balanced accuracy across folds, using LLMs (with context), as well as using conventional ML, for the sepsis (top), gastric cancer (middle), and leukemia (bottom) datasets.


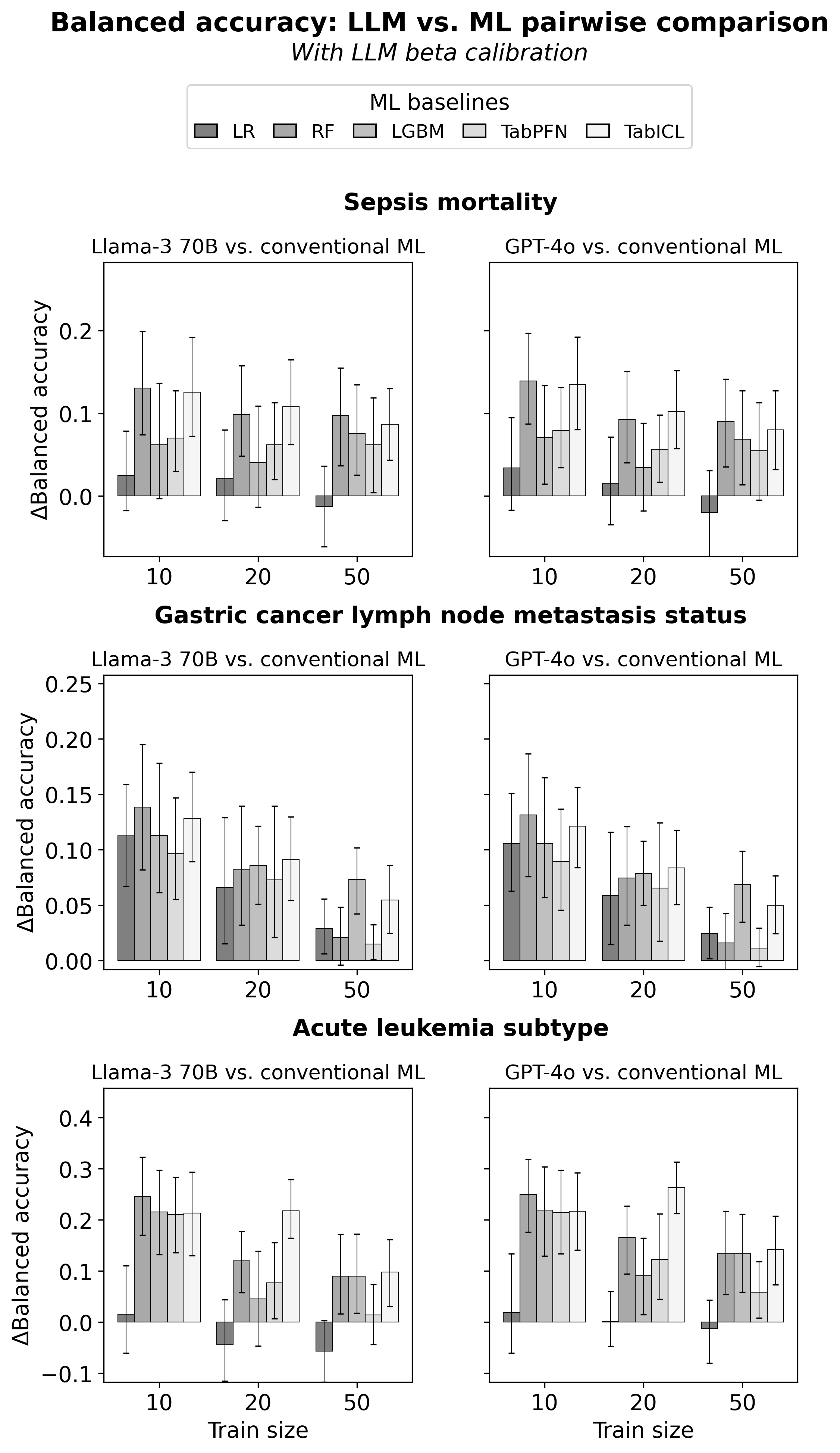


Figure S27: Difference in balanced accuracy between LLM and ML (point value and 95% CI for the mean across folds), for the sepsis (top), gastric cancer (middle), and leukemia (bottom) datasets.


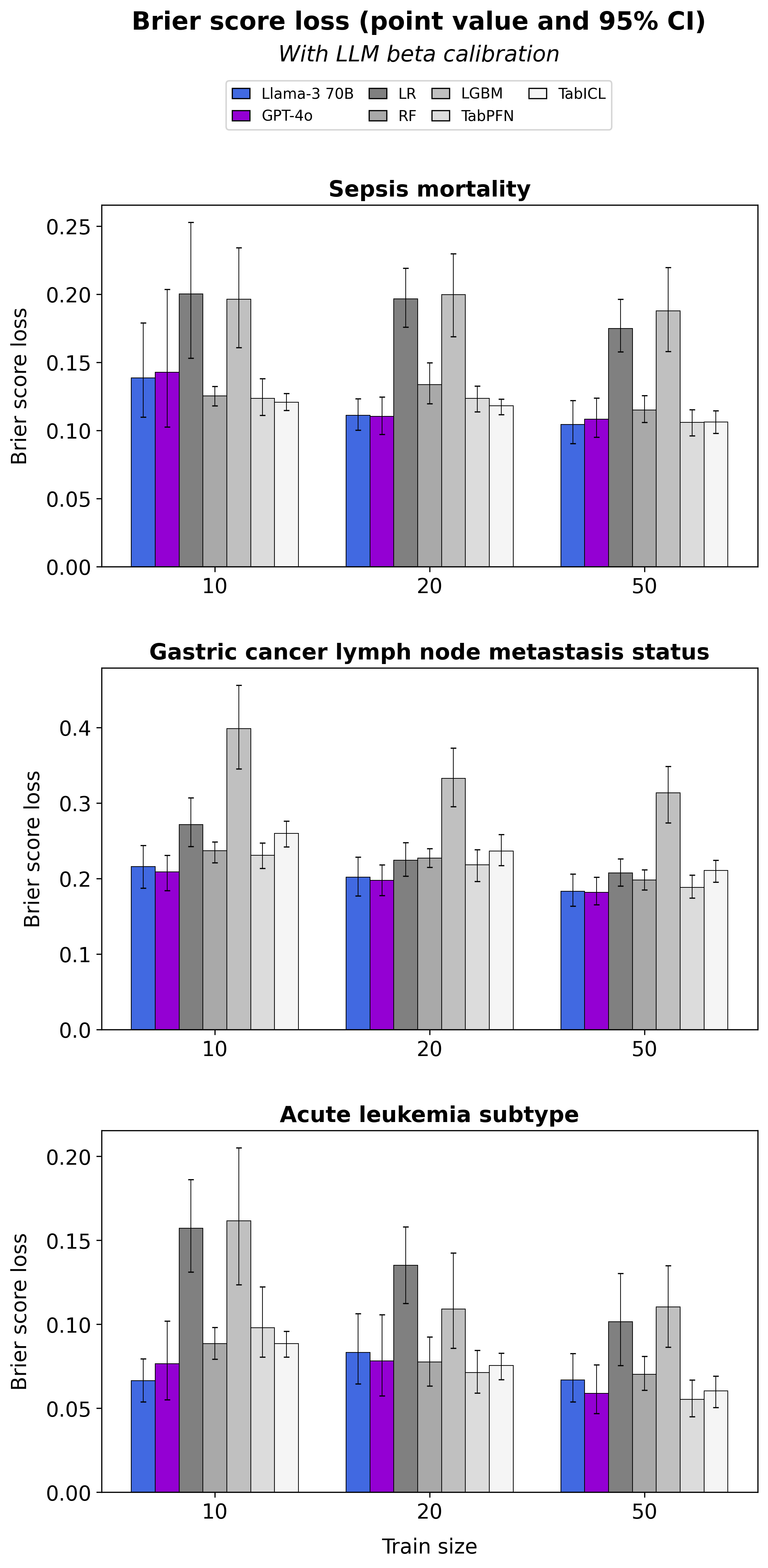


Figure S28: Point value and 95% CI for the mean Brier score loss across folds, using LLMs (with context), as well as using conventional ML, for the sepsis (top), gastric cancer (middle), and leukemia (bottom) datasets.


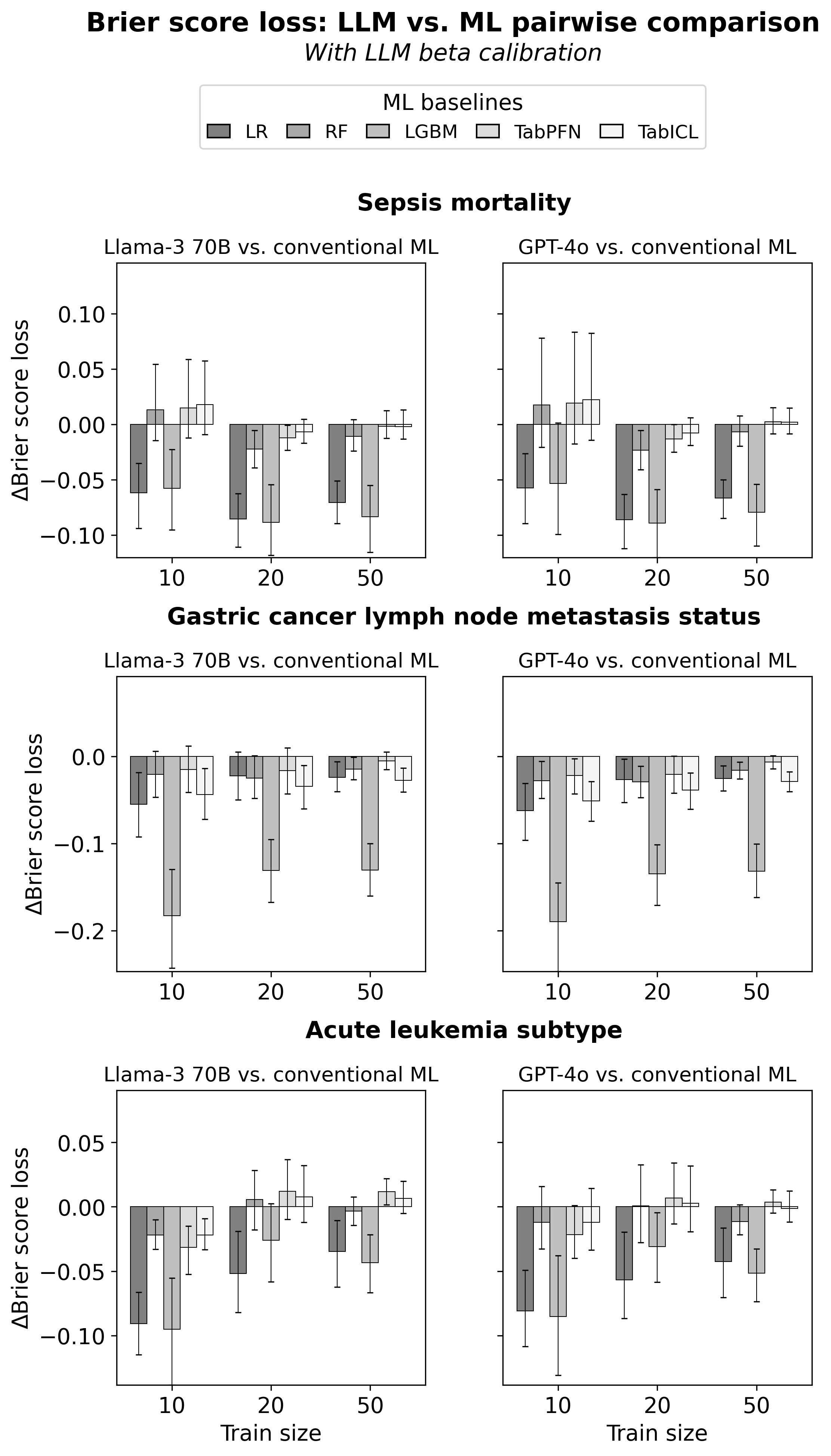


Figure S29: Difference in Brier score loss between LLM and ML (point value and 95% CI for the mean across folds), for the sepsis (top), gastric cancer (middle), and leukemia (bottom) datasets.


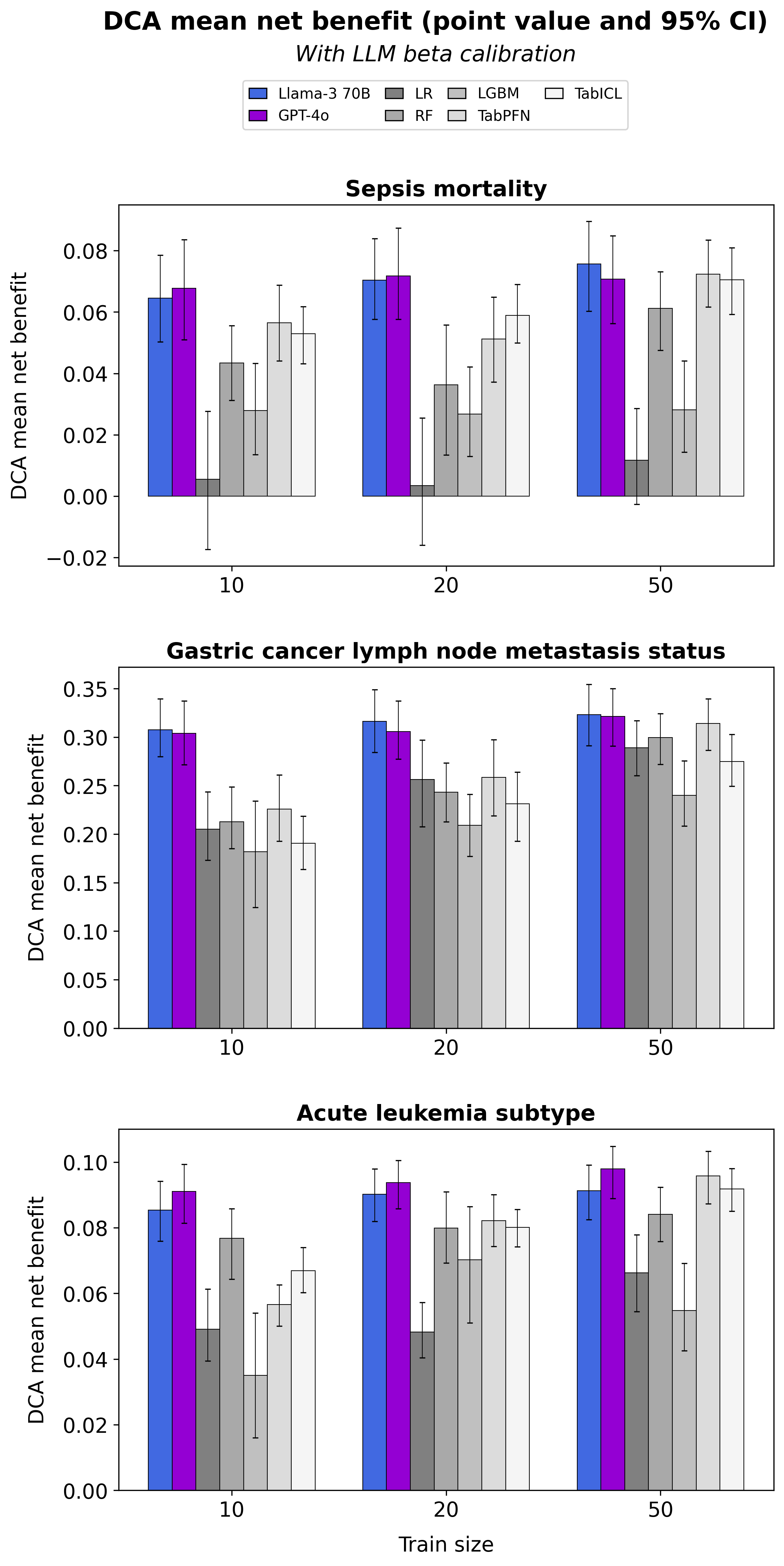


Figure S30: Point value and 95% CI for the mean (across folds) Decision Curve Analysis (DCA) mean net benefit (across the clinically relevant thresholds), using LLMs (with context), as well as using conventional ML, for the sepsis (top), gastric cancer (middle), and leukemia (bottom) datasets.


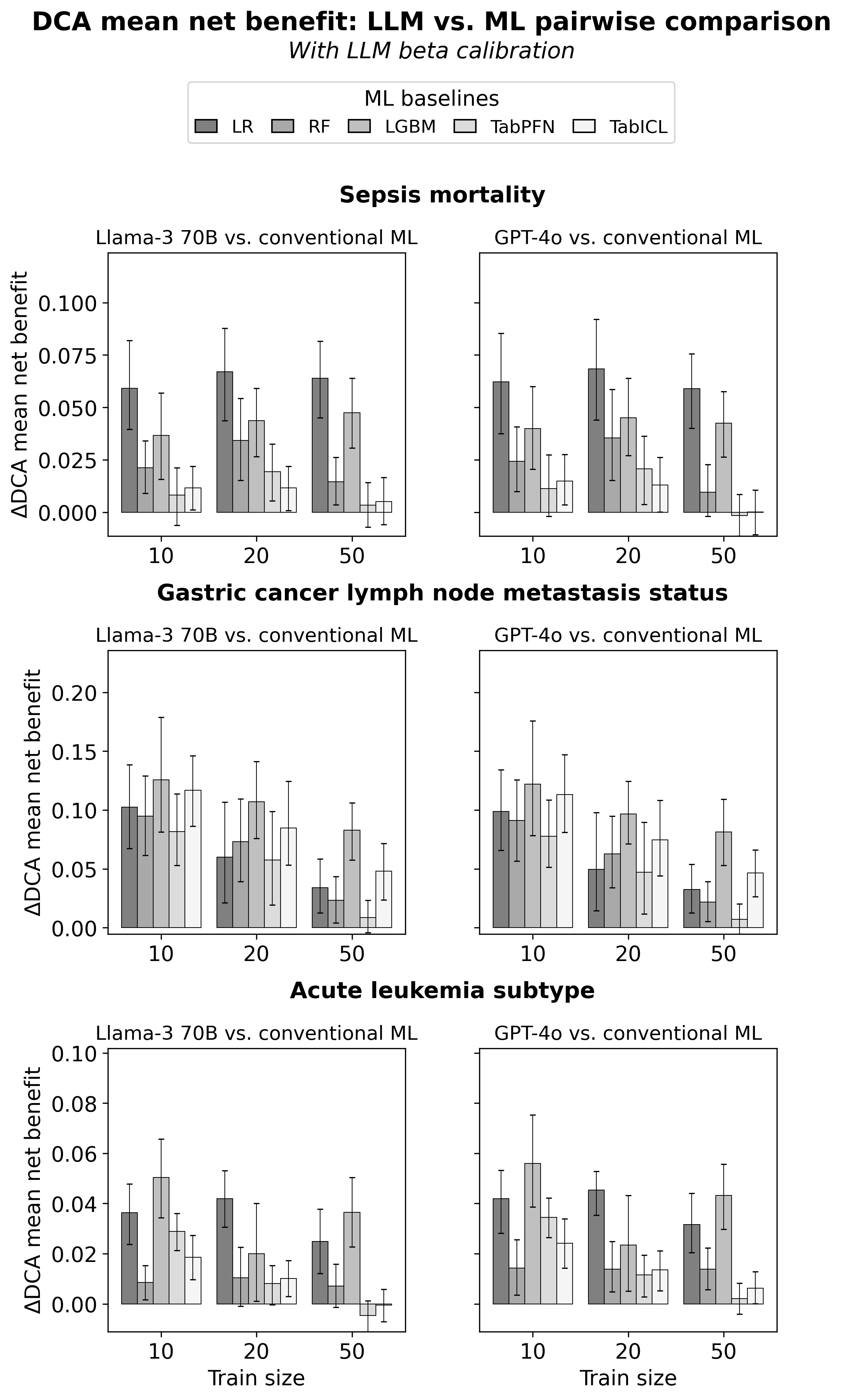


Figure S31: Difference in Decision Curve Analysis (DCA) mean net benefit (across the clinically relevant thresholds) between LLM and ML (point value and 95% CI for the mean across folds), for the sepsis (top), gastric cancer (middle), and leukemia (bottom) datasets.

Prediction intervals

Without LLM calibration


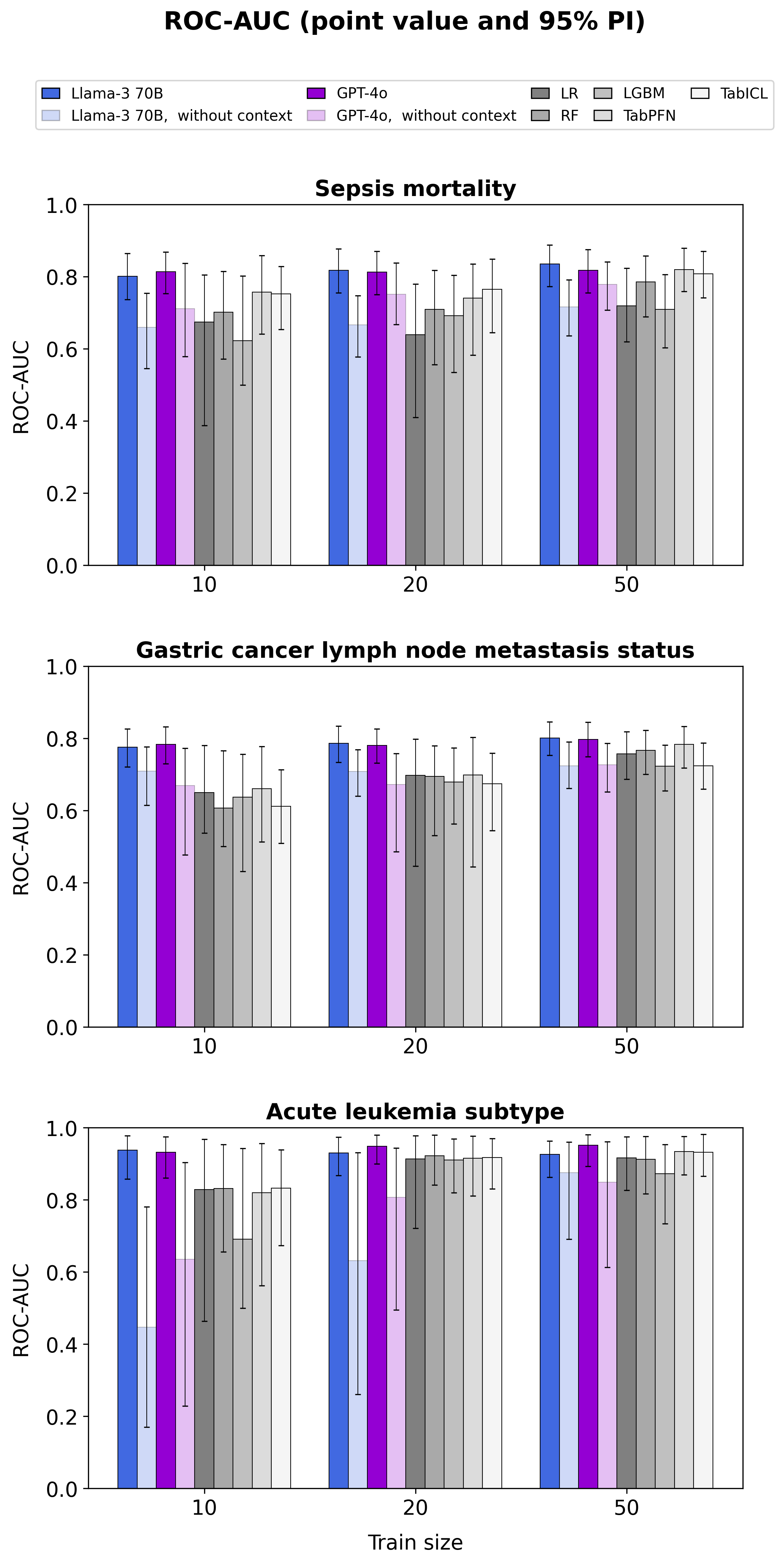


Figure S32: Point value (mean across folds) and 95% prediction interval for the ROC-AUC, using LLMs with or without context, as well as using conventional ML, for the sepsis (top), gastric cancer (middle), and leukemia (bottom) datasets.


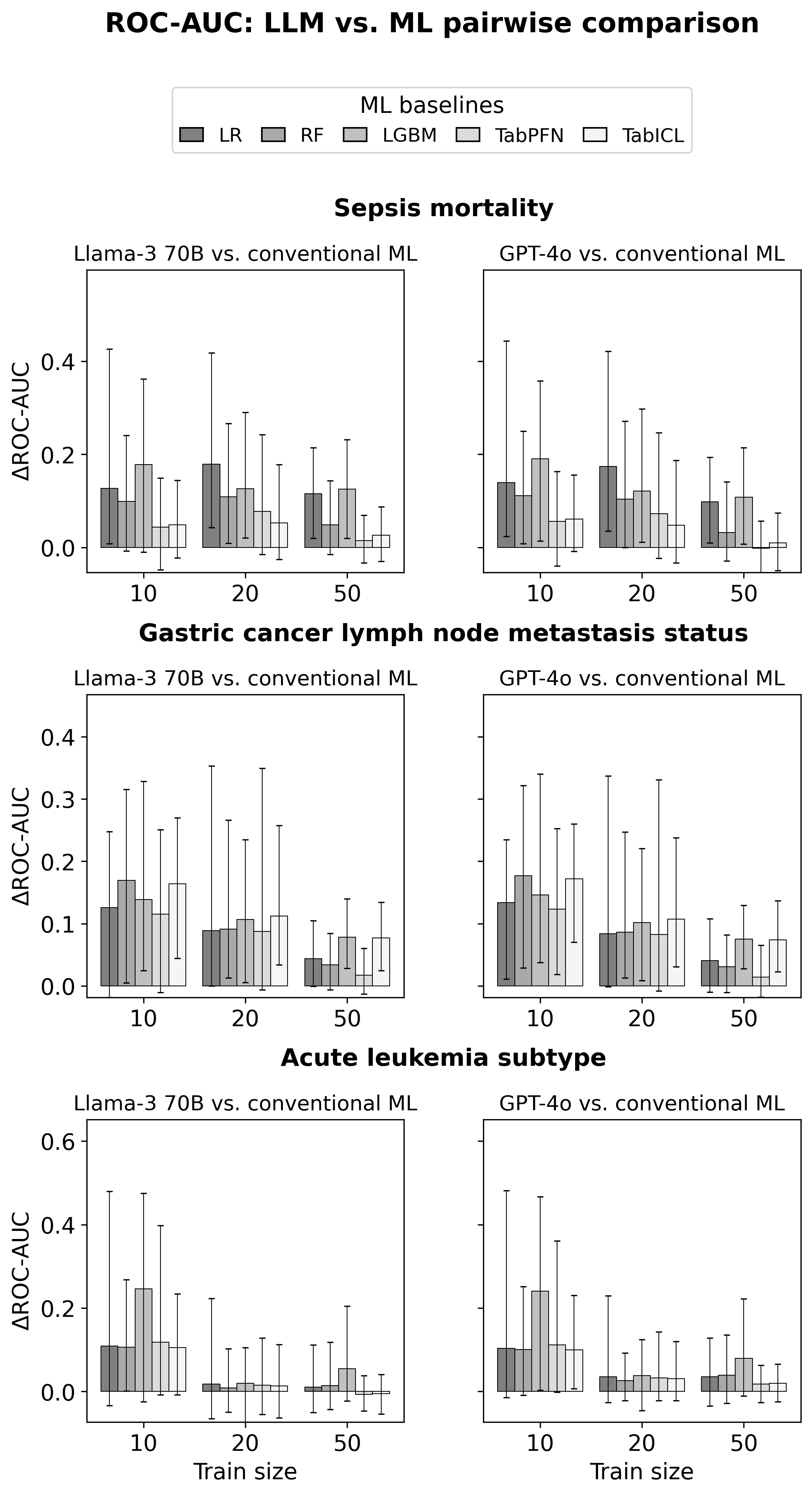


Figure S33: Difference in ROC-AUC between LLM and ML (point value – mean across folds – and 95% prediction interval), for the sepsis (top), gastric cancer (middle), and leukemia (bottom) datasets.


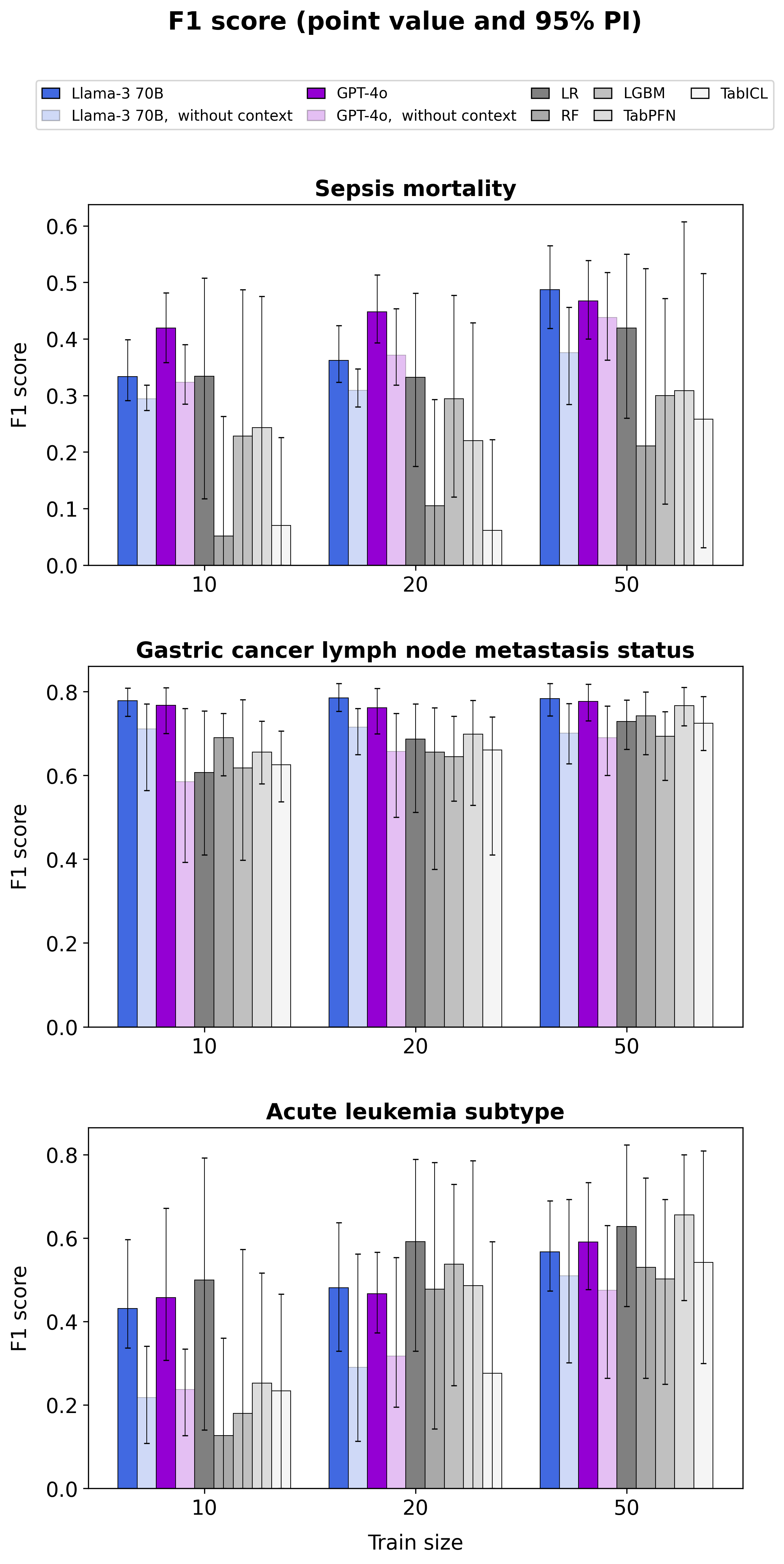


Figure S34: Point value (mean across folds) and 95% prediction interval for the F1 score, using LLMs with or without context, as well as using conventional ML, for the sepsis (top), gastric cancer (middle), and leukemia (bottom) datasets.


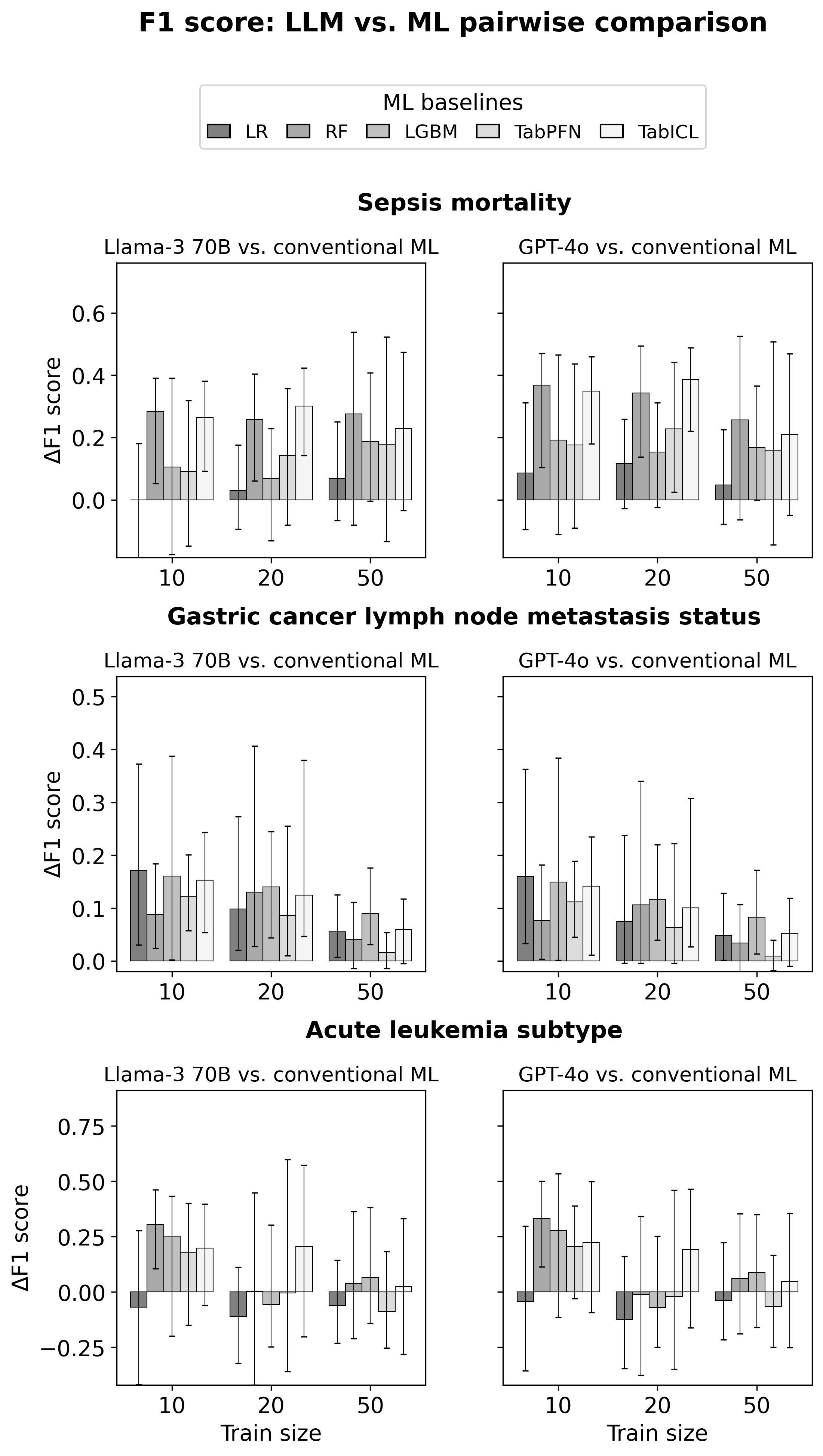


Figure S35: Difference in F1 score between LLM and ML (point value – mean across folds – and 95% prediction interval), for the sepsis (top), gastric cancer (middle), and leukemia (bottom) datasets.


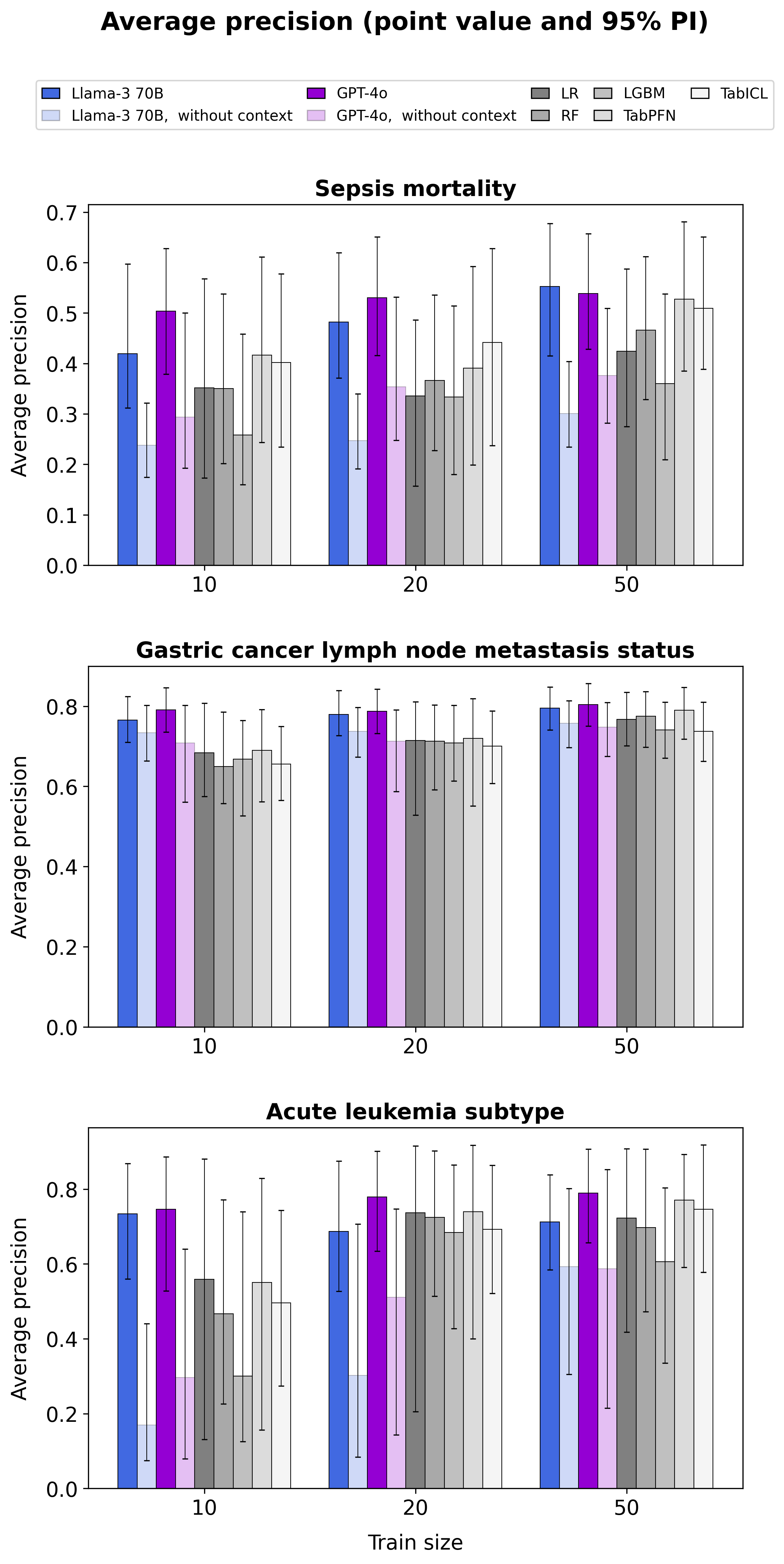


Figure S36: Point value (mean across folds) and 95% prediction interval for the Average Precision (AP), using LLMs with or without context, as well as using conventional ML, for the sepsis (top), gastric cancer (middle), and leukemia (bottom) datasets.


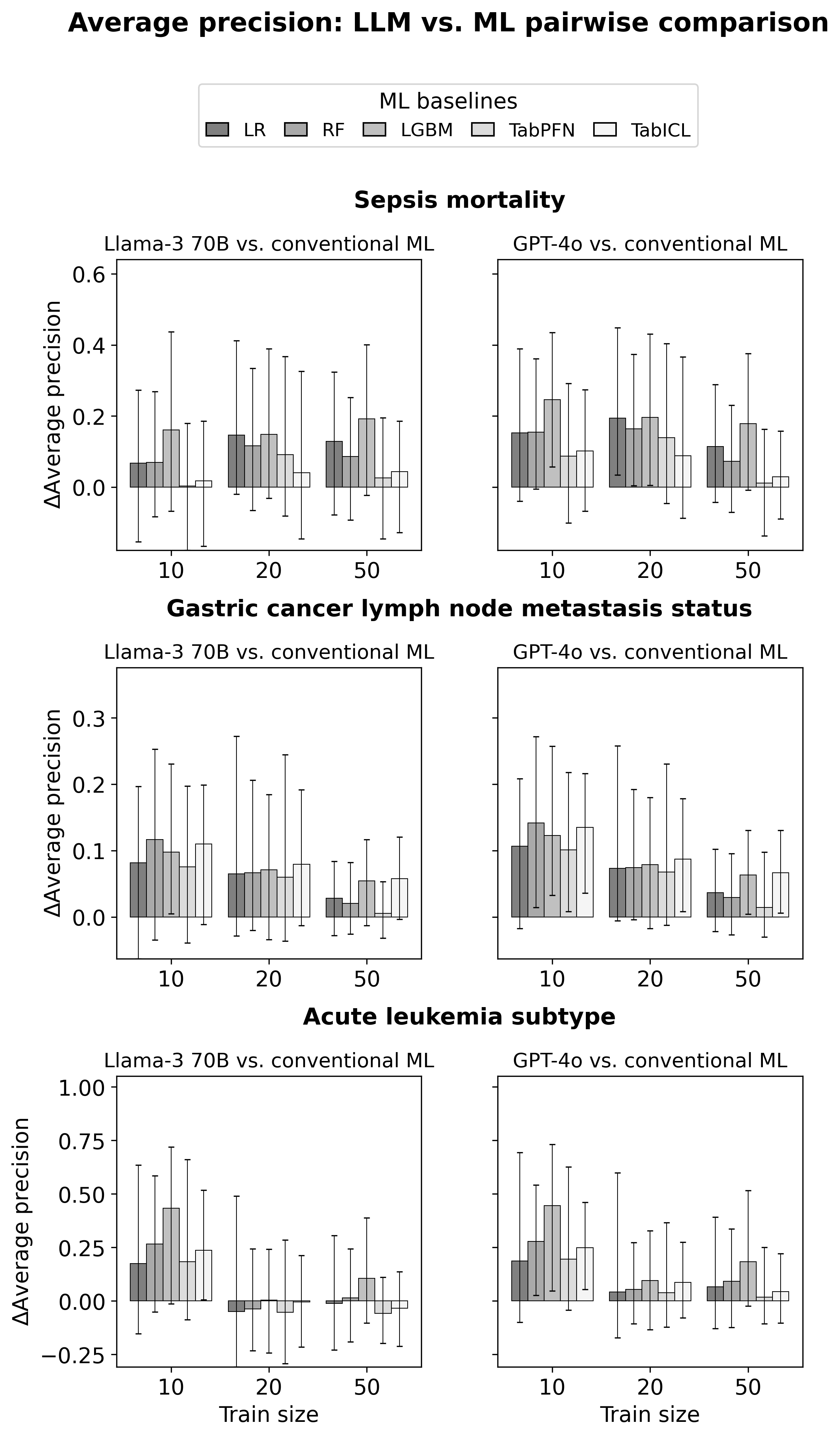


Figure S37: Difference in Average Precision (AP) between LLM and ML (point value – mean across folds – and 95% prediction interval), for the sepsis (top), gastric cancer (middle), and leukemia (bottom) datasets.


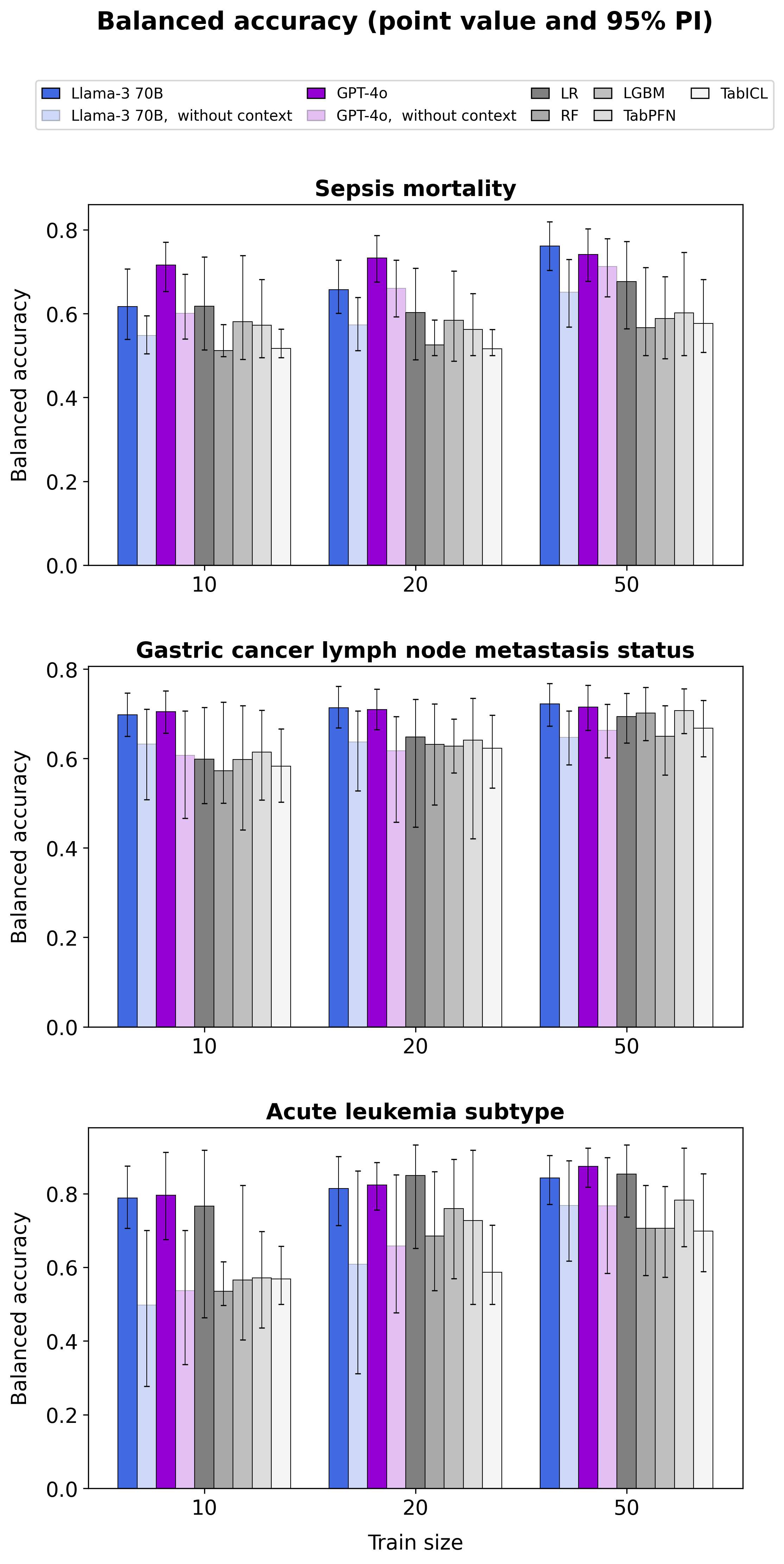


Figure S38: Point value (mean across folds) and 95% prediction interval for the balanced accuracy, using LLMs with or without context, as well as using conventional ML, for the sepsis (top), gastric cancer (middle), and leukemia (bottom) datasets.


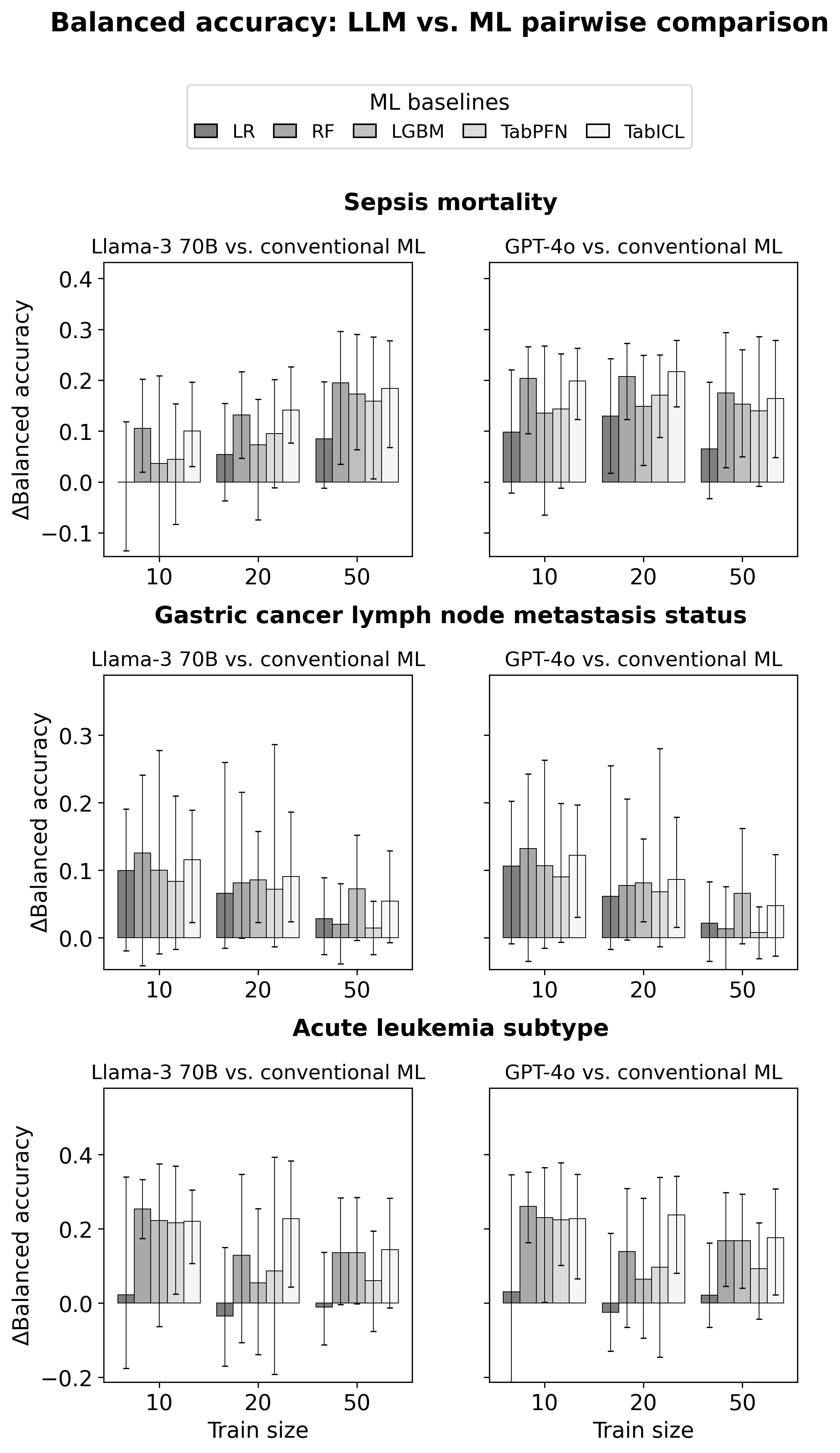


Figure S39: Difference in balanced accuracy between LLM and ML (point value – mean across folds – and 95% prediction interval), for the sepsis (top), gastric cancer (middle), and leukemia (bottom) datasets.


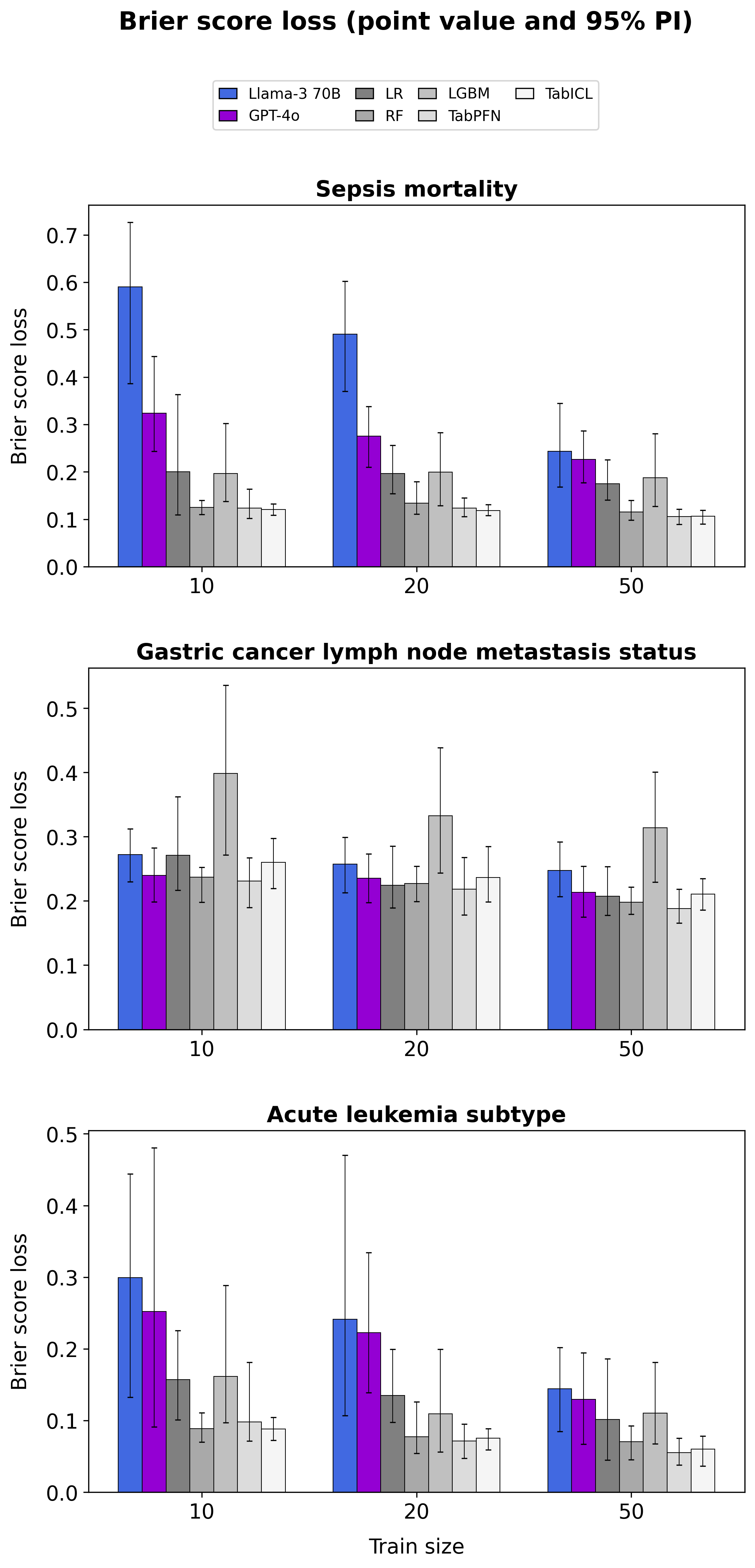


Figure S40: Point value (mean across folds) and 95% prediction interval for the Brier score loss, using LLMs (with context), as well as using conventional ML, for the sepsis (top), gastric cancer (middle), and leukemia (bottom) datasets.


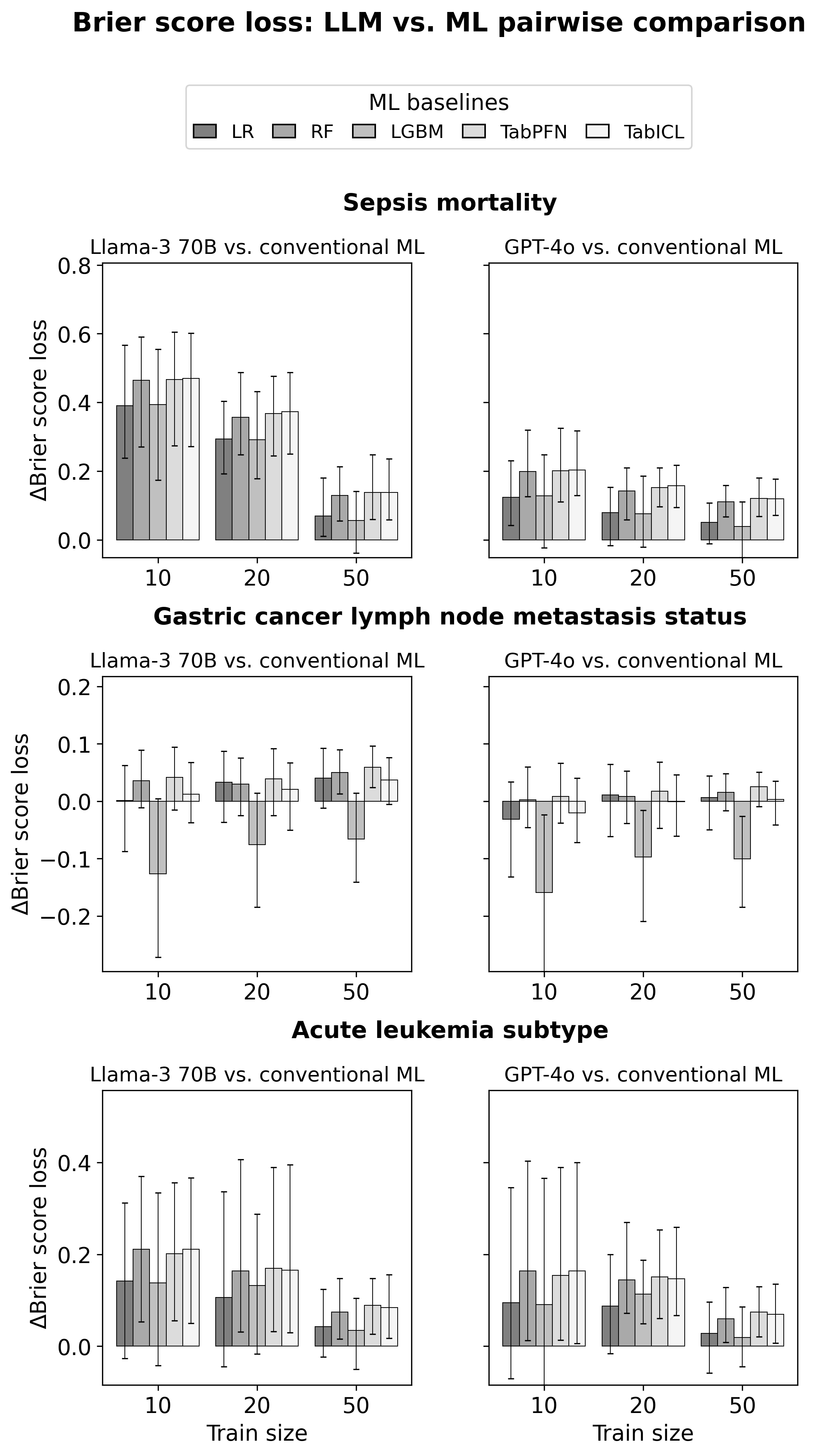


Figure S41: Difference in Brier score loss between LLM and ML (point value – mean across folds – and 95% prediction interval), for the sepsis (top), gastric cancer (middle), and leukemia (bottom) datasets.


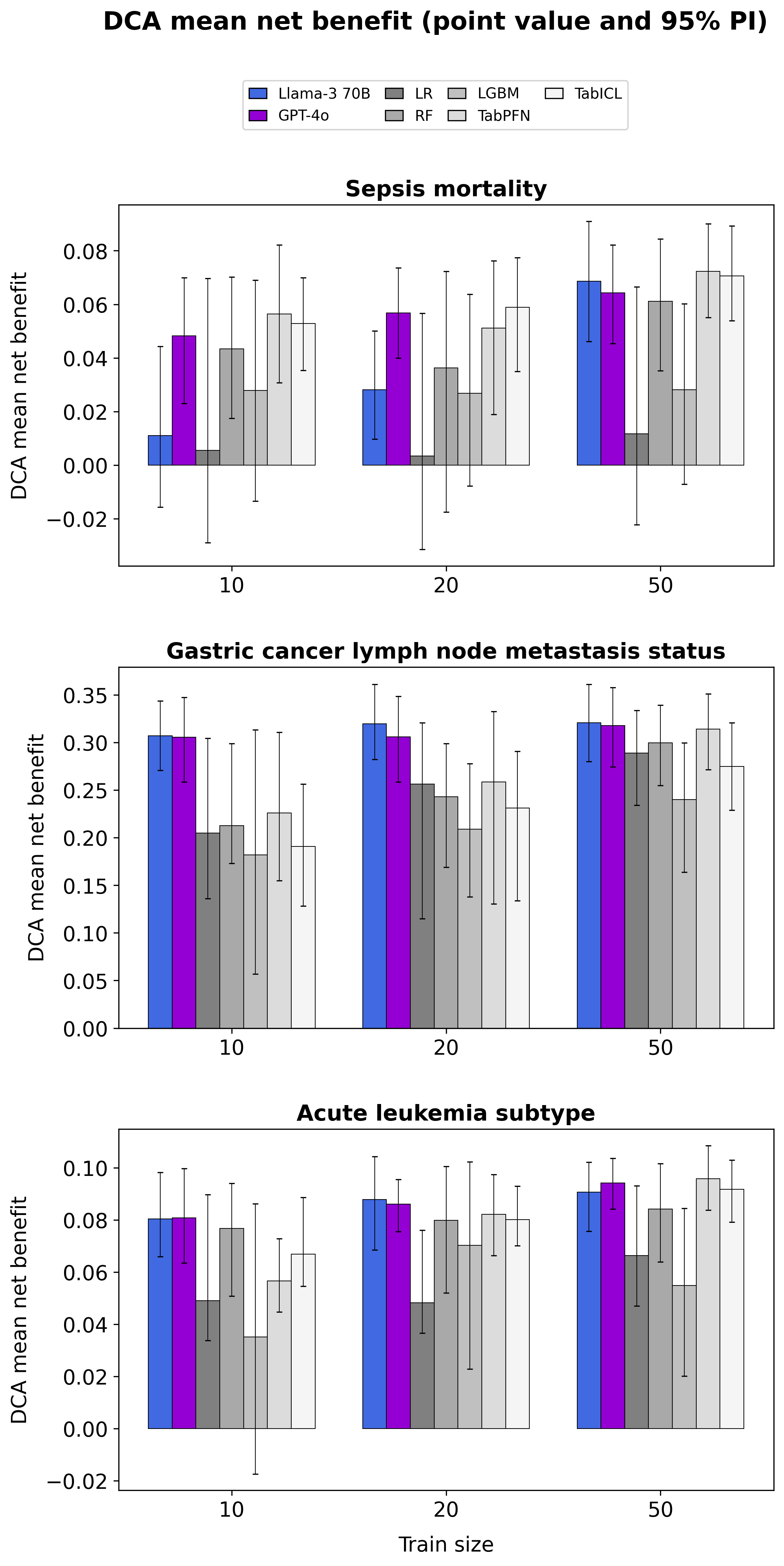


Figure S42: Point value (mean across folds) and 95% prediction interval for the Decision Curve Analysis (DCA) mean net benefit (across the clinically relevant thresholds), using LLMs (with context), as well as using conventional ML, for the sepsis (top), gastric cancer (middle), and leukemia (bottom) datasets.


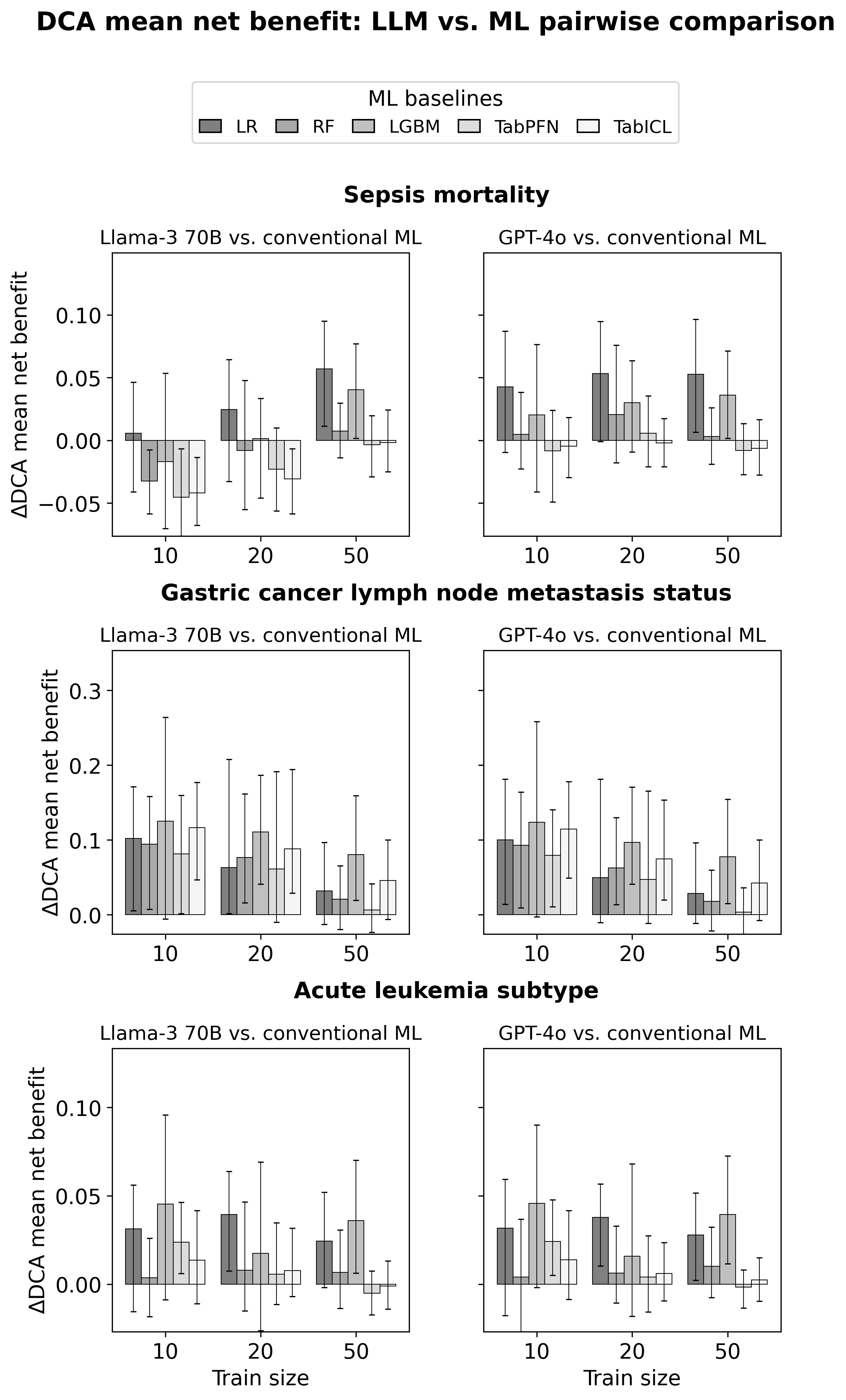


Figure S43: Difference in Decision Curve Analysis (DCA) mean net benefit (across the clinically relevant thresholds) between LLM and ML (point value – mean across folds – and 95% prediction interval), for the sepsis (top), gastric cancer (middle), and leukemia (bottom) datasets.

Prediction intervals

With LLM beta calibration


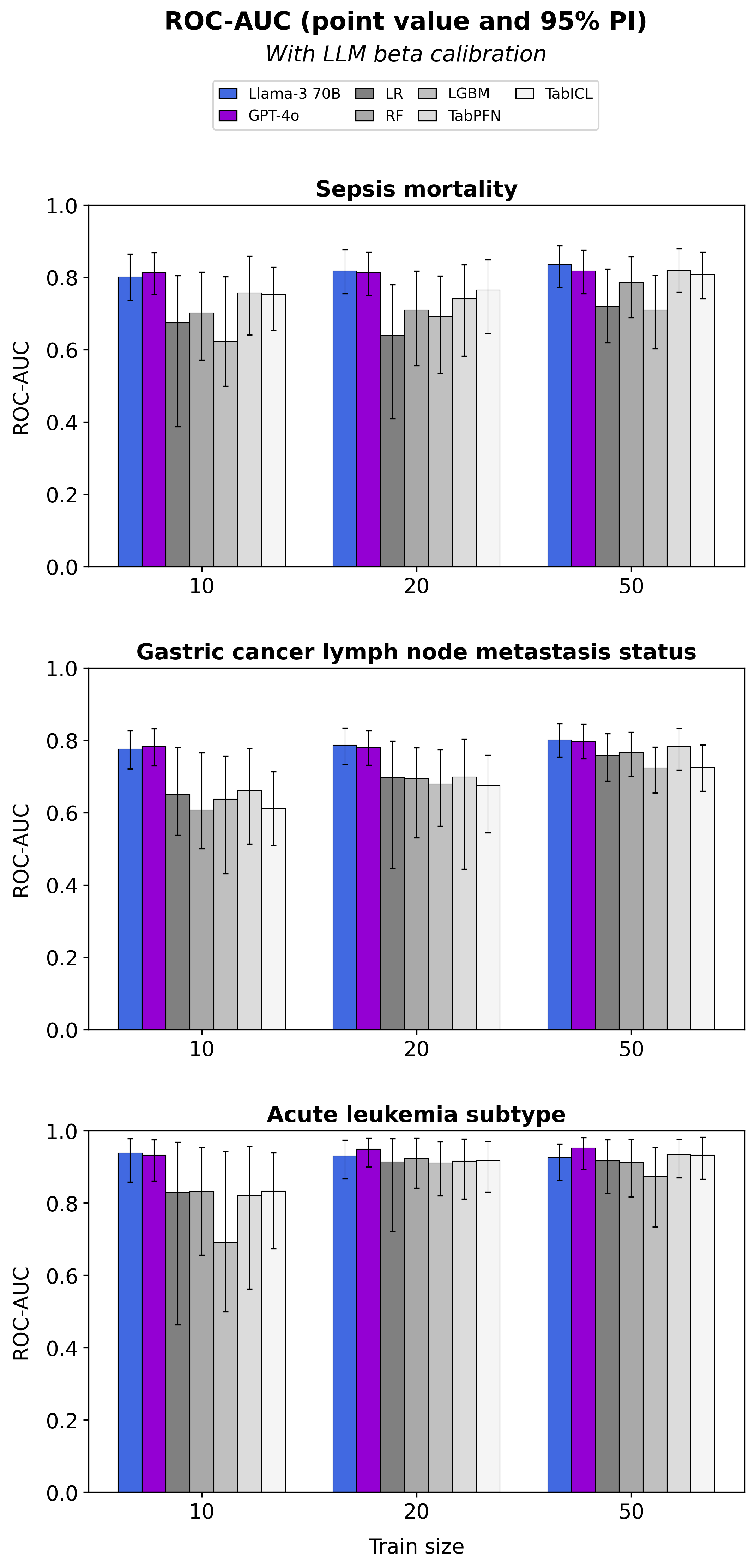


Figure S44: Point value (mean across folds) and 95% prediction interval for the ROC-AUC, using LLMs with or without context, as well as using conventional ML, for the sepsis (top), gastric cancer (middle), and leukemia (bottom) datasets.


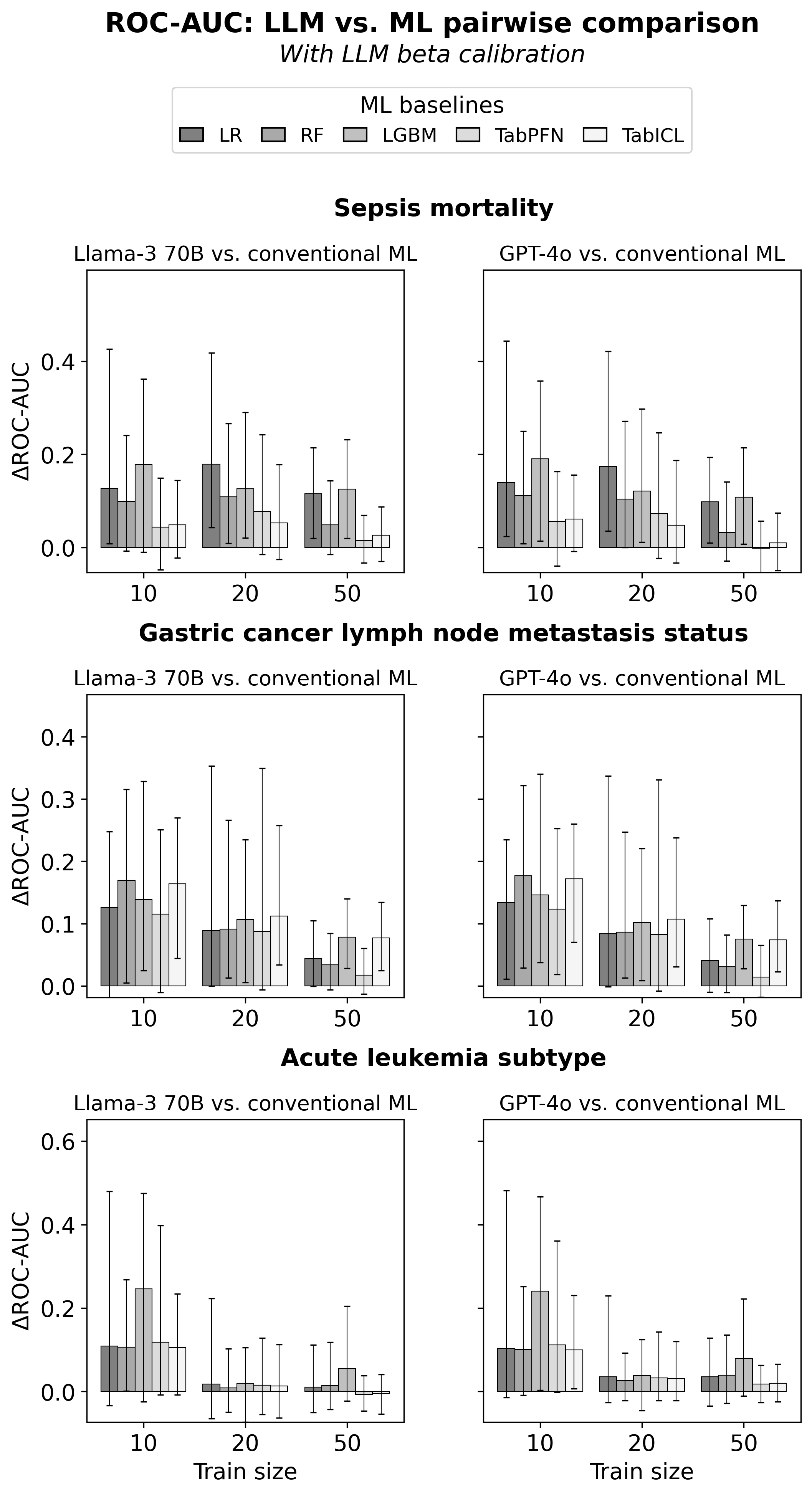


Figure S45: Difference in ROC-AUC between LLM and ML (point value – mean across folds – and 95% prediction interval), for the sepsis (top), gastric cancer (middle), and leukemia (bottom) datasets.


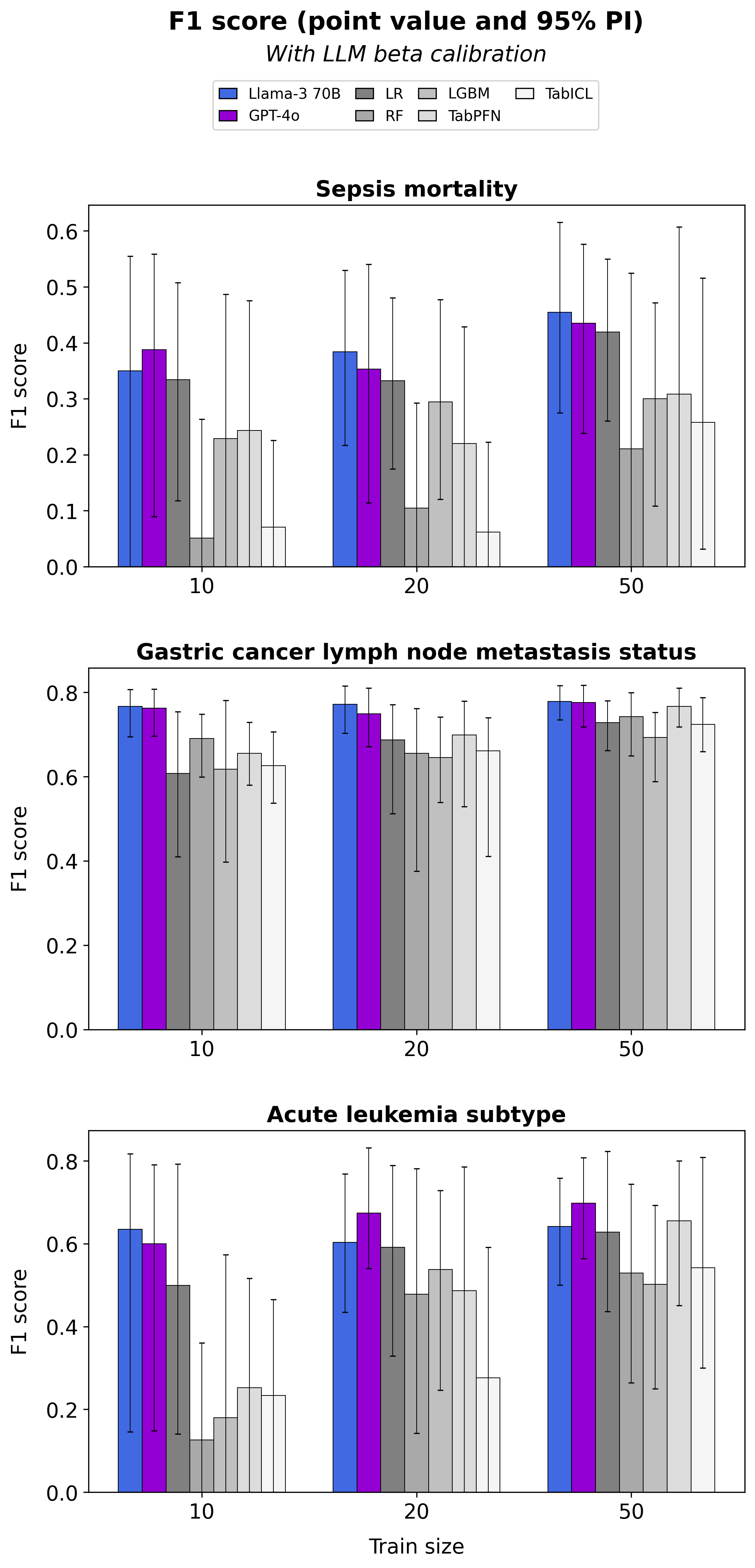


Figure S46: Point value (mean across folds) and 95% prediction interval for the F1 score, using LLMs with or without context, as well as using conventional ML, for the sepsis (top), gastric cancer (middle), and leukemia (bottom) datasets.


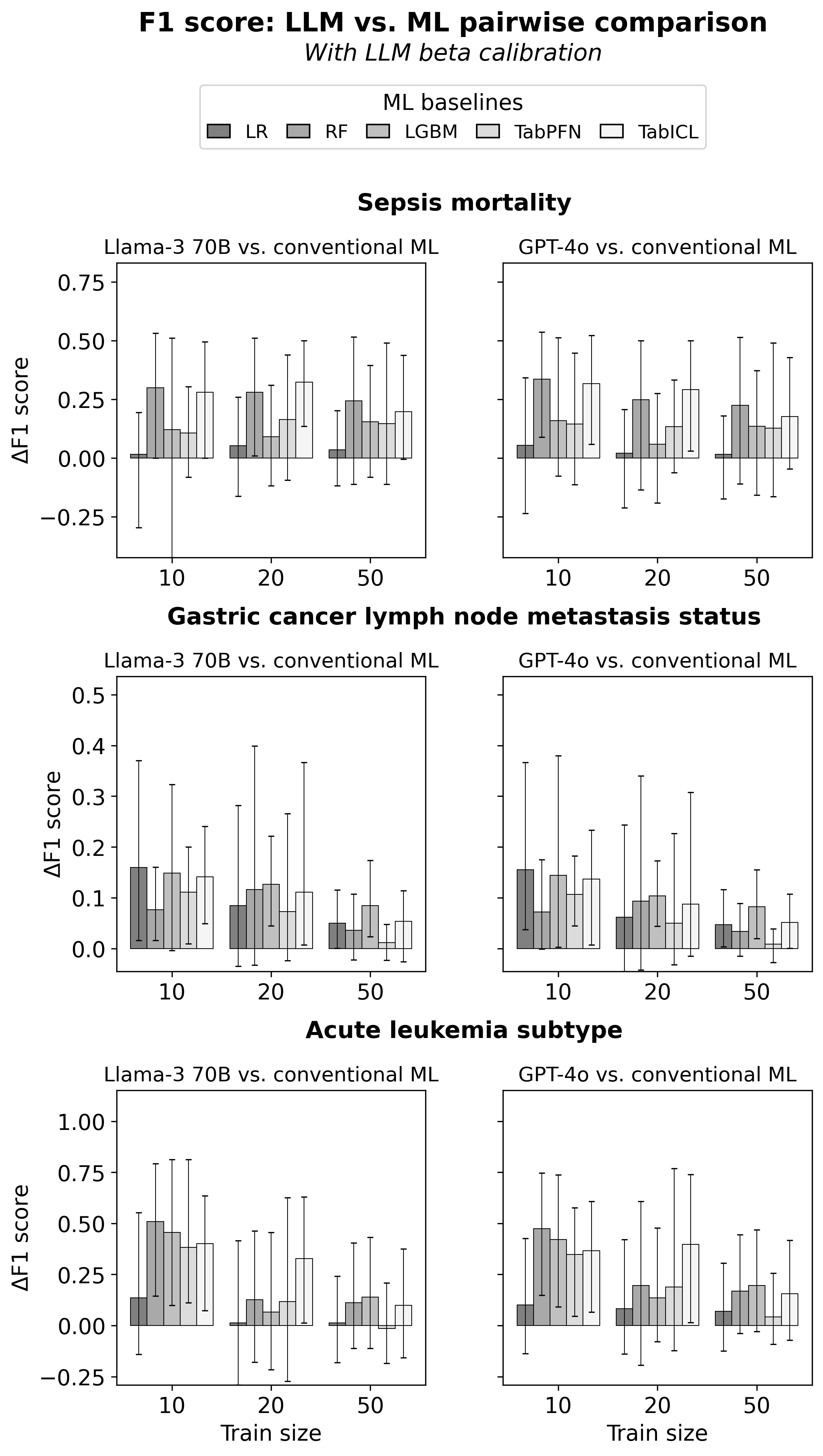


Figure S47: Difference in F1 score between LLM and ML (point value – mean across folds – and 95% prediction interval), for the sepsis (top), gastric cancer (middle), and leukemia (bottom) datasets.


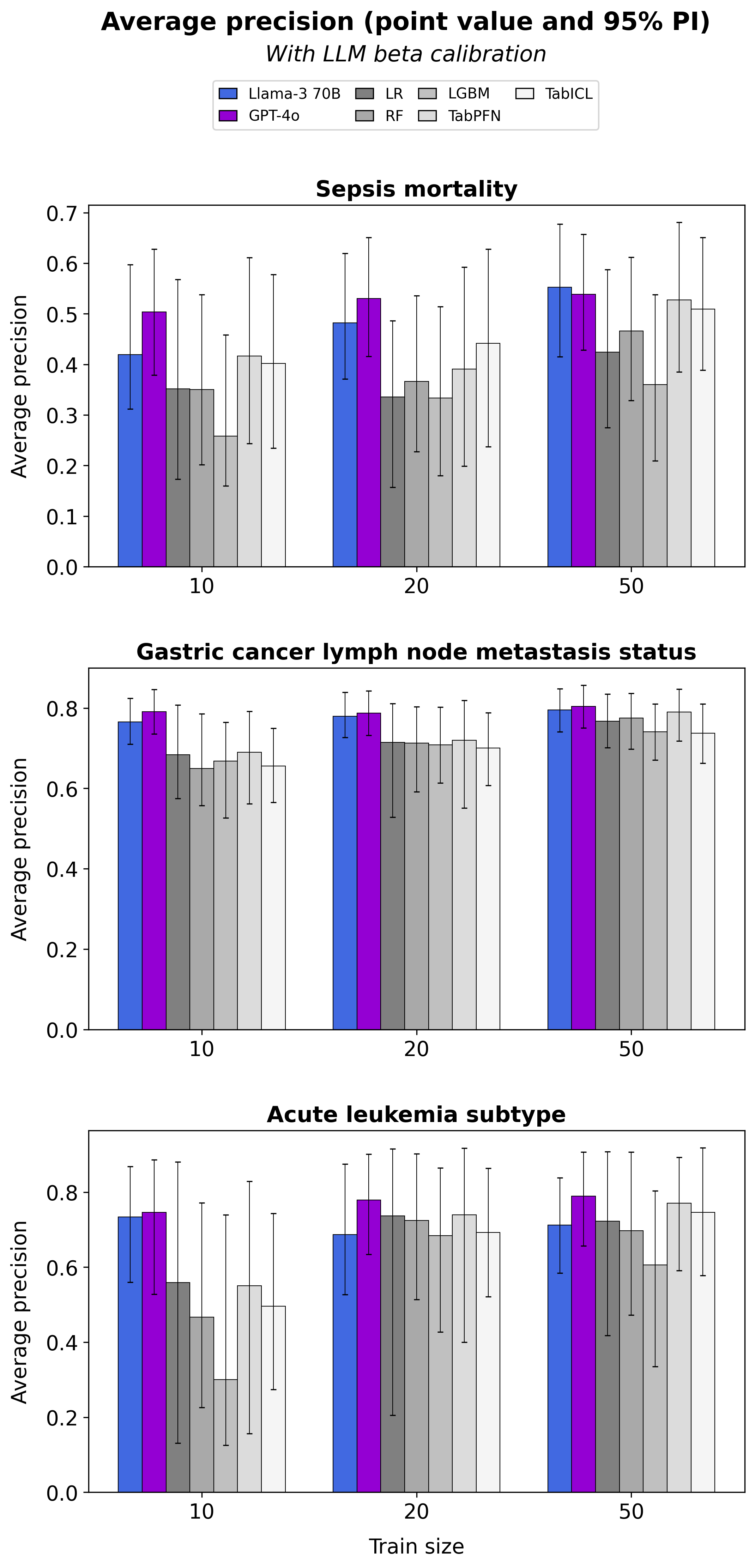


Figure S48: Point value (mean across folds) and 95% prediction interval for the Average Precision (AP), using LLMs with or without context, as well as using conventional ML, for the sepsis (top), gastric cancer (middle), and leukemia (bottom) datasets.


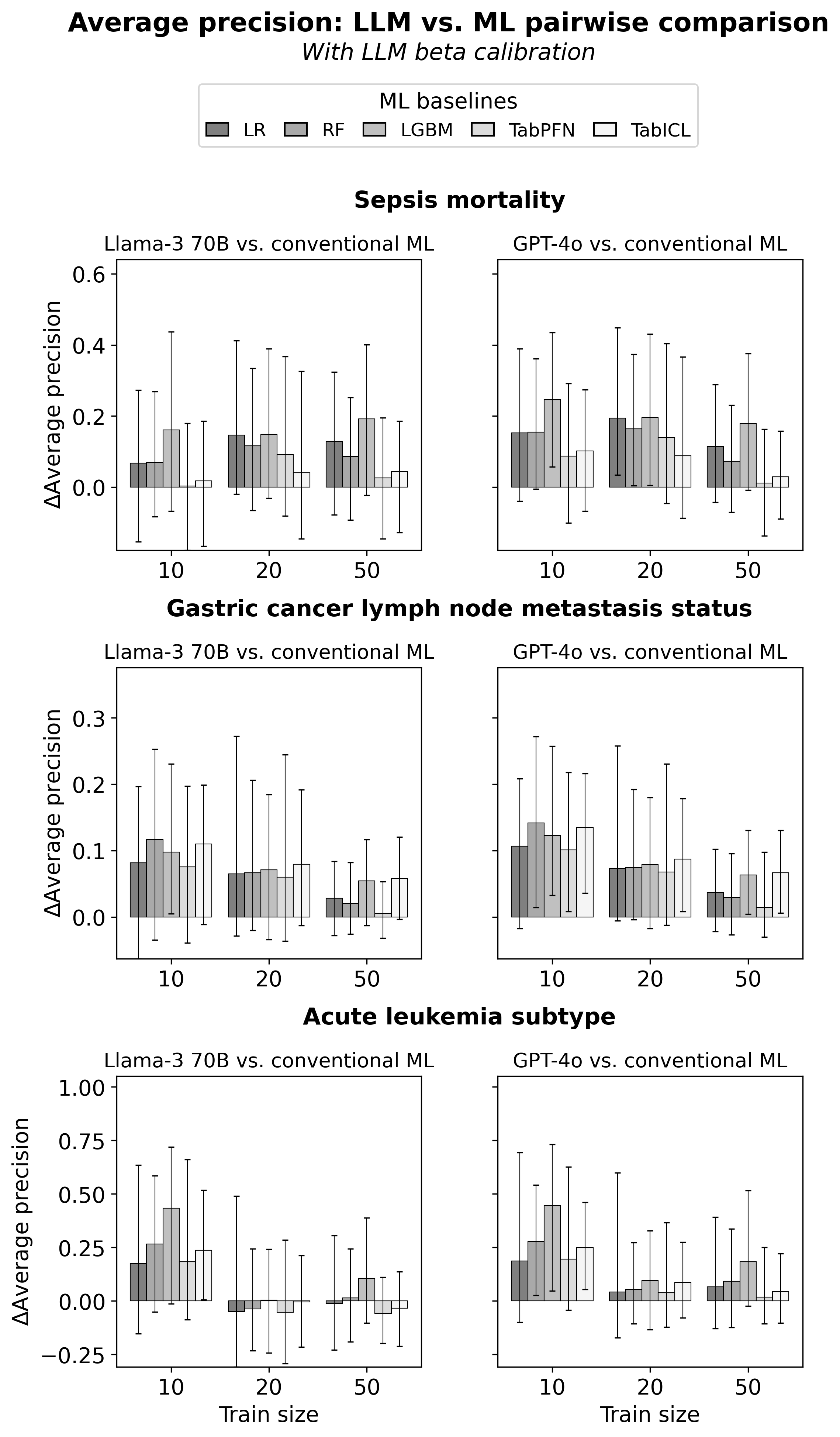


Figure S49: Difference in Average Precision (AP) between LLM and ML (point value – mean across folds – and 95% prediction interval), for the sepsis (top), gastric cancer (middle), and leukemia (bottom) datasets.


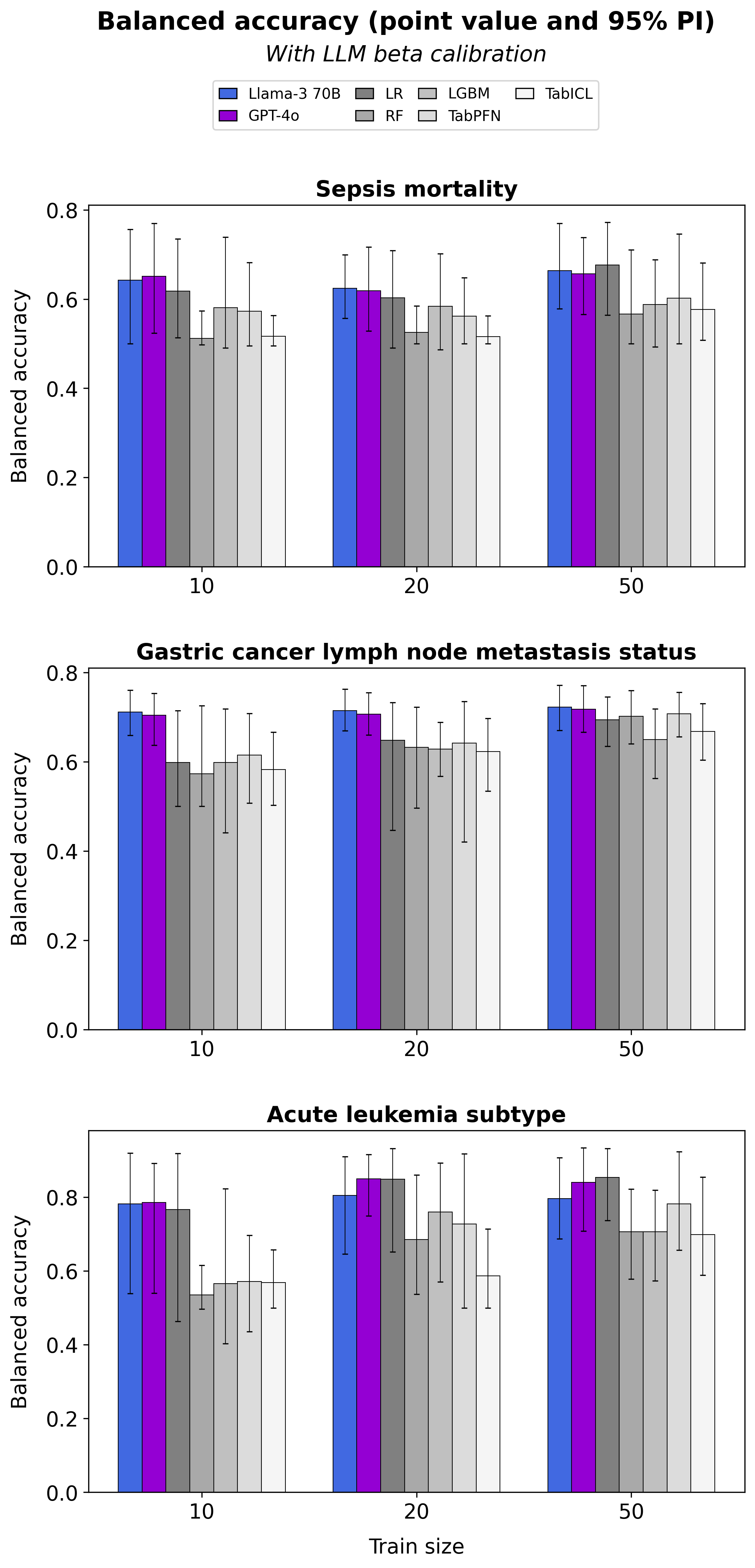


Figure S50: Point value (mean across folds) and 95% prediction interval for the balanced accuracy, using LLMs with or without context, as well as using conventional ML, for the sepsis (top), gastric cancer (middle), and leukemia (bottom) datasets.


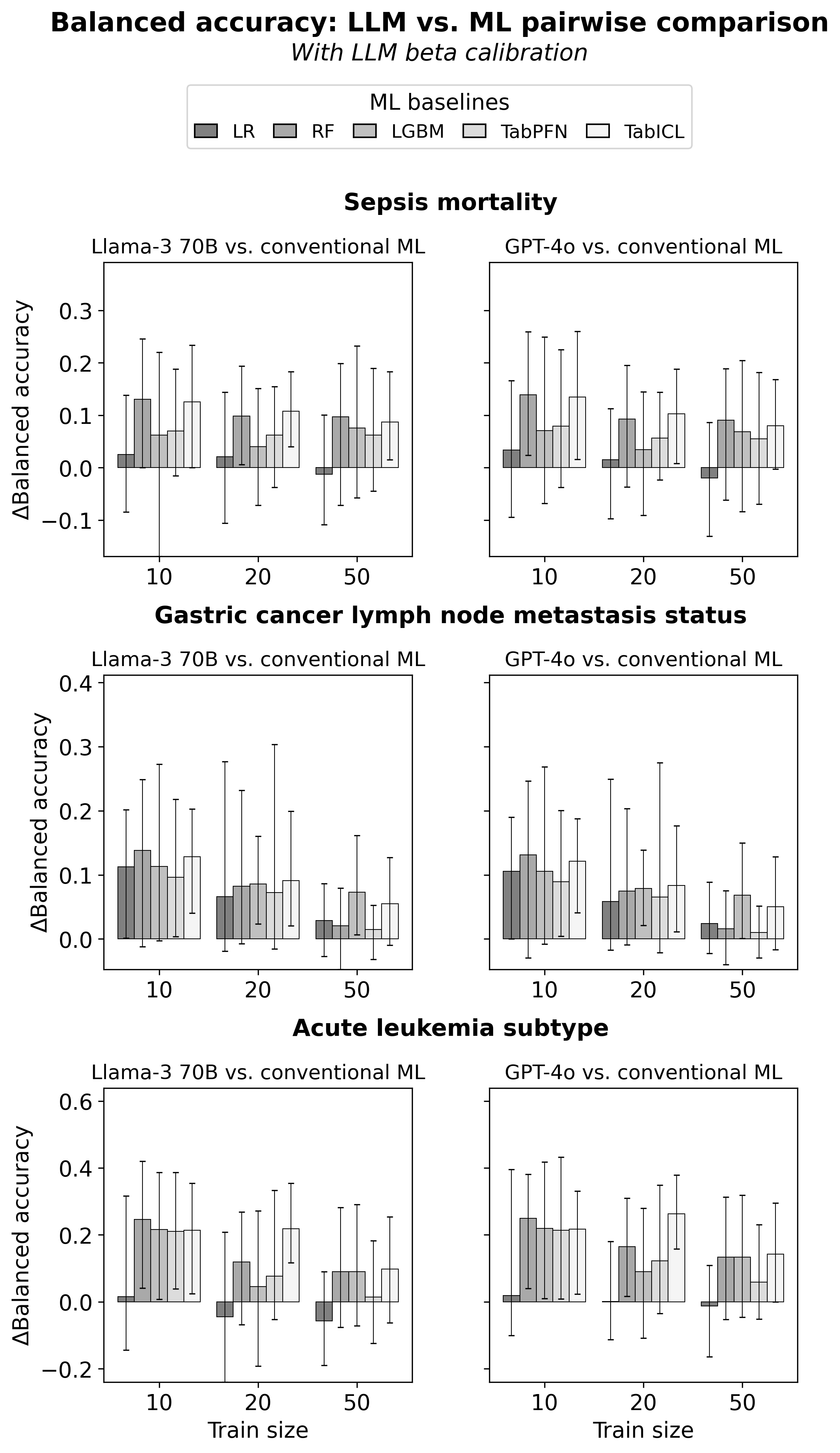


Figure S51: Difference in balanced accuracy between LLM and ML (point value – mean across folds – and 95% prediction interval), for the sepsis (top), gastric cancer (middle), and leukemia (bottom) datasets.


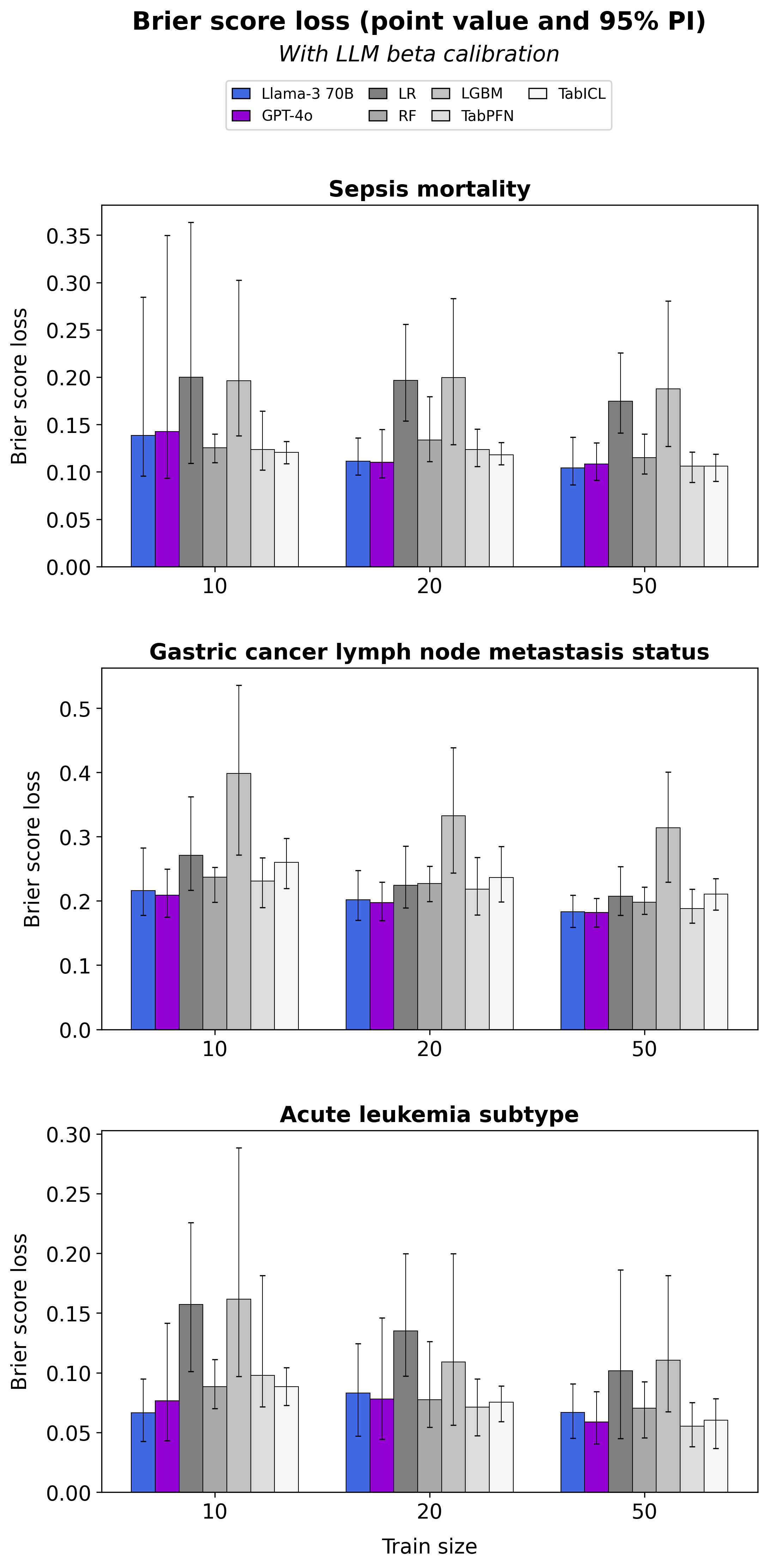


Figure S52: Point value (mean across folds) and 95% prediction interval for the Brier score loss, using LLMs (with context), as well as using conventional ML, for the sepsis (top), gastric cancer (middle), and leukemia (bottom) datasets.


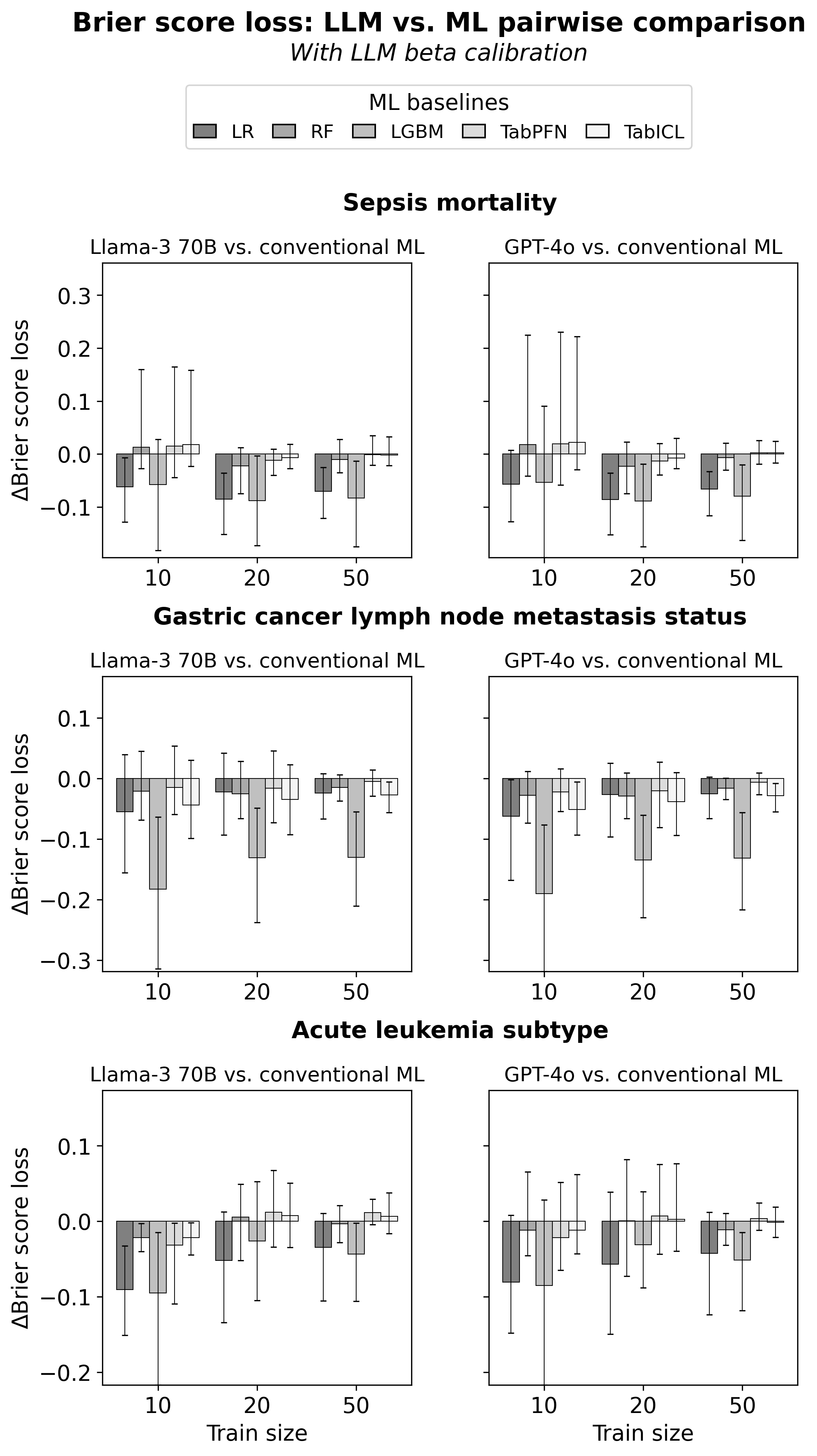


Figure S53: Difference in Brier score loss between LLM and ML (point value – mean across folds – and 95% prediction interval), for the sepsis (top), gastric cancer (middle), and leukemia (bottom) datasets.


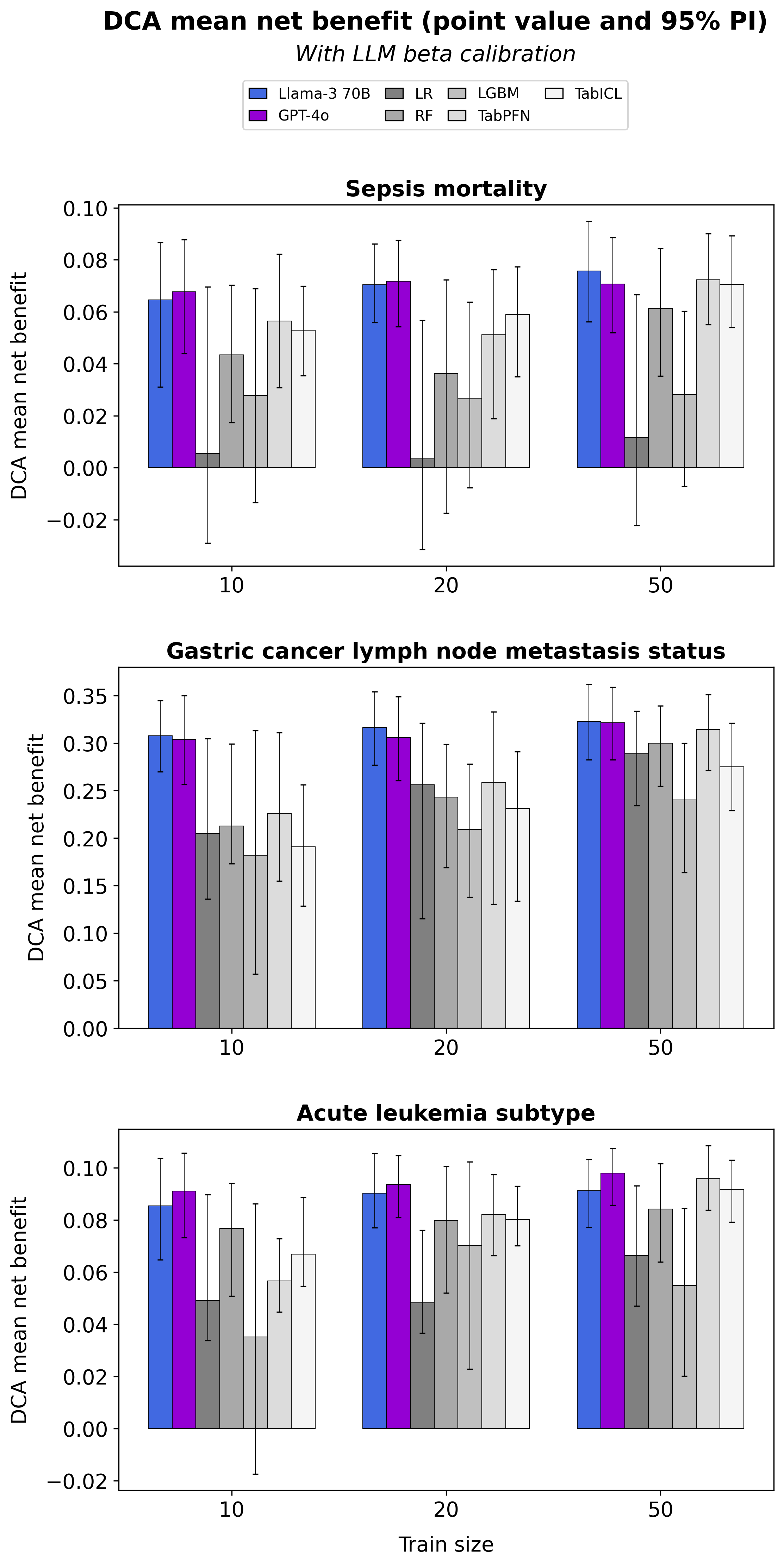


Figure S54: Point value (mean across folds) and 95% prediction interval for the Decision Curve Analysis (DCA) mean net benefit (across the clinically relevant thresholds), using LLMs (with context), as well as using conventional ML, for the sepsis (top), gastric cancer (middle), and leukemia (bottom) datasets.


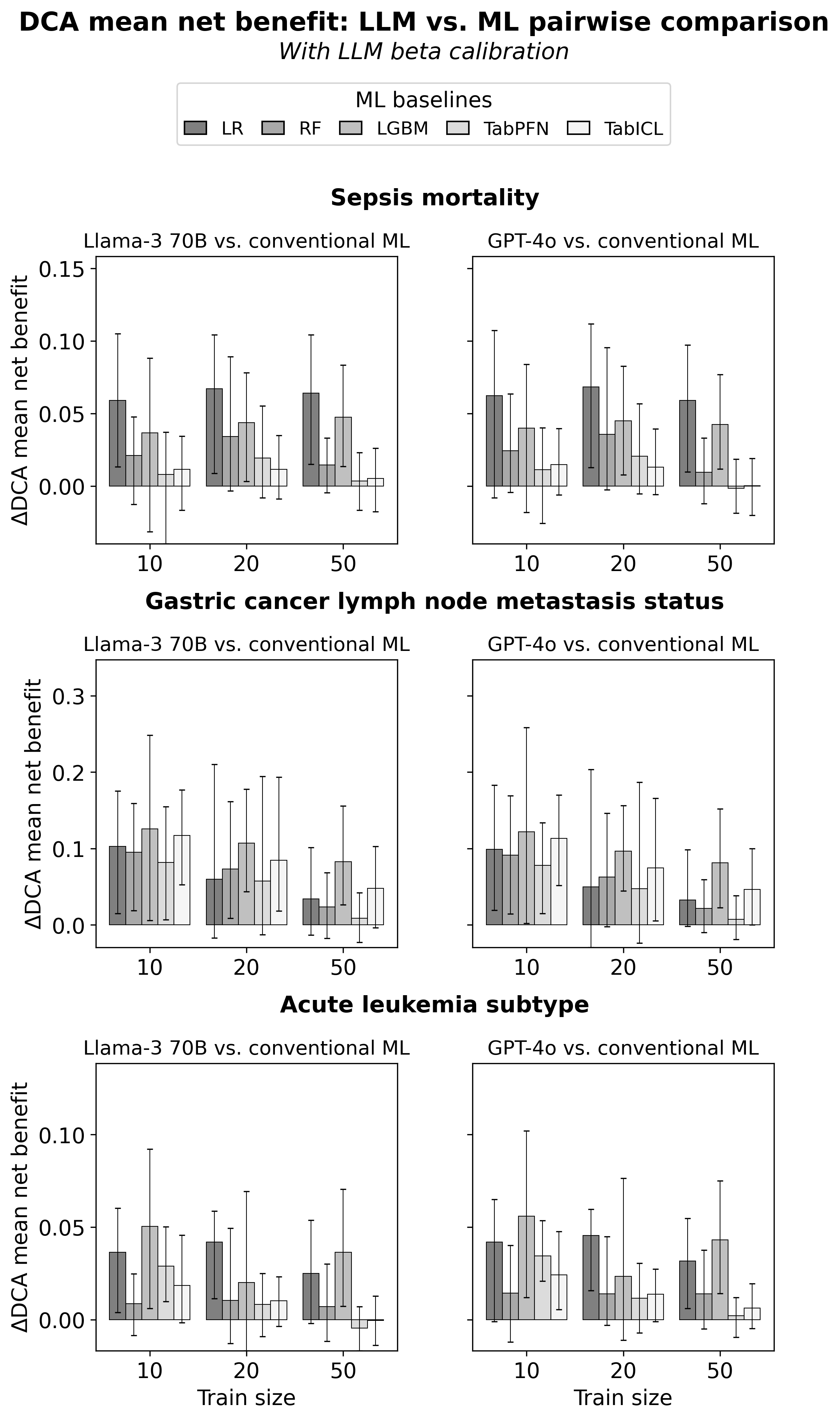


Figure S55: Difference in Decision Curve Analysis (DCA) mean net benefit (across the clinically relevant thresholds) between LLM and ML (point value – mean across folds – and 95% prediction interval), for the sepsis (top), gastric cancer (middle), and leukemia (bottom) datasets.

Calibration curves

Llama 3 70B


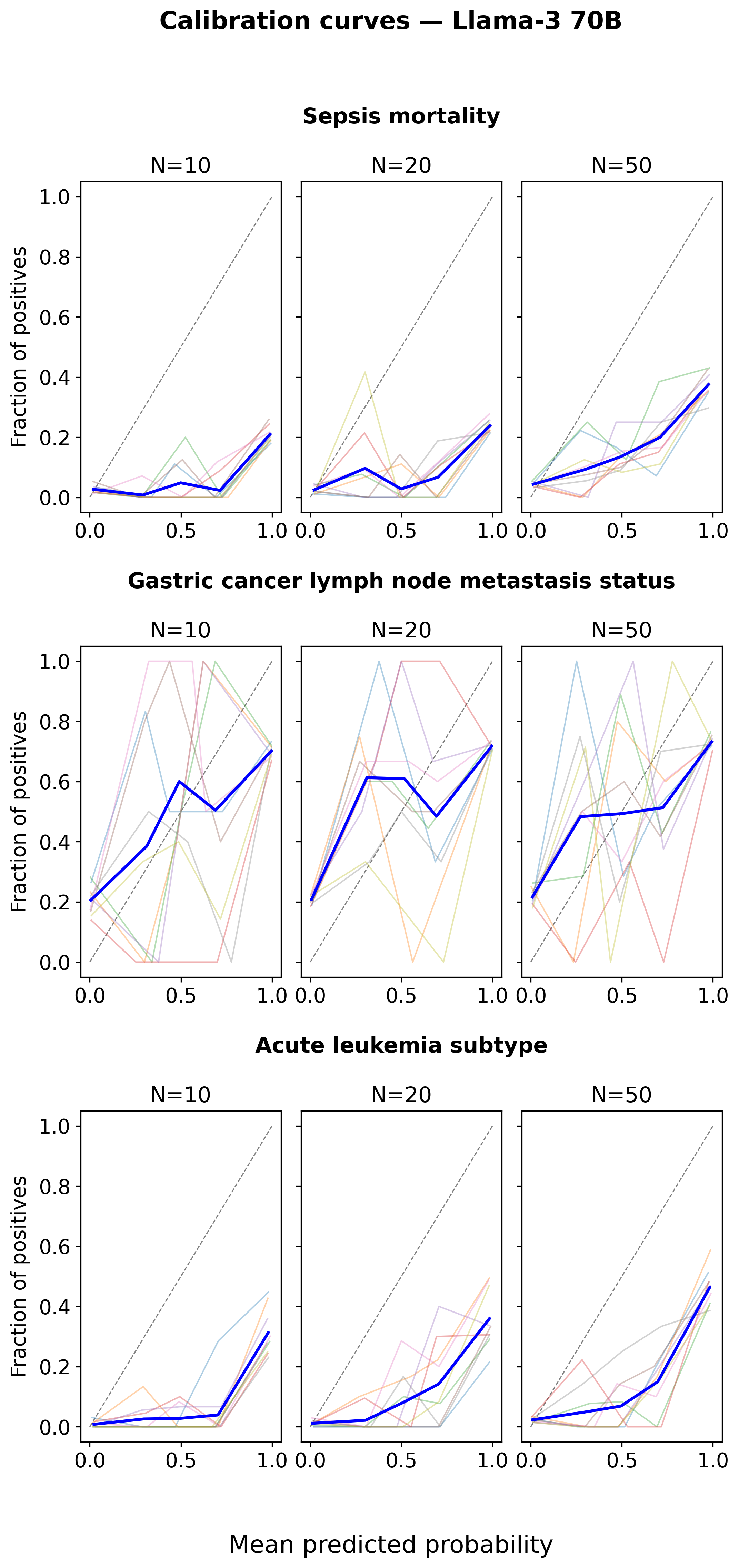


Figure S56: Calibration curves using 5 equal-width bins for Llama 3 70B without calibration, for the sepsis (top), gastric cancer (middle), and leukemia (bottom) datasets. The light-colored curves correspond to individual folds, and the blue curve is the mean curve across folds.


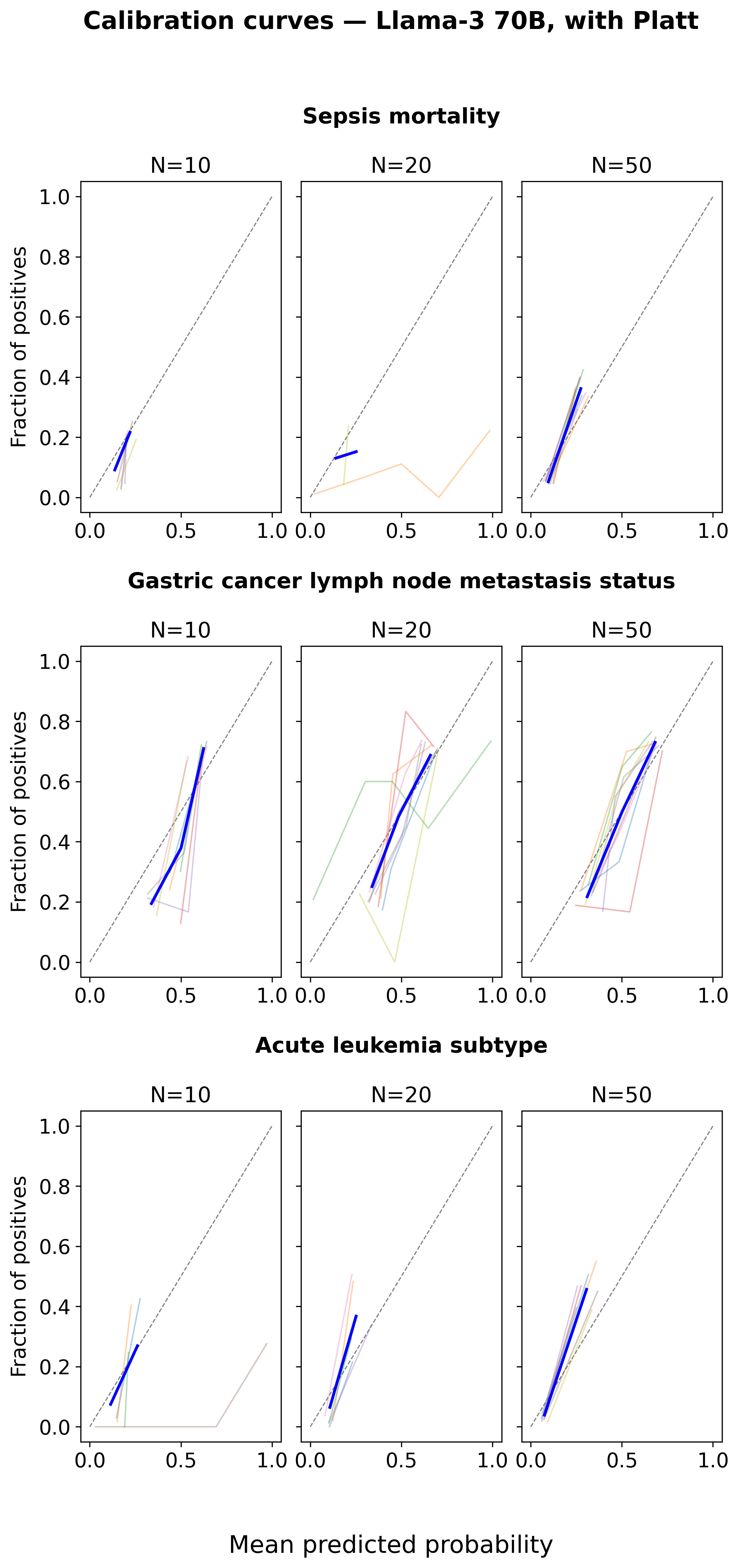


Figure S57: Calibration curves using 5 equal-width bins for Llama 3 70B with Platt calibration, for the sepsis (top), gastric cancer (middle), and leukemia (bottom) datasets. The light-colored curves correspond to individual folds, and the blue curve is the mean curve across folds.


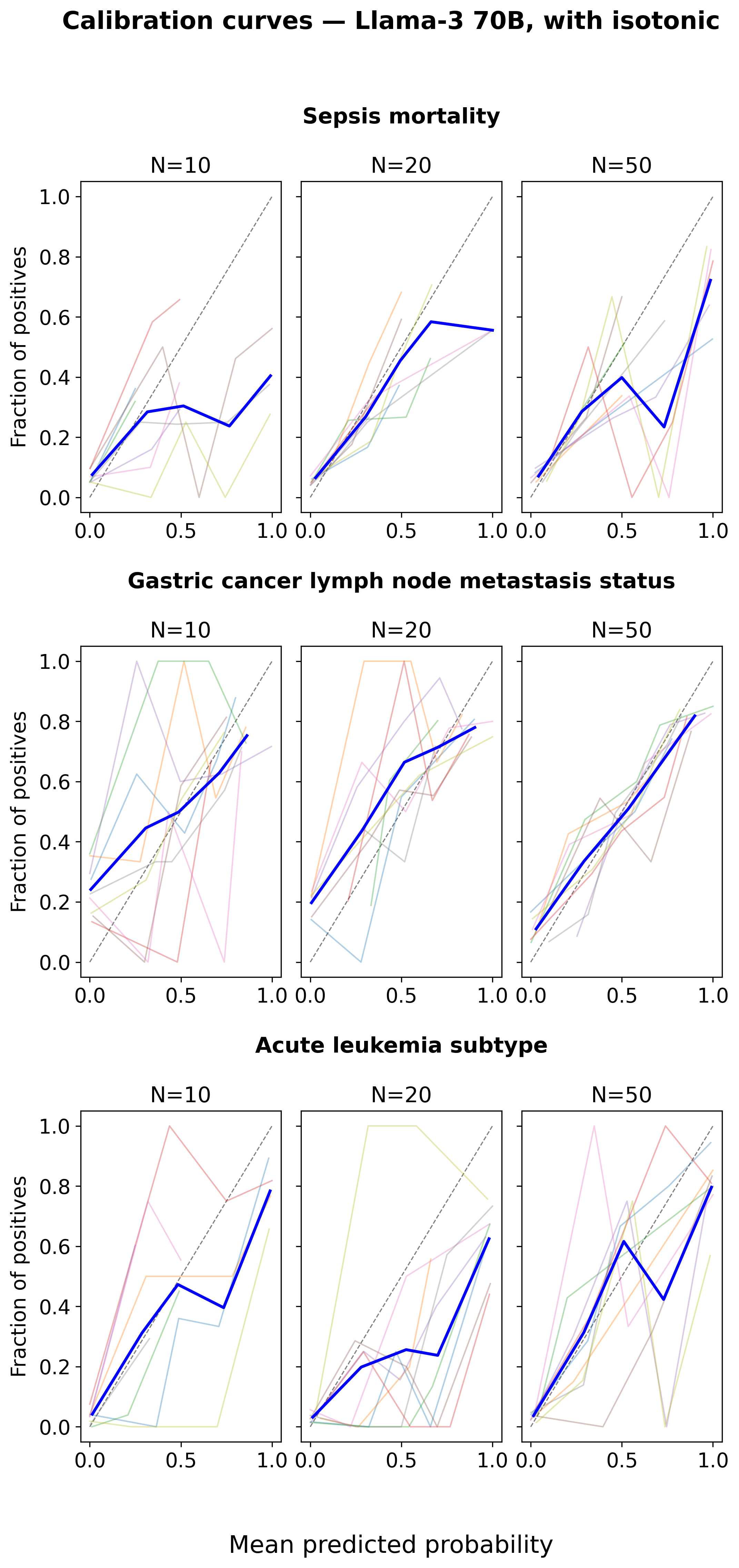


Figure S58: Calibration curves using 5 equal-width bins for Llama 3 70B with isotonic calibration, for the sepsis (top), gastric cancer (middle), and leukemia (bottom) datasets. The light-colored curves correspond to individual folds, and the blue curve is the mean curve across folds.


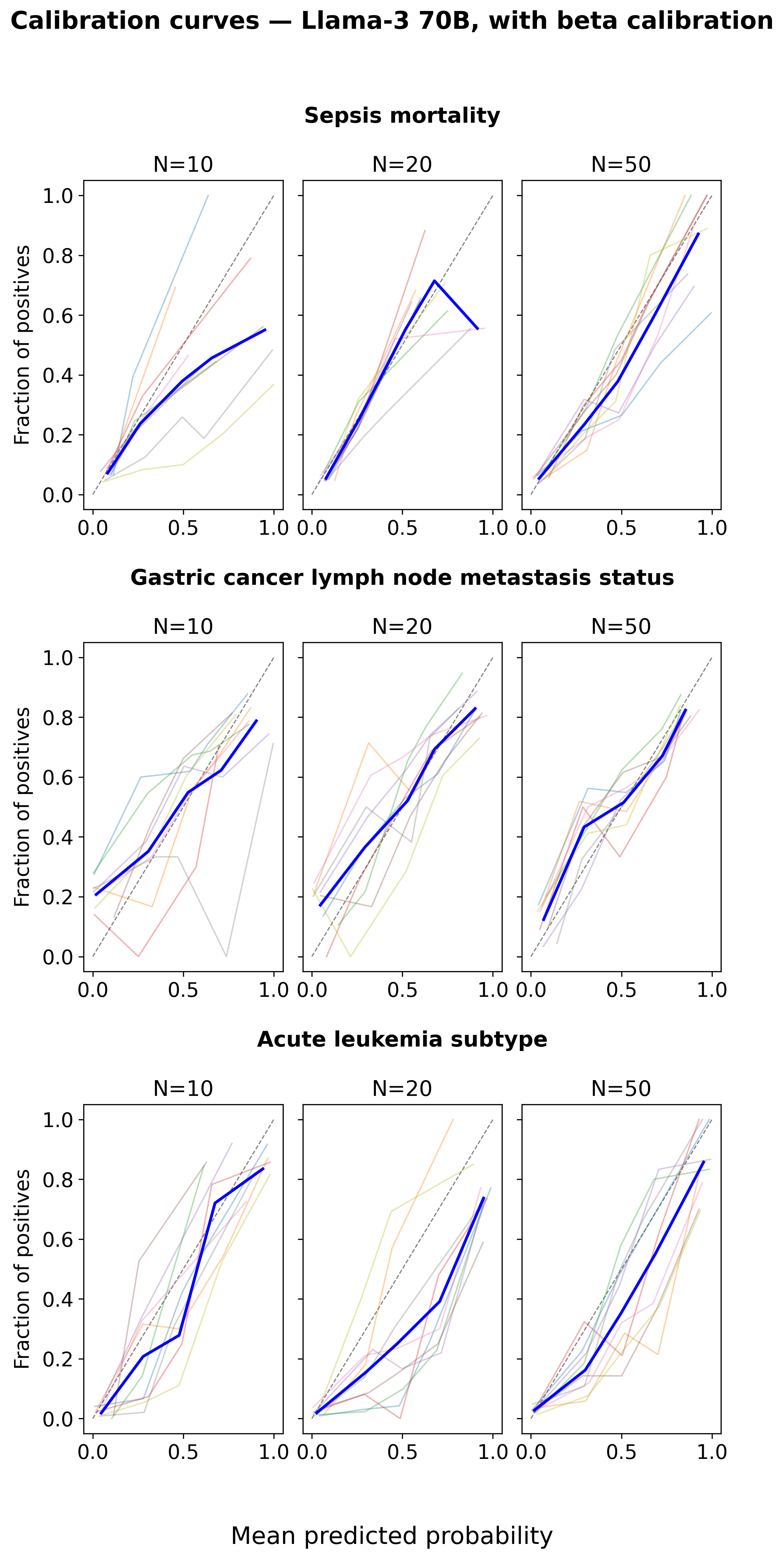


Figure S59: Calibration curves using 5 equal-width bins for Llama 3 70B with beta calibration, for the sepsis (top), gastric cancer (middle), and leukemia (bottom) datasets. The light-colored curves correspond to individual folds, and the blue curve is the mean curve across folds.

Calibration curves

GPT-4o


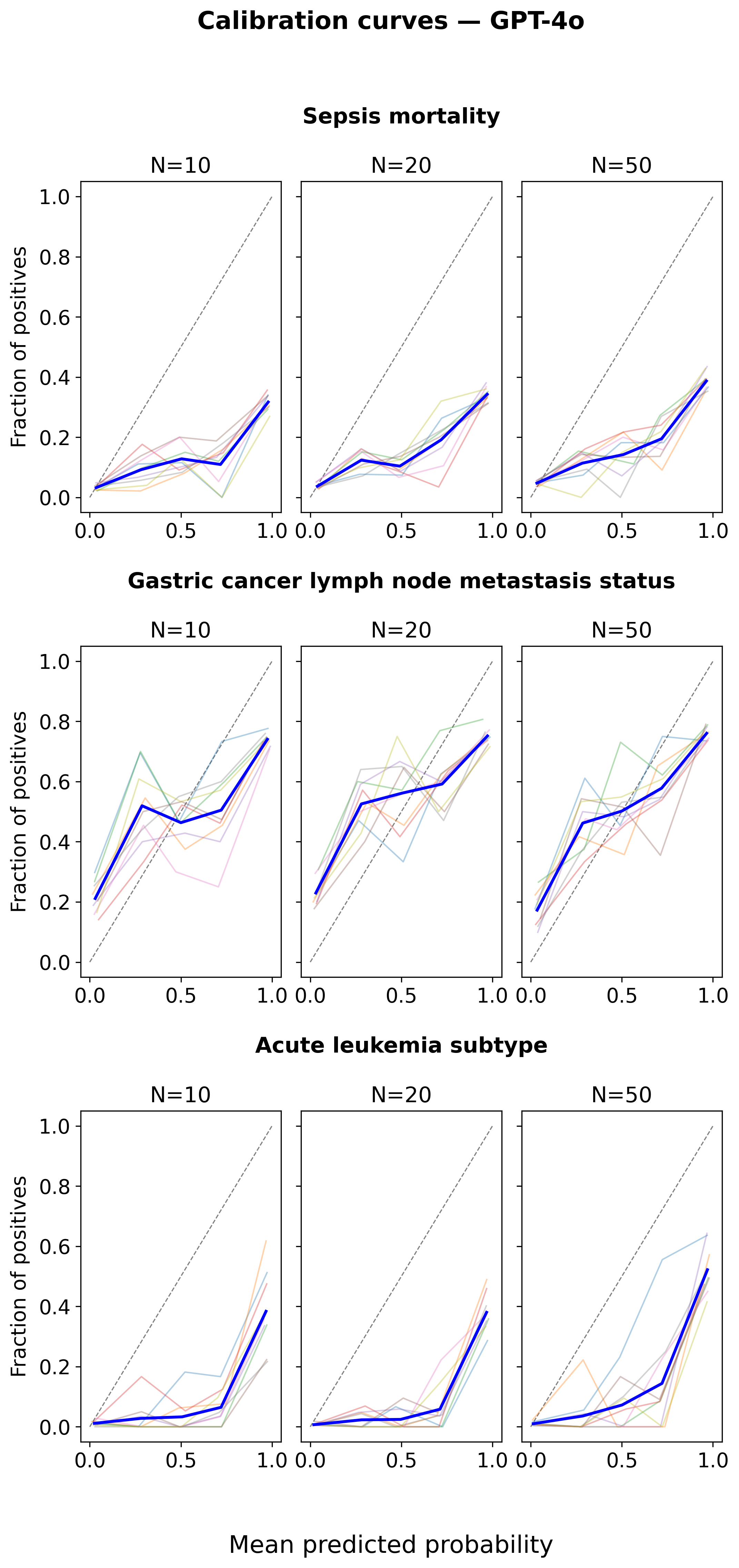


Figure S60: Calibration curves using 5 equal-width bins for GPT-4o without calibration, for the sepsis (top), gastric cancer (middle), and leukemia (bottom) datasets. The light-colored curves correspond to individual folds, and the blue curve is the mean curve across folds.


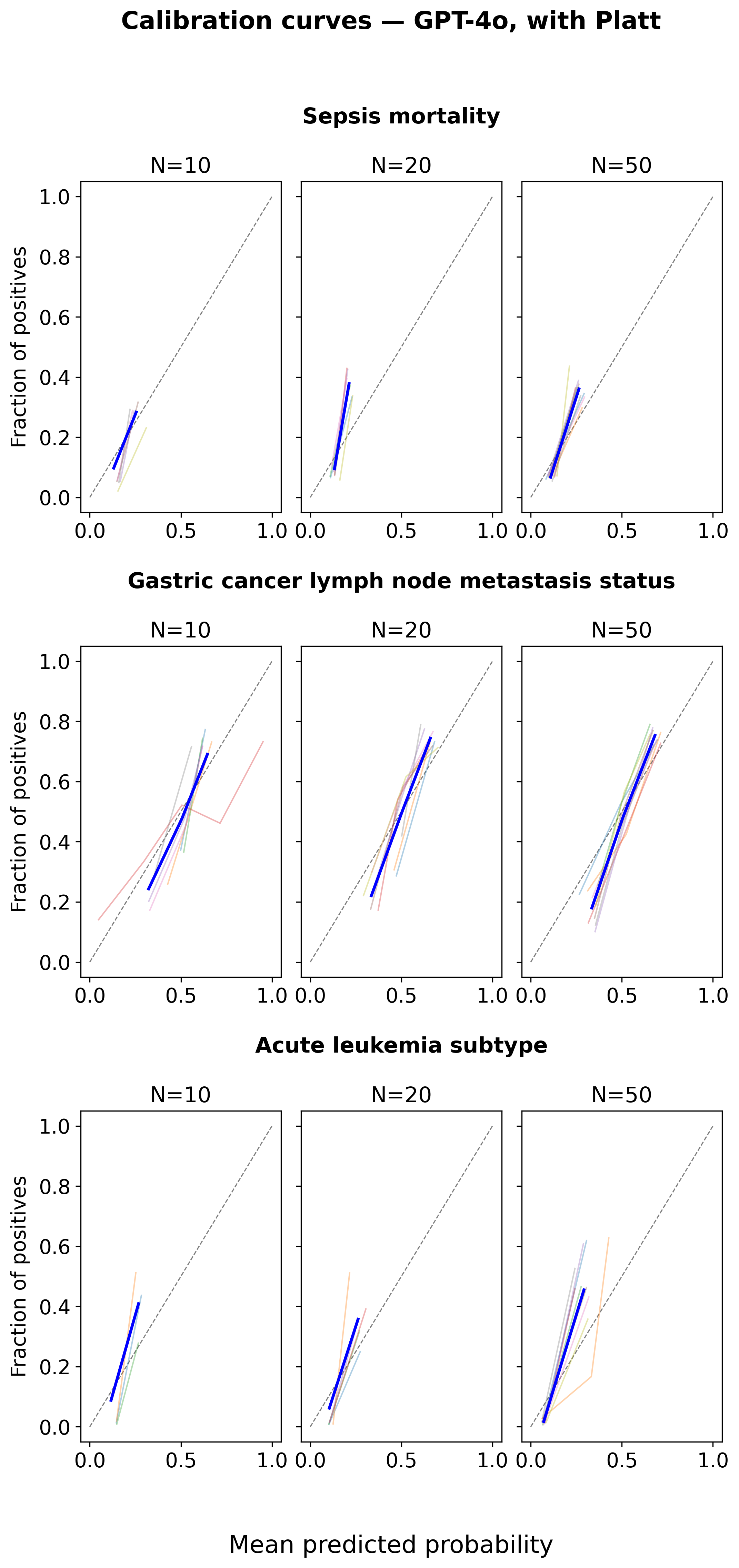


Figure S61: Calibration curves using 5 equal-width bins for GPT-4o with Platt calibration, for the sepsis (top), gastric cancer (middle), and leukemia (bottom) datasets. The light-colored curves correspond to individual folds, and the blue curve is the mean curve across folds.


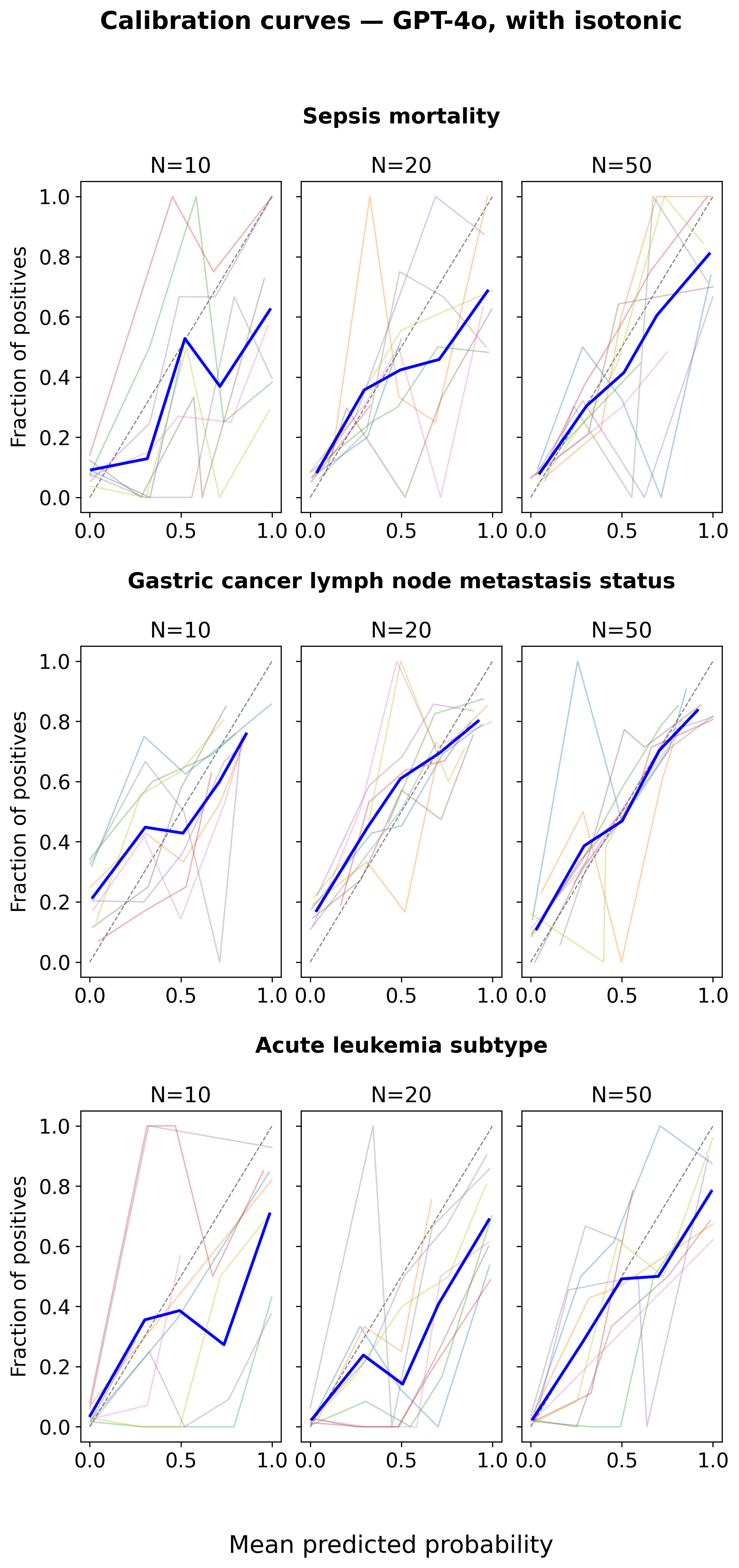


Figure S62: Calibration curves using 5 equal-width bins for GPT-4o with isotonic calibration, for the sepsis (top), gastric cancer (middle), and leukemia (bottom) datasets. The light-colored curves correspond to individual folds, and the blue curve is the mean curve across folds.


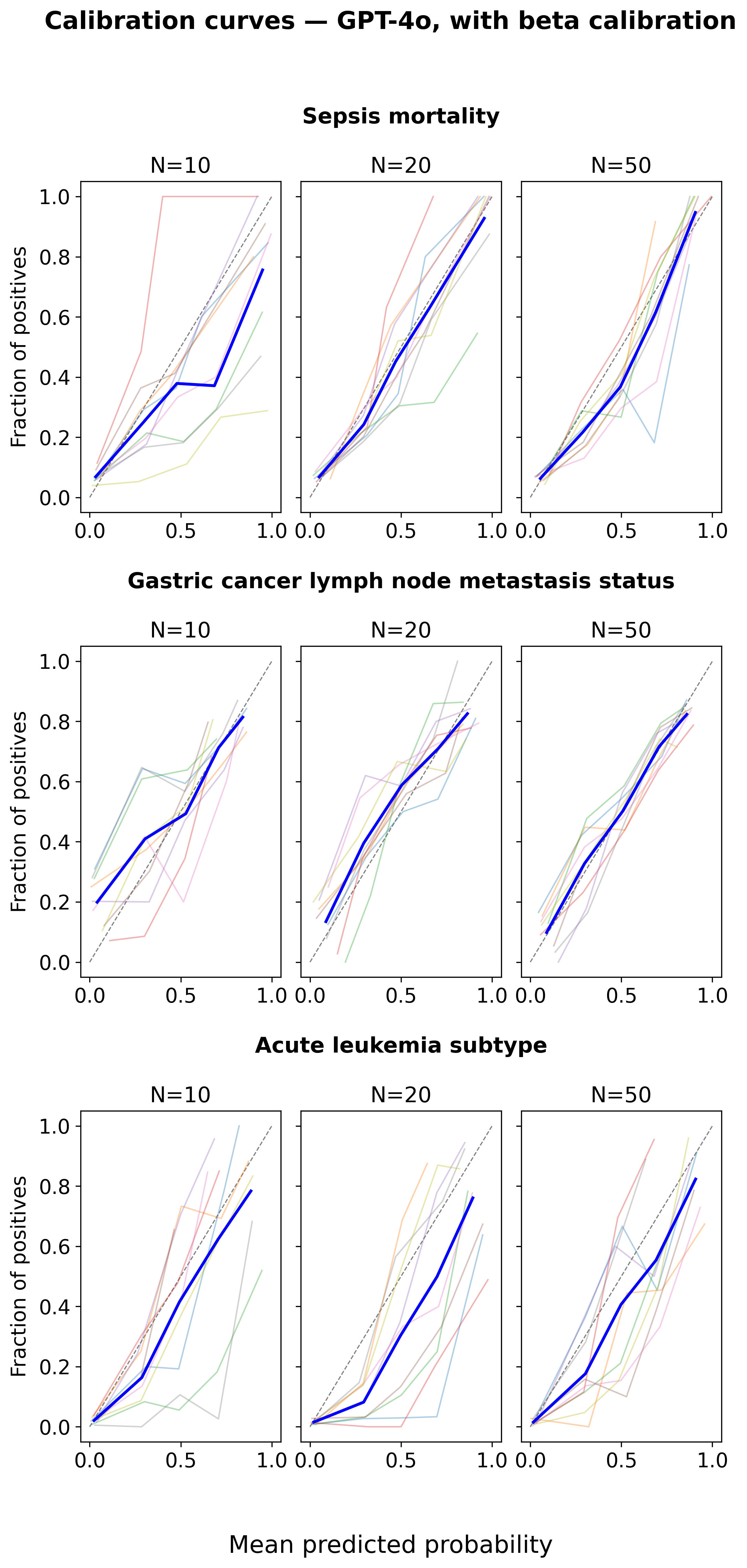


Figure S63: Calibration curves using 5 equal-width bins for GPT-4o with beta calibration, for the sepsis (top), gastric cancer (middle), and leukemia (bottom) datasets. The light-colored curves correspond to individual folds, and the blue curve is the mean curve across folds.

Calibration curves

Conventional ML


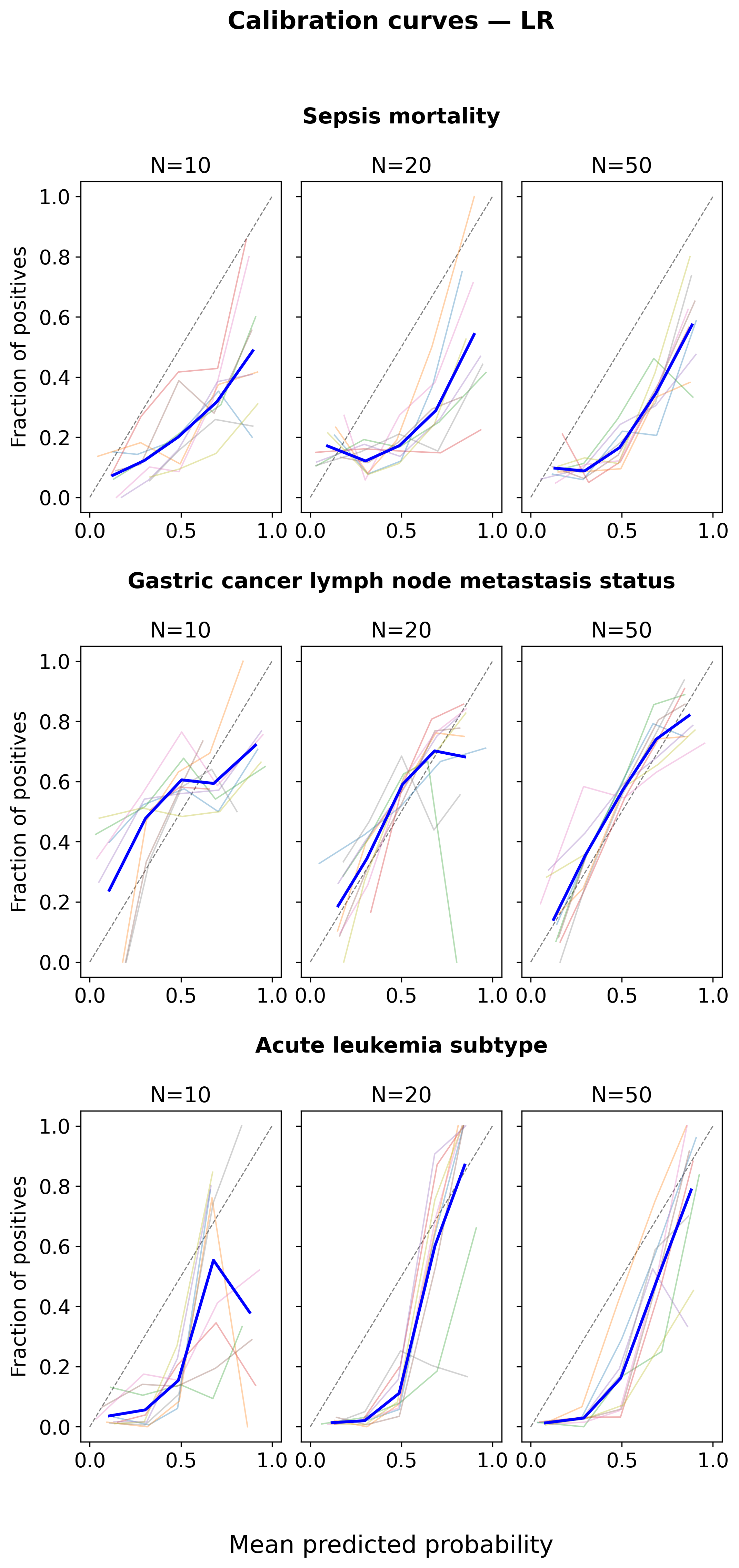


Figure S64: Calibration curves using 5 equal-width bins for Logistic Regression (LR) without calibration, for the sepsis (top), gastric cancer (middle), and leukemia (bottom) datasets. The light-colored curves correspond to individual folds, and the blue curve is the mean curve across folds.


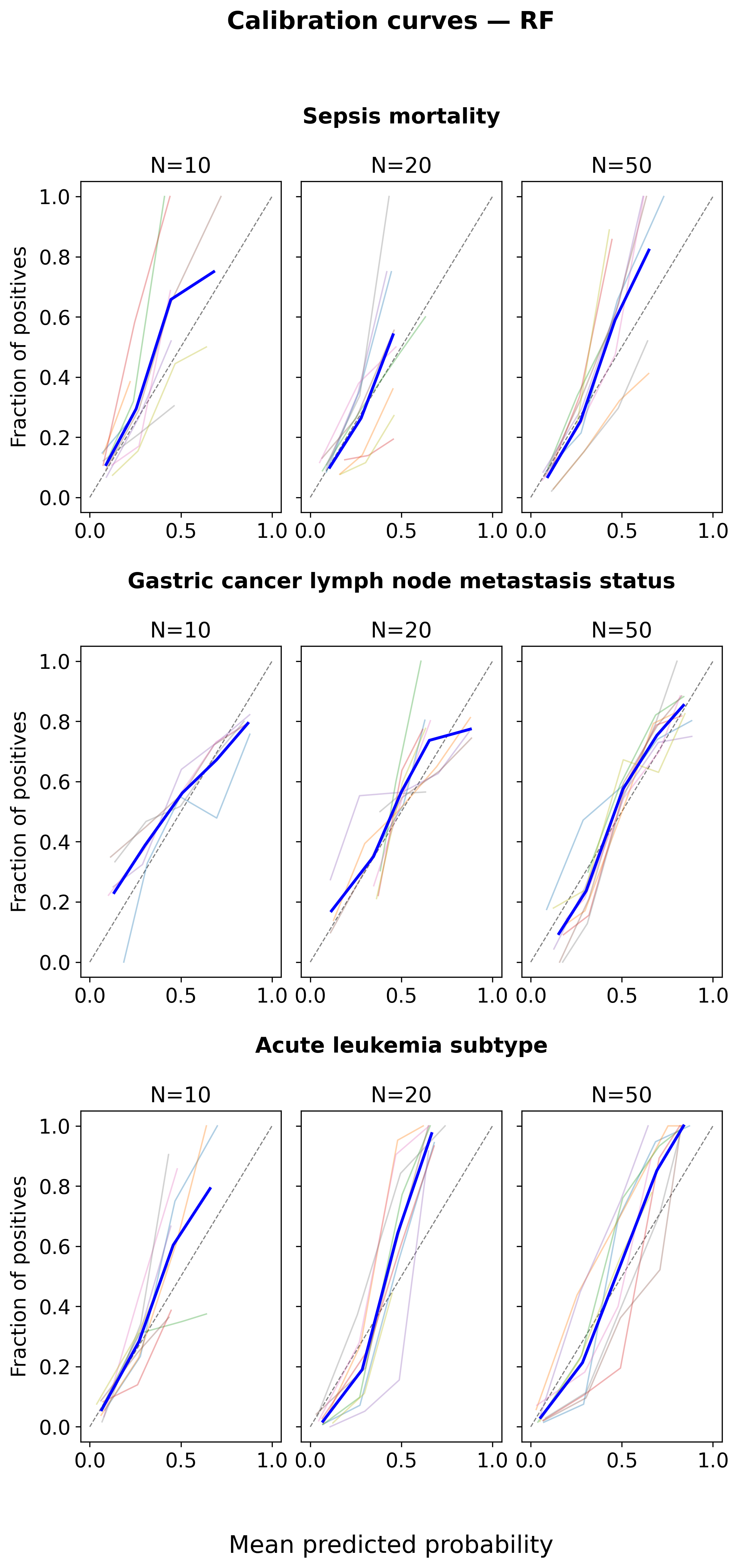


Figure S65: Calibration curves using 5 equal-width bins for Random Forest (RF) without calibration, for the sepsis (top), gastric cancer (middle), and leukemia (bottom) datasets. The light-colored curves correspond to individual folds, and the blue curve is the mean curve across folds.


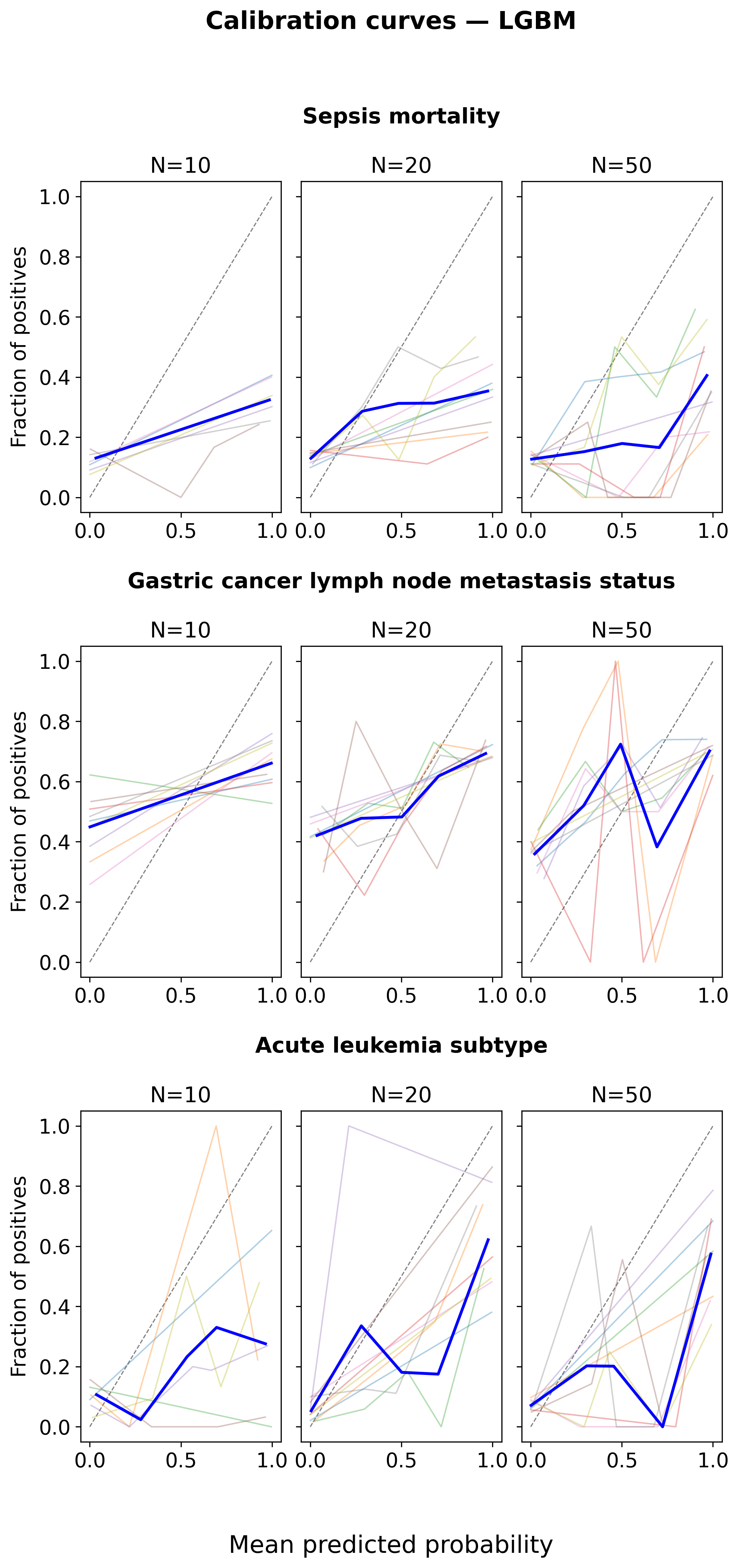


Figure S66: Calibration curves using 5 equal-width bins for LGBM without calibration, for the sepsis (top), gastric cancer (middle), and leukemia (bottom) datasets. The light-colored curves correspond to individual folds, and the blue curve is the mean curve across folds.


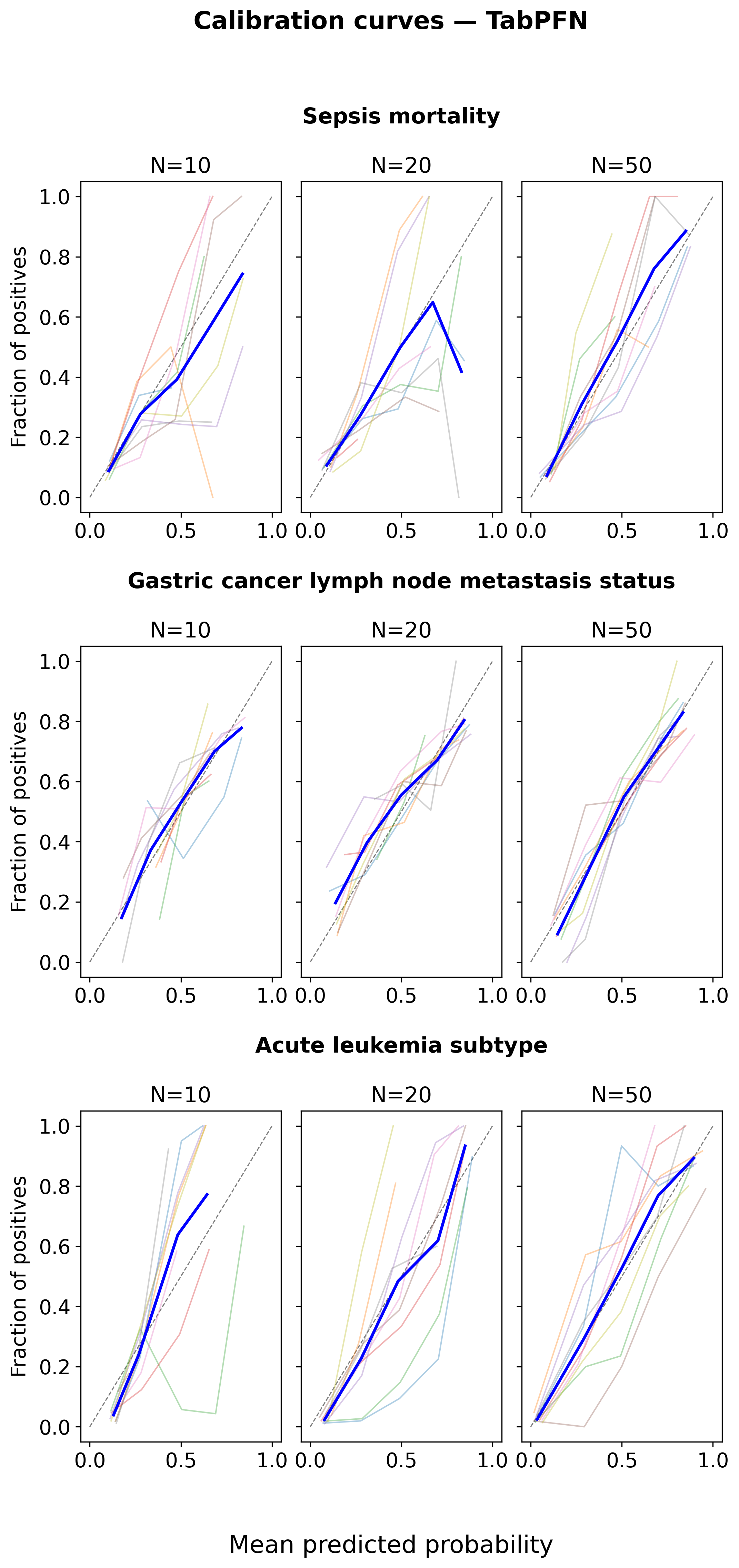


Figure S67: Calibration curves using 5 equal-width bins for TabPFN without calibration, for the sepsis (top), gastric cancer (middle), and leukemia (bottom) datasets. The light-colored curves correspond to individual folds, and the blue curve is the mean curve across folds.


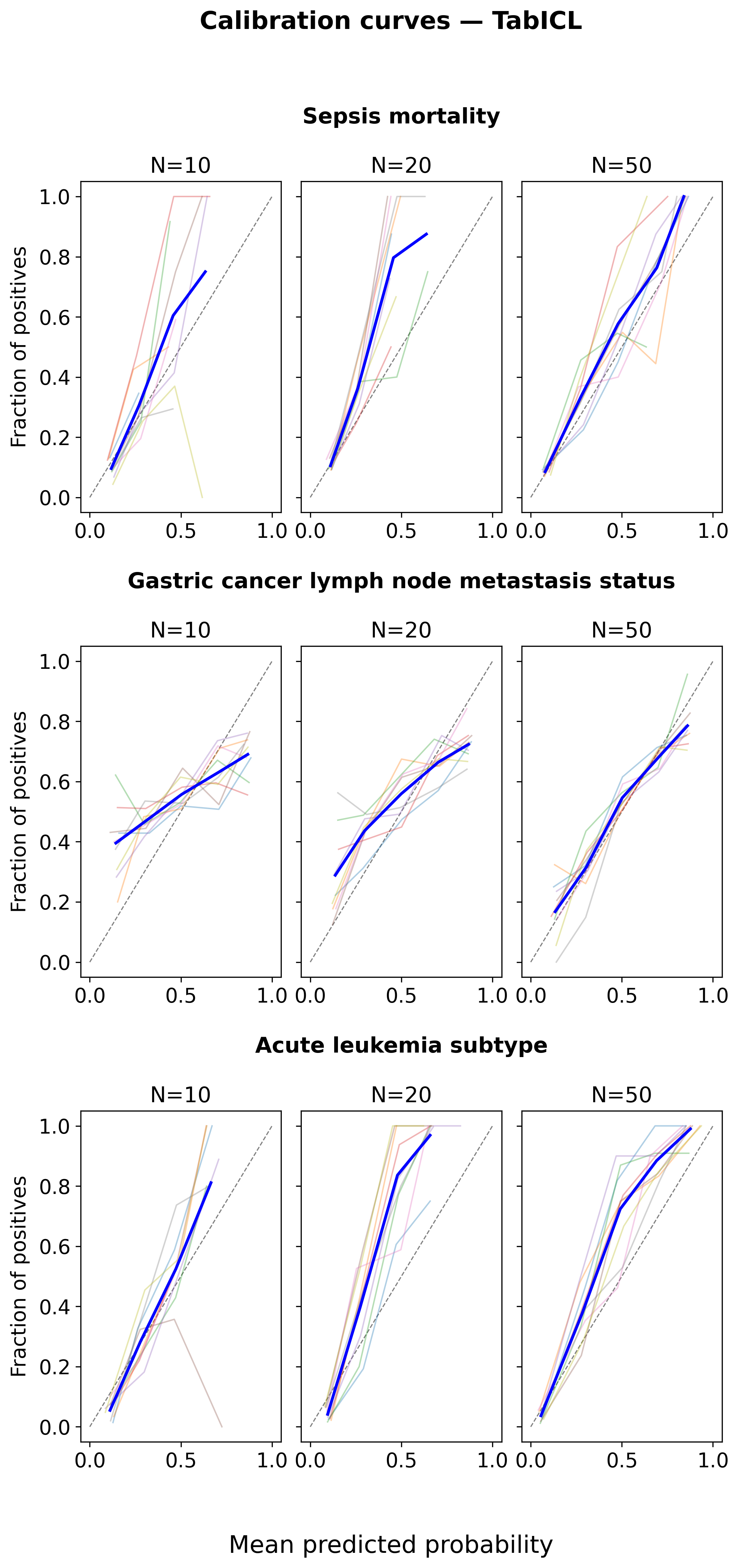


Figure S68: Calibration curves using 5 equal-width bins for TabICL without calibration, for the sepsis (top), gastric cancer (middle), and leukemia (bottom) datasets. The light-colored curves correspond to individual folds, and the blue curve is the mean curve across folds.

Decision curves

Llama 3 70B


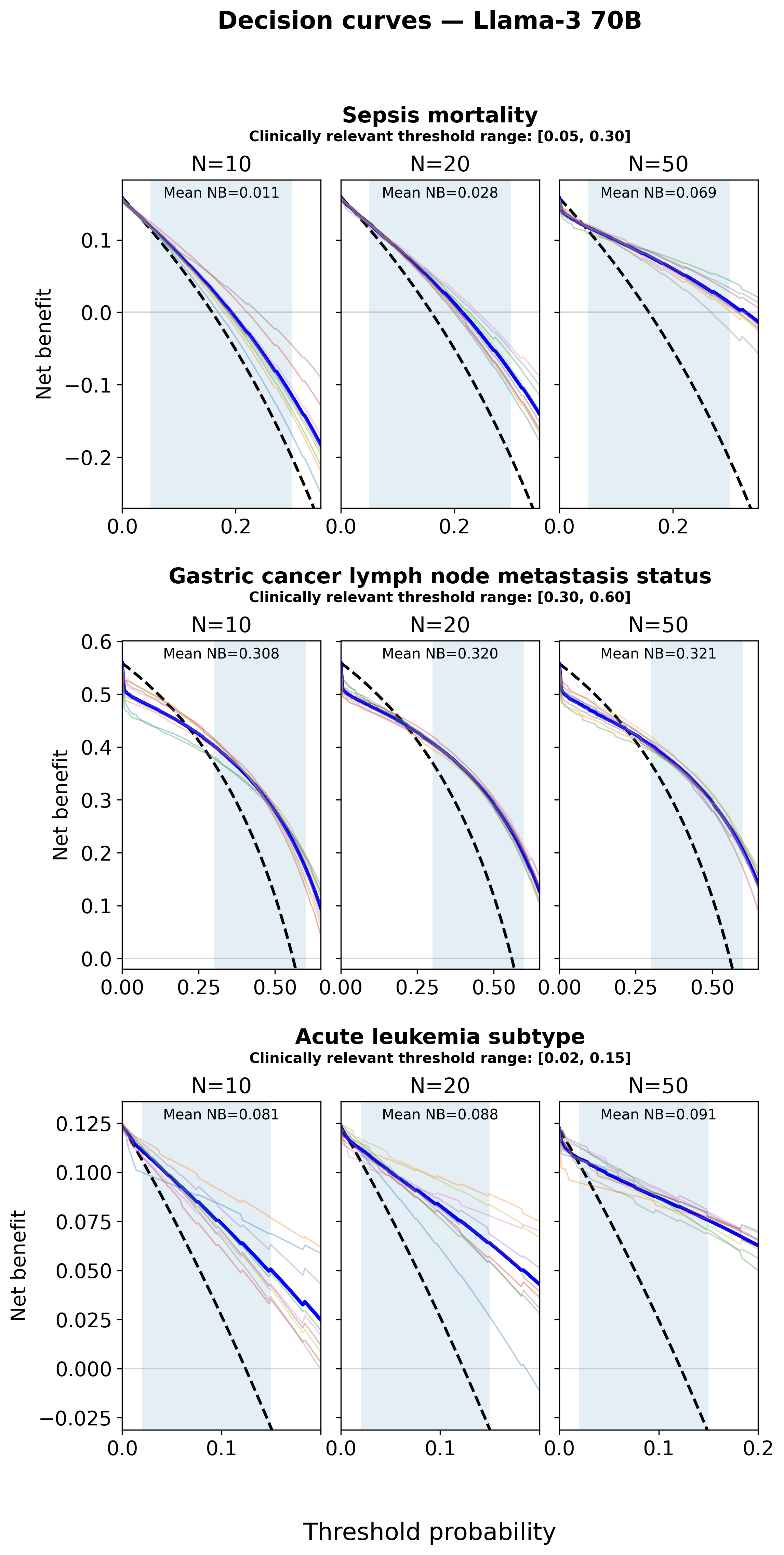


Figure S69: DCA net benefit curves for Llama 3 70B without calibration, for the sepsis (top), gastric cancer (middle), and leukemia (bottom) datasets. The light-colored curves correspond to individual folds, and the blue curve is the mean curve across folds. The black dashed line corresponds to ‘treat all’, and the thin horizontal line to ‘treat none’. The shaded area spans the clinically relevant threshold interval, over which the mean net benefit is computed.


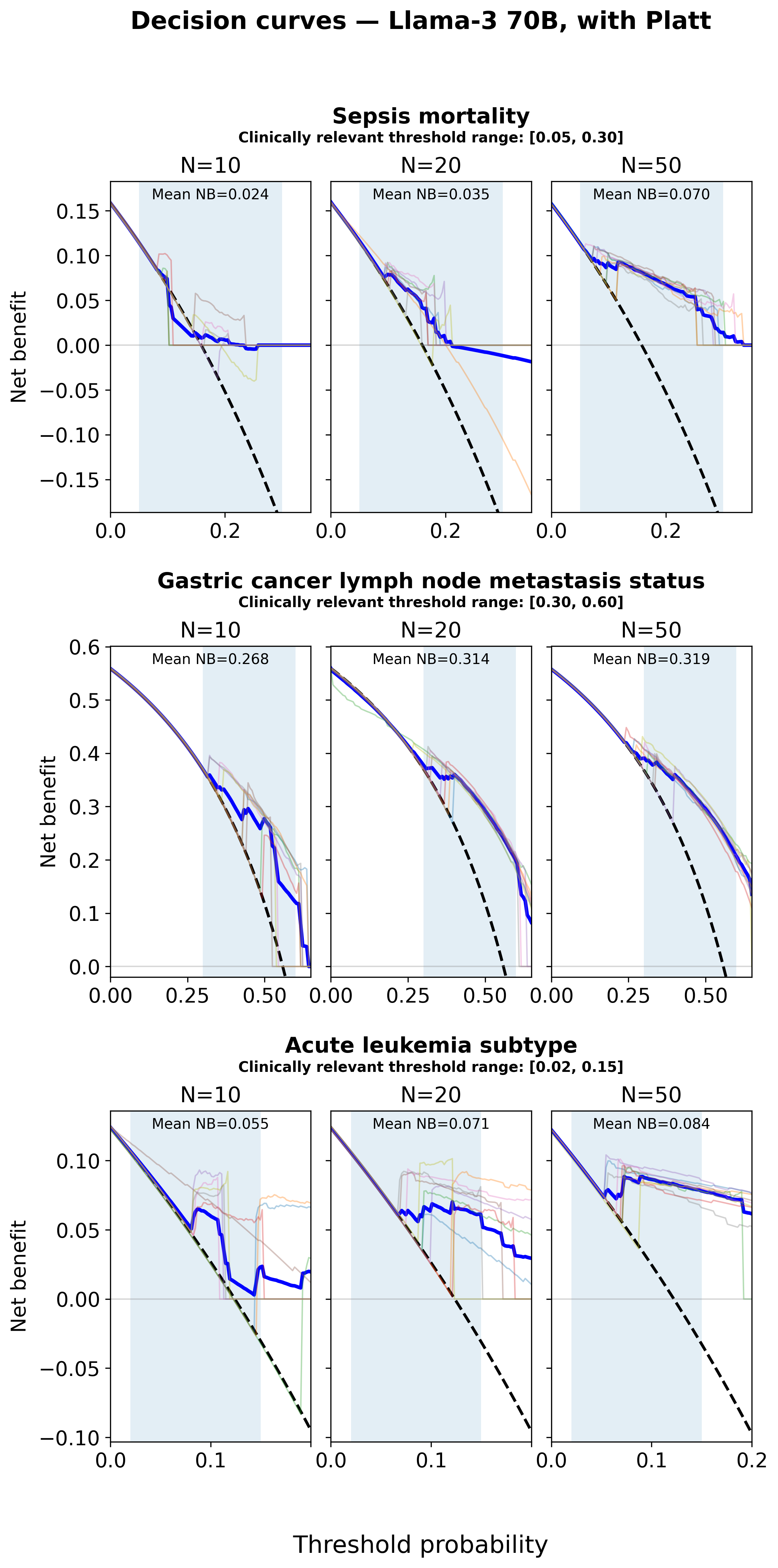


Figure S70: DCA net benefit curves for Llama 3 70B with Platt calibration, for the sepsis (top), gastric cancer (middle), and leukemia (bottom) datasets. The light-colored curves correspond to individual folds, and the blue curve is the mean curve across folds. The black dashed line corresponds to ‘treat all’, and the thin horizontal line to ‘treat none’. The shaded area spans the clinically relevant threshold interval, over which the mean net benefit is computed.


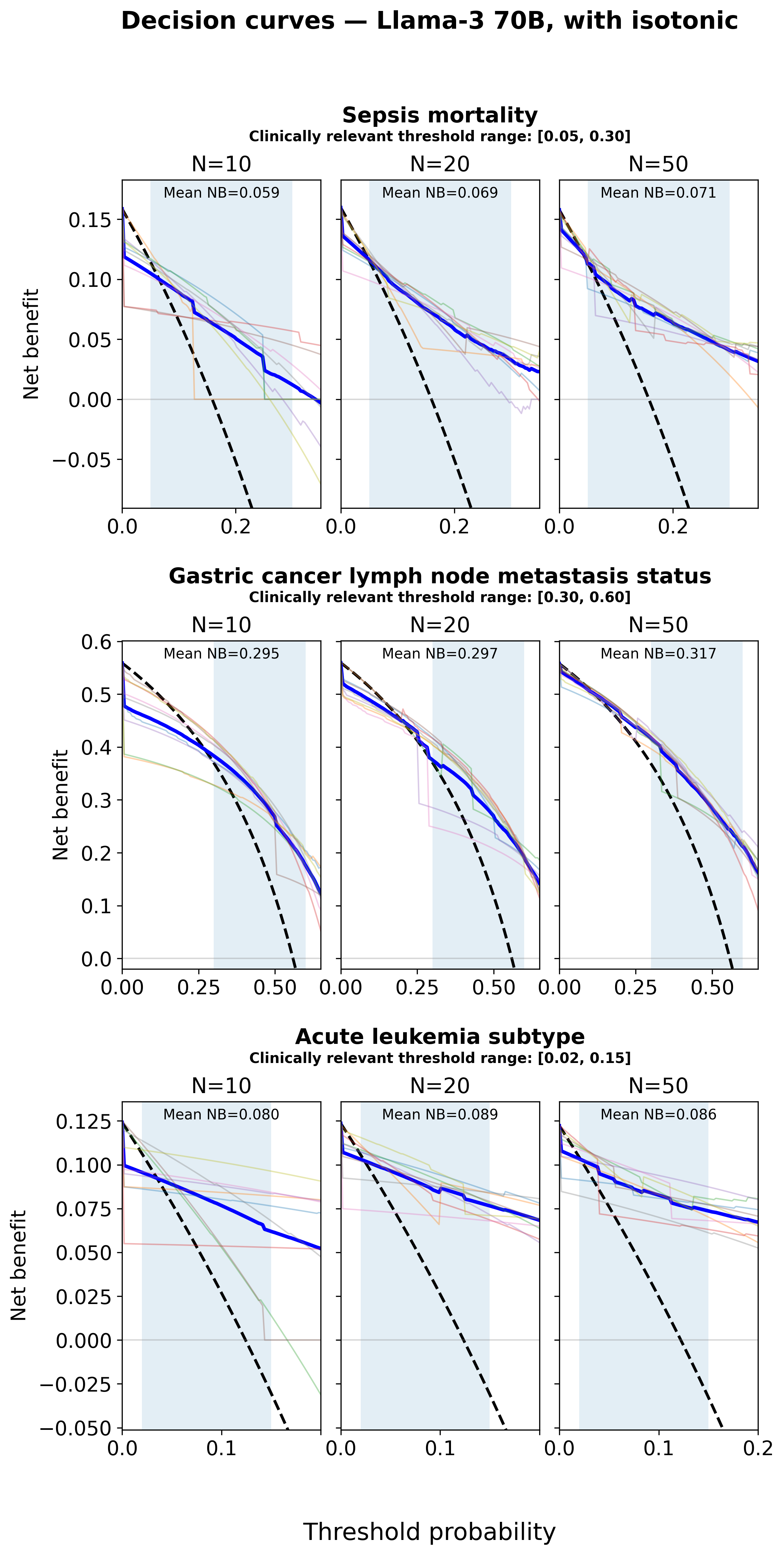


Figure S71: DCA net benefit curves for Llama 3 70B with isotonic calibration, for the sepsis (top), gastric cancer (middle), and leukemia (bottom) datasets. The light-colored curves correspond to individual folds, and the blue curve is the mean curve across folds. The black dashed line corresponds to ‘treat all’, and the thin horizontal line to ‘treat none’. The shaded area spans the clinically relevant threshold interval, over which the mean net benefit is computed.


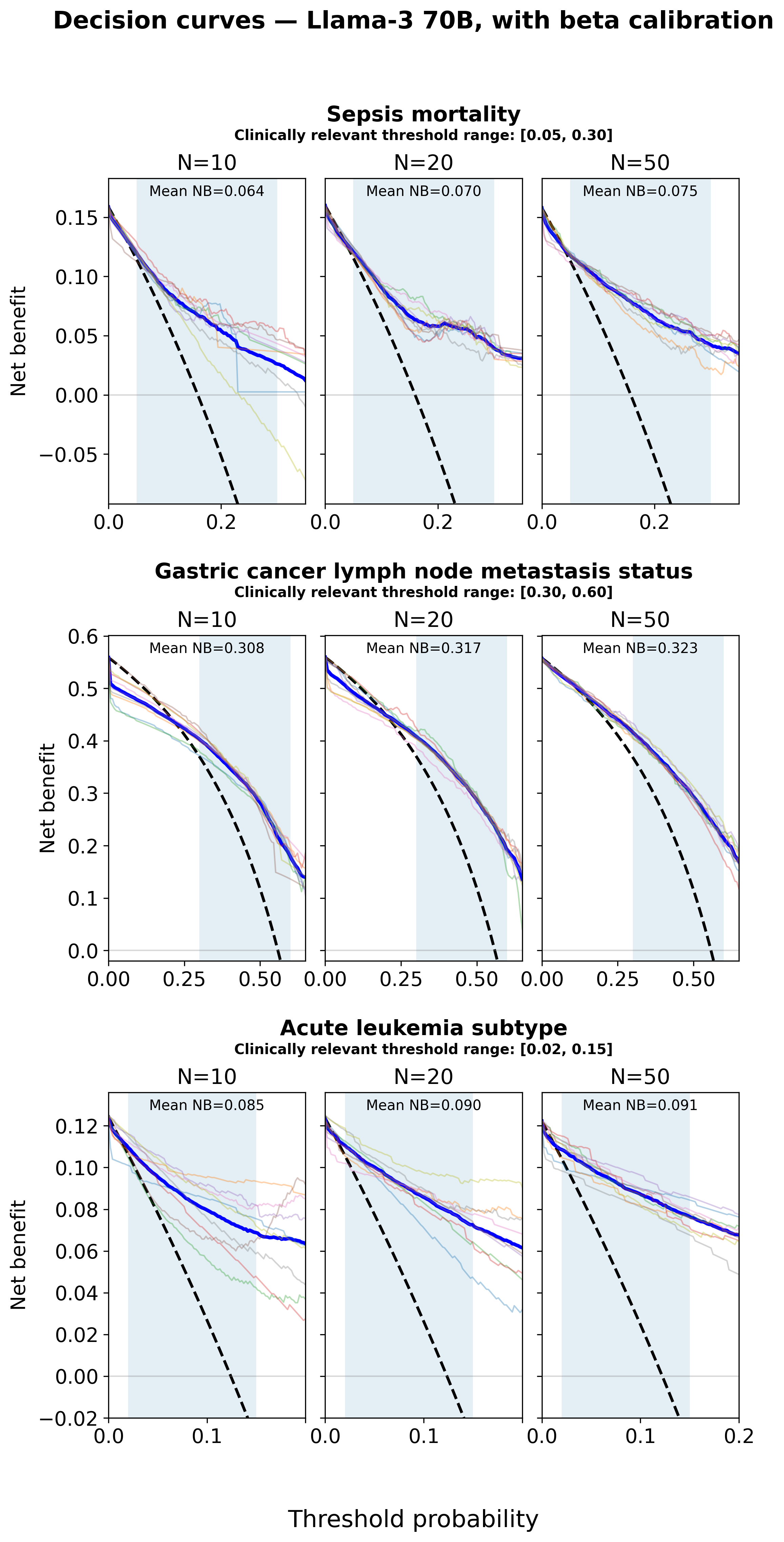


Figure S72: DCA net benefit curves for Llama 3 70B with beta calibration, for the sepsis (top), gastric cancer (middle), and leukemia (bottom) datasets. The light-colored curves correspond to individual folds, and the blue curve is the mean curve across folds. The black dashed line corresponds to ‘treat all’, and the thin horizontal line to ‘treat none’. The shaded area spans the clinically relevant threshold interval, over which the mean net benefit is computed.

Decision curves

GPT-4o


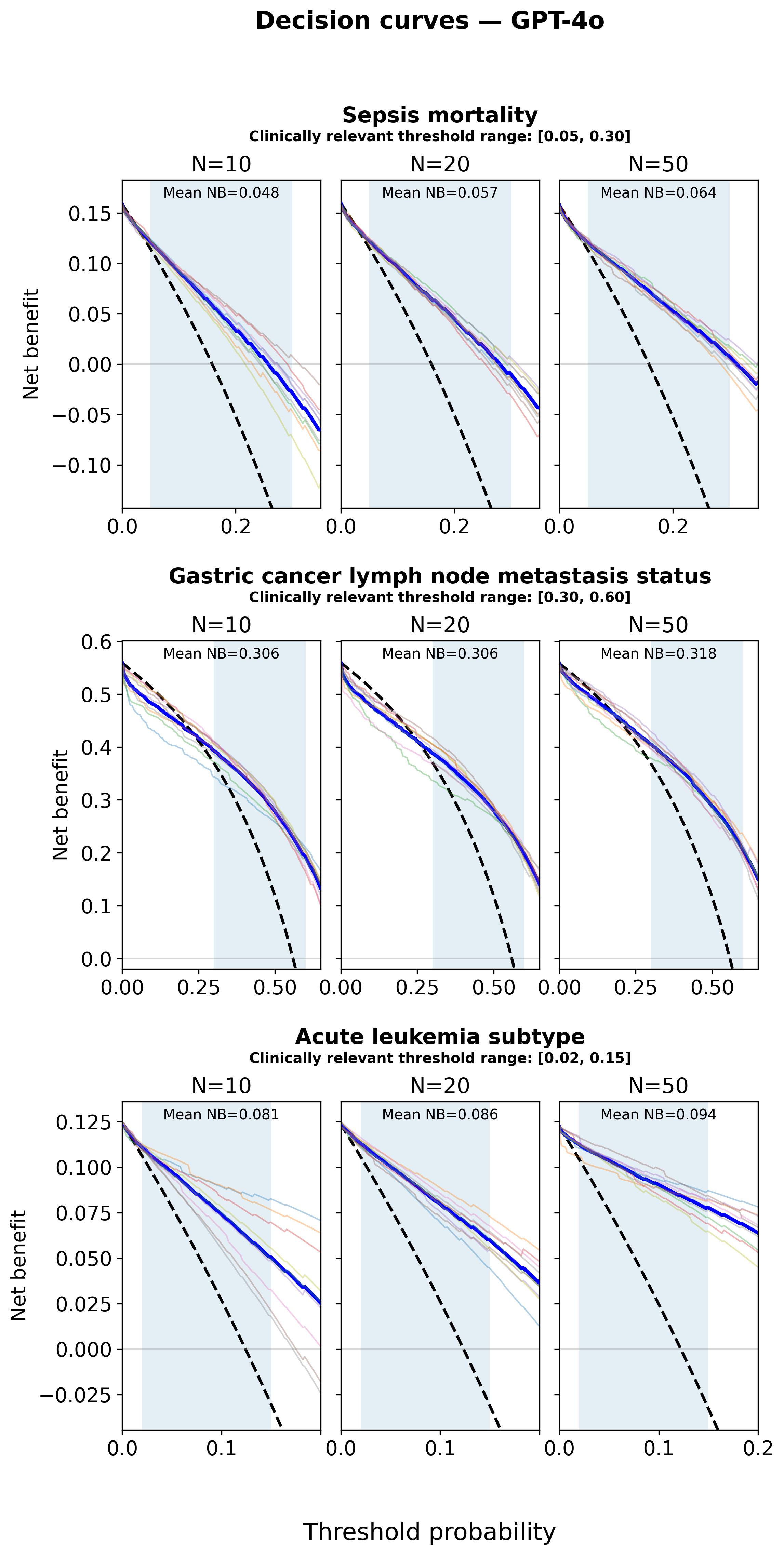


Figure S73: DCA net benefit curves for GPT-4o without calibration, for the sepsis (top), gastric cancer (middle), and leukemia (bottom) datasets. The light-colored curves correspond to individual folds, and the blue curve is the mean curve across folds. The black dashed line corresponds to ‘treat all’, and the thin horizontal line to ‘treat none’. The shaded area spans the clinically relevant threshold interval, over which the mean net benefit is computed.


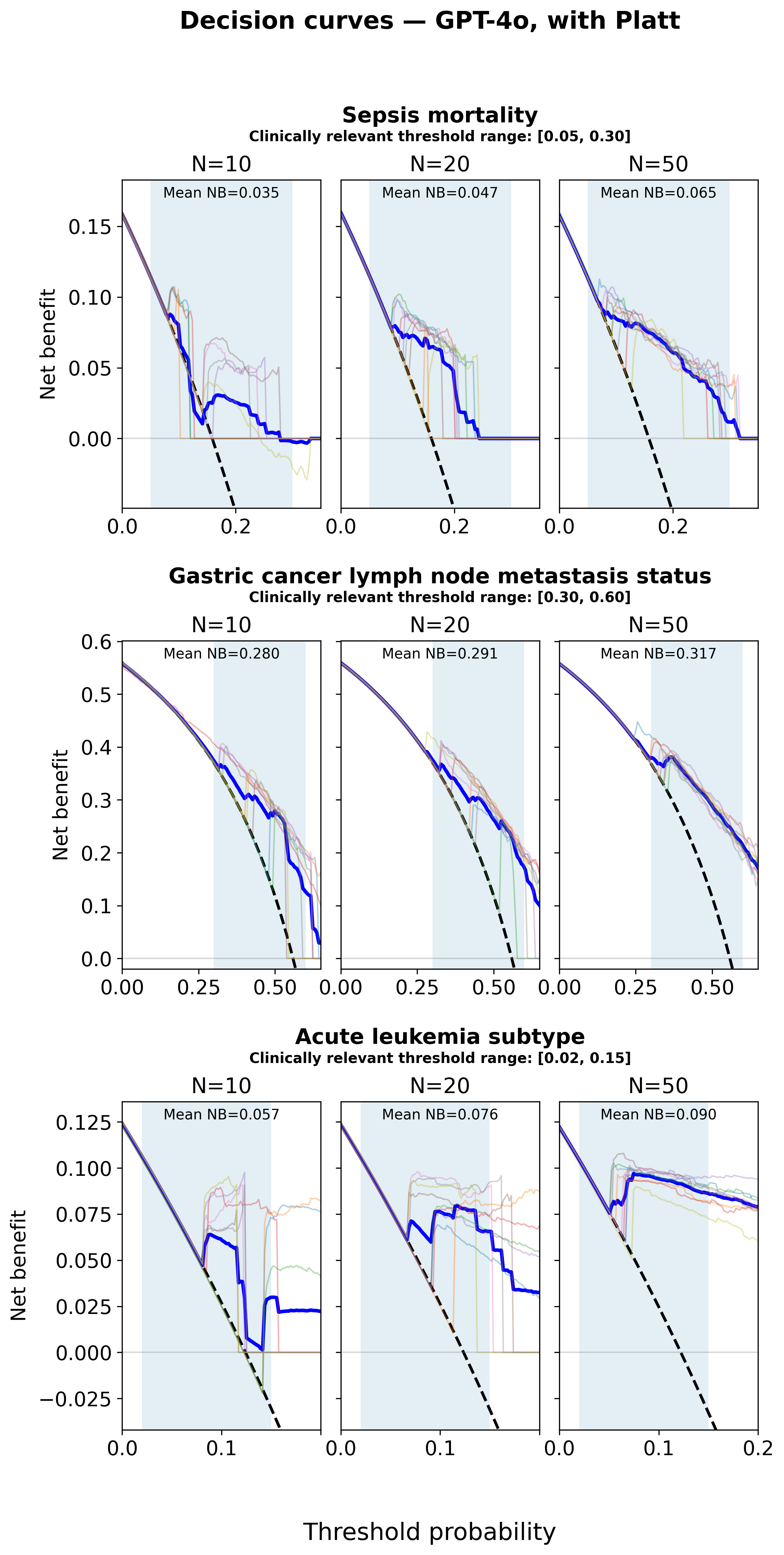


Figure S74: DCA net benefit curves for GPT-4o with Platt calibration, for the sepsis (top), gastric cancer (middle), and leukemia (bottom) datasets. The light-colored curves correspond to individual folds, and the blue curve is the mean curve across folds. The black dashed line corresponds to ‘treat all’, and the thin horizontal line to ‘treat none’. The shaded area spans the clinically relevant threshold interval, over which the mean net benefit is computed.


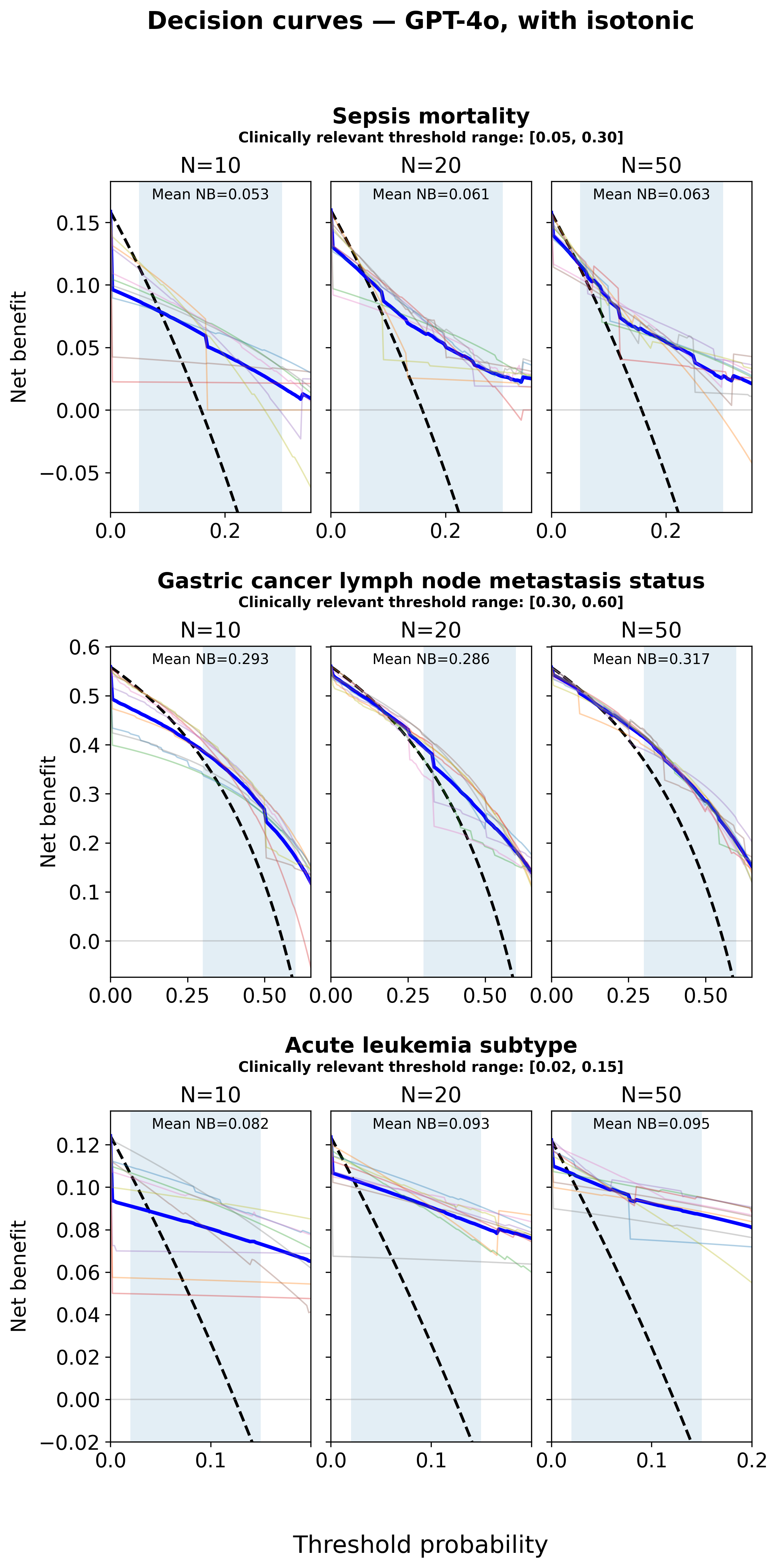


Figure S75: DCA net benefit curves for GPT-4o with isotonic calibration, for the sepsis (top), gastric cancer (middle), and leukemia (bottom) datasets. The light-colored curves correspond to individual folds, and the blue curve is the mean curve across folds. The black dashed line corresponds to ‘treat all’, and the thin horizontal line to ‘treat none’. The shaded area spans the clinically relevant threshold interval, over which the mean net benefit is computed.


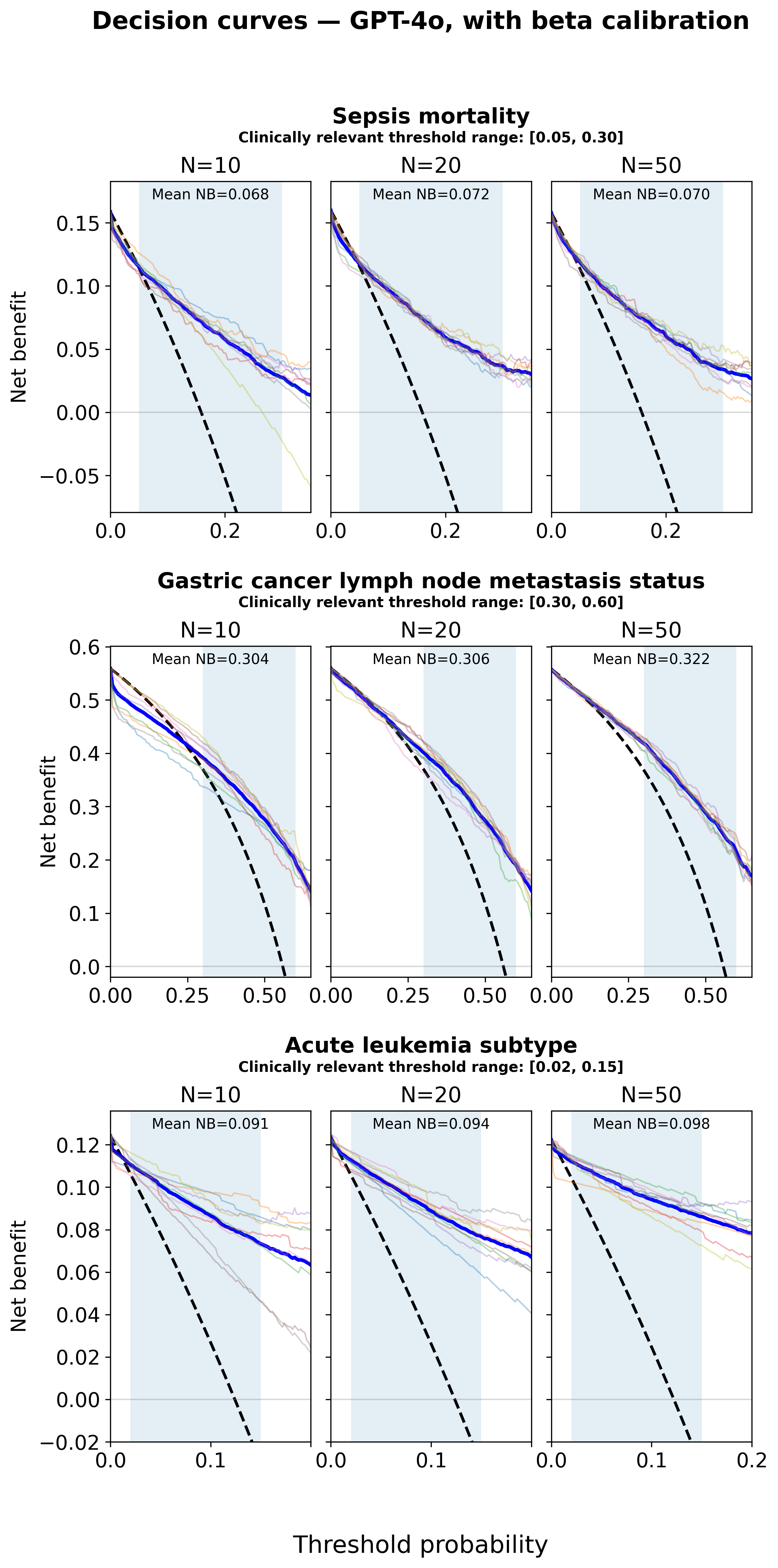


Figure S76: DCA net benefit curves for GPT-4o with beta calibration, for the sepsis (top), gastric cancer (middle), and leukemia (bottom) datasets. The light-colored curves correspond to individual folds, and the blue curve is the mean curve across folds. The black dashed line corresponds to ‘treat all’, and the thin horizontal line to ‘treat none’. The shaded area spans the clinically relevant threshold interval, over which the mean net benefit is computed.

Decision curves

Conventional ML


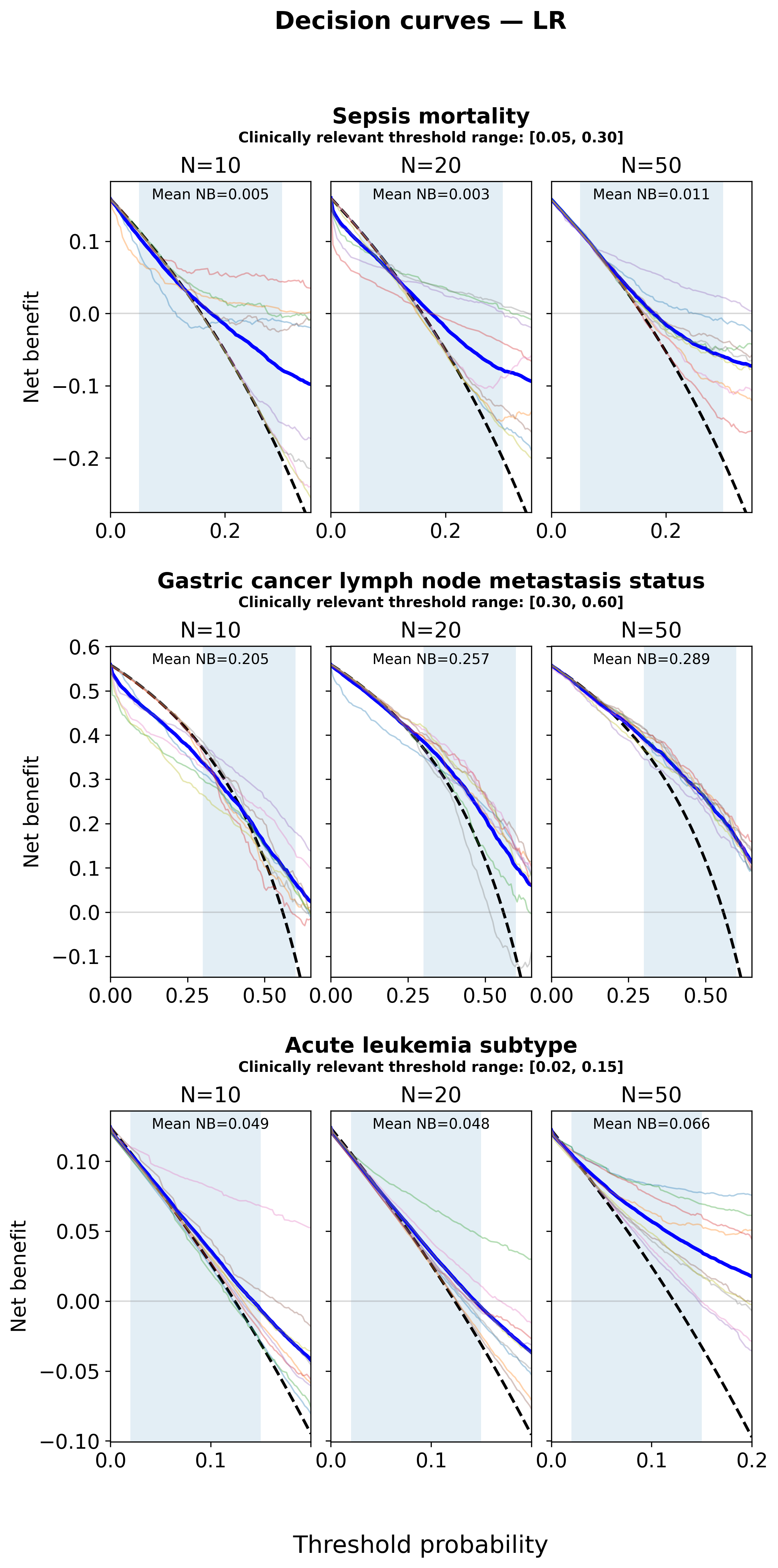


Figure S77: DCA net benefit curves for Logistic Regression (LR) without calibration, for the sepsis (top), gastric cancer (middle), and leukemia (bottom) datasets. The light-colored curves correspond to individual folds, and the blue curve is the mean curve across folds. The black dashed line corresponds to ‘treat all’, and the thin horizontal line to ‘treat none’. The shaded area spans the clinically relevant threshold interval, over which the mean net benefit is computed.


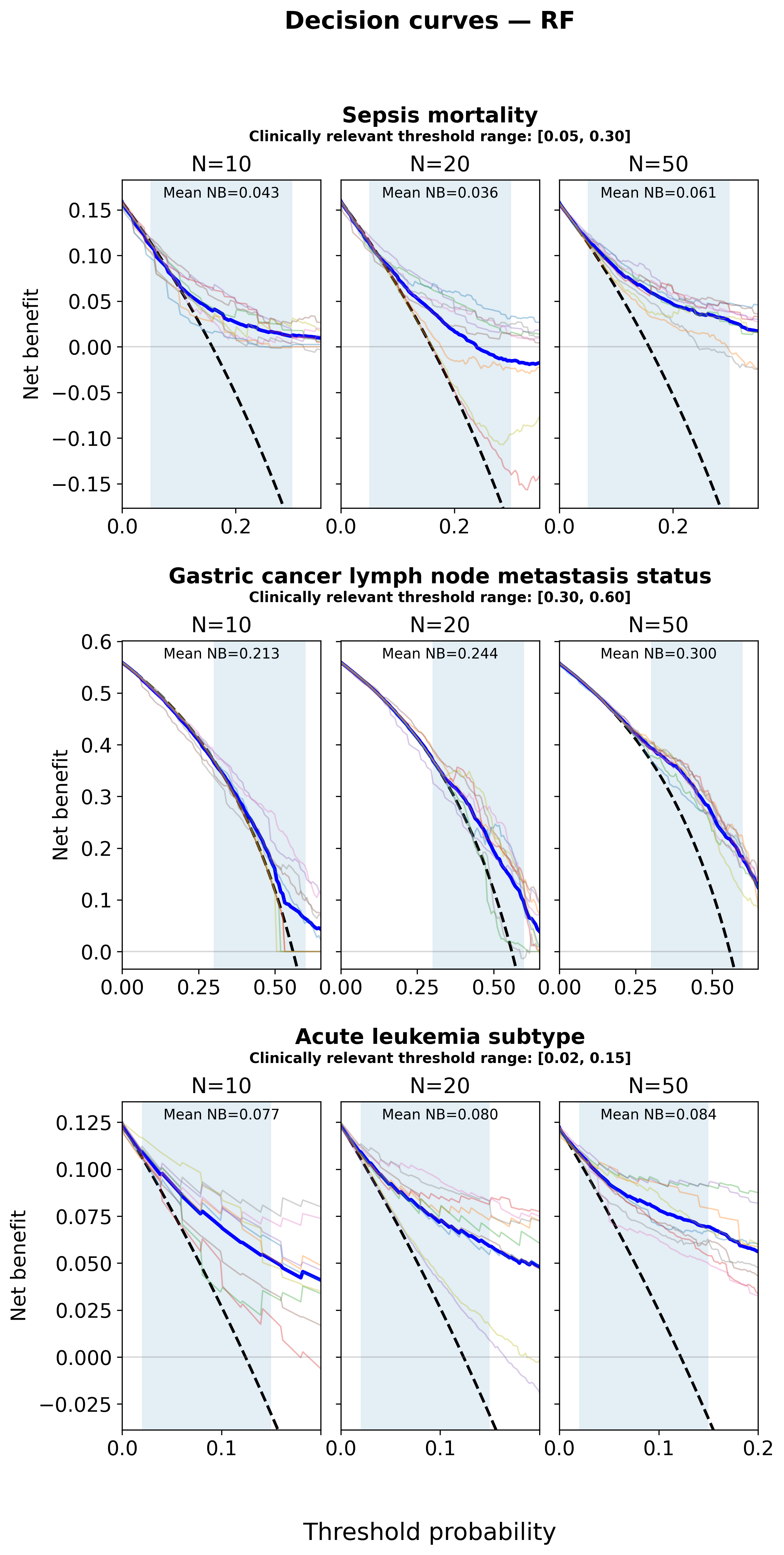


Figure S78: DCA net benefit curves for Random Forest (RF) without calibration, for the sepsis (top), gastric cancer (middle), and leukemia (bottom) datasets. The light-colored curves correspond to individual folds, and the blue curve is the mean curve across folds. The black dashed line corresponds to ‘treat all’, and the thin horizontal line to ‘treat none’. The shaded area spans the clinically relevant threshold interval, over which the mean net benefit is computed.


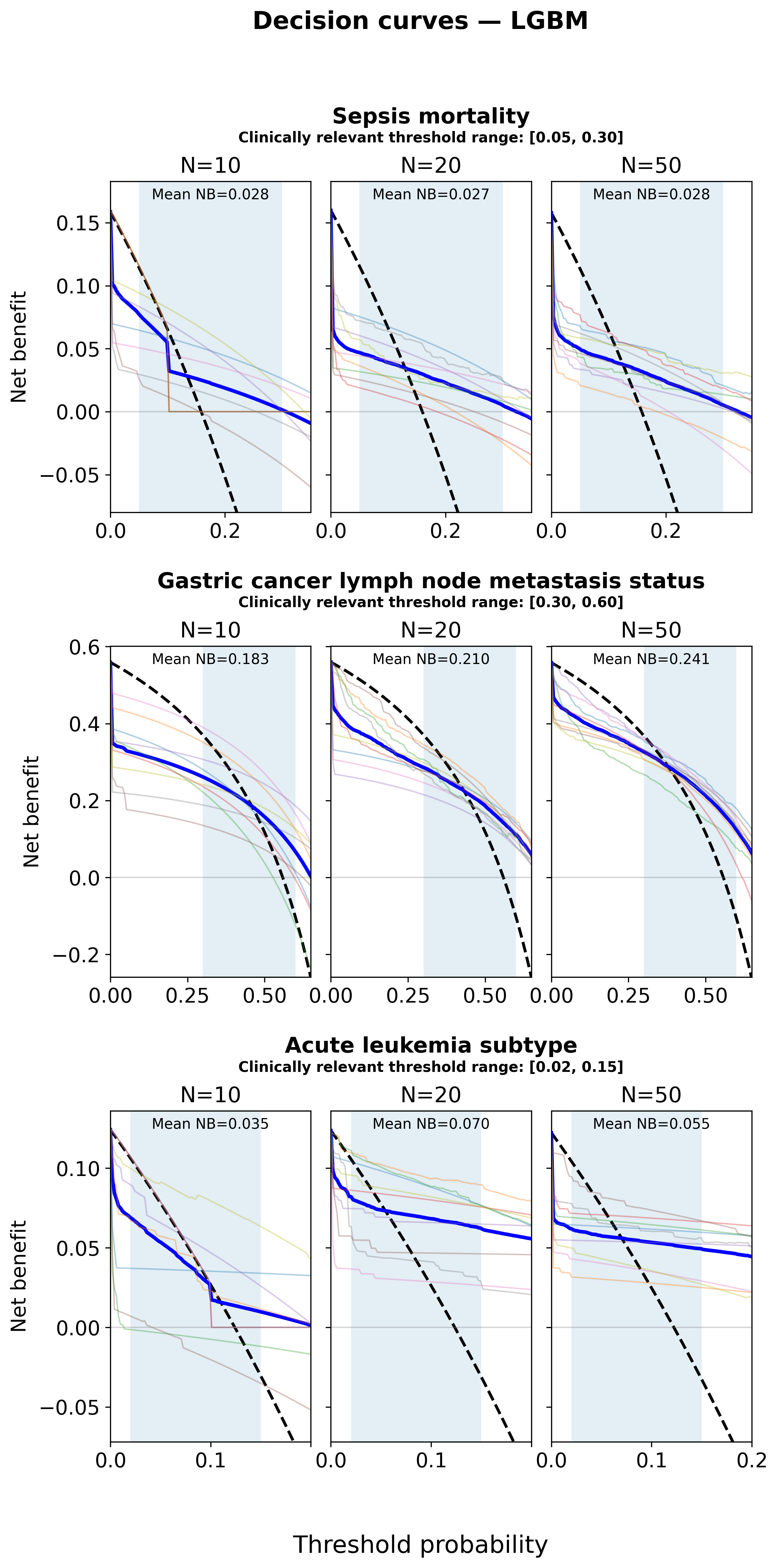


Figure S79: DCA net benefit curves for LGBM without calibration, for the sepsis (top), gastric cancer (middle), and leukemia (bottom) datasets. The light-colored curves correspond to individual folds, and the blue curve is the mean curve across folds. The black dashed line corresponds to ‘treat all’, and the thin horizontal line to ‘treat none’. The shaded area spans the clinically relevant threshold interval, over which the mean net benefit is computed.


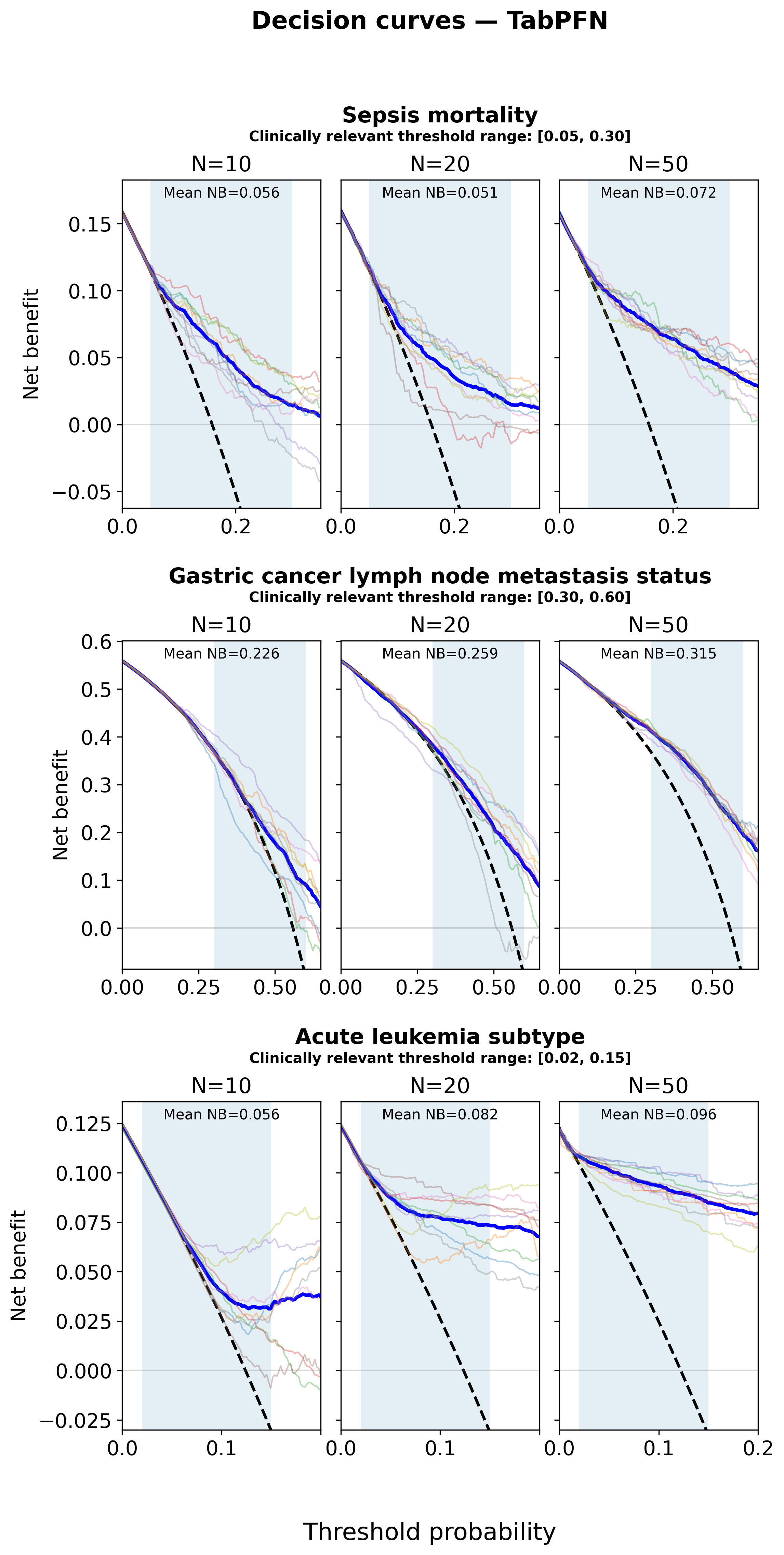


Figure S80: DCA net benefit curves for TabPFN without calibration, for the sepsis (top), gastric cancer (middle), and leukemia (bottom) datasets. The light-colored curves correspond to individual folds, and the blue curve is the mean curve across folds. The black dashed line corresponds to ‘treat all’, and the thin horizontal line to ‘treat none’. The shaded area spans the clinically relevant threshold interval, over which the mean net benefit is computed.


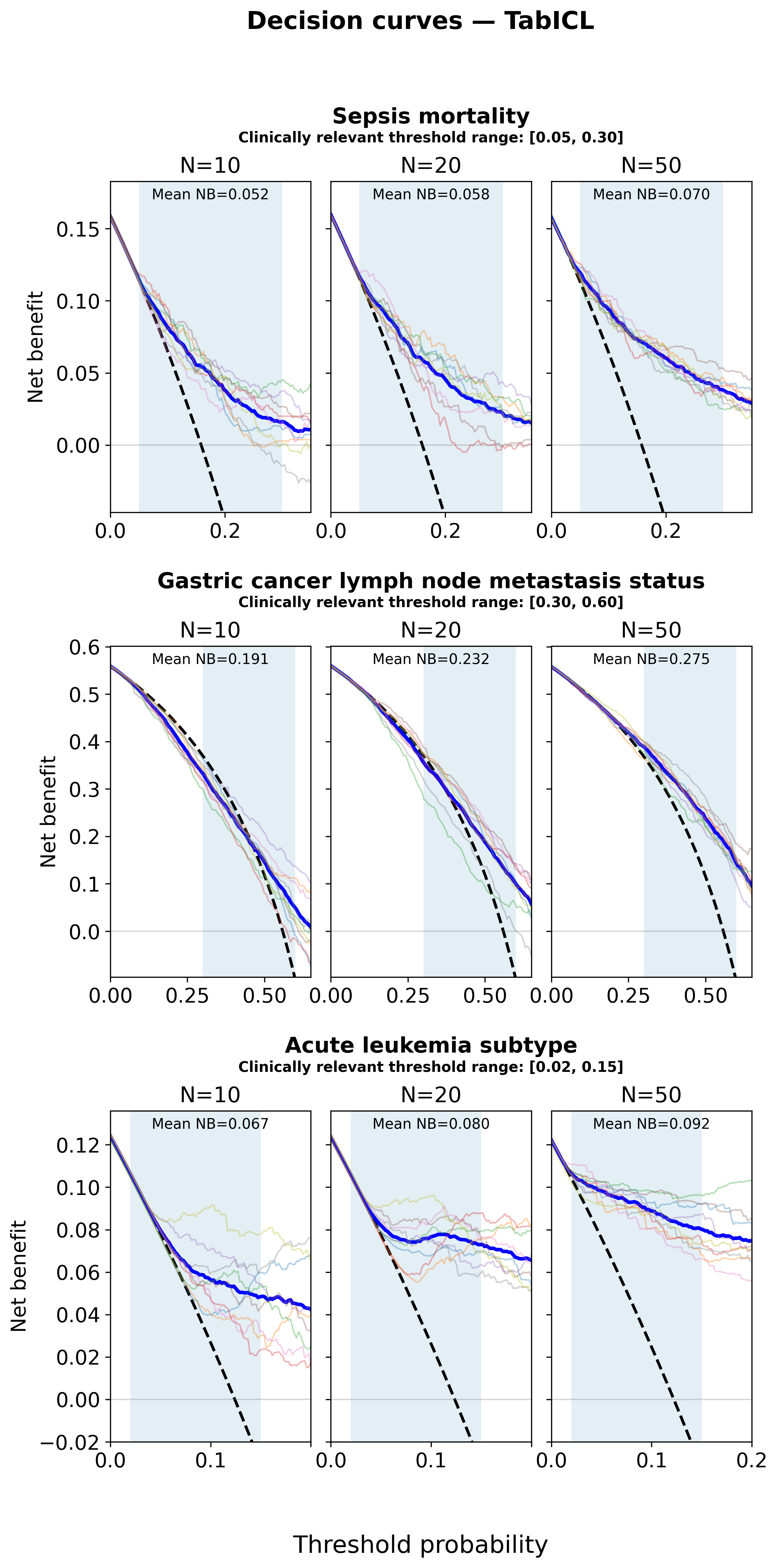


Figure S81: DCA net benefit curves for TabICL without calibration, for the sepsis (top), gastric cancer (middle), and leukemia (bottom) datasets. The light-colored curves correspond to individual folds, and the blue curve is the mean curve across folds. The black dashed line corresponds to ‘treat all’, and the thin horizontal line to ‘treat none’. The shaded area spans the clinically relevant threshold interval, over which the mean net benefit is computed.
